# Systematic review of antimicrobial pharmacokinetic/pharmacodynamic indices in murine thigh and hollow fibre dose fractionation studies analysed with a standard method

**DOI:** 10.1093/jac/dkaf446

**Published:** 2025-12-17

**Authors:** Najla Alabdulkarim, John Readman, Andrew Mead, Robert Oakley, Suzanne Wenker, Japhette Kembou, Stefano Azzariti, Mona Bajaj-Elliott, Mario Cortina-Borja, Joseph F Standing

**Affiliations:** Great Ormond Street Institute of Child Health, University College London, London, UK; Department of Clinical Pharmacy, Princess Nourah Bint Abdulrahman University, Riyadh, Saudi Arabia; PK/PD of Anti-Infectives Study Group of the European Society of Clinical Microbiology and Infectious Diseases, Basel, Switzerland; Great Ormond Street Institute of Child Health, University College London, London, UK; Department of Comparative Biomedical Science, The Royal Veterinary College, London, UK; PK/PD of Anti-Infectives Study Group of the European Society of Clinical Microbiology and Infectious Diseases, Basel, Switzerland; Department of Pharmacy, St George’s University Hospitals NHS Foundation Trust, London, UK; Great Ormond Street Institute of Child Health, University College London, London, UK; Department of Clinical Pharmacy and Toxicology, Leiden University, Leiden, Netherlands; Great Ormond Street Institute of Child Health, University College London, London, UK; Department of Comparative Biomedical Science, The Royal Veterinary College, London, UK; Great Ormond Street Institute of Child Health, University College London, London, UK; Population, Policy and Practice Research and Teaching Department, UCL Great Ormond Street Institute of Child Health, London, UK; Great Ormond Street Institute of Child Health, University College London, London, UK; PK/PD of Anti-Infectives Study Group of the European Society of Clinical Microbiology and Infectious Diseases, Basel, Switzerland; Department of Pharmacy, Great Ormond Street Hospital for Children, London, UK

## Abstract

**Background:**

Pre-clinical models are commonly used to determine human antibiotic dosage regimens using pharmacokinetic/pharmacodynamic (PKPD) indices. The murine thigh infection model (MTIM) is most commonly used for PKPD index determination, while the hollow fibre infection model (HFIM) may be a viable alternative. However, there is no standardized method for determining the PKPD index and *R*^2^ may not be the ideal metric to determine goodness of fit for nonlinear models. This study aimed to reanalyse PKPD indices published in MTIM and HFIM, using a standardized modelling approach.

**Methods:**

Systematic literature review was conducted to identify MTIM and HFIM dose fractionation studies. Searches covered databases including PubMed, MEDLINE, BIOSIS, SCOPUS and EMBASE. Data were extracted and modelled using eight variations of Emax model, with model selection based on the lowest Akaike information criterion (AIC) and parameter plausibility in terms of precision and interpretability.

**Results:**

A total of 53 studies were included: 50 MTIM (of 1138) and 3 HFIM (of 316). Among the 53 studies, reporting issues included an infrequent use of AIC for model selection as applied in only one paper, and a lack of methodological transparency in 29 papers. Remodelling revealed some disagreement in optimal PKPD indices in six studies.

**Conclusions:**

This study suggests a standard method for PKPD index model selection and provides a database on PKPD index analysis. Building the Emax model from one to four estimated parameters and assessing them with AIC is recommended to avoid over fitting. Too few HFIM dose fractionation studies were found to allow comparison of PKPD index with MTIM.

## Introduction

Antimicrobial resistance (AMR) is a growing global health crisis that compromises the effectiveness of antibiotics, rendering common infections more difficult to treat and increasing the risk of severe illness and death.^1–4^ AMR is primarily caused by antibiotic misuse and overuse, highlighting the urgent need to optimize antimicrobial dosage regimens.^5,6^ Setting optimal doses is essential for both eradicating pathogens and mitigating the risk of resistance. To achieve this, it is essential to understand and apply pharmacokinetic/pharmacodynamic (PKPD) principles that guide dosing regimen design.

The PKPD index helps quantify the relationship between drug exposure and bacterial effect by using pharmacokinetic metrics, usually AUC, *C*_max_ and time above a threshold, in relation to pharmacodynamic parameters, usually MIC.^7,8^ This relationship is often described using an Emax model, which captures the concentration-effect curve. There are eight variations of the Emax model, in which certain parameters (*E0*, *EC50*, Emax or *γ*) are estimated or fixed.^9,10^ Emax model selection is typically guided by information criteria such as Akaike information criterion (AIC) and Bayesian information criterion (BIC),^11,12^ which provide a more robust assessment of model fit than the coefficient of determination *R*².^9,13^ Although commonly used in pharmaceutical literature, *R*² is not suitable for evaluating goodness of fit in nonlinear regression models, such as Emax model, due to its low sensitivity against poorly fitted models and its tendency to increase with model complexity, leading to overfitting.^9,10^ As the goodness of fit naturally increases with the number of parameters, both AIC and BIC penalize the observed likelihood value with the number of parameters. BIC includes a stronger penalization term than AIC and can lead to more conservative model selection especially when the number of parameters is large. Furthermore, because there is no exact solution to the best fit of a nonlinear model, model building, testing models with different combinations of estimated and fixed parameters is necessary to avoid over fitting and to ensure that the fitting algorithm adequately converges. Unlike *R*^2^, AIC values do not lie on an interval and should be used only to establish preference across competing models.

The most commonly used PKPD indices are AUC/MIC, *C*_max_/MIC and *T* > MIC.^14,15^ The choice of PKPD index depends on the antibiotic’s pharmacodynamic properties. For example, with concentration-dependent antibiotics, where higher drug concentrations result in more rapid bacterial killing, the *C*_max_/MIC ratio is most relevant.^16,17^ Once the appropriate PKPD index is determined, the next step is to use PKPD modelling to simulate different dosing regimens and predict their outcomes, helping to establish the optimal PKPD target value for effective therapy.^7^

PKPD index is determined through dose fractionation studies often employing the murine thigh infection model (MTIM). The hollow fibre infection model (HFIM) offers a promising alternative to traditional *in vivo* models. The HFIM offers control over PK and experimental conditions, allowing flexible testing of multiple organisms and dose levels.^18^ While the absence of immune cells can be seen as a limitation, it also allows for a clear assessment of antibiotic effects without immune interference. However, drawbacks include the use of synthetic media and the possibility of drug adhering to the plastic components, which could affect the overall drug concentration and results.^18^

PKPD indices derived from MTIM and HFIM are generally applicable to humans, as they capture species-independent drug–pathogen interactions. When modelling these indices, delays between drug concentration and bacterial killing are typically not included, since the modelled effect already reflects the overall antibacterial effect.

Although MTIM and HFIM enable the evaluation of PKPD indices, determining these indices still poses some challenges. Compared with the long history of antibiotic use, the application of PKPD indices to guide dosing strategies is relatively recent. As a result, it has not been explored for all antibiotics. In addition, the absence of standardized guidelines for performing and reporting PKPD index analyses may lead to inconsistencies and potential gaps in the data. These challenges may impede the effective application of PKPD indices in combating AMR.

Herein, the primary aim was to systematically review and evaluate PKPD indices derived from studies using the MTIM and HFIM. Rather than relying on a single model per index, a standardized modelling framework was applied to test all eight Emax variants within each index, with the best from each then used to choose between indices.

## Methods

### Study protocol

The systematic review adhered to the Preferred Reporting Items for Systematic Review and Meta-analysis (PRISMA) statement.^19^ The review protocol was registered with the International Prospective Register of Systematic Reviews (PROSPERO) under registration number CRD42022375851, which can be accessed at the following link: https://www.crd.york.ac.uk/prospero/display_record.php?RecordID=375851.

### Data sources and search strategy

A systematic search of the published literature from January 1980 to June 2025 was conducted using the following electronic databases: PubMed, MEDLINE, BIOSIS, SCOPUS and EMBASE. Furthermore, reference checking, Google Scholar and the *Oxford Journal of Infectious Diseases* were searched for relevant publications. The search was conducted twice for each database, once for manuscripts that used the MTIM and once for those that used the HFIM. With respect to the latter, the search strategy was 2-fold. Initially, it incorporated bibliography from the systematic review published by Sadouki *et al*., covering from January 1980 to January 2020.^20^ Subsequently, the search was extended to include publications from January 2020 to June 2025.

In the search terms, keywords included PKPD index, antibiotic, MTIM and HFIM. These were refined and systematically combined according to the requirements of each database. Information on the search strategy can be found in Tables S1 and S2 (available as Supplementary data at *JAC* Online).

### Eligibility criteria

#### Inclusion criteria

All publications examining the optimal PKPD index for antibiotic efficacy through dose fractionation studies were included if they met the following criteria: (i) used either MTIM or HFIM; (ii) investigated the change in colony-forming units (cfu) for the three PKPD indices (AUC/MIC, *C*_max_/MIC, *T* > MIC) either as numerical values or as plots and (iii) conducted dose fractionation study for a duration of 24 hours. Antibiotics were defined as substances that either kill bacteria or inhibit their growth. There were no restrictions on the type of antibiotic used.

#### Exclusion criteria

Exclusion criteria included: (i) not written in English, (ii) did not use a MTIM or HFIM during dose fractionation, (iii) experiments lasting longer than 24 hours and (iv) administered a drug that was not injectable. In addition, abstracts, short communications, oral presentations or letters were excluded. Studies were also excluded if they did not provide sufficient details for all three PKPD indices (AUC/MIC, *C*_max_/MIC and *T* > MIC), either in the form of plots or tables. In addition, studies that did not use viable bacterial counts (either absolute cfu or changes in cfu) as the PD endpoint were excluded.

### Quality assessment tool

The CAMARADES checklist was used to evaluate studies using the MTIM. There is, however, no standardized method or quality assessment tool for studies conducted with the HFIM. The initial quality assessment by A.M., R.O., S.W., J.K. and S.A. was followed by independent assessment by N.A., and discrepancies were solved by discussion.

### Data extraction

Data extraction was conducted using a prepared Microsoft Excel form to retrieve information from the dose fractionation experiments presented in each manuscript. The extracted data included various aspects, including study characteristics (year, author and journal), the drug under investigation (name, doses, protein binding effects and class), type of performed experiment (MTIM or HFIM), experimental conditions (neutropenic status, inoculum size, route of drug administration), research design (control and treatment groups, timing of treatment initiation, growth media used), primary findings (optimal PKPD index and target values for stasis, 1-log kill, 2-log kill) and data analysis [model fitting methods, parameters estimated, model selection process (e.g. AIC versus *R*²)]. WebPlotDigitizer^21^ were used to extract data from plots showing changes in cfu for each PKPD index. Where there were clearly multiple overlying points by visual inspection or by counting the expected number of points, multiple points were included. The extracted data, which are essential for subsequent modelling, were stored in CSV files corresponding to each publication. Initial data extraction involved six reviewers (J.R., A.M., R.O., S.W., J.K., S.A.). Subsequently, the data were independently extracted from all papers by N.A., which were then cross-checked. Any discrepancies encountered during this process were resolved through discussion with J.F.S.

### Outcome measurement

The primary outcome of this systematic review was to explore the optimal PKPD index and targets associated with efficacy. Secondary outcome included an evaluation and analysis of the methodologies and reporting practices used in the identified studies.

### Data modelling

A standardized R code (version 4.4.1, R Core Team 2024)^22^ was used to fit the Emax model via nonlinear least squares.^23^ This script was then applied to each study individually, ensuring that all datasets were re-analysed using the same modelling framework.

#### Model building

To determine the optimal PKPD index, eight model variants (Table 1) derived from the Emax model (Equation 1) were tested for each index, and the best-fitting model was selected.


(1) $$E=E0-\left(\frac{Emax\times C^{\gamma }}{EC50^{\gamma }+C^{\gamma }}\right)$$

**Table 1.** Models derived from the Emax model for predicting PKPD indices

Formula	Model	Estimated parameter	Fixed parameter	Description
1	$E=E0_{\;fix}-\left(\frac{Emax_{\;fix}\times C}{EC50+C}\right)$	EC50	E0, Emax, ‘γ’	Basic Emax model with estimated EC50, with ‘γ’ set to the value of 1
2	$E=E0_{\;fix}-\left(\frac{Emax_{\;fix}\times \;C^{\gamma }}{EC50^{\gamma }+\;C^{\gamma }}\right)$	EC50, ‘γ’	E0, Emax,	Introduces Hill coefficient ‘γ’ for steepness of curve
3	$E=E0_{\;fix}-\left(\frac{Emax\times C}{EC50+C}\right)$	EC50, Emax	E0, ‘γ’	Emax parameter is estimated; allows variability in maximum achievable effect, with ‘γ’ set to the value of 1
4	$E=E0_{\;fix}-\;\left(\frac{Emax\;\times \;C^{\gamma }}{EC50^{\gamma }+\;C^{\gamma }}\right)$	EC50, Emax, ‘γ’	E0	Introduces ‘γ’ parameter to modify curve shape; captures variation in maximum effects among doses
5	$E=E0-\left(\frac{Emax_{\;fix}\times C}{EC50+C}\right)$	EC50, E0	Emax, ‘γ’	Estimates baseline effect (E0) with fixed maximum effect (Emax) and ‘γ’ set to 1. Suitable for scenarios where maximum effect is constant but baseline effect varies
6	$E=E0-\left(\frac{Emax_{\;fix}\times \;C^{\gamma }}{EC50^{\gamma }+\;C^{\gamma }}\right)$	EC50, E0, ‘γ’	Emax	Introduces ‘γ’ parameter to modify curve shape; suitable for scenarios with varying baseline effects and dose–response nonlinearity
7	$E=E0-\left(\frac{Emax\times C}{EC50+C}\right)$	EC50, E0, Emax	‘γ’	Estimates baseline effect (E0) and maximum effect (Emax), with ‘γ’ set to the value of 1. Suitable for scenarios with changing baseline and maximum effects
8	$E=E0-\left(\frac{Emax\times C^{\gamma }}{EC50^{\gamma }+C^{\gamma }}\right)$	EC50, E0, Emax, ‘γ’	None	Introduces ‘γ’ parameter to modify curve shape; provides information on dose–response nonlinearity

where *E* is the antibiotic effect or response (e.g. the change in bacterial count), *E*0 is the baseline effect (the bacterial concentration when the drug concentration is zero), *Emax* is the maximum effect achievable by the drug, *C* is the drug concentration, *EC*50 is the drug concentration at which the effect is half of Emax and represents the drug’s potency and *γ* is the Hill coefficient, which describes the slope of the concentration-effect curve. It is a measure of how steep the curve is and can be used to characterize the cooperativity or interaction of multiple binding sites or components involved in the response.

#### Criteria for model selection

The model selection procedure was based on minimizing AIC, which assesses goodness of fit while penalizing for model complexity measured by the number of model parameters. If two models had identical AIC values, the model with the highest *R²* value was selected. Models with unrealistic EC50, E0 or Emax estimates beyond the range of experimental data or with poor visual fit were rejected. Following the identification of the most suitable model for each PKPD index, the indices were compared against each other by evaluating their AIC values and visual fit.

## Result

### Study characteristics: search findings

**Figure 1.**
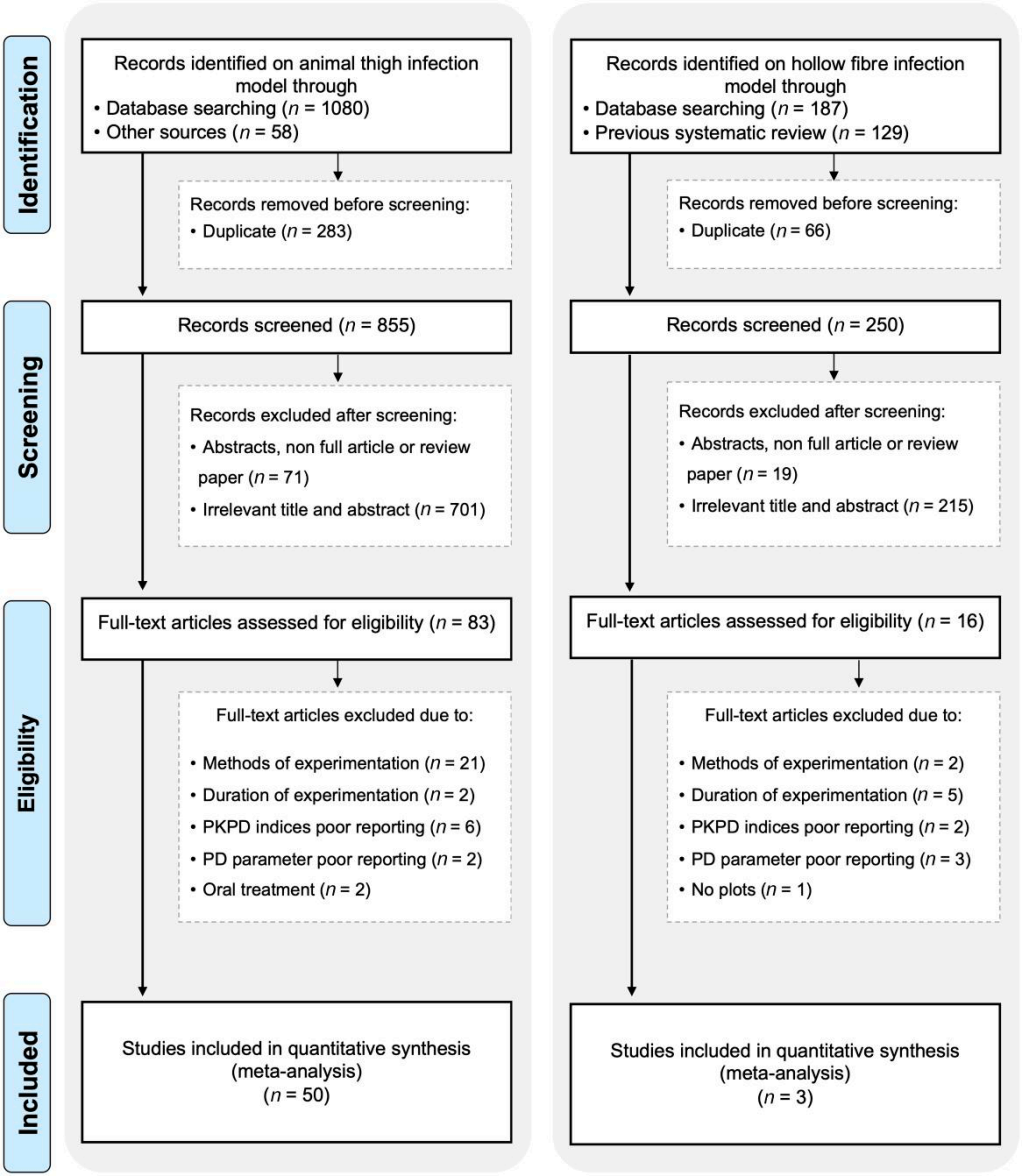
The PRISMA flow chart illustrates the identification, screening, eligibility and inclusion of literature.

Out of 1138 identified records, 50 were MTIM studies, whereas three out of 316 studies were a HFIM, as depicted in Figure 1 of the PRISMA chart. The summarized results in Table 2 offer an overview of the characteristics and details of the included MTIM and HFIM studies. The compiled dataset is available from the UCL Research Data Repository.^76^

**Table 2.** Summary of pharmacokinetic/pharmacodynamic indices across studies using either murine thigh or HFIMs

Study (year)	Journal	Model	Drug (drug class)	Number of regimens/organism	Time in hours to initiate therapy	Tested organisms	Mice weight g (sex)	Inoculum size (cfu)	Author’s identified PKPD index	Remodelled PKPD index
Lepak *et al.* (2017)^24^	Antimicrob Agents Chemother	MTIM	Fosfomycin (Phosphonic acid derivative)	20	2	*E. coli* 1-741-1	24–27 (F)	$2\;\times 10^{6}$	AUC/MIC	AUC/MIC
XIAO and XIAO (2008)^25^	Acta Pharmacol Sin	MTIM^a^	Antofloxacin (Fluoroquinolone)	32	2	*S. aureus* ATCC 29213	19–21 (F)	$1\;\times 10^{5}$	AUC/MIC	AUC/MIC
						Clinical 03229			AUC/MIC	AUC/MIC
Ji *et al*. (2020)^26^	Antimicrob Agents Chemother	MTIM^a^	Benapenem (Carbapenem)	21	2	ATCC 25922	NA (F and M)	$1\;\times 10^{4}$	*T* > MIC	AUC/MIC
						13G136			AUC/MIC	AUC/MIC
						7742692			AUC/MIC	T > MIC
						ATCC 700603			*C*_max_/MIC	AUC/MIC
						13C285			AUC/MIC	AUC/MIC
						13H279			*T* > MIC	*T* > MIC
Takata *et al*. (2004)^27^	J Infect Chemother	MTIM	Biapenem (Carbapenem)	24	2	*P. aeruginosa* TH-4950	18–22 (M)	$1\;\times 10^{6}$	*T* > MIC	*T* > MIC
			Imipenem/Cilastatin (Carbapenem)	24					*T* > MIC	*T* > MIC
			Meropenem/Cilastatin (Carbapenem)	24					*T* > MIC	*T* > MIC
			Ceftazidime (Cephalosporin)	18					*T* > MIC	*T* > MIC
Nakamura *et al*. (2019)^28^	Antimicrob Agents Chemother	MTIM	Cefepime (Cephalosporin)	16	2	*P. aeruginosa* SR27016	17–20 (M)	$1\;\times 10^{5}$	*T* > MIC	*T* > MIC
			Cefiderocol (Cephalosporin)	16	2				*T* > MIC	*T* > MIC
Takemura *et al*. (2021)^29^	Pharm Res	MTIM^a^	Cefmetazole (Cephalosporin)	28	2	*ESBL-EC* (EC19 and EC9)	NA (F)	$3.75\;\times 10^{5}$	*T* > MIC	*T* > MIC
Guo *et al*. (2016)^30^	Antimicrob Agents Chemother	MTIM	Cefquinome (Cephalosporin)	24	2	*S. suis* ATCC 43765	22–27 (F)	$1\;\times 10^{5}$	*T* > MIC	*T* > MIC
Shan *et al*. (2014)^31^	Antimicrob Agents Chemother	MTIM	Cefquinome (Cephalosporin)	20	2	*E. coli* ATCC 25922	24–27 (F)	$1\;\times 10^{5}$	*T* > MIC	*T* > MIC
Shan and Wang, 2017^32^	J Vet Pharmacol Ther	MTIM	Cefquinome (Cephalosporin)	20	2	*K. pneumoniae*	22–25 (F)	$1\;\times 10^{5}$	*T *> MIC	*T* > MIC
Wang *et al*. (2014)^33^	Antimicrob Agents Chemother	MTIM	Cefquinome (Cephalosporin)	25	2	*S. aureus* ATCC 29213	24–27 (F)	$1\;\times 10^{5}$	T > MIC	*T* > MIC
Craig and Andes (2008)^34^	Antimicrob Agents Chemother	MTIM	Ceftobiprole (Cephalosporin)	18	2–10	*S. aureus* ATCC 33591	23–27 (F)	$1\;\times 10^{6}$	*T* > MIC	*T* > MIC
Craig and Andes (2013)^35^	Antimicrob Agents Chemother	MTIM	Ceftolozane (Cephalosporin)	8	2	*K. pneumoniae* ATCC 43816	23–27 (F)	$1\;\times 10^{5}$	*T* > MIC	*T* > MIC
Dudhani *et al*. (2010)^36^	Antimicrob Agents Chemother	MTIM	Colistin (Polymyxin)	10	2	*P. aeruginosa* ATCC 27853	22–26 (F)	$5\;\times 10^{5}$	AUC/MIC	AUC/MIC
Dudhani *et al*. (2010)^37^	J Antimicrob Chemother	MTIM	Colistin (Polymyxin)	8	2	*A. baumannii* ATCC19606	22–26 (F)	$5\;\times 10^{5}$	AUC/MIC	*C*_max_/MIC
Zhao *et al*. (2017)^38^	Antimicrob Agents Chemother	MTIM	Eravacycline (Tetracycline)	8	2	*E. coli* ATCC 25922	23–27 (F)	$1\;\times 10^{6}$	AUC/MIC	AUC/MIC
Tashiro *et al*. (2021)^39^	Pharm Res	MTIM^a^	Flomoxef (Cephalosporin)	4	2	*E. coli* 12, *E. coli* 9	NA (F)	$3.75\;\times 10^{5}$	*T *> MIC	*T* > MIC
Roelofsen *et al*. (2022)^40^	Antibiotics	MTIM^a^	Flucloxacillin (penicillin)	12	2	*S. aureus* MUP1621	20–30 (F)	$5\;\times 10^{6}$	*T* > MIC	*T* > MIC
						*S. aureus* MUP4421			*T* > MIC	*T* > MIC
Chavan *et al*. (2023)^41^	Microb Drug Resist	MTIM^a^	Fosfomycin (Phosphonic acid derivative)	18	2	*E. coli* ATCC 25922	25–30 (M)	$2.50\;\times 10^{5}$	AUC/MIC	AUC/MIC
						*K. pneumoniae* NCTC 13368			AUC/MIC	AUC/MIC
Andes and Craig (2003)^42^	Antimicrob Agents Chemother	MTIM^a^	Garenoxacin (Fluoroquinolone)	8	2	*S. aureus* ATCC 33591	23–27 (F)	$3.16\;\times 10^{5}$	AUC/MIC	AUC/MIC
						*S. pneumoniae* ATCC 10813			AUC/MIC	AUC/MIC
Andes and Craig (2002)^43^	Antimicrob Agents Chemother	MTIM	Gatifloxacin (Fluoroquinolone)	8	2	*S. pneumoniae* ATCC 10813	23–27 (F)	$1\;\times 10^{5}$	AUC/MIC	AUC/MIC
Bulik *et al*. (2017)^44^	Antimicrob Agents Chemother	MTIM^a^	Gepotidacin (Triazaacenaphthylene bacterial topoisomerase)	20	2	*S. aureus* ATCC 33591	19–21 (F)	$1\;\times 10^{6}$	AUC/MIC *T* > MIC	AUC/MIC
						*S. pneumoniae* ATCC 10813			*T* > MIC	AUC/MIC
Ferrari *et al*. (2003)^45^	Antimicrob Agents Chemother	MTIM	GV143253A (trinem)	6	2	*MSSA* ATCC 25923	NA (F)	$8.80\;\times 10^{6}$	*T* > MIC	*T* > MIC
Wicha *et al*. (2019)^46^	J Antimicrob Chemother	MTIM^a^	Lefamulin (Pleuromutilin)	8	2	*S. aureus* ATCC 25923	23–27 (F)	$1\;\times 10^{5}$	AUC/MIC	*T > *MIC
						*S. pneumoniae* ATCC 10813			AUC/MIC	*T* > MIC
Growcott *et al*. (2019)^47^	J Antimicrob Chemother	MTIM	LYS228 (Monobactam)	NA	2	*E. coli* ATCC 25922	17–20 (F)	$5\;\times 10^{4}$	*T* > MIC	*T* > MIC
Kristoffersson *et al*. (2016)^48^	Pharm Res	MTIM	Meropenem (Carbapenem)	12	NA	*P. aeruginosa*	24.4 (F) and 29.6 (M)	$3.16\;\times 10^{6}$	*T* > MIC	*T* > MIC
Fratoni *et al*. (2022)^49^	J Antimicrob Chemother	MTIM	Minocycline (Tetracycline)	9	2	*S. maltophilia* STM C42-70	20–22 (F)	$2\;\times 10^{6}$	AUC/MIC	AUC/MIC
Melchers *et al*. (2019)^50^	Antimicrob Agents Chemother	MTIM	Murepavadin (Peptidomimetic)	8	2	*P. aeruginosa* ATCC 27853	22–25 (NA)	$5\;\times 10^{5}$	AUC/MIC	AUC/MIC
Zhao *et al*. (2018)^51^	Antimicrob Agents Chemother	MTIM	NOSO-502 (Odilorhabdin)	8	2	*E. coli* ATCC 25922	23–27 (F)	$1.26\;\times 10^{6}$	AUC/MIC	AUC/MIC
Andes *et al*. (2009)^52^	Antimicrob Agents Chemother	MTIM	NZ2114 (Plectasin)	8	2	*S. pneumoniae* ATCC 10813	22–27 (F)	$2.51\;\times 10^{5}$	*C*_max_/MIC,	*C*_max_ /MIC,
						*S.aureus* ATCC 25923			AUC/MIC^b^	AUC/MIC^b^
Umezaki *et al*. (2022)^53^	Antibiotics	MTIM	Pazufloxacin (Quinolone)	8	2	*P. aeruginosa* ATCC 27853	NA (F)	$6\;\times 10^{3}$	AUC/MIC	AUC/MIC
Lepak *et al*. (2020)^54^	Antimicrob Agents Chemother	MTIM	Polymyxin MRX-8 (Polymyxin)	20	2	*E. coli* ATCC 25922	24–27 (F)	$2.51\;\times 10^{6}$	*C*_max_ /MIC	*C*_max_ /MIC
Andes and Craig (2006)^55^	Antimicrob Agents Chemother	MTIM	PPI-0903 (TAK-599) (Cephalosporin)	8	2	*K. pneumoniae* ATCC 43816	23–27 (F)	$1\;\times 10^{6}$	*T* > MIC	*T* > MIC
						*S. aureus* ATCC 33591			*T *> MIC	*T* > MIC
						*S. pneumoniae* ATCC 10813			*T* > MIC	*T* > MIC
Hirai *et al*. (2016)^16^	J Infect Chemother	MTIM	Rifampicin (Macrolide)	12	3	*S. aureus* ATCC 25923	22 (F)	$1\;\times 10^{7}$	*C*_max_/MIC	*C*_max_/MIC
Griffith *et al*. (2008)^56^	Antimicrob Agents Chemother	MTIM^a^	RWJ-54428 (Cephalosporin)	8	2	*E. faecalis* EFS 007	NA (M)	$5\;\times 10^{5}$	*T* > MIC	*T* > MIC
						*S. aureus* COL (MRSA)			*T* > MIC	*T* > MIC
						*S. pneumoniae* SP 019			*T* > MIC	*T* > MIC
Yokoyama *et al*. (2014)^57^	Int J Antimicrob Agents	MTIM	Sulbactam (β-lactamase inhibitor)	18	2	*A. baumannii* ATCC 19606	NA (F)	$3.75\;\times 10^{5}$	*T* > MIC	*T* > MIC
Hegde *et al*. (2012)^58^	Antimicrob Agents Chemother	MTIM	TD-1792 (Cephalosporin)	12	1	*MRSA* ATCC 33591	18–30 (F)	$5\;\times 10^{4}$	AUC/MIC	AUC/MIC
Liu *et al*. (2023)^59^	Pharm Res	MTIM^a^	Tedizolid (Oxazolidinone)	31	2	*MRSA* ATCC 33591, *MRSA* ATCC 43300	22–24 (F)	$5\;\times 10^{5}$	AUC/MIC	AUC/MIC
						*VRE* ATCC 700221			AUC/MIC	AUC/MIC
Watanabe *et al*. (2021)^60^	J Glob Antimicrob Resist	MTIM	Teicoplanin (Glycopeptide)	36	2	*S. aureus* ATCC 29213	NA (M)	$3.75\;\times 10^{5}$	*C*_max_/MIC	*C*_max_/MIC
						*S. aureus* ATCC 43300			AUC/MIC^b^	*C*_max_/MIC
Hegde *et al*. (2004)^61^	Antimicrob Agents Chemother	MTIM	Telavancin (TD-6424) (Lipoglycopeptide)	20	1	*MRSA* 33591	20–25 (F)	$5\;\times 10^{4}$	AUC/MIC	AUC/MIC
Sugihara *et al*. (2010)^62^	Antimicrob Agents Chemother	MTIM	Tomopenem (+cilastatin) (1-beta-methylcarbapenem)	20	2	*MRSA* 12372	NA (M)	$1\;\times 10^{6}$	*T* > MIC	*T* > MIC
						*P. aeruginosa* 12467			*T* > MIC	*T > *MIC
			Meropenem (Carbapenem)	20	2	*P. aeruginosa* 12467			*T* > MIC	*T* > MIC
Louie *et al*. (2011)^63^	Antimicrob Agents Chemother	MTIM	Torezolid Phosphate (TR-701) (Oxazolidinone)	12	2	*MRSA* ATCC 33591	22–25 (F)	$1\;\times 10^{5}$	AUC/MIC	AUC/MIC
Hagihara *et al*. (2020)^64^	Chemotherapy	MTIM	Trimethoprim/Sulfamethoxazole (Sulfonamides)	9	2	*S. aureus* (3 MSSA and 2 MRSA)	22 (F)	$1\;\times 10^{7}$	*T* > MIC	*T* > MIC
Lepak *et al*. (2015)^65^	Antimicrob Agents Chemother	MTIM	TXA-709 (Methoxybenzamide FtsZ inhibitor)	16	2	*S. aureus* ATCC 25923	23–27 (F)	$3.16\;\times 10^{5}$	AUC/MIC	AUC/MIC
Andes and Craig (2006)^66^	Antimicrob Agents Chemother	MTIM	XRP 2868 (Streptograrnin)	20	2	*S. aureus* ATCC 29213	23–27 (F)	$1\;\times 10^{6}$	AUC/MIC	*C*_max_/MIC
						*S. pneumoniae* ATCC 10813			AUC/MIC	*T* > MIC^b^
Vogelman *et al*. (1988)^67^	J Infect Dis	MTIM	Tobramycin (Aminoglycoside)	30	2	*P. aeruginosa* ATCC 27853	24–26 (F)	$1\;\times 10^{5}$	AUC/MIC	AUC/MIC
			Ticarcillin (Pencillin)	56	2				*T* > MIC	*T* > MIC
He *et al*. (2023)^68^	Infect. Drug Resist	MTIM	LYSC98 (Miscellaneous)	12	2	*S. aureus* ATCC29213	16–22 (M)	$2\;\times 10^{6}$	*C*_max_/MIC	*C*_max_/MIC
Van den Berg *et al*. (2025)^69^	J Antimicrob Chemother	MTIM	NOSO-502 (Miscellaneous)	8	2	*K. pneumoniae* ATCC 43816	23–25 (F)	$4\;\times 10^{5}$	AUC/MIC × 1/tau	AUC/MIC × 1/tau
Eguchi *et al*. (2009)^70^	Antimicrob Agents Chemother	MTIM	SMP-601 (PTZ601) (Carbapenem)	8	2	*VREF* TL-3273	18–22 (M)	$1\;\times 10^{5}$	AUC/MIC	AUC/MIC
						*VREF* TL-3621			AUC/MIC	AUC/MIC
						*MRSA* SP-12249		$1\;\times 10^{6}$	*T* > MIC	*T* > MIC
						*MRSA* TL-3677			*T* > MIC	*T* > MIC
Andes *et al*. (2002)^71^	Antimicrob Agents Chemother	MTIM	Linezolid (Oxazolidinone)	16	2	*S. pneumoniae* ATCC 10813	NA (M)	$1\;\times 10^{6}$	AUC/MIC	AUC/MIC
						*S. aureus* ATCC 6538p			AUC/MIC	AUC/MIC
Van Wart *et al*. (2009)^72^	Diagn Microbiol Infect Dis	MTIM	Doripenem (Carbapenem)	8	2	*K. pneumoniae* ATCC 43816	23–27 (F)	$1\;\times 10^{6}$	*T* > MIC	*T* > MIC
Basarab *et al*. (2015)^73^	Scientific Reports	HFIM	ETX0914 (Miscellaneous)	NA	NA	*S. aureus* ARC516	—	$1\;\times 10^{6}$	AUC/MIC	AUC/MIC
O'Donnell *et al*. (2024)^74^	Antimicrob. Agents Chemother	HFIM	Sulbactam (β-lactamase inhibitor)	NA	1	*A. baumannii* ARC2058	—	$1.5\;\times 10^{7}$	*T* > MIC	*T* > MIC
			Durlobactam (β-lactamase inhibitor)		1	*A. baumannii* ARC5081			AUC/MIC	AUC/MIC
Singh *et al*. (2015)^75^	Antimicrob. Agents Chemother	HFIM	Aztreonam/Avibactam (Monobactam/β-lactamase inhibitor)	7	NA	*K. pneumoniae* ARC3802	—	$1.5\;\times 10^{7}$	*T* > MIC	*T* > MIC

NA, not available; F, Female; M, Male.

^a^Indicates PKPD index derived from multiple organisms with similar MICs.

^b^Denotes cases where multiple PKPD indices arise due to the use of different organisms.

#### Murine thigh infection model studies

Murine subjects, age range from 4 to 8 weeks, and weight ranges from 17 to 30 g, were rendered neutropenic. In most of the cases, mice were aged 6 weeks (46%) and weighing between 23 and 27 g (24%). The ICR/Swiss strain was the most frequently used (44%), followed by CD-1 mice (18%) and ddY mice (10%). Other strains, like JcL:ICR, Swiss mice, NSA mice and Swiss albino mice, were less common. Female mice were used in 78% of the papers. Some papers, however, failed to report the age 24%, weight 22% and gender 2% of the mice, yet these papers were still included.

Treatment initiation occurred 2 hours after inoculation in 88%. Inoculum sizes ranged widely from 1.00 × 10^4^ to 1.00 × 10^7^ cfu, averaging 1.13 × 10^6^ cfu. Subcutaneous drug administration was the primary method used in most of the experiments, while intramuscular, intravenous and intraperitoneal routes were less common, with each of these routes being used in three separate publications.

#### Quality assessment of MTIM studies

The MTIM studies were systematically assessed using the CAMARADES Quality Checklist (Table S3), comprising 10 critical elements including peer-reviewed publication, temperature control, randomization, allocation concealment, blinded outcome assessment, avoidance of certain anaesthetics, animal health considerations, sample size calculation, regulatory compliance statement and conflict of interest disclosure. Each study was evaluated against these criteria using a binary scoring system (1 for present, 0 for absent). The ‘Total score’ column aggregated individual scores, ranging from 1 to 6, indicating the overall study quality (Table S4). The highest score obtained was 6, the lowest was 1, with an average total score of 2. This assessment provided insight into the methodological strengths and limitations of the MTIM studies, aiding the evaluation of their robustness and reliability.

#### Hollow fibre infection model study

Three studies were identified that used the HFIM. The inoculum sizes used in these studies were $1\times 10^{6}$ cfu,^73^ and $1.5\times 10^{7}$cfu.^74,75^ It was not specified in any study whether treatment began immediately after inoculation or after a specified period. Furthermore, none provided information regarding the media used in the HFIM system.

### Study outcomes

#### Optimal PKPD index

**Figure 2.**
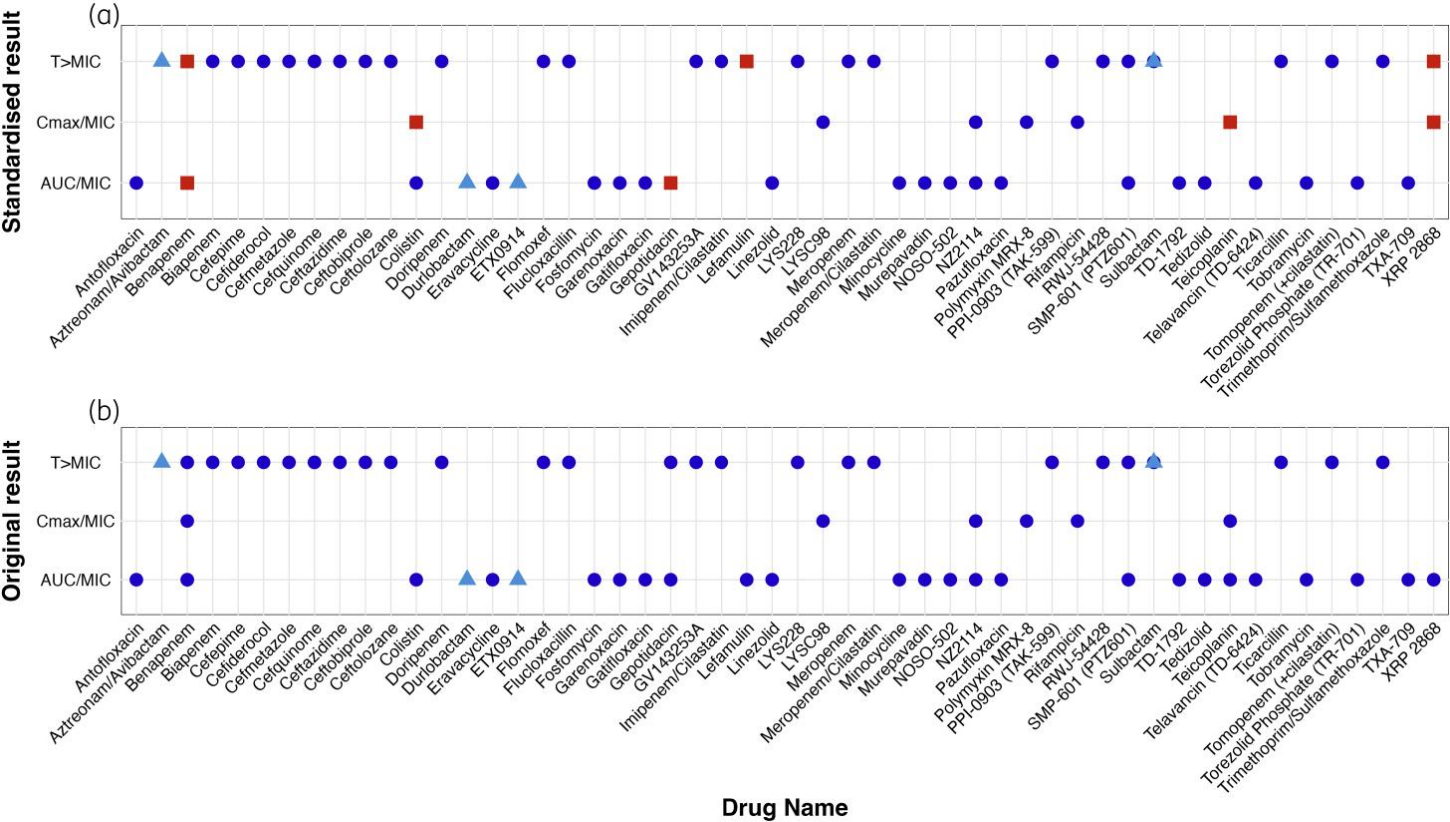
Combined scatter plots illustrating the PKPD indices of identified antibiotics. (a) The original PKPD indices results. (b) The remodelled PKPD indices using a standardized model, with squares indicating instances where results differ from those in (a). HFIM is represented by triangles and MTIM by circles in both plots. The results with asterisks* were from two papers: one used fAUC/MIC·1/τ^69^ and the other used fAUC/MIC.^51^

Full PKPD index outputs for each study are provided in Figure S1. For comparative analysis, the concordance between the PKPD indices from the original studies and those from the remodelled data obtained in this study was assessed. This was facilitated through the construction of two distinct box plots (Figure 2). An analysis of remodelling outcomes revealed disparities in six papers compared with the original findings.^26,36,44,46,60,64,66^

Benapenem was tested against five organisms in the study by Ji *et al*., with discrepancies being observed in three of the organisms. The reported indices for *E. coli* (ATCC 25922), *K. pneumoniae* (7742692), and *E. cloacae* (ATCC 700603) were *T* > MIC, AUC/MIC and *C*_max_/MIC, respectively, while remodelling identified AUC/MIC, *T* > MIC and AUC/MIC, respectively. Dudhani *et al*. reported an AUC/MIC for colistin against *A. baumannii*, but upon remodelling a *C*_max_/MIC was found. Gepotidacin was tested against *S. aureus* and *S. pneumoniae* by Bulik *et al*., and the original indices reported were AUC/MIC and *T* > MIC, respectively, while the modelling indices were consistently identified as AUC/MIC. Wicha *et al*. reported AUC/MIC for lefamulin, whereas *T* > MIC was identified through remodelling. In Watanabe *et al*., teicoplanin was reported as AUC/MIC, but remodelled result showed *C*_max_/MIC. Last, Andes and Craig reported AUC/MIC for XRP 2868 against *S. aureus* (ATCC 29213) and *S. pneumoniae* (ATCC 10813), whereas remodelling identifies Cmax/MIC and *T* > MIC respectively.

### Target attainment

Some discrepancies were found between the original data and the remodelled results for the targets of stasis, 1-log kill and 2-log kill. The experimental design of one study resulted in the failure to achieve the stasis target.^64^ A mismatch between the original and remodelled results, defined as being >2-fold higher or lower, was observed in 17% of stasis targets,^16,26,44,46,50,55,60,65,66^ 28% of 1-log kill targets^16,25,26,29,41,44,46,49,50,55,60,65,66^ and 15% of 2-log kill targets.^31,37,41,43,55,60,66,70^

### Experimental design and modelling practice

#### Diversity of microorganisms

Out of 53 papers, 65%^16,24,27,28,30–38,43,45,47–51,53,54,57,58,61,63,65,67–69,72–75^ focused solely on one organism, whereas 23%^25,29,39–42,44,46,52,60,62,66^ investigated two organisms, and 12% examined the optimal PKPD index on more than two organisms.^26,55,56,59,64,70^

#### Dose ranges

The number of distinct dosing regimens tested per study varied widely, ranging from 6 to 56. One study, for example, tested only six unique dosing regimens, representing the lower end of the range.^40^ However, it is crucial to provide justification when using a limited number of dosing schedules.

#### Impact of antibiotic protein binding

The impact protein binding may have on PKPD indices was evaluated. The focus was on MTIM studies, as HFIM generally uses Mueller–Hinton broth, which has negligible protein binding capacity.^77^ Among 50 MTIM studies, six required recalibrations due to substantial protein binding (17.9% to 66%).^16,27,43,45,55,58^ Authors reported total drug concentrations, prompting calculation of free drug concentrations. Following the recalibration of the free drug concentrations and a comparison with the total drug concentrations, the optimal PKPD indices remained consistent.

#### Model selection criteria

In all studies analysed, only Fratoni *et al*. used AIC as a guiding statistic for selecting the optimal PKPD index. The remaining papers, including both HFIM and MTIM studies, relied on *R*² values for this purpose.

#### Response metric

Out of 53 papers in the dataset, 14 used log cfu as a response metric without showing the log change in cfu. To address this inconsistency, a recalibration process was implemented for the remodelling. This process involved adjusting the *y*-axis to represent log change in cfu rather than log cfu alone. The adjustment was made where the log change in cfu equals the difference between log cfu at the end of a treatment and the initial inoculum size at time 0 for growth control experiments.

## Discussion

Drug approval by regulatory agencies requires a clear understanding of PK and PD for target attainment of antibiotics.^7,78–81^ However, many antibiotics were developed befire a full understanding of PKPD concepts. The existing literature lacks standardized approaches for conducting, analysing and reporting PKPD index dose fractionation studies, presenting a knowledge gap. In the current systematic review, the following significant findings emerged:

### Variation in PKPD index results

Upon data remodelling, discrepancies in the PKPD index between the original papers and our analyses were identified. Methodological and modelling differences, along with complexities in drug response, may contribute to these variations.

The study by Andes and Craig exemplifies a scenario where the author identified AUC/MIC as the optimal index for the drug XRP 2868 on *S. aureus*, using *R*² values for selection, whereas both AUC/MIC (*R*² = 91%) and *C*_max_/MIC (*R*² = 90%) exhibited similar values. Remodelling guided by AIC rather than *R*² identified *C*_max_/MIC as the optimal index with an AIC of 42.3 compared with 49.7. This highlights the way AIC can give a clearer distinction of optimal model fit, but also the pitfalls of using a numerical metric of model fit alone. The use of *C*_max_/MIC in the MTIM possibly is limited by the dependence of *C*_max_ on the timing of peak concentration measurements, and the best PKPD index for this drug in other organisms was AUC/MIC, possibly explaining why the best-fitting index was not chosen.

In other example, Wicha *et al*. (lefamulin) and Dudhani *et al*. (colistin) used cfu rather than the change in cfu to model results. This choice in experimental design may have contributed to differences found between reported and the remodelled outcomes since we transformed the data to change in cfu to standardize outputs. Although it could be argued that change in cfu potentially introduces bias through conditioning the results on the growth control results, it is the most commonly used method and readily allows for metrics such as bacteriostasis, 1-log and 2-log kill to be calculated.

Whether or not parameters such as E0 or Emax were fixed was rarely reported and most reports did not describe Emax model building processes. In Sanne *et al*., using a single Emax model identified %fT > MIC as the best standard index, while fAUC/MIC·1/*τ* showed the strongest overall correlation. Re-analysis with eight Emax model variations per index found fAUC/MIC to be more predictive than %fT > MIC or f*C*_max_/MIC, although fAUC/MIC·1/τ remained the best overall. These findings are consistent with those of Zhao *et al.*, who also identified fAUC/MIC as the optimal index for the same drug (NOSO-502), support the value of systematically comparing multiple Emax model forms using AIC, a step that was not undertaken in their analysis.

The differences in inoculum sizes across the included studies, as outlined in Table 2, may have impacted the PKPD index analysis. This is particularly relevant for antibiotics that exhibit an inoculum effect,^82,83^ where higher initial bacterial loads can reduce drug activity due to factors such as increased enzyme production or changes in growth kinetics. In such cases, a lower inoculum may lead to an overestimation of the drug’s effect, while a higher inoculum may result in underestimation. Therefore, standardizing the initial inoculum size can be considered best practice in PKPD studies.

### Predominance of single-organism experiments

The current study also revealed a predominant focus on single-organism experiments, with only 35% of studies incorporating multiple organisms. Within this subset, some studies used organisms with similar MICs,^25,41,42,46,66^ potentially limiting the generalizability of findings. Conversely, studies deliberately selected organisms with varying MICs,^26,40,44,52,55,56,60^ offering a more comprehensive assessment of antimicrobial efficacy.

The pharmacodynamic response observed in PKPD studies can be influenced by the range of organisms used. The lack of diversity of organisms may hinder the generalizability of models. As a result, the model may not adequately reflect variation in drug response across species and strains.

To improve model applicability, the European Medicines Agency (EMA) recommends including a basic set of 4–5 organisms representing major groups in pre-clinical PKPD studies.^7^ These organisms should reflect clinical relevance and include strains with and without specific resistance mechanisms, including those with high MICs. This comprehensive approach aims to enhance the relevance, diversity, and reliability of pre-clinical PKPD studies.

While including multiple species in PKPD studies may increase animal use, ethical and financial implications must be considered. The development of alternative methods, such as the *in vitro* HFIM, aims to reduce animal usage while maintaining the quality and predictive power of the results while following the principles of the NC3Rs.^84^

### *Prevalence of* R*² values as model selection metric*

In both MTIM and HFIM studies, *R*² values were frequently used as the primary metric for model selection, raising concerns about potential overfitting. Disparities between author-reported and remodelled PKPD indices were observed only in studies that relied on *R*² values; the single study that used AIC showed no such discrepancies, highlighting the influence of chosen criteria on index determination. This consistent pattern underscores the need for standardized criteria, possibly favouring AIC over *R*², to enhance consistency and comparability across studies.

In PKPD index modelling, metrics such as AIC and BIC offer a balance between goodness of fit and model complexity, particularly valuable for nonlinear models such as Emax.^9,10,13^ Although *R*² is simple, and easy to interpret, its effectiveness diminishes when applied to complex and nonlinear PKPD models.^9,10^ The observed preference for *R*² over AIC may stem from its simplicity and alignment with traditional linear regression techniques.

### The use of total drug concentration in modelling

Most papers relied on free drug concentration to calculate the PK/PD index before fitting the model, while only a subset (6 out of 53) used total drug concentration to identify PKPD indices. This raises concerns about variations in drug exposure assessments and their implications for modelling accuracy.

In drug pharmacodynamics, the choice between total and free drug concentrations is crucial for accurately representing drug activity. Free drug fractions exert pharmacological effects, while bound portions attached to plasma proteins like albumin remain inactive. Many PKPD models, including the widely used Emax model, assume that the PD effect depends on free drug concentration. Therefore, PKPD indices, which quantify the relationship between drug exposure and effect, are more accurately expressed in terms of free drug concentrations.

Despite some studies assuming similar protein binding levels between animal models and humans, inter-species variations and physiological differences can significantly affect drug–protein interactions. Regulatory agencies, such as the EMA, recommend expressing PKPD indices as a function of free drug concentrations, emphasizing the importance of considering the biologically active fraction for consistency and accuracy in the assessment of drug efficacy.

### Overall implications and recommendations

To advance methodological practices and ensure the reliability of study outcomes, the current systematic review suggests several implications for PKPD index finding:

- Organism diversity: limited diversity of tested organisms may compromise PKPD index generalizability, emphasizing the importance of including a range of organisms for more robust studies.
- Dose range optimization: researchers should aim for a diverse set of dosing regimens, ideally five or more with two dosing regimens expected to achieve concentrations below MIC and two above MIC, to better capture the dose–response curve for reliable Emax model fitting.
- Protein binding influence: for an accurate representation of drug activity, it is essential to consider free drug concentrations when performing PKPD analysis.
- Response metric: careful consideration of response metrics, such as the change in cfu over time, is recommended to improve PKPD analyses accuracy and interpretability.
- Model selection: adopting standardized criteria for Emax model selection, possibly favouring AIC over *R*², can support more reliable identification of the best-performing index within a given dataset. When selecting metrics, it is important to carefully weigh the simplicity of *R*² against the robustness of AIC, and also consider how the index will be interpreted and whether adopting a single index across different organisms for a drug might be more practical than having different indices for different strains.

### Limitation

The limited number of HFIM studies, combined with the use of different antibiotics compared with those in the MTIM studies, prevented a meaningful comparison between the two models. If HFIM is to replace MTIM further dose fractionation studies in HFIM are required. In addition, details on how the PK was determined in the included studies, such as whether infected or healthy animals were used, were not extracted, which could influence the overall PKPD index result. This is largely due to inconsistent or insufficient reporting across the included studies.

## Conclusion

In conclusion, selecting optimal PKPD indices is crucial for the design of an effective antibiotic dosing regimen. The current review highlights the importance of establishing standard approaches and methodological consistency in PKPD index. The discrepancy between reported and remodelled PKPD indices provides valuable insights into methodologies and highlight areas for further refinement to enhance the reliability of these indices. The applicability of the Emax model can be constrained in cases with limited dose ranges and organism diversity, which may reduce the reliability of PKPD index estimation under such conditions.

## Supplementary Material

dkaf446_Supplementary_Data

## Supplementary data

Figure S1 and Tables S1 to S4 are available as Supplementary data at *JAC* Online.
