# Supplementary material for: Systematic review of antimicrobial pharmacokinetic/pharmacodynamic indices in murine thigh and hollow fibre dose fractionation studies analysed with a standard method
Source: J Antimicrob Chemother. 2025 Dec 17;81(1):dkaf446. doi: 10.1093/jac/dkaf446 (PMC12802965; doi:10.1093/jac/dkaf446)
Supplement: dkaf446_Supplementary_Data [file dkaf446_supplementary_data.pdf]

Supplementary

Table S1 Search builder for systematic review databases on the murine thigh infection model

Database PubMed

Number	Search terms	Results
**1**	Search **((((((PKPD index) OR (PKPD indices)) OR (pharmacokinetic/pharmacodynamic index)) OR (pharmacokinetic/pharmacodynamic indices)) OR (dose fraction)) OR (dose fraction study)) OR ((dose) AND (fraction)) OR (pharmacokinetic/pharmacodynamic)**	88,460
**2**	Search **((((((((murine model) AND (thigh infection)) OR (murine thigh infection model)) OR (neutropenic thigh infection model)) OR (neutropenic murine thigh infection model)) OR (murine infection model)) OR (neutropenic mouse)) OR (neutropenic mouse model)) OR (neutropenic mice)**	130,332
**3**	Search **((((((antibiotic agent) OR (antibiotic)) OR (antiinfective agent)) OR (antimicrobial)) OR (antibacterial)) OR (antibacterial agent) OR (("Anti-Bacterial Agents/pharmacokinetics"[MAJR]) OR "Anti-Bacterial Agents/pharmacology"[MeSH]) OR "Anti-Bacterial Agents/therapeutic use"[MeSH])**	2,344,057
**4**	Search **((#1) AND (#2)) AND (#3) AND [Full text]**	455

Database SCOPUS		
Number	Search terms	Results
**1**	Search **(TITLE-ABS-KEY ("murine model of thigh infection") OR TITLE-ABS-KEY ("murine thigh infection model") OR TITLE-ABS-KEY ("neutropenic thigh infection model") OR TITLE-ABS-KEY ("neutropenic murine thigh infection model") OR TITLE-ABS-KEY ("murine infection model") OR TITLE-ABS-KEY ("neutropenic mouse") OR TITLE-ABS-KEY ("neutropenic mouse model") OR TITLE-ABS-KEY ("neutropenic mice"))**	1,495
**2**	Search **(TITLE-ABS-KEY ("antibiotic agent") OR TITLE-ABS-KEY ("antibiotic") OR TITLE-ABS-KEY ("antiinfective agent") OR TITLE-ABS-KEY ("antimicrobial") OR TITLE-ABS-KEY ("antibacterial") OR TITLE-ABS-KEY ("antibacterial agent"))**	1,622,254
**3**	Search **(TITLE-ABS-KEY ("pk pd index") OR TITLE-ABS-KEY ("pk pd indices") OR TITLE-ABS-KEY ("pharmacokinetic pharmacodynamic index") OR TITLE-ABS-KEY ("pharmacokinetic pharmacodynamic indices") OR TITLE-ABS-KEY ("dose fraction") OR TITLE-ABS-KEY ("dose fraction study") OR TITLE-ABS-KEY ("dose and fraction"))**	1,586
**4**	Search **("#1") AND ("#2") AND ("#3") AND (LIMIT-TO (LANGUAGE , "English"))**	40

Database BIOSIS		
Number	Search terms	Results
**1**	Search **antibiotic agent (Topic) OR antibiotic (Topic) OR antiinfective agent (Topic) OR antimicrobial (Topic) OR antibacterial (Topic) OR antibacterial agent (Topic)**	1,110,378
**2**	Search **murine model of thigh infection (Topic) OR murine thigh infection model (Topic) OR neutropenic thigh infection model (Topic) OR neutropenic murine thigh infection model (Topic) OR murine infection model (Topic) OR neutropenic mouse (Topic) OR neutropenic mouse model (Topic) OR neutropenic mice (Topic)**	27,531
**3**	Search **PK PD index (Topic) OR PK PD indices (Topic) OR pharmacokinetic pharmacodynamic index (Topic) OR pharmacokinetic pharmacodynamic indices (Topic) OR PKPD studies (Topic) OR pharmacokinetic/pharmacodynamic studies (Topic) OR PKPD analysis (Topic) OR pharmacokinetic/pharmacodynamic analysis (Topic) OR model based analysis (Topic) OR dose fraction (Topic) OR dose fraction study (Topic) OR dose and fraction (Topic)**	465,684
**4**	Search **#1 AND #2 AND #3 and English (Languages)**	477

**Database** EMBASE		
Number	Search terms	Results
**1**	Search **(antibiotic agent or antibiotic or antiinfective agent or antimicrobial or antibacterial or antibacterial agent).af.**	134,6550
**2**	Search **(murine model of thigh infection or murine thigh infection model or neutropenic thigh infection model or neutropenic murine thigh infection model or murine infection model or neutropenic mouse or neutropenic mouse model or neutropenic mice).af.**	1,495
**3**	Search **(PK PD index or PK PD indices or pharmacokinetic pharmacodynamic index or pharmacokinetic pharmacodynamic indices or PK PD studies or pharmacokinetic pharmacodynamic studies or PK PD analysis or pharmacokinetic pharmacodynamic analysis or model based analysis or dose fraction or dose fraction study or (dose and fraction)).af.**	93,626
**4**	Search **1 and 2 and 3**	59

**Database** MEDLINE		
Number	Search terms	Results
**1**	Search **(antibiotic agent or antibiotic or antiinfective agent or antimicrobial or antibacterial or antibacterial agent).af.**	648,534
**2**	Search **(murine model of thigh infection or murine thigh infection model or neutropenic thigh infection model or neutropenic murine thigh infection model or murine infection model or neutropenic mouse or neutropenic mouse model or neutropenic mice).af.**	1,231
**3**	Search **(PK PD index or PK PD indices or pharmacokinetic pharmacodynamic index or pharmacokinetic pharmacodynamic indices or PK PD studies or pharmacokinetic pharmacodynamic studies or PK PD analysis or pharmacokinetic pharmacodynamic analysis or model based analysis or dose fraction or dose fraction study or (dose and fraction)).af.**	45,666
**4**	Search **1 and 2 and 3**	49

Table S2 *Search builder for systematic review databases on the hollow fibre infection model*

**Database** PubMed		
Number	Search terms	Results
**1**	Search **(((((antibiotic agent) OR (antibiotic)) OR (antiinfective agent)) OR (antimicrobial)) OR (antibacterial)) OR (antibacterial agent)**	2,346,251
**2**	Search **((((((((hollow fibre infection model) OR (hollow fiber infection model)) OR (hollow fibre bioreactor)) OR (hollow fiber bioreactor)) OR (hollow fibre reactor)) OR (hollow fiber reactor)) OR (HFIM)) OR (hollow fibre)) OR (hollow fiber)**	9, 025
**3**	Search **(((((((((((PK PD index) OR (PK PD indices)) OR (pharmacokinetic pharmacodynamic index)) OR (pharmacokinetic pharmacodynamic indices)) OR (PK PD studies)) OR (pharmacokinetic pharmacodynamic studies)) OR (PK PD analysis)) OR (pharmacokinetic pharmacodynamic analysis)) OR (model based analysis)) OR (dose fraction)) OR (dose fraction study)) OR (dose and fraction)**	1,049,792
**4**	Search **((#1) AND (#2)) AND (#3)**	278
**5**	Search **((#1) AND (#2)) AND (#3) AND [from 2020/1/1 - 2025/06/01]**	107

**Database** MEDLINE		
Number	Search terms	Results
**1**	Search **(antibiotic agent or antibiotic or antiinfective agent or antimicrobial or antibacterial or antibacterial agent).af.**	651,823
**2**	Search **(hollow fibre infection model or hollow fiber infection model or hollow fibre bioreactor or hollow fiber bioreactor or hollow fibre reactor or hollow fiber reactor or HFIM or (hollow fibre or hollow fiber)).af.**	5,836
**3**	Search **(PK PD index or PK PD indices or pharmacokinetic pharmacodynamic index or pharmacokinetic pharmacodynamic indices or PK PD studies or pharmacokinetic pharmacodynamic studies or PK PD analysis or pharmacokinetic pharmacodynamic analysis or model based analysis or dose fraction study).af.**	2,816
**4**	Search **1 and 2 and 3**	29
**5**	Search **limit 4 to (english language and full text and yr="2020 -Current" and "remove preprint records")**	2

**Database** BIOSIS		
Number	Search terms	Results
**1**	Search **antibiotic agent (Topic) OR antibiotic (Topic) OR antiinfective agent (Topic) OR antimicrobial (Topic) OR antibacterial (Topic) OR antibacterial agent (Topic)**	1,113,115
**2**	Search **hollow fibre infection model (Topic) OR hollow fiber infection model (Topic) OR hollow fibre bioreactor (Topic) OR hollow fiber bioreactor (Topic) OR hollow fibre reactor (Topic) OR hollow fiber reactor (Topic) OR HFIM (Topic) OR hollow fibre (Topic) OR hollow fiber (Topic)**	7,998
**3**	Search **TS=(PK PD index) OR TS=(PK PD indices) OR TS=(pharmacokinetic pharmacodynamic index) OR TS=(pharmacokinetic pharmacodynamic indices) OR TS=(PK PD studies) OR TS=(pharmacokinetic pharmacodynamic studies) OR TS=(PK PD analysis) OR TS=(pharmacokinetic pharmacodynamic analysis) OR TS=(model based analysis) OR TS=(dose fraction) OR TS=(dose fraction study) OR TS=(dose and fraction)**	480,739
**4**	Search **#1 AND #2 AND #3**	143
**5**	Search **#1 AND #2 AND #3** **Timespan: 2019-12-12 to 2025-06-01**	56

**Database** EMBASE		
Number	Search terms	Results
**1**	Search **(antibiotic agent or antibiotic or antiinfective agent or antimicrobial or antibacterial or antibacterial agent).af.**	1,351,735
**2**	Search **(hollow fibre infection model or hollow fiber infection model or hollow fibre bioreactor or hollow fiber bioreactor or hollow fibre reactor or hollow fiber reactor or HFIM or hollow fibre or hollow fiber).af.**	10,626
**3**	Search **(PK PD index or PK PD indices or pharmacokinetic pharmacodynamic index or pharmacokinetic pharmacodynamic indices or PK PD studies or pharmacokinetic pharmacodynamic studies or PK PD analysis or pharmacokinetic pharmacodynamic analysis or model based analysis or dose fraction or dose fraction study or (dose and fraction)).af.**	93,982
**4**	Search **1 and 2 and 3**	33
**5**	Search **limit 4 to (full text and english language and "remove preprint records" and yr="2020 -Current")**	4

**Database** SCOPUS		
Number	Search terms	Results
**1**	Search **(TITLE-ABS-KEY (antibiotic AND agent) OR TITLE-ABS-KEY (antibiotic) OR TITLE-ABS-KEY (antiinfective AND agent) OR TITLE-ABS-KEY (antimicrobial) OR TITLE-ABS-KEY (antibacterial) OR TITLE-ABS-KEY (antibacterial AND agent))**	1,622,254
**2**	Search **(TITLE-ABS-KEY (pk AND pd AND index) OR TITLE-ABS-KEY (pk AND pd AND indices) OR TITLE-ABS-KEY (pharmacokinetic AND pharmacodynamic AND index) OR TITLE-ABS-KEY (pharmacokinetic AND pharmacodynamic AND indices) OR TITLE-ABS-KEY (dose AND fraction) OR TITLE-ABS-KEY (dose AND fraction AND study) OR TITLE-ABS-KEY (dose AND fraction))**	108,632
**3**	Search **(TITLE-ABS-KEY (hollow AND fibre AND infection AND model) OR TITLE-ABS-KEY (hollow AND fiber AND infection AND model) OR TITLE-ABS-KEY (hollow AND fibre AND bioreactor) OR TITLE-ABS-KEY (hollow AND fiber AND bioreactor) OR TITLE-ABS-KEY (hollow AND fibre AND reactor) OR TITLE-ABS-KEY (hollow AND fiber AND reactor) OR TITLE-ABS-KEY (hfim) OR TITLE-ABS-KEY (hollow AND fibre) OR TITLE-ABS-KEY (hollow AND fiber))**	38,065
**4**	Search **((#1) AND (#2)) AND (#3) AND [PUBYEAR > 2019]**	16

Table S3 CAMARADES checklist

CAMARADES checklist	0 (NO)	1 (YES)
1- Publication in peer-reviewed journal		
2- Statement of control of temperature		
3- Randomisation of treatment or control		
4- Allocation concealment		
5- Blinded assessment of outcome		
6- Avoidance of anaesthetics with marked intrinsic properties		
7- Use of animals with hypertension or diabetes		
8- Sample size calculation		
9- Statement of compliance with regulatory requirements		
10- Statement regarding possible conflict of interest		

Table S4 Quality Assessment of Murine Thigh Infection Model Studies using CAMARADES Quality Checklist

Study	1	2	3	4	5	6	7	8	9	10	Total score
Lepak *et al.* (2017) [1]	1	0	0	0	0	0	0	0	1	0	2
XIAO and XIAO (2008) [2]	1	0	0	0	0	0	0	0	1	0	2
Ji *et al.* (2020) [3]	1	0	0	0	0	0	0	0	1	1	3
Takata *et al.* (2004) [4]	1	0	0	0	0	0	0	0	0	0	1
Nakamura *et al.* (2019) [5]	1	0	0	0	0	0	0	0	1	0	2
Takemura *et al.* (2021) [6]	1	1	0	0	0	0	0	0	1	1	4
Guo *et al.* (2016) [7]	1	0	0	0	0	0	0	0	1	0	2
Shan *et al.* (2014) [8]	1	0	0	0	0	0	0	0	1	0	2
Shan and Wang (2017) [9]	1	0	0	0	0	0	0	0	1	0	2
Wang *et al.* (2014) [10]	1	0	0	0	0	0	0	0	1	0	2
Craig and Andes (2008) [11]	1	0	0	0	0	0	0	0	1	0	2
Craig and Andes (2013) [12]	1	0	0	0	0	0	0	0	1	0	2
Dudhani *et al.* (2010) [13]	1	1	0	0	0	0	0	0	1	0	3
Dudhani *et al.* (2010) [14]	1	0	0	0	0	0	0	0	1	0	2
Zhao *et al.* (2017) [15]	1	0	0	0	0	0	0	0	1	0	2
Tashiro *et al.* (2021) [16]	1	1	0	0	0	0	0	0	1	0	3
Roelofsen *et al.* (2022) [17]	1	0	0	0	0	0	0	0	1	0	2
Chavan *et al.* (2023) [18]	1	1	0	0	0	0	0	0	1	0	3

Table S4 Quality Assessment of Murine Thigh Infection Model Studies using CAMARADES Quality Checklist

**Study**	**1**	**2**	**3**	**4**	**5**	**6**	**7**	**8**	**9**	**10**	**Total score**
Andes and Craig (2003) [19]	1	0	0	0	0	0	0	0	1	0	2
Andes and Craig (2002) [20]	1	0	0	0	0	0	0	0	1	0	2
Bulik *et al.* (2017) [21]	1	0	0	0	0	0	0	0	1	0	2
Ferrari *et al.* (2003) [22]	1	1	0	0	0	0	0	0	1	0	3
Wicha *et al.* (2019) [23]	1	0	0	0	0	0	0	0	1	1	3
Growcott *et al.* (2019) [24]	1	1	0	0	0	0	0	0	1	1	4
Kristoffersson *et al.* (2016) [25]	1	0	0	0	0	0	0	0	0	0	1
Fratoni *et al.* (2022) [26]	1	1	1	0	0	0	0	1	1	1	6
Melchers *et al.* (2019) [27]	1	0	0	0	0	0	0	0	0	1	2
Zhao *et al.* (2018) [28]	1	0	0	0	0	1	0	0	1	0	3
Andes *et al.* (2009) [29]	1	0	0	0	0	1	0	0	1	0	3
Umezaki *et al.* (2022) [30]	1	0	0	0	0	1	0	0	1	1	4
Lepak *et al.* (2020) [31]	1	0	0	0	0	1	0	0	1	0	3
Andes and Craig (2006) [32]	1	0	0	0	0	1	0	0	1	0	3
Hirai *et al.* (2016) [33]	1	0	0	0	0	1	0	0	1	1	4
Griffith *et al.* (2008) [34]	1	0	0	0	0	1	0	0	1	0	3
Yokoyama *et al.* (2014) [35]	1	0	0	0	0	1	0	0	1	1	4
Hegde *et al.* (2012) [36]	1	0	0	0	0	1	0	0	1	0	3
Liu *et al.* (2023) [37]	1	0	0	0	0	0	0	0	0	1	2

Table S4 Quality Assessment of Murine Thigh Infection Model Studies using CAMARADES Quality Checklist

**Study**	**1**	**2**	**3**	**4**	**5**	**6**	**7**	**8**	**9**	**10**	**Total score**
Watanabe *et al*. (2021) [38]	1	0	0	0	0	0	0	0	1	1	3
Hegde *et al*. (2004) [39]	1	0	0	0	0	0	0	0	1	0	2
Sugihara *et al*. (2010) [40]	1	0	0	0	0	0	0	0	0	0	1
Louie *et al*. (2011) [41]	1	0	0	0	0	0	0	0	0	1	2
Hagihara *et al*. (2020) [42]	1	0	0	0	0	0	0	0	1	1	3
Lepak *et al*. (2015) [43]	1	0	0	0	0	0	0	0	1	0	2
Andes and Craig (2006) [44]	1	0	0	0	0	0	0	0	1	0	2
Vogelman *et al*. (1988) [45]	1	0	0	0	0	0	0	0	1	1	3
He *et al*. (2023) [46]	1	0	0	0	0	1	0	0	1	0	3
van den Berg *et al*. (2025) [47]	1	1	0	0	0	0	0	0	1	1	4
Eguchi *et al.* (2009) [48]	1	1	0	0	0	0	0	0	1	1	4
Andes *et al.* (2002) [49]	1	1	0	0	0	0	0	0	1	1	4
Van Wart *et al.* (2009) [50]	1	0	0	0	0	0	0	0	1	1	3

This table presents the results of the quality assessment for Murine Thigh Infection Model studies using the CAMARADES Quality Checklist. The checklist comprises 10 elements evaluating various aspects of study design, conduct, and reporting.

Each row corresponds to a specific study, and columns 1 to 10 represent the checklist elements (1- Publication in peer-reviewed journal, 2- Statement of control of temperature, 3- Randomisation of treatment or control, 4- Allocation concealment, 5- Blinded assessment of outcome, 6- Avoidance of anaesthetics with marked intrinsic properties, 7- Use of animals with hypertension or diabetes, 8- Sample size calculation, 9- Statement of compliance with regulatory

Table S4 Quality Assessment of Murine Thigh Infection Model Studies using CAMARADES Quality Checklist

Study	1	2	3	4	5	6	7	8	9	10	Total score

requirements, 10- Statement regarding possible conflict of interest). Each element is assessed with a binary scoring system (1 for present, 0 for absent), and the 'Total score' column provides an aggregate score for each study based on the checklist criteria. A higher total score indicates a higher overall quality of the study.

Figures S1 PKPD index outputs for remodelled studies

Lepak et al. (2017) PMID:28396549 Drug:Fosfomycin

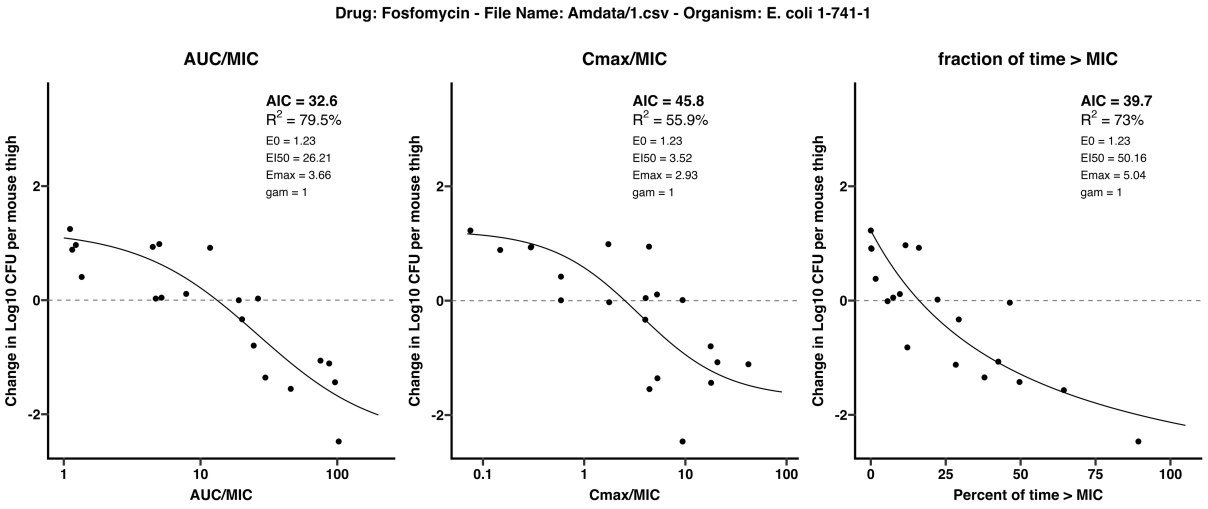

#	Model	Model Number	R²	AIC	EC50 hat	Emax hat	E0 hat	Gamma hat	Status	Best Fit Model	Optimal PKPD Index	Target for Stasis	Log1 Kill	Log2 Kill
1	AUC/MIC	1	0.771	32.9	15.410	2.933 FIX	1.226 FIX	1 FIX	successful	3	-			
2	AUC/MIC	2	0.776	34.4	15.950	2.933 FIX	1.226 FIX	1.184	successful	3	-			
3	AUC/MIC	3	0.795	32.6	26.210	3.663	1.226 FIX	1 FIX	successful	3	AUC/MIC	13.2	40.6	193.5
4	AUC/MIC	4	0.798	33.9	79.880	5.52	1.226 FIX	0.6941	successful	3	-			
5	AUC/MIC	5	0.800	33.6	38.420	3.76	0.9907	1 FIX	successful	3	-			
6	AUC/MIC	6	0.808	34.8	23.410	2.229	0.6607	4.06	successful	3	-			
7	AUC/MIC	7	0.792	33.6	23.730	2.933 FIX	0.9594	1 FIX	successful	3	-			
8	AUC/MIC	8	0.802	33.4	27.480	2.933 FIX	0.8473	1.451	successful	3	-			
9	Cmax/MIC	1	0.559	45.8	3.518	2.933 FIX	1.226 FIX	1 FIX	successful	1	-			
10	Cmax/MIC	2	0.557	47.4	3.338	2.933 FIX	1.226 FIX	0.805	successful	1	-			
11	Cmax/MIC	3	0.557	47.6	2.729	2.687	1.226 FIX	1 FIX	successful	1	-			
12	Cmax/MIC	4	0.557	49.4	3.315	2.926	1.226 FIX	0.8075	successful	1	-			
13	Cmax/MIC	5	0.559	49.3	3.587	2.59	1.034	1 FIX	successful	1	-			
14	Cmax/MIC	6	0.568	50.9	4.232	1.853	0.6876	4.219	successful	1	-			
15	Cmax/MIC	7	0.557	47.6	4.505	2.933 FIX	1.086	1 FIX	successful	1	-			
16	Cmax/MIC	8	0.558	49.4	3.740	2.933 FIX	1.172	0.8192	successful	1	-			
17	T>MIC	1	0.659	40.7	15.670	2.933 FIX	1.226 FIX	1 FIX	successful	3	-			
18	T>MIC	2	0.664	42.6	16.330	2.933 FIX	1.226 FIX	1.109	successful	3	-			
19	T>MIC	3	0.730	39.7	50.160	5.039	1.226 FIX	1 FIX	successful	3	-			
20	T>MIC	4							unsuccessful	3	-			
21	T>MIC	5	0.749	37.9	203.700	10.11	0.7729	1 FIX	successful	3	-			
22	T>MIC	6							unsuccessful	3	-			
23	T>MIC	7	0.702	40.3	28.080	2.933 FIX	0.8399	1 FIX	successful	3	-			
24	T>MIC	8	0.718	40.4	35.270	2.933 FIX	0.6544	1.743	successful	3	-			

XIAO and XIAO (2008) PMID:18817632 Drug:Antofloxacin

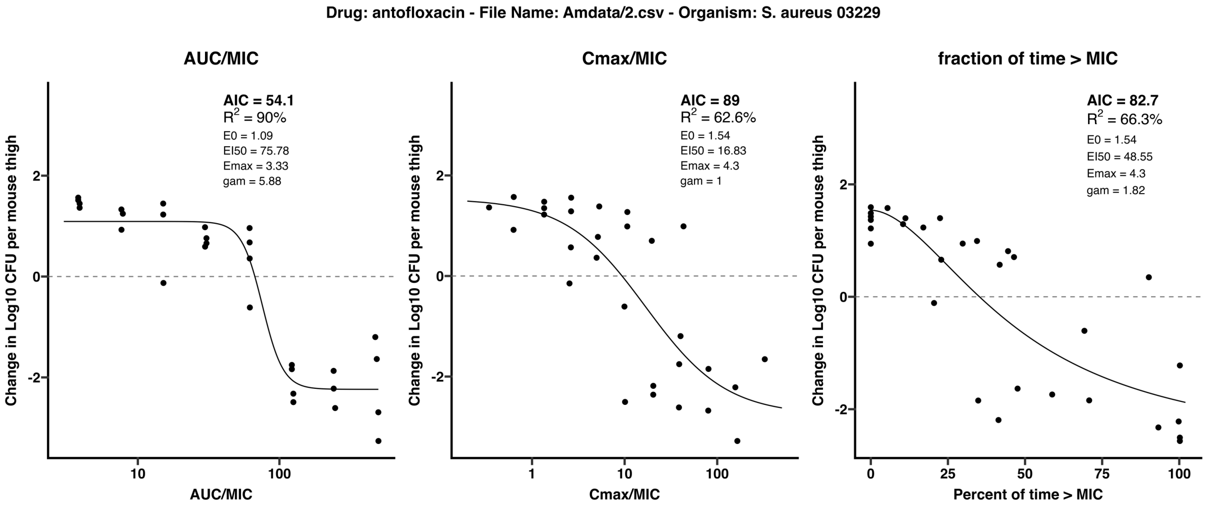

#	Model	Model Number	R²	AIC	EC50 hat	Emax hat	E0 hat	Gamma hat	Status	Best Fit Model	Optimal PKPD Index	Target for Stasis	Log1 Kill	Log2 Kill
1	AUC/MIC	1	0.835	65.9	75.08	4.303 FIX	1.54 FIX	1 FIX	successful	6	-			
2	AUC/MIC	2	0.869	60.2	75.71	4.303 FIX	1.54 FIX	1.871	successful	6	-			
3	AUC/MIC	3	0.835	65.5	104.50	5.011	1.54 FIX	1 FIX	successful	6	-			
4	AUC/MIC	4	0.883	60.8	70.83	3.938	1.54 FIX	2.772	successful	6	-			
5	AUC/MIC	5	0.837	66.8	88.31	5.095	1.748	1 FIX	successful	6	-			
6	AUC/MIC	6	0.900	54.1	75.78	3.328	1.092	5.881	successful	6	AUC/MIC	67	83	117
7	AUC/MIC	7	0.834	67.8	71.83	4.303 FIX	1.582	1 FIX	successful	6	-			
8	AUC/MIC	8	0.870	61.8	80.92	4.303 FIX	1.443	1.943	successful	6	-			
9	Cmax/MIC	1	0.626	89.0	16.83	4.303 FIX	1.54 FIX	1 FIX	successful	1	-			
10	Cmax/MIC	2	0.627	91.0	16.72	4.303 FIX	1.54 FIX	1.039	successful	1	-			
11	Cmax/MIC	3	0.627	91.0	16.18	4.233	1.54 FIX	1 FIX	successful	1	-			
12	Cmax/MIC	4	0.629	92.8	13.83	3.985	1.54 FIX	1.178	successful	1	-			
13	Cmax/MIC	5	0.627	93.0	15.53	4.257	1.585	1 FIX	successful	1	-			
14	Cmax/MIC	6	0.630	94.7	14.22	3.714	1.378	1.35	successful	1	-			
15	Cmax/MIC	7	0.627	91.0	15.70	4.303 FIX	1.597	1 FIX	successful	1	-			
16	Cmax/MIC	8	0.627	92.9	15.79	4.303 FIX	1.588	1.033	successful	1	-			
17	T>MIC	1	0.614	85.4	49.84	4.303 FIX	1.54 FIX	1 FIX	successful	2	-			
18	T>MIC	2	0.663	82.7	48.55	4.303 FIX	1.54 FIX	1.821	successful	2	-			
19	T>MIC	3	0.654	83.4	247.30	12.13	1.54 FIX	1 FIX	successful	2	-			
20	T>MIC	4	0.663	84.7	47.48	4.231	1.54 FIX	1.859	successful	2	-			
21	T>MIC	5	0.654	85.4	273.30	12.91	1.503	1 FIX	successful	2	-			
22	T>MIC	6	0.663	86.5	46.45	3.886	1.404	2.094	successful	2	-			
23	T>MIC	7	0.616	87.4	51.75	4.303 FIX	1.5	1 FIX	successful	2	-			
24	T>MIC	8	0.663	84.5	52.10	4.303 FIX	1.428	1.84	successful	2	-			

XIAO and XIAO (2008) PMID:18817632 Drug:Antofloxacin

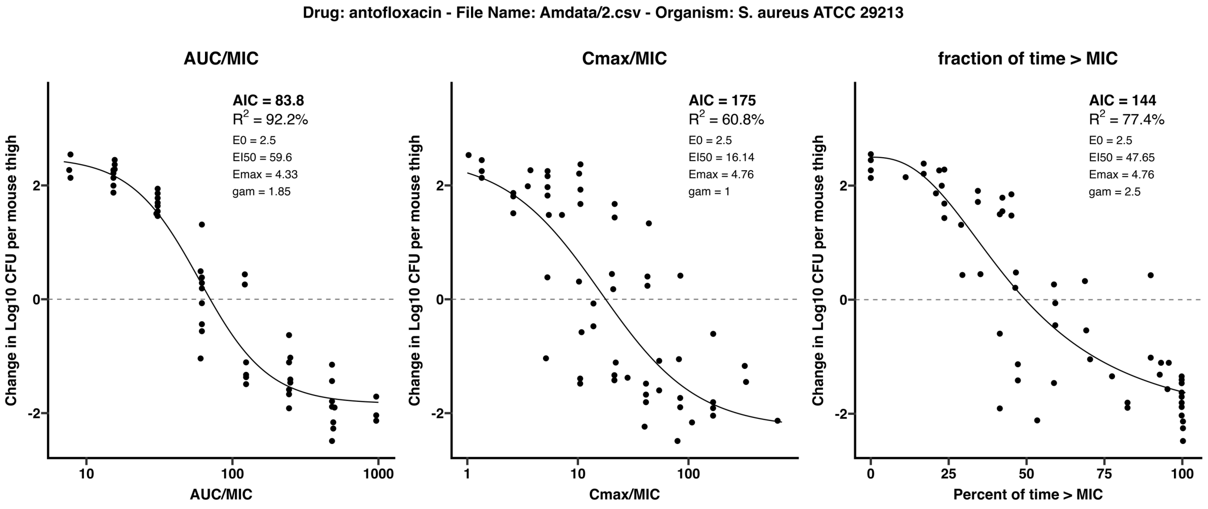

#	Model	Model Number	R²	AIC	EC50 hat	Emax hat	E0 hat	Gamma hat	Status	Best Fit Model	Optimal PKPD Index	Target for Stasis	Log1 Kill	Log2 Kill
1	AUC/MIC	1	0.909	105.0	74.11	4.762 FIX	2.498 FIX	1 FIX	successful	4	-			
2	AUC/MIC	2	0.916	87.0	72.23	4.762 FIX	2.498 FIX	1.445	successful	4	-			
3	AUC/MIC	3	0.899	101.0	94.73	5.282	2.498 FIX	1 FIX	successful	4	-			
4	AUC/MIC	4	0.922	83.8	59.60	4.332	2.498 FIX	1.847	successful	4	AUC/MIC	70.5	129.5	1,000
5	AUC/MIC	5	0.914	89.6	53.03	5.962	3.504	1 FIX	successful	4	-			
6	AUC/MIC	6	0.922	85.6	58.27	4.481	2.611	1.732	successful	4	-			
7	AUC/MIC	7	0.834	67.8	71.83	4.303 FIX	1.582	1 FIX	successful	4	-			
8	AUC/MIC	8	0.870	61.8	80.92	4.303 FIX	1.443	1.943	successful	4	-			
9	Cmax/MIC	1	0.608	175.0	16.14	4.762 FIX	2.498 FIX	1 FIX	successful	1	-			
10	Cmax/MIC	2	0.608	177.0	16.14	4.762 FIX	2.498 FIX	0.9863	successful	1	-			
11	Cmax/MIC	3	0.611	176.0	14.08	4.517	2.498 FIX	1 FIX	successful	1	-			
12	Cmax/MIC	4	0.614	178.0	12.23	4.237	2.498 FIX	1.24	successful	1	-			
13	Cmax/MIC	5	0.613	178.0	11.15	4.792	2.865	1 FIX	successful	1	-			
14	Cmax/MIC	6	0.614	180.0	12.02	4.301	2.548	1.212	successful	1	-			
15	Cmax/MIC	7	0.613	176.0	11.25	4.762 FIX	2.84	1 FIX	successful	1	-			
16	Cmax/MIC	8	0.613	178.0	11.10	4.762 FIX	2.858	1.033	successful	1	-			
17	T>MIC	1	0.678	175.0	42.39	4.762 FIX	2.498 FIX	1 FIX	successful	2	-			
18	T>MIC	2	0.774	144.0	47.65	4.762 FIX	2.498 FIX	2.495	successful	2	-			
19	T>MIC	3	0.754	150.0	709.70	34.54	2.498 FIX	1 FIX	successful	2	-			
20	T>MIC	4	0.774	146.0	47.34	4.733	2.498 FIX	2.522	successful	2	-			
21	T>MIC	5	0.756	151.0	316.10	18.83	2.77	1 FIX	successful	2	-			
22	T>MIC	6	0.775	148.0	47.36	4.563	2.412	2.67	successful	2	-			
23	T>MIC	7	0.690	177.0	47.09	4.762 FIX	2.364	1 FIX	successful	2	-			
24	T>MIC	8	0.774	146.0	48.52	4.762 FIX	2.457	2.487	successful	2	-			

Ji et al. (2020) PMID:31844001 Drug:Benapenem

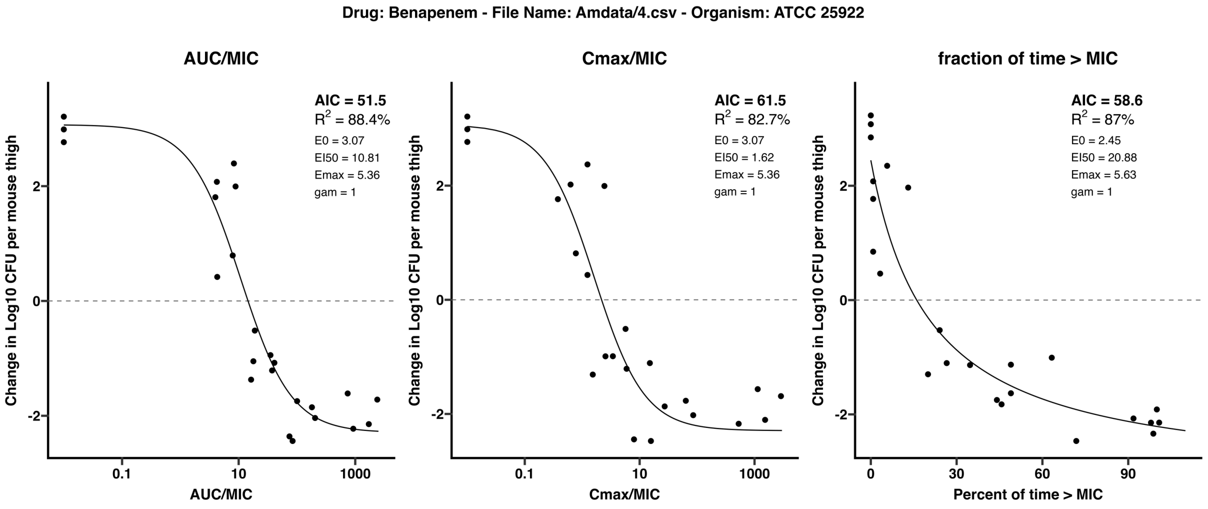

#	Model	Model Number	R²	AIC	EC50 hat	Emax hat	E0 hat	Gamma hat	Status	Best Fit Model	Optimal PKPD Index	Target for Stasis	Log1 Kill	Log2 Kill
1	AUC/MIC	1	0.884	51.5	10.810	5.363 FIX	3.07 FIX	1 FIX	successful	1	AUC/MIC	14.47	34.03	187.05
2	AUC/MIC	2	0.896	51.6	11.290	5.363 FIX	3.07 FIX	1.301	successful	1	-			
3	AUC/MIC	3	0.884	53.5	10.830	5.365	3.07 FIX	1 FIX	successful	1	-			
4	AUC/MIC	4	0.897	52.7	10.440	5.138	3.07 FIX	1.447	successful	1	-			
5	AUC/MIC	5	0.884	55.5	10.660	5.401	3.108	1 FIX	successful	1	-			
6	AUC/MIC	6	0.898	54.3	11.320	4.865	2.823	1.586	successful	1	-			
7	AUC/MIC	7	0.884	53.5	10.700	5.363 FIX	3.082	1 FIX	successful	1	-			
8	AUC/MIC	8	0.896	53.4	10.600	5.363 FIX	3.158	1.327	successful	1	-			
9	Cmax/MIC	1	0.827	61.5	1.624	5.363 FIX	3.07 FIX	1 FIX	successful	1	-			
10	Cmax/MIC	2	0.826	63.5	1.621	5.363 FIX	3.07 FIX	0.9916	successful	1	-			
11	Cmax/MIC	3	0.828	62.8	1.433	5.13	3.07 FIX	1 FIX	successful	1	-			
12	Cmax/MIC	4	0.829	64.6	1.428	5.068	3.07 FIX	1.129	successful	1	-			
13	Cmax/MIC	5	0.828	64.8	1.450	5.106	3.044	1 FIX	successful	1	-			
14	Cmax/MIC	6	0.829	66.6	1.505	4.934	2.943	1.168	successful	1	-			
15	Cmax/MIC	7	0.827	63.1	1.394	5.363 FIX	3.233	1 FIX	successful	1	-			
16	Cmax/MIC	8	0.828	65.1	1.396	5.363 FIX	3.239	1.027	successful	1	-			
17	T>MIC	1	0.850	59.6	9.162	5.363 FIX	3.07 FIX	1 FIX	successful	5	-			
18	T>MIC	2	0.848	61.1	7.431	5.363 FIX	3.07 FIX	0.8326	successful	5	-			
19	T>MIC	3	0.864	60.6	13.430	5.884	3.07 FIX	1 FIX	successful	5	-			
20	T>MIC	4	0.880	56.6	1,811.000	20.68	3.07 FIX	0.3613	successful	5	-			
21	T>MIC	5	0.870	58.6	20.880	5.629	2.446	1 FIX	successful	5	-			
22	T>MIC	6	0.880	58.6	794.500	17.09	2.984	0.3875	successful	5	-			
23	T>MIC	7	0.870	56.8	18.500	5.363 FIX	2.414	1 FIX	successful	5	-			
24	T>MIC	8	0.869	58.7	19.440	5.363 FIX	2.398	1.063	successful	5	-			

Ji et al. (2020) PMID:31844001 Drug:Benapenem

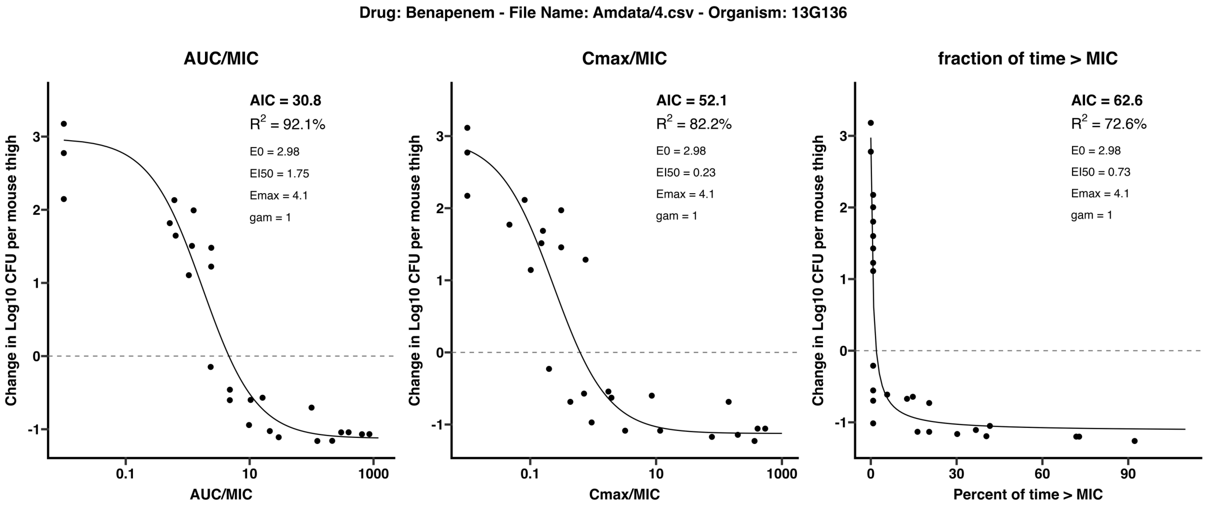

#	Model	Model Number	R²	AIC	EC50 hat	Emax hat	E0 hat	Gamma hat	Status	Best Fit Model	Optimal PKPD Index	Target for Stasis	Log1 Kill	Log2 Kill
1	AUC/MIC	1	0.921	30.8	1.7460	4.099 FIX	2.975 FIX	1 FIX	successful	1	AUC/MIC	4.62	55.97	1,200
2	AUC/MIC	2	0.926	32.0	1.7760	4.099 FIX	2.975 FIX	1.145	successful	1	-			
3	AUC/MIC	3	0.922	32.7	1.7960	4.143	2.975 FIX	1 FIX	successful	1	-			
4	AUC/MIC	4	0.926	34.0	1.7620	4.085	2.975 FIX	1.154	successful	1	-			
5	AUC/MIC	5	0.923	33.9	2.0160	3.962	2.781	1 FIX	successful	1	-			
6	AUC/MIC	6	0.930	33.5	2.1290	3.676	2.597	1.372	successful	1	-			
7	AUC/MIC	7	0.923	32.2	1.9490	4.099 FIX	2.882	1 FIX	successful	1	-			
8	AUC/MIC	8	0.927	33.6	1.9290	4.099 FIX	2.899	1.128	successful	1	-			
9	Cmax/MIC	1	0.822	52.1	0.2344	4.099 FIX	2.975 FIX	1 FIX	successful	1	-			
10	Cmax/MIC	2	0.823	53.5	0.2278	4.099 FIX	2.975 FIX	0.8454	successful	1	-			
11	Cmax/MIC	3	0.821	53.9	0.2203	4.013	2.975 FIX	1 FIX	successful	1	-			
12	Cmax/MIC	4	0.823	55.5	0.2246	4.079	2.975 FIX	0.8533	successful	1	-			
13	Cmax/MIC	5	0.823	55.4	0.2649	3.783	2.719	1 FIX	successful	1	-			
14	Cmax/MIC	6	0.823	57.4	0.2529	3.886	2.799	0.9274	successful	1	-			
15	Cmax/MIC	7	0.822	54.1	0.2333	4.099 FIX	2.978	1 FIX	successful	1	-			
16	Cmax/MIC	8	0.823	55.5	0.2284	4.099 FIX	2.973	0.8453	successful	1	-			
17	T>MIC	1	0.726	62.6	0.7304	4.099 FIX	2.975 FIX	1 FIX	successful	1	-			
18	T>MIC	2	0.726	64.6	0.7250	4.099 FIX	2.975 FIX	0.962	successful	1	-			
19	T>MIC	3	0.726	64.6	0.7444	4.129	2.975 FIX	1 FIX	successful	1	-			
20	T>MIC	4							unsuccessful	1	-			
21	T>MIC	5	0.726	66.6	0.7464	4.124	2.969	1 FIX	successful	1	-			
22	T>MIC	6	0.728	68.4	0.8343	4.385	2.981	0.6888	successful	1	-			
23	T>MIC	7	0.726	64.6	0.7520	4.099 FIX	2.948	1 FIX	successful	1	-			
24	T>MIC	8							unsuccessful	1	-			

Ji et al. (2020) PMID:31844001 Drug:Benapenem

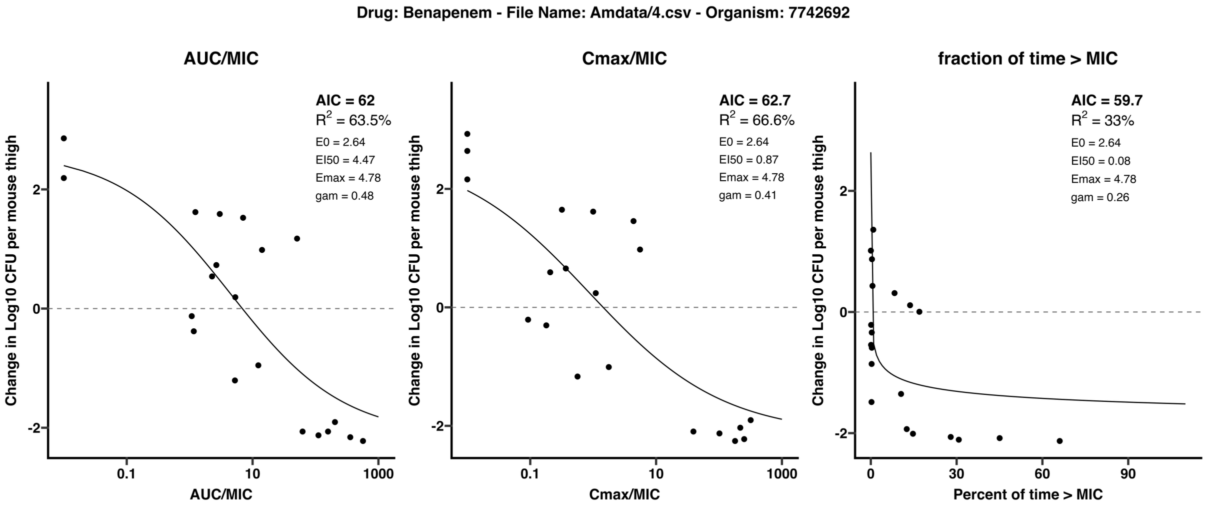

#	Model	Model Number	R²	AIC	EC50 hat	Emax hat	E0 hat	Gamma hat	Status	Best Fit Model	Optimal PKPD Index	Target for Stasis	Log1 Kill	Log2 Kill
1	AUC/MIC	1	0.566	66.2	4.89900	4.78 FIX	2.637 FIX	1 FIX	successful	2	-			
2	AUC/MIC	2	0.635	62.0	4.47300	4.78 FIX	2.637 FIX	0.4835	successful	2	-			
3	AUC/MIC	3	0.547	66.6	2.87200	4.143	2.637 FIX	1 FIX	successful	2	-			
4	AUC/MIC	4	0.664	62.2	605.20000	10.33	2.637 FIX	0.2606	successful	2	-			
5	AUC/MIC	5	0.596	65.9	34.29000	3.615	1.147	1 FIX	successful	2	-			
6	AUC/MIC	6							unsuccessful	2	-			
7	AUC/MIC	7							unsuccessful	2	-			
8	AUC/MIC	8	0.646	63.4	14.38000	4.78 FIX	2.08	0.4936	successful	2	-			
9	Cmax/MIC	1	0.544	70.4	0.71250	4.78 FIX	2.637 FIX	1 FIX	successful	2	-			
10	Cmax/MIC	2	0.666	62.7	0.86760	4.78 FIX	2.637 FIX	0.4086	successful	2	-			
11	Cmax/MIC	3	0.524	69.6	0.25530	3.88	2.637 FIX	1 FIX	successful	2	-			
12	Cmax/MIC	4	0.683	63.3	23.88000	7.419	2.637 FIX	0.2592	successful	2	-			
13	Cmax/MIC	5	0.621	66.9	15.13000	3.466	1.052	1 FIX	successful	2	-			
14	Cmax/MIC	6							unsuccessful	2	-			
15	Cmax/MIC	7	0.587	67.2	69.80000	4.78 FIX	0.9939	1 FIX	successful	2	-			
16	Cmax/MIC	8	0.670	64.3	2.80700	4.78 FIX	2.197	0.4071	successful	2	-			
17	T>MIC	1	0.206	71.3	0.16880	4.78 FIX	2.637 FIX	1 FIX	successful	2	-			
18	T>MIC	2	0.330	59.7	0.07565	4.78 FIX	2.637 FIX	0.2599	successful	2	T>MIC	1	7	110
19	T>MIC	3	0.175	63.9	0.03304	3.617	2.637 FIX	1 FIX	successful	2	-			
20	T>MIC	4							unsuccessful	2	-			
21	T>MIC	5	0.454	57.6	39.17000	3.862	0.008438	1 FIX	successful	2	-			
22	T>MIC	6							unsuccessful	2	-			
23	T>MIC	7	0.452	55.7	56.66000	4.78 FIX	-0.006443	1 FIX	successful	2	-			
24	T>MIC	8							unsuccessful	2	-			

Ji et al. (2020) PMID:31844001 Drug:Benapenem

Drug: Benapenem - File Name: Amdata/4.csv - Organism: ATCC 700603

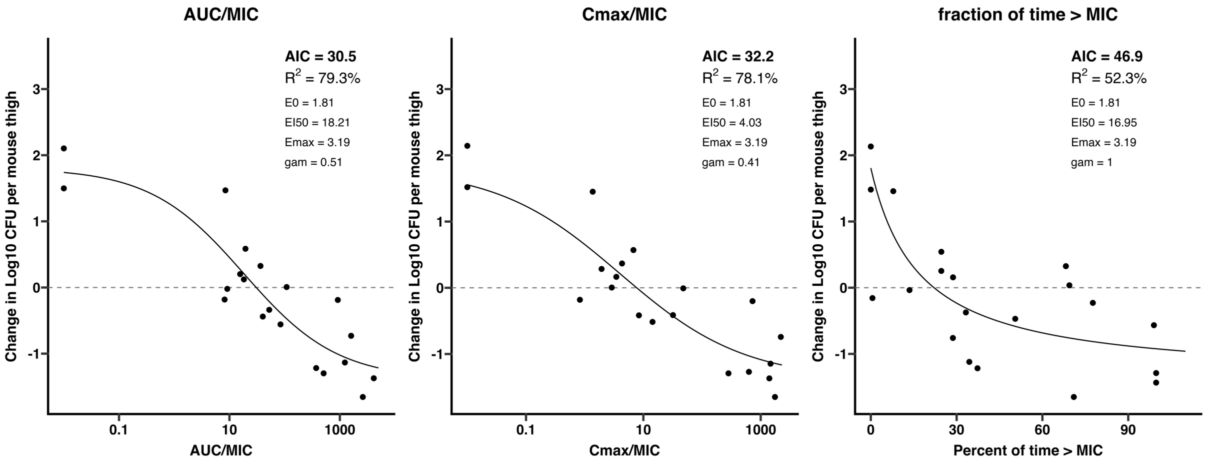

#	Model	Model Number	R²	AIC	EC50 hat	Emax hat	E0 hat	Gamma hat	Status	Best Fit Model	Optimal PKPD Index	Target for Stasis	Log1 Kill	Log2 Kill
1	AUC/MIC	1	0.758	35.2	20.810	3.193 FIX	1.807 FIX	1 FIX	successful	2	-			
2	AUC/MIC	2	0.793	30.5	18.210	3.193 FIX	1.807 FIX	0.5117	successful	2	AUC/MIC	30.58	879.46	5,000
3	AUC/MIC	3	0.760	33.5	13.800	2.816	1.807 FIX	1 FIX	successful	2	-			
4	AUC/MIC	4	0.794	32.4	23.830	3.388	1.807 FIX	0.4463	successful	2	-			
5	AUC/MIC	5	0.761	35.4	15.950	2.681	1.656	1 FIX	successful	2	-			
6	AUC/MIC	6	0.795	34.3	25.160	3.84	2.01	0.3647	successful	2	-			
7	AUC/MIC	7	0.758	35.2	12.550	3.193 FIX	2.088	1 FIX	successful	2	-			
8	AUC/MIC	8	0.793	32.5	18.790	3.193 FIX	1.795	0.5089	successful	2	-			
9	Cmax/MIC	1	0.725	40.3	4.098	3.193 FIX	1.807 FIX	1 FIX	successful	2	-			
10	Cmax/MIC	2	0.781	32.2	4.031	3.193 FIX	1.807 FIX	0.4099	successful	2	-			
11	Cmax/MIC	3	0.723	36.9	2.381	2.722	1.807 FIX	1 FIX	successful	2	-			
12	Cmax/MIC	4	0.781	34.1	3.594	3.13	1.807 FIX	0.4293	successful	2	-			
13	Cmax/MIC	5	0.726	38.5	3.211	2.492	1.546	1 FIX	successful	2	-			
14	Cmax/MIC	6							unsuccessful	2	-			
15	Cmax/MIC	7	0.718	39.4	1.997	3.193 FIX	2.173	1 FIX	successful	2	-			
16	Cmax/MIC	8							unsuccessful	2	-			
17	T>MIC	1	0.523	46.9	16.950	3.193 FIX	1.807 FIX	1 FIX	successful	1	-			
18	T>MIC	2	0.524	47.4	4.456	3.193 FIX	1.807 FIX	0.3711	successful	1	-			
19	T>MIC	3	0.520	48.8	14.920	3.076	1.807 FIX	1 FIX	successful	1	-			
20	T>MIC	4							unsuccessful	1	-			
21	T>MIC	5	0.527	49.2	23.820	2.719	1.299	1 FIX	successful	1	-			
22	T>MIC	6	0.539	50.7	18.760	2.018	1.206	2.451	successful	1	-			
23	T>MIC	7	0.523	47.6	34.670	3.193 FIX	1.329	1 FIX	successful	1	-			
24	T>MIC	8							unsuccessful	1	-			

Ji et al. (2020) PMID:31844001 Drug:Benapenem

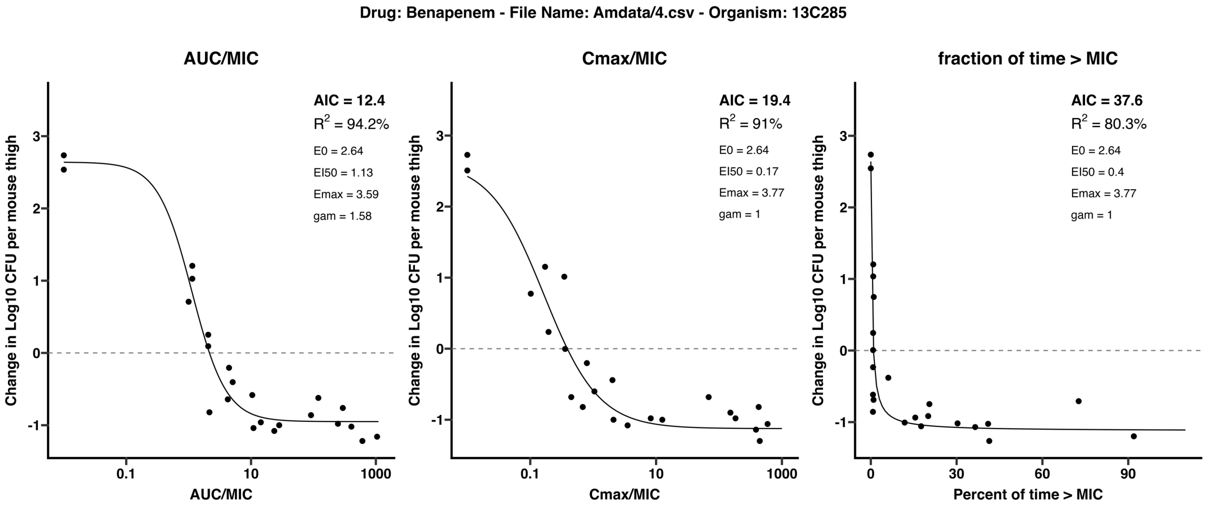

#	Model	Model Number	R²	AIC	EC50 hat	Emax hat	E0 hat	Gamma hat	Status	Best Fit Model	Optimal PKPD Index	Target for Stasis	Log1 Kill	Log2 Kill
1	AUC/MIC	1	0.931	12.8	1.0470	3.765 FIX	2.641 FIX	1 FIX	successful	4	-			
2	AUC/MIC	2	0.938	13.7	1.1630	3.765 FIX	2.641 FIX	1.234	successful	4	-			
3	AUC/MIC	3	0.932	14.3	0.9826	3.697	2.641 FIX	1 FIX	successful	4	-			
4	AUC/MIC	4	0.942	12.4	1.1310	3.591	2.641 FIX	1.585	successful	4	AUC/MIC	2.16	1,100	1,100
5	AUC/MIC	5	0.932	16.1	0.9493	3.774	2.72	1 FIX	successful	4	-			
6	AUC/MIC	6	0.942	14.4	1.1300	3.595	2.644	1.584	successful	4	-			
7	AUC/MIC	7	0.932	14.1	0.9512	3.765 FIX	2.712	1 FIX	successful	4	-			
8	AUC/MIC	8	0.942	13.1	1.0810	3.765 FIX	2.782	1.481	successful	4	-			
9	Cmax/MIC	1	0.910	19.4	0.1653	3.765 FIX	2.641 FIX	1 FIX	successful	1	-			
10	Cmax/MIC	2	0.910	21.4	0.1639	3.765 FIX	2.641 FIX	0.9824	successful	1	-			
11	Cmax/MIC	3	0.913	20.4	0.1500	3.663	2.641 FIX	1 FIX	successful	1	-			
12	Cmax/MIC	4	0.912	22.3	0.1530	3.646	2.641 FIX	1.069	successful	1	-			
13	Cmax/MIC	5	0.914	21.8	0.1330	3.868	2.856	1 FIX	successful	1	-			
14	Cmax/MIC	6	0.914	23.8	0.1257	3.942	2.921	0.9509	successful	1	-			
15	Cmax/MIC	7	0.913	19.9	0.1386	3.765 FIX	2.761	1 FIX	successful	1	-			
16	Cmax/MIC	8	0.913	21.9	0.1401	3.765 FIX	2.764	1.024	successful	1	-			
17	T>MIC	1	0.803	37.6	0.4027	3.765 FIX	2.641 FIX	1 FIX	successful	1	-			
18	T>MIC	2	0.812	38.1	0.2481	3.765 FIX	2.641 FIX	0.6212	successful	1	-			
19	T>MIC	3	0.805	39.0	0.3545	3.64	2.641 FIX	1 FIX	successful	1	-			
20	T>MIC	4	0.815	39.7	0.2685	4.238	2.641 FIX	0.3734	successful	1	-			
21	T>MIC	5	0.805	40.9	0.3571	3.623	2.624	1 FIX	successful	1	-			
22	T>MIC	6							unsuccessful	1	-			
23	T>MIC	7	0.805	39.1	0.3487	3.765 FIX	2.744	1 FIX	successful	1	-			
24	T>MIC	8							unsuccessful	1	-			

Ji et al. (2020) PMID:31844001 Drug:Benapenem

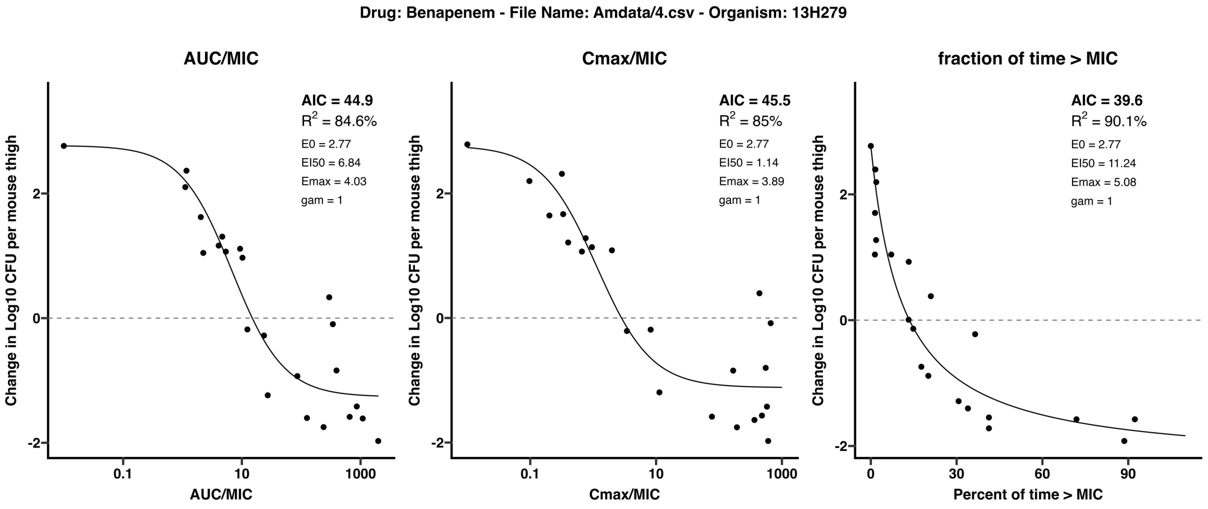

#	Model	Model Number	R²	AIC	EC50 hat	Emax hat	E0 hat	Gamma hat	Status	Best Fit Model	Optimal PKPD Index	Target for Stasis	Log1 Kill	Log2 Kill
1	AUC/MIC	1	0.845	48.2	9.2820	4.503 FIX	2.77 FIX	1 FIX	successful	3	-			
2	AUC/MIC	2	0.839	46.2	10.3300	4.503 FIX	2.77 FIX	0.6426	successful	3	-			
3	AUC/MIC	3	0.846	44.9	6.8390	4.033	2.77 FIX	1 FIX	successful	3	-			
4	AUC/MIC	4	0.847	46.7	7.1370	4.095	2.77 FIX	0.888	successful	3	-			
5	AUC/MIC	5	0.847	46.8	7.6340	3.895	2.617	1 FIX	successful	3	-			
6	AUC/MIC	6	0.847	48.7	7.3250	4.038	2.722	0.9088	successful	3	-			
7	AUC/MIC	7	0.843	46.8	5.6430	4.503 FIX	3.164	1 FIX	successful	3	-			
8	AUC/MIC	8	0.846	47.1	6.4190	4.503 FIX	3.058	0.7459	successful	3	-			
9	Cmax/MIC	1	0.853	53.2	1.7090	4.503 FIX	2.77 FIX	1 FIX	successful	3	-			
10	Cmax/MIC	2	0.828	49.1	2.4880	4.503 FIX	2.77 FIX	0.5038	successful	3	-			
11	Cmax/MIC	3	0.850	45.5	1.1390	3.888	2.77 FIX	1 FIX	successful	3	-			
12	Cmax/MIC	4	0.853	46.7	1.2470	3.957	2.77 FIX	0.8018	successful	3	-			
13	Cmax/MIC	5	0.853	46.8	1.5790	3.573	2.425	1 FIX	successful	3	-			
14	Cmax/MIC	6	0.854	48.6	1.4180	3.775	2.599	0.864	successful	3	-			
15	Cmax/MIC	7	0.839	49.3	0.8106	4.503 FIX	3.292	1 FIX	successful	3	-			
16	Cmax/MIC	8	0.849	47.4	0.9281	4.503 FIX	3.226	0.6483	successful	3	-			
17	T>MIC	1	0.890	40.0	7.6680	4.503 FIX	2.77 FIX	1 FIX	successful	3	-			
18	T>MIC	2	0.890	41.9	7.8290	4.503 FIX	2.77 FIX	1.04	successful	3	-			
19	T>MIC	3	0.901	39.6	11.2400	5.078	2.77 FIX	1 FIX	successful	3	T>MIC	14	32	110
20	T>MIC	4	0.905	40.0	17.5300	6.015	2.77 FIX	0.7541	successful	3	-			
21	T>MIC	5	0.903	40.3	14.5400	5.018	2.49	1 FIX	successful	3	-			
22	T>MIC	6	0.905	42.0	16.9900	5.749	2.674	0.8033	successful	3	-			
23	T>MIC	7	0.902	39.8	11.5900	4.503 FIX	2.42	1 FIX	successful	3	-			
24	T>MIC	8	0.901	41.0	13.2800	4.503 FIX	2.345	1.168	successful	3	-			

Takata et al. (2004) PMID:15160299 Drug:Biapenem

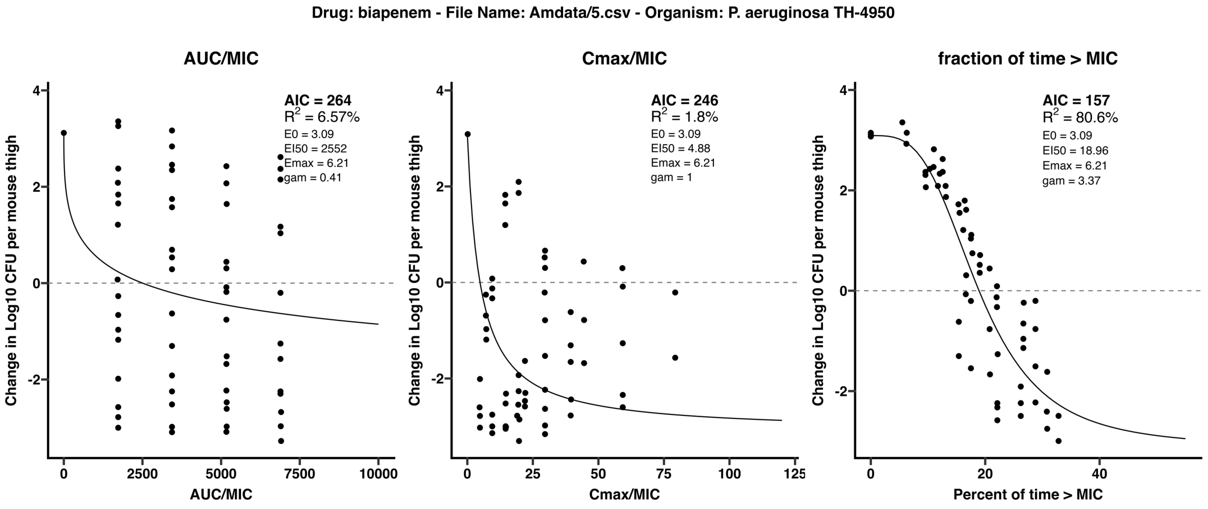

#	Model	Model Number	R²	AIC	EC50 hat	Emax hat	E0 hat	Gamma hat	Status	Best Fit Model	Optimal PKPD Index	Target for Stasis	Log1 Kill	Log2 Kill
1	AUC/MIC	1	0.0548	265	3,153.0000	6.206 FIX	3.091 FIX	1 FIX	successful	2	-			
2	AUC/MIC	2	0.0657	264	2,552.0000	6.206 FIX	3.091 FIX	0.4069	successful	2	-			
3	AUC/MIC	3	0.0643	265	757.7000	4.078	3.091 FIX	1 FIX	successful	2	-			
4	AUC/MIC	4							unsuccessful	2	-			
5	AUC/MIC	5	0.0643	267	765.2000	4.056	3.068	1 FIX	successful	2	-			
6	AUC/MIC	6							unsuccessful	2	-			
7	AUC/MIC	7	0.0456	266	17,130.0000	6.206 FIX	0.9684	1 FIX	successful	2	-			
8	AUC/MIC	8							unsuccessful	2	-			
9	Cmax/MIC	1	0.0180	246	4.8750	6.206 FIX	3.091 FIX	1 FIX	successful	1	-			
10	Cmax/MIC	2							unsuccessful	1	-			
11	Cmax/MIC	3	0.1110	228	0.3474	4.619	3.091 FIX	1 FIX	successful	1	-			
12	Cmax/MIC	4	0.0053	237	150.3000	4.515	3.091 FIX	-3.047	successful	1	-			
13	Cmax/MIC	5	0.1510	227	-0.9683	3.629	2.444	1 FIX	successful	1	-			
14	Cmax/MIC	6							unsuccessful	1	-			
15	Cmax/MIC	7							unsuccessful	1	-			
16	Cmax/MIC	8							unsuccessful	1	-			
17	T>MIC	1	0.6550	208	19.5200	6.206 FIX	3.091 FIX	1 FIX	successful	2	-			
18	T>MIC	2	0.8060	157	18.9600	6.206 FIX	3.091 FIX	3.366	successful	2	T>MIC	19	23	30
19	T>MIC	3							unsuccessful	2	-			
20	T>MIC	4	0.8080	158	17.6000	5.565	3.091 FIX	4.009	successful	2	-			
21	T>MIC	5							unsuccessful	2	-			
22	T>MIC	6	0.8080	160	17.5900	5.636	3.129	3.929	successful	2	-			
23	T>MIC	7	0.6390	210	17.3200	6.206 FIX	3.292	1 FIX	successful	2	-			
24	T>MIC	8	0.8070	159	18.2200	6.206 FIX	3.26	3.369	successful	2	-			

Takata et al. (2004) PMID:15160299 Drug:Imipenem/Cilastatin

Drug: imipenem/cilastatin - File Name: Amdata/5.csv - Organism: P. aeruginosa TH-4950

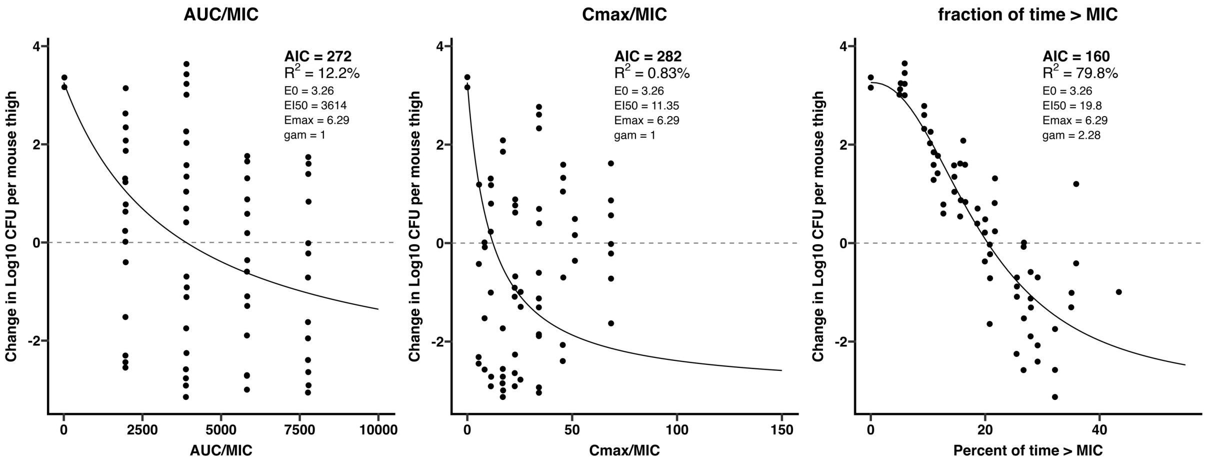

#	Model	Model Number	R²	AIC	EC50 hat	Emax hat	E0 hat	Gamma hat	Status	Best Fit Model	Optimal PKPD Index	Target for Stasis	Log1 Kill	Log2 Kill
1	AUC/MIC	1	0.1220	272	3,614.0000	6.291 FIX	3.261 FIX	1 FIX	successful	1	-			
2	AUC/MIC	2	0.1300	273	3,317.0000	6.291 FIX	3.261 FIX	0.5989	successful	1	-			
3	AUC/MIC	3	0.1280	273	1,465.0000	4.604	3.261 FIX	1 FIX	successful	1	-			
4	AUC/MIC	4	0.1310	275	8,287.0000	8.057	3.261 FIX	0.4983	successful	1	-			
5	AUC/MIC	5	0.1280	275	1,504.0000	4.566	3.21	1 FIX	successful	1	-			
6	AUC/MIC	6							unsuccessful	1	-			
7	AUC/MIC	7	0.1130	274	8,595.0000	6.291 FIX	2.049	1 FIX	successful	1	-			
8	AUC/MIC	8	0.1300	275	2,601.0000	6.291 FIX	3.476	0.5905	successful	1	-			
9	Cmax/MIC	1	0.0083	282	11.3500	6.291 FIX	3.261 FIX	1 FIX	successful	1	-			
10	Cmax/MIC	2							unsuccessful	1	-			
11	Cmax/MIC	3	0.1620	253	-1.0860	3.668	3.261 FIX	1 FIX	successful	1	-			
12	Cmax/MIC	4							unsuccessful	1	-			
13	Cmax/MIC	5	0.1620	255	-1.0890	3.652	3.246	1 FIX	successful	1	-			
14	Cmax/MIC	6							unsuccessful	1	-			
15	Cmax/MIC	7	0.1580	259	-0.6084	6.291 FIX	5.781	1 FIX	successful	1	-			
16	Cmax/MIC	8							unsuccessful	1	-			
17	T>MIC	1	0.7460	197	20.8400	6.291 FIX	3.261 FIX	1 FIX	successful	2	-			
18	T>MIC	2	0.7980	160	19.8000	6.291 FIX	3.261 FIX	2.284	successful	2	T>MIC	20	27	40
19	T>MIC	3	0.7530	177	196.4000	32.5	3.261 FIX	1 FIX	successful	2	-			
20	T>MIC	4							unsuccessful	2	-			
21	T>MIC	5	0.7680	171	49.8700	14.54	4.342	1 FIX	successful	2	-			
22	T>MIC	6							unsuccessful	2	-			
23	T>MIC	7	0.7270	198	16.1700	6.291 FIX	3.678	1 FIX	successful	2	-			
24	T>MIC	8	0.8020	161	17.5800	6.291 FIX	3.612	2.239	successful	2	-			

Takata et al. (2004) PMID:15160299 Drug:Meropenem/Cilastatin

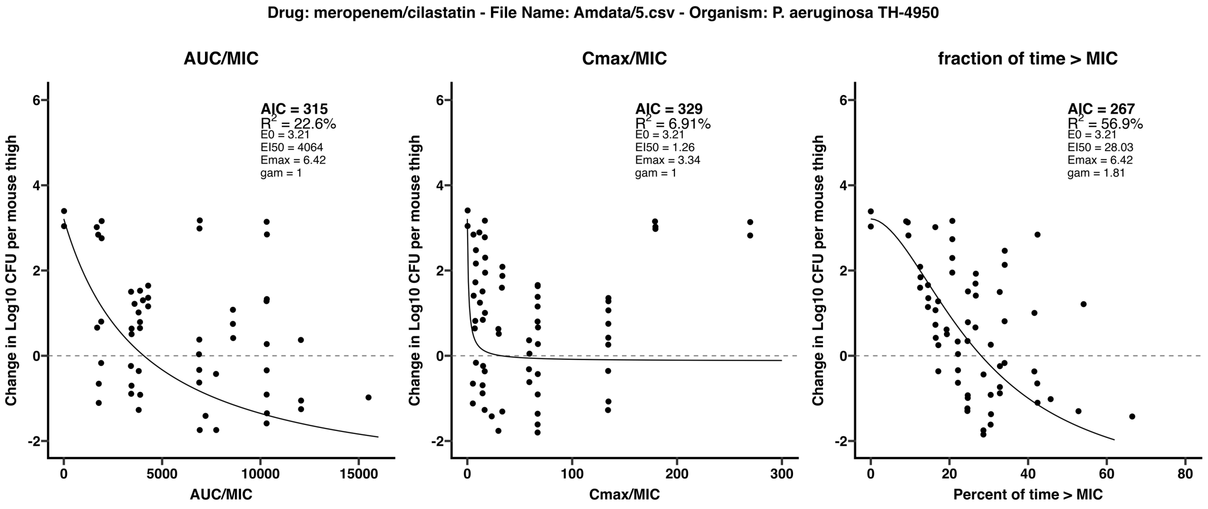

#	Model	Model Number	R²	AIC	EC50 hat	Emax hat	E0 hat	Gamma hat	Status	Best Fit Model	Optimal PKPD Index	Target for Stasis	Log1 Kill	Log2 Kill
1	AUC/MIC	1	0.22600	315	4,064.0000	6.416 FIX	3.21 FIX	1 FIX	successful	1	-			
2	AUC/MIC	2	0.22900	316	3,763.0000	6.416 FIX	3.21 FIX	0.7875	successful	1	-			
3	AUC/MIC	3	0.23100	316	2,590.0000	5.49	3.21 FIX	1 FIX	successful	1	-			
4	AUC/MIC	4	0.23300	317	2,144.0000	4.75	3.21 FIX	1.522	successful	1	-			
5	AUC/MIC	5	0.23100	318	2,405.0000	5.625	3.384	1 FIX	successful	1	-			
6	AUC/MIC	6	0.23300	319	2,116.0000	4.81	3.263	1.507	successful	1	-			
7	AUC/MIC	7	0.23100	316	2,206.0000	6.416 FIX	4.025	1 FIX	successful	1	-			
8	AUC/MIC	8	0.23000	318	2,914.0000	6.416 FIX	3.528	0.8167	successful	1	-			
9	Cmax/MIC	1	0.00110	368	29.1300	6.416 FIX	3.21 FIX	1 FIX	successful	3	-			
10	Cmax/MIC	2	0.00626	334	88.6000	6.416 FIX	3.21 FIX	0.07315	successful	3	-			
11	Cmax/MIC	3	0.06910	329	1.2650	3.335	3.21 FIX	1 FIX	successful	3	-			
12	Cmax/MIC	4							unsuccessful	3	-			
13	Cmax/MIC	5	0.06370	332	-0.4483	1.063	1.139	1 FIX	successful	3	-			
14	Cmax/MIC	6	0.08070	332	4.0900	3.247	3.16	2.428	successful	3	-			
15	Cmax/MIC	7							unsuccessful	3	-			
16	Cmax/MIC	8							unsuccessful	3	-			
17	T>MIC	1	0.55200	278	27.2300	6.416 FIX	3.21 FIX	1 FIX	successful	2	-			
18	T>MIC	2	0.56900	267	28.0300	6.416 FIX	3.21 FIX	1.81	successful	2	T>MIC	28	40	63
19	T>MIC	3	0.58400	264	133.0000	17.36	3.21 FIX	1 FIX	successful	2	-			
20	T>MIC	4	0.58400	266	115.2000	15.94	3.21 FIX	1.027	successful	2	-			
21	T>MIC	5	0.58400	266	108.4000	15.62	3.384	1 FIX	successful	2	-			
22	T>MIC	6	0.58400	268	140.3000	18.13	3.426	0.9452	successful	2	-			
23	T>MIC	7							unsuccessful	2	-			
24	T>MIC	8	0.57300	269	33.6900	6.416 FIX	2.747	1.852	successful	2	-			

Takata et al. (2004) PMID:15160299 Drug:Ceftazidime

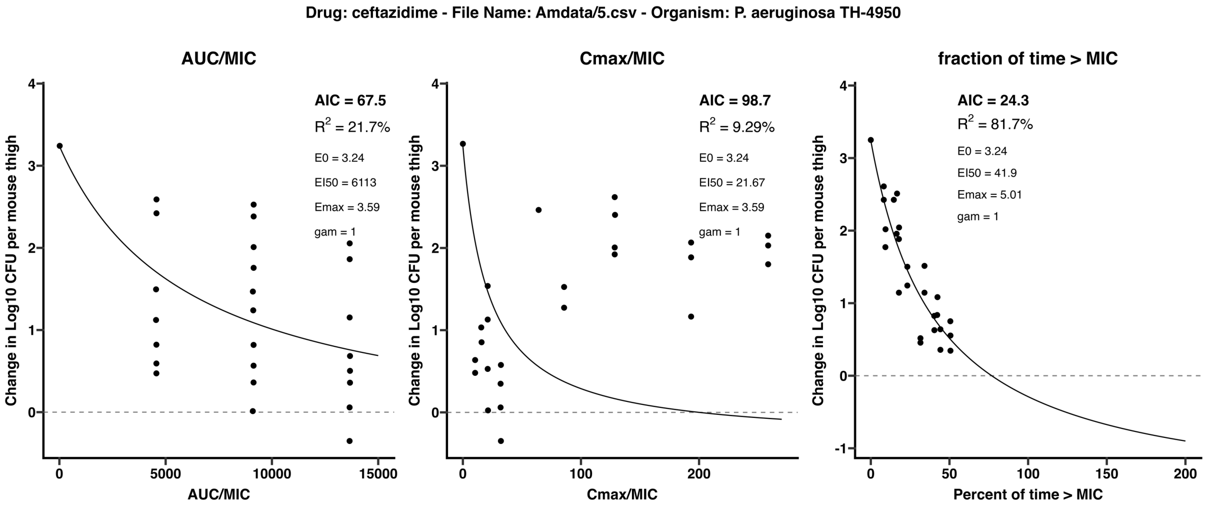

#	Model	Model Number	R²	AIC	EC50 hat	Emax hat	E0 hat	Gamma hat	Status	Best Fit Model	Optimal PKPD Index	Target for Stasis	Log1 Kill	Log2 Kill
1	AUC/MIC	1	0.2170	67.5	6,113.000	3.591 FIX	3.24 FIX	1 FIX	successful	1	-			
2	AUC/MIC	2	0.2350	68.3	4,820.000	3.591 FIX	3.24 FIX	0.5617	successful	1	-			
3	AUC/MIC	3	0.2310	68.5	2,264.000	2.659	3.24 FIX	1 FIX	successful	1	-			
4	AUC/MIC	4	0.2360	70.3	10,420.000	4.36	3.24 FIX	0.4801	successful	1	-			
5	AUC/MIC	5	0.2310	70.5	2,277.000	2.653	3.233	1 FIX	successful	1	-			
6	AUC/MIC	6							unsuccessful	1	-			
7	AUC/MIC	7	0.2280	69.4	3,376.000	3.591 FIX	3.69	1 FIX	successful	1	-			
8	AUC/MIC	8							unsuccessful	1	-			
9	Cmax/MIC	1	0.0929	98.7	21.670	3.591 FIX	3.24 FIX	1 FIX	successful	1	-			
10	Cmax/MIC	2	0.1510	74.4	77.010	3.591 FIX	3.24 FIX	-0.6909	successful	1	-			
11	Cmax/MIC	3	0.4130	60.3	-4.761	1.642	3.24 FIX	1 FIX	successful	1	-			
12	Cmax/MIC	4	0.2820	67.5	195.900	2.412	3.24 FIX	-1.427	successful	1	-			
13	Cmax/MIC	5	0.4150	62.1	-4.450	1.901	3.516	1 FIX	successful	1	-			
14	Cmax/MIC	6							unsuccessful	1	-			
15	Cmax/MIC	7							unsuccessful	1	-			
16	Cmax/MIC	8							unsuccessful	1	-			
17	T>MIC	1	0.8030	26.3	21.690	3.591 FIX	3.24 FIX	1 FIX	successful	3	-			
18	T>MIC	2	0.8140	24.8	22.480	3.591 FIX	3.24 FIX	1.316	successful	3	-			
19	T>MIC	3	0.8170	24.3	41.900	5.007	3.24 FIX	1 FIX	successful	3	T>MIC	77	200	200
20	T>MIC	4	0.8170	26.3	45.870	5.237	3.24 FIX	0.9721	successful	3	-			
21	T>MIC	5	0.8170	26.3	44.090	5.071	3.207	1 FIX	successful	3	-			
22	T>MIC	6	0.8170	28.3	43.310	5.022	3.205	1.007	successful	3	-			
23	T>MIC	7	0.8050	28.2	23.060	3.591 FIX	3.187	1 FIX	successful	3	-			
24	T>MIC	8	0.8150	26.5	25.850	3.591 FIX	3.094	1.338	successful	3	-			

Nakamura et al. (2019) PMID:31262762 Drug:Cefiderocol

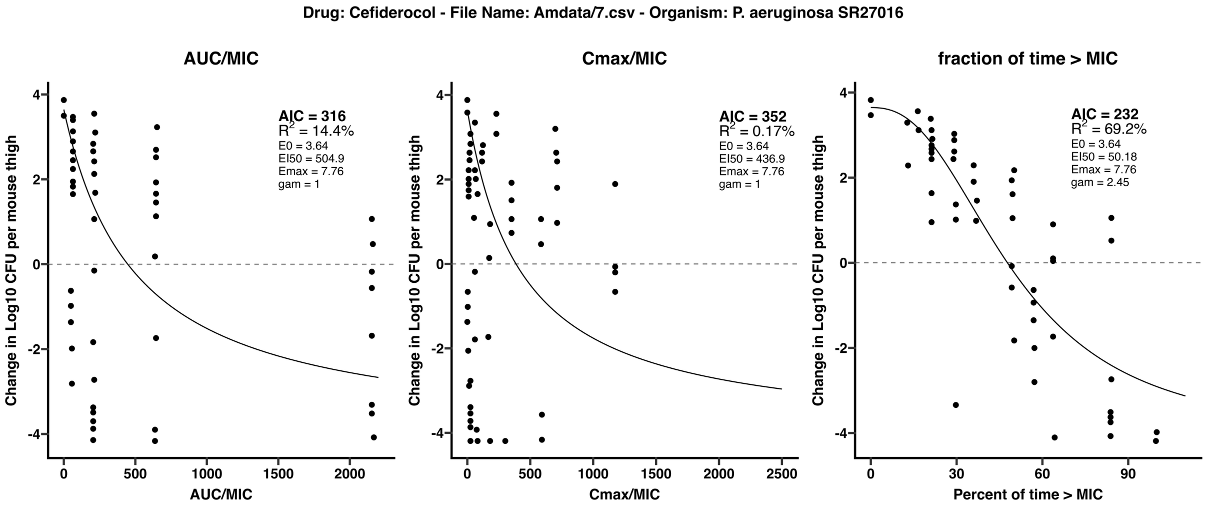

#	Model	Model Number	R²	AIC	EC50 hat	Emax hat	E0 hat	Gamma hat	Status	Best Fit Model	Optimal PKPD Index	Target for Stasis	Log1 Kill	Log2 Kill
1	AUC/MIC	1	0.14400	316	504.9000	7.762 FIX	3.645 FIX	1 FIX	successful	1	-			
2	AUC/MIC	2							unsuccessful	1	-			
3	AUC/MIC	3	0.13200	308	73.2300	4.342	3.645 FIX	1 FIX	successful	1	-			
4	AUC/MIC	4							unsuccessful	1	-			
5	AUC/MIC	5	0.15000	308	3,304.0000	7.681	1.491	1 FIX	successful	1	-			
6	AUC/MIC	6							unsuccessful	1	-			
7	AUC/MIC	7	0.11500	313	27.3300	7.762 FIX	7.028	1 FIX	successful	1	-			
8	AUC/MIC	8							unsuccessful	1	-			
9	Cmax/MIC	1	0.00172	352	436.9000	7.762 FIX	3.645 FIX	1 FIX	successful	1	-			
10	Cmax/MIC	2							unsuccessful	1	-			
11	Cmax/MIC	3	0.02250	315	-1.0170	3.064	3.645 FIX	1 FIX	successful	1	-			
12	Cmax/MIC	4							unsuccessful	1	-			
13	Cmax/MIC	5	0.00198	318	198.0000	-0.3928	0.3874	1 FIX	successful	1	-			
14	Cmax/MIC	6							unsuccessful	1	-			
15	Cmax/MIC	7	0.01740	330	-0.1038	7.762 FIX	7.965	1 FIX	successful	1	-			
16	Cmax/MIC	8							unsuccessful	1	-			
17	T>MIC	1	0.64000	259	58.3400	7.762 FIX	3.645 FIX	1 FIX	successful	2	-			
18	T>MIC	2	0.69200	232	50.1800	7.762 FIX	3.645 FIX	2.454	successful	2	T>MIC	48	59	75
19	T>MIC	3							unsuccessful	2	-			
20	T>MIC	4	0.70100	232	87.9800	13.27	3.645 FIX	1.727	successful	2	-			
21	T>MIC	5							unsuccessful	2	-			
22	T>MIC	6							unsuccessful	2	-			
23	T>MIC	7	0.62000	260	45.7500	7.762 FIX	4.138	1 FIX	successful	2	-			
24	T>MIC	8	0.69800	233	56.8000	7.762 FIX	3.139	2.722	successful	2	-			

Nakamura et al. (2019) PMID:31262762 Drug:Cefepime

Drug: Cefepime - File Name: Amdata/7.csv - Organism: P. aeruginosa SR27016

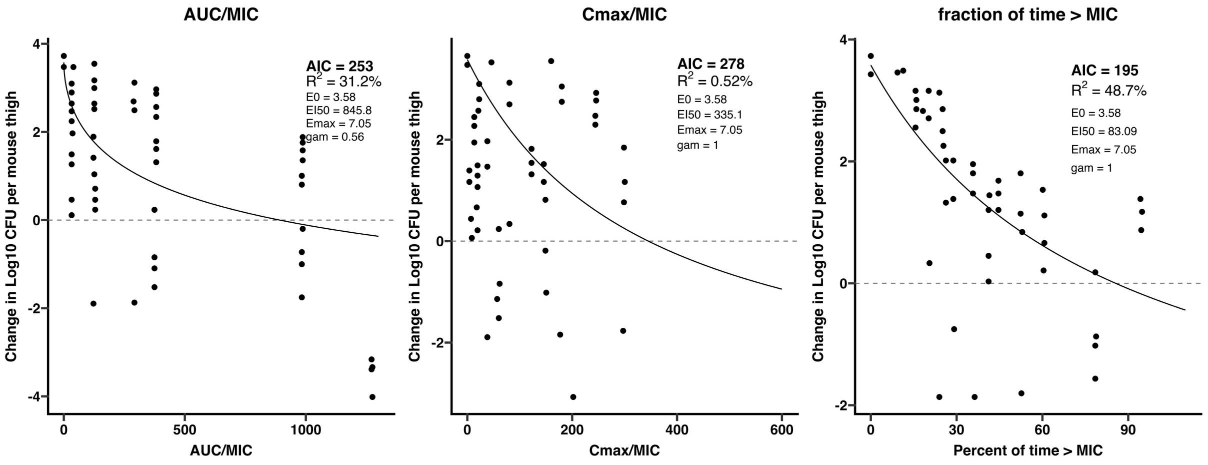

#	Model	Model Number	R²	AIC	EC50 hat	Emax hat	E0 hat	Gamma hat	Status	Best Fit Model	Optimal PKPD Index	Target for Stasis	Log1 Kill	Log2 Kill
1	AUC/MIC	1	0.32000	257	666.9000	7.053 FIX	3.579 FIX	1 FIX	successful	2	-			
2	AUC/MIC	2	0.31200	253	845.8000	7.053 FIX	3.579 FIX	0.5569	successful	2	-			
3	AUC/MIC	3	0.28500	257	208.9000	4.497	3.579 FIX	1 FIX	successful	2	-			
4	AUC/MIC	4							unsuccessful	2	-			
5	AUC/MIC	5							unsuccessful	2	-			
6	AUC/MIC	6							unsuccessful	2	-			
7	AUC/MIC	7	0.33600	250	1,566.0000	7.053 FIX	2.464	1 FIX	successful	2	-			
8	AUC/MIC	8	0.46000	239	1,151.0000	7.053 FIX	1.8	10.66	successful	2	-			
9	Cmax/MIC	1	0.00524	278	335.1000	7.053 FIX	3.579 FIX	1 FIX	successful	1	-			
10	Cmax/MIC	2							unsuccessful	1	-			
11	Cmax/MIC	3	0.04910	251	-0.4192	2.018	3.579 FIX	1 FIX	successful	1	-			
12	Cmax/MIC	4							unsuccessful	1	-			
13	Cmax/MIC	5	0.01040	256	-6.6030	0.07855	1.695	1 FIX	successful	1	-			
14	Cmax/MIC	6							unsuccessful	1	-			
15	Cmax/MIC	7	0.00515	254	5,442.0000	7.053 FIX	1.719	1 FIX	successful	1	-			
16	Cmax/MIC	8							unsuccessful	1	-			
17	T>MIC	1	0.48700	195	83.0900	7.053 FIX	3.579 FIX	1 FIX	successful	1	T>MIC	86	110	110
18	T>MIC	2	0.48500	197	77.0100	7.053 FIX	3.579 FIX	1.115	successful	1	-			
19	T>MIC	3	0.48700	197	84.0400	7.105	3.579 FIX	1 FIX	successful	1	-			
20	T>MIC	4							unsuccessful	1	-			
21	T>MIC	5	0.49600	197	45.2200	6.561	4.302	1 FIX	successful	1	-			
22	T>MIC	6							unsuccessful	1	-			
23	T>MIC	7	0.49500	195	53.0200	7.053 FIX	4.26	1 FIX	successful	1	-			
24	T>MIC	8	0.49600	197	53.3300	7.053 FIX	4.241	1.017	successful	1	-			

Takemura et al. (2021) PMID:34853981 Drug:Cefmetazole

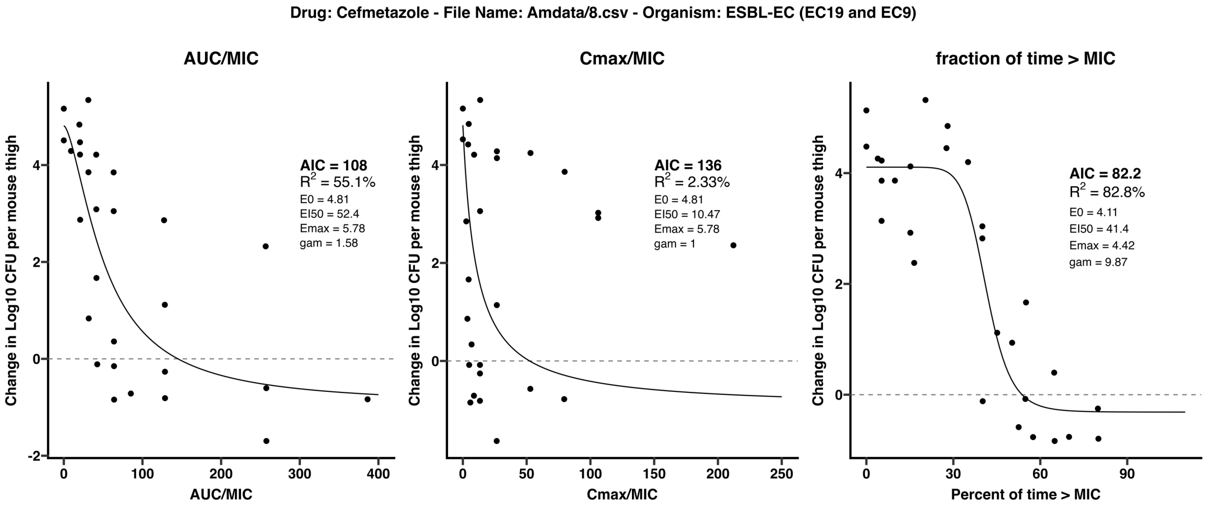

#	Model	Model Number	R²	AIC	EC50 hat	Emax hat	E0 hat	Gamma hat	Status	Best Fit Model	Optimal PKPD Index	Target for Stasis	Log1 Kill	Log2 Kill
1	AUC/MIC	1	0.5350	108.0	53.9500	5.775 FIX	4.812 FIX	1 FIX	successful	2	-			
2	AUC/MIC	2	0.5510	108.0	52.4000	5.775 FIX	4.812 FIX	1.58	successful	2	-			
3	AUC/MIC	3	0.5320	109.0	72.0100	6.626	4.812 FIX	1 FIX	successful	2	-			
4	AUC/MIC	4	0.5660	109.0	40.6700	4.813	4.812 FIX	2.604	successful	2	-			
5	AUC/MIC	5	0.5350	111.0	56.0300	6.955	5.353	1 FIX	successful	2	-			
6	AUC/MIC	6	0.5660	111.0	41.1500	4.718	4.735	2.699	successful	2	-			
7	AUC/MIC	7							unsuccessful	2	-			
8	AUC/MIC	8	0.5600	109.0	41.4000	5.775 FIX	5.267	1.776	successful	2	-			
9	Cmax/MIC	1	0.0233	136.0	10.4700	5.775 FIX	4.812 FIX	1 FIX	successful	1	-			
10	Cmax/MIC	2							unsuccessful	1	-			
11	Cmax/MIC	3	0.1220	126.0	0.1578	3.023	4.812 FIX	1 FIX	successful	1	-			
12	Cmax/MIC	4							unsuccessful	1	-			
13	Cmax/MIC	5	0.1220	128.0	0.1567	3.05	4.838	1 FIX	successful	1	-			
14	Cmax/MIC	6							unsuccessful	1	-			
15	Cmax/MIC	7							unsuccessful	1	-			
16	Cmax/MIC	8							unsuccessful	1	-			
17	T>MIC	1	0.6080	101.0	33.3700	5.775 FIX	4.812 FIX	1 FIX	successful	6	-			
18	T>MIC	2	0.8160	86.0	41.9400	5.775 FIX	4.812 FIX	5.625	successful	6	-			
19	T>MIC	3							unsuccessful	6	-			
20	T>MIC	4	0.8230	87.4	40.1700	5.221	4.812 FIX	7.999	successful	6	-			
21	T>MIC	5							unsuccessful	6	-			
22	T>MIC	6	0.8280	82.2	41.4000	4.421	4.107	9.866	successful	6	T>MIC	54	110	110
23	T>MIC	7	0.6200	103.0	37.7800	5.775 FIX	4.627	1 FIX	successful	6	-			
24	T>MIC	8	0.8100	83.6	46.3500	5.775 FIX	4.241	4.907	successful	6	-			

Guo et al. (2016) PMID:26666923 Drug:Cefquinome

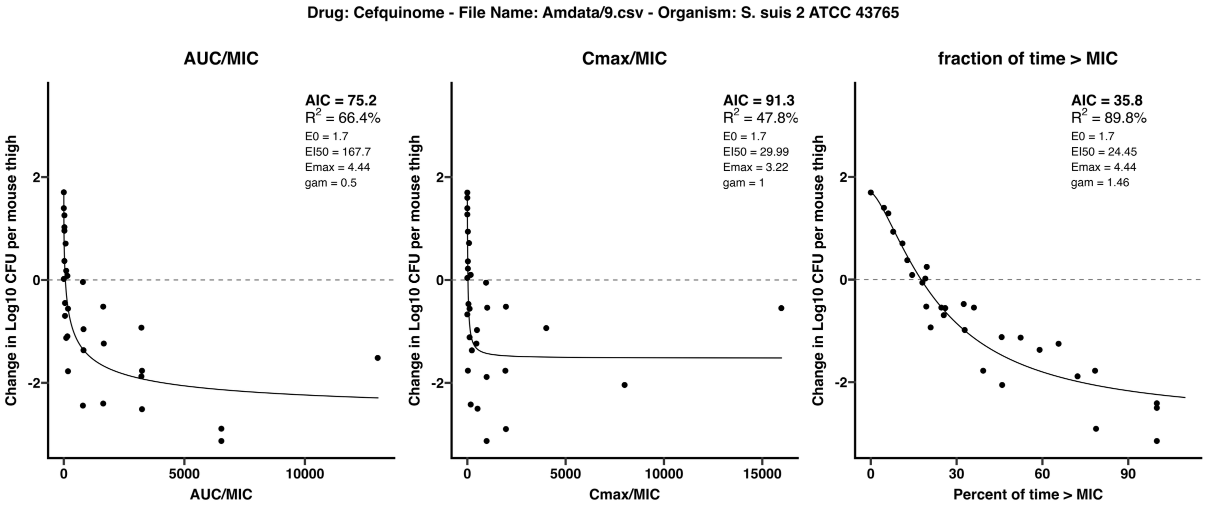

#	Model	Model Number	R²	AIC	EC50 hat	Emax hat	E0 hat	Gamma hat	Status	Best Fit Model	Optimal PKPD Index	Target for Stasis	Log1 Kill	Log2 Kill
1	AUC/MIC	1	0.667	84.2	147.20	4.443 FIX	1.697 FIX	1 FIX	successful	2	-			
2	AUC/MIC	2	0.664	75.2	167.70	4.443 FIX	1.697 FIX	0.5005	successful	2	-			
3	AUC/MIC	3	0.660	75.7	68.66	3.551	1.697 FIX	1 FIX	successful	2	-			
4	AUC/MIC	4	0.665	77.0	105.50	4.011	1.697 FIX	0.6182	successful	2	-			
5	AUC/MIC	5	0.670	75.7	111.30	3.05	1.13	1 FIX	successful	2	-			
6	AUC/MIC	6	0.674	77.3	167.00	3.449	1.17	0.6946	successful	2	-			
7	AUC/MIC	7	0.656	82.9	63.88	4.443 FIX	2.256	1 FIX	successful	2	-			
8	AUC/MIC	8							unsuccessful	2	-			
9	Cmax/MIC	1	0.474	100.0	111.20	4.443 FIX	1.697 FIX	1 FIX	successful	3	-			
10	Cmax/MIC	2	0.449	92.9	69.52	4.443 FIX	1.697 FIX	0.2924	successful	3	-			
11	Cmax/MIC	3	0.478	91.3	29.99	3.22	1.697 FIX	1 FIX	successful	3	-			
12	Cmax/MIC	4							unsuccessful	3	-			
13	Cmax/MIC	5	0.490	88.9	55.61	2.455	0.8817	1 FIX	successful	3	-			
14	Cmax/MIC	6	0.490	90.9	56.76	2.404	0.8635	1.125	successful	3	-			
15	Cmax/MIC	7	0.490	102.0	57.43	4.443 FIX	2.095	1 FIX	successful	3	-			
16	Cmax/MIC	8							unsuccessful	3	-			
17	T>MIC	1	0.892	45.2	22.91	4.443 FIX	1.697 FIX	1 FIX	successful	2	-			
18	T>MIC	2	0.898	35.8	24.45	4.443 FIX	1.697 FIX	1.457	successful	2	T>MIC	18	33	73
19	T>MIC	3	0.907	33.1	47.02	6.218	1.697 FIX	1 FIX	successful	2	-			
20	T>MIC	4	0.907	35.1	42.92	5.96	1.697 FIX	1.04	successful	2	-			
21	T>MIC	5	0.907	34.8	41.17	6.145	1.846	1 FIX	successful	2	-			
22	T>MIC	6	0.907	36.8	44.84	6.442	1.874	0.954	successful	2	-			
23	T>MIC	7	0.900	46.6	27.00	4.443 FIX	1.522	1 FIX	successful	2	-			
24	T>MIC	8	0.900	37.3	27.60	4.443 FIX	1.537	1.447	successful	2	-			

Shan et al. (2014) PMID:25070101 Drug:Cefquinome

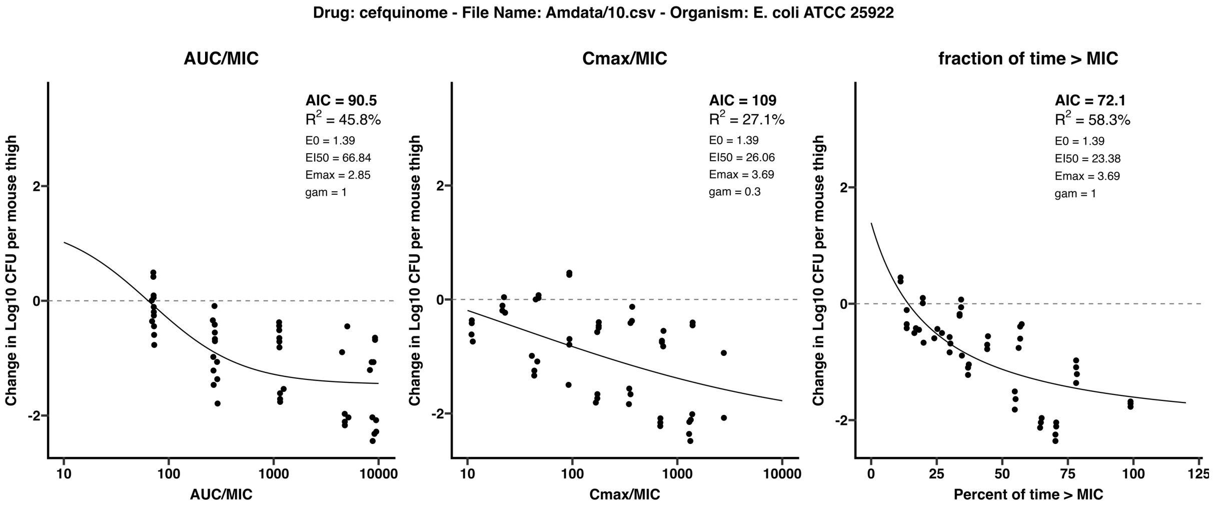

#	Model	Model Number	R²	AIC	EC50 hat	Emax hat	E0 hat	Gamma hat	Status	Best Fit Model	Optimal PKPD Index	Target for Stasis	Log1 Kill	Log2 Kill
1	AUC/MIC	1	0.467	120.0	174.400	3.694 FIX	1.39 FIX	1 FIX	successful	3	-			
2	AUC/MIC	2	0.474	89.0	136.700	3.694 FIX	1.39 FIX	0.3649	successful	3	-			
3	AUC/MIC	3	0.458	90.5	66.840	2.849	1.39 FIX	1 FIX	successful	3	-			
4	AUC/MIC	4	0.478	90.7	75.580	3.206	1.39 FIX	0.528	successful	3	-			
5	AUC/MIC	5	0.467	91.7	196.300	1.891	0.3394	1 FIX	successful	3	-			
6	AUC/MIC	6							unsuccessful	3	-			
7	AUC/MIC	7	0.452	91.1	41.510	3.694 FIX	2.261	1 FIX	successful	3	-			
8	AUC/MIC	8							unsuccessful	3	-			
9	Cmax/MIC	1	0.249	140.0	58.010	3.694 FIX	1.39 FIX	1 FIX	successful	2	-			
10	Cmax/MIC	2	0.271	109.0	26.060	3.694 FIX	1.39 FIX	0.3012	successful	2	-			
11	Cmax/MIC	3	0.168	116.0	9.374	2.637	1.39 FIX	1 FIX	successful	2	-			
12	Cmax/MIC	4							unsuccessful	2	-			
13	Cmax/MIC	5	0.297	109.0	376.200	1.534	-0.3643	1 FIX	successful	2	-			
14	Cmax/MIC	6	0.298	111.0	329.400	1.347	-0.4144	1.251	successful	2	-			
15	Cmax/MIC	7	0.149	117.0	4.738	3.694 FIX	2.489	1 FIX	successful	2	-			
16	Cmax/MIC	8							unsuccessful	2	-			
17	T>MIC	1	0.583	72.1	23.380	3.694 FIX	1.39 FIX	1 FIX	successful	1	T>MIC	14	43	120
18	T>MIC	2	0.582	73.8	24.080	3.694 FIX	1.39 FIX	1.083	successful	1	-			
19	T>MIC	3	0.590	72.9	30.590	4.108	1.39 FIX	1 FIX	successful	1	-			
20	T>MIC	4							unsuccessful	1	-			
21	T>MIC	5	0.605	72.9	98.090	4.919	0.5717	1 FIX	successful	1	-			
22	T>MIC	6	0.615	73.7	47.300	1.787	-0.1379	3.416	successful	1	-			
23	T>MIC	7	0.600	72.1	49.320	3.694 FIX	0.7521	1 FIX	successful	1	-			
24	T>MIC	8	0.607	72.7	72.210	3.694 FIX	0.3179	1.328	successful	1	-			

Shan and Wang (2017) PMID:27682189 Drug:Cefquinome

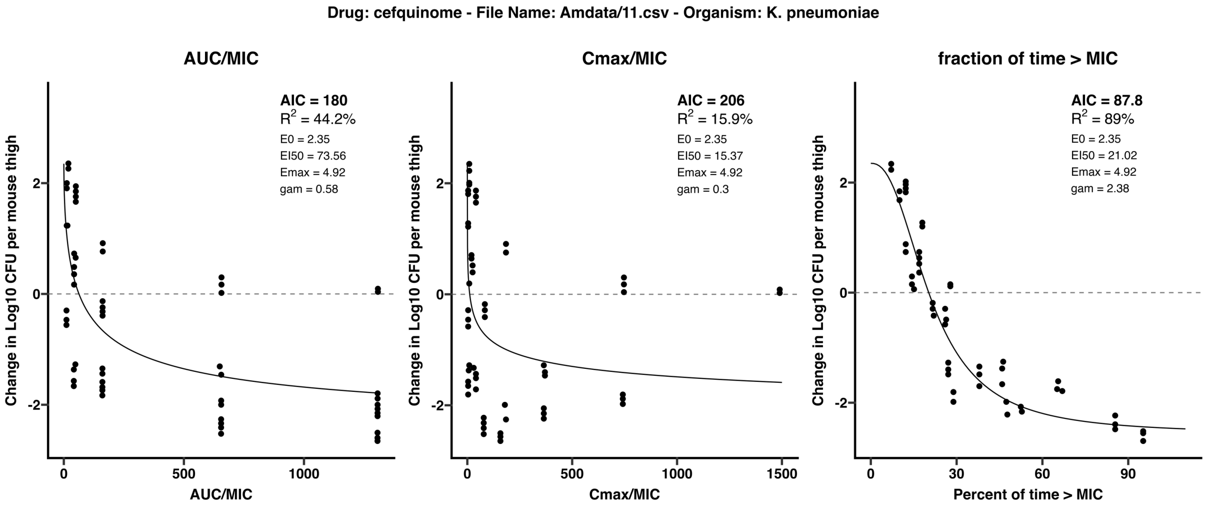

#	Model	Model Number	R²	AIC	EC50 hat	Emax hat	E0 hat	Gamma hat	Status	Best Fit Model	Optimal PKPD Index	Target for Stasis	Log1 Kill	Log2 Kill
1	AUC/MIC	1	0.446	186.0	80.510	4.92 FIX	2.35 FIX	1 FIX	successful	2	-			
2	AUC/MIC	2	0.442	180.0	73.560	4.92 FIX	2.35 FIX	0.5792	successful	2	-			
3	AUC/MIC	3	0.430	181.0	42.830	4.105	2.35 FIX	1 FIX	successful	2	-			
4	AUC/MIC	4	0.442	182.0	76.210	4.963	2.35 FIX	0.5706	successful	2	-			
5	AUC/MIC	5	0.447	181.0	104.200	3.305	1.313	1 FIX	successful	2	-			
6	AUC/MIC	6	0.449	183.0	106.100	2.801	1.035	1.42	successful	2	-			
7	AUC/MIC	7	0.409	184.0	27.290	4.92 FIX	3.184	1 FIX	successful	2	-			
8	AUC/MIC	8	0.442	181.0	96.540	4.92 FIX	2.184	0.5671	successful	2	-			
9	Cmax/MIC	1	0.195	221.0	25.090	4.92 FIX	2.35 FIX	1 FIX	successful	2	-			
10	Cmax/MIC	2	0.159	206.0	15.370	4.92 FIX	2.35 FIX	0.3031	successful	2	-			
11	Cmax/MIC	3	0.139	207.0	4.496	3.456	2.35 FIX	1 FIX	successful	2	-			
12	Cmax/MIC	4	0.167	207.0	5.281	3.962	2.35 FIX	0.4955	successful	2	-			
13	Cmax/MIC	5	0.198	205.0	37.210	2.1	0.6347	1 FIX	successful	2	-			
14	Cmax/MIC	6							unsuccessful	2	-			
15	Cmax/MIC	7	0.117	208.0	1.895	4.92 FIX	3.914	1 FIX	successful	2	-			
16	Cmax/MIC	8							unsuccessful	2	-			
17	T>MIC	1	0.873	143.0	18.860	4.92 FIX	2.35 FIX	1 FIX	successful	2	-			
18	T>MIC	2	0.890	87.8	21.020	4.92 FIX	2.35 FIX	2.377	successful	2	T>MIC	20	29	49
19	T>MIC	3	0.825	118.0	46.830	7.763	2.35 FIX	1 FIX	successful	2	-			
20	T>MIC	4	0.892	88.7	20.060	4.712	2.35 FIX	2.62	successful	2	-			
21	T>MIC	5	0.885	92.1	9.035	11.14	7.601	1 FIX	successful	2	-			
22	T>MIC	6	0.894	89.8	18.160	5.471	2.978	2.155	successful	2	-			
23	T>MIC	7							unsuccessful	2	-			
24	T>MIC	8	0.893	88.2	19.300	4.92 FIX	2.549	2.486	successful	2	-			

Wang et al. (2014) PMID:24614373 Drug:Cefquinome

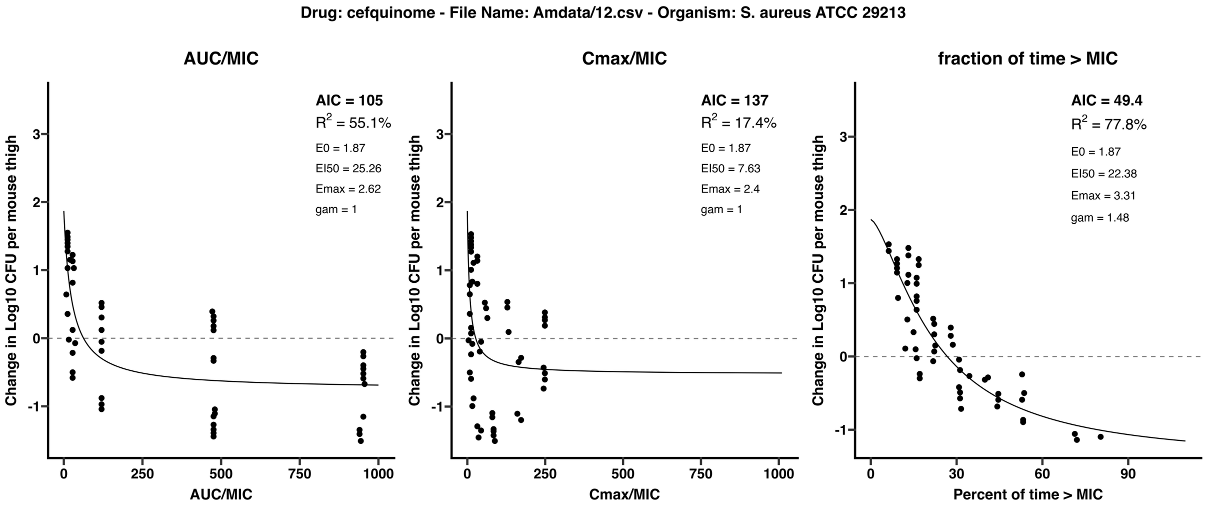

#	Model	Model Number	R²	AIC	EC50 hat	Emax hat	E0 hat	Gamma hat	Status	Best Fit Model	Optimal PKPD Index	Target for Stasis	Log1 Kill	Log2 Kill
1	AUC/MIC	1	0.543	121.0	60.000	3.308 FIX	1.87 FIX	1 FIX	successful	3	-			
2	AUC/MIC	2	0.542	106.0	59.590	3.308 FIX	1.87 FIX	0.5388	successful	3	-			
3	AUC/MIC	3	0.551	105.0	25.260	2.623	1.87 FIX	1 FIX	successful	3	-			
4	AUC/MIC	4	0.552	107.0	26.870	2.681	1.87 FIX	0.8999	successful	3	-			
5	AUC/MIC	5	0.551	107.0	25.060	2.63	1.878	1 FIX	successful	3	-			
6	AUC/MIC	6							unsuccessful	3	-			
7	AUC/MIC	7	0.548	105.0	14.460	3.308 FIX	2.6	1 FIX	successful	3	-			
8	AUC/MIC	8	0.553	107.0	17.190	3.308 FIX	2.443	0.7578	successful	3	-			
9	Cmax/MIC	1	0.179	149.0	22.910	3.308 FIX	1.87 FIX	1 FIX	successful	3	-			
10	Cmax/MIC	2	0.159	138.0	18.670	3.308 FIX	1.87 FIX	0.3878	successful	3	-			
11	Cmax/MIC	3	0.174	137.0	7.626	2.395	1.87 FIX	1 FIX	successful	3	-			
12	Cmax/MIC	4							unsuccessful	3	-			
13	Cmax/MIC	5	0.180	139.0	16.240	1.711	1.122	1 FIX	successful	3	-			
14	Cmax/MIC	6							unsuccessful	3	-			
15	Cmax/MIC	7	0.163	138.0	4.243	3.308 FIX	2.822	1 FIX	successful	3	-			
16	Cmax/MIC	8							unsuccessful	3	-			
17	T>MIC	1	0.779	60.6	22.490	3.308 FIX	1.87 FIX	1 FIX	successful	2	-			
18	T>MIC	2	0.778	49.4	22.380	3.308 FIX	1.87 FIX	1.484	successful	2	T>MIC	27	79	110
19	T>MIC	3	0.772	51.2	44.800	4.799	1.87 FIX	1 FIX	successful	2	-			
20	T>MIC	4	0.778	51.4	23.520	3.413	1.87 FIX	1.426	successful	2	-			
21	T>MIC	5	0.779	51.3	23.660	4.6	2.459	1 FIX	successful	2	-			
22	T>MIC	6	0.779	53.3	23.290	4.08	2.215	1.15	successful	2	-			
23	T>MIC	7	0.779	62.5	23.210	3.308 FIX	1.844	1 FIX	successful	2	-			
24	T>MIC	8	0.778	51.4	22.850	3.308 FIX	1.848	1.483	successful	2	-			

Craig and Andes (2008) PMID:18676887 Drug:Ceftobiprole

Drug: ceftobiprole - File Name: Amdata/13.csv - Organism: S. aureus ATCC 33591

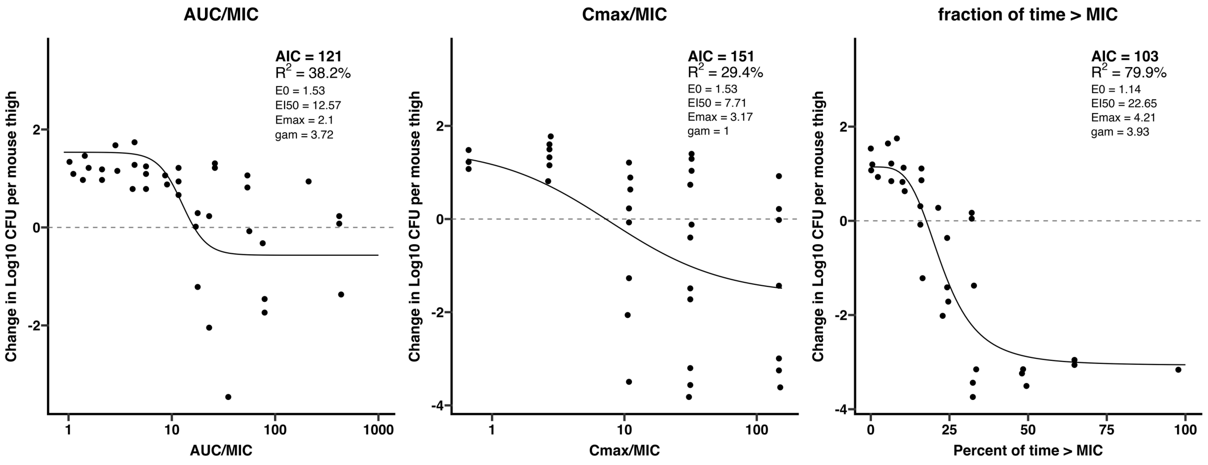

#	Model	Model Number	R²	AIC	EC50 hat	Emax hat	E0 hat	Gamma hat	Status	Best Fit Model	Optimal PKPD Index	Target for Stasis	Log1 Kill	Log2 Kill
1	AUC/MIC	1	0.233	132	48.710	4.212 FIX	1.535 FIX	1 FIX	successful	4	-	-	-	-
2	AUC/MIC	2	0.236	126	134.400	4.212 FIX	1.535 FIX	0.4335	successful	4	-	-	-	-
3	AUC/MIC	3	0.317	122	11.680	2.323	1.535 FIX	1 FIX	successful	4	-	-	-	-
4	AUC/MIC	4	0.382	121	12.570	2.102	1.535 FIX	3.722	successful	4	-	-	-	-
5	AUC/MIC	5							unsuccessful	4	-	-	-	-
6	AUC/MIC	6							unsuccessful	4	-	-	-	-
7	AUC/MIC	7	0.261	125	1.657	4.212 FIX	3.765	1 FIX	successful	4	-	-	-	-
8	AUC/MIC	8							unsuccessful	4	-	-	-	-
9	Cmax/MIC	1	0.268	151	17.300	4.212 FIX	1.535 FIX	1 FIX	successful	3	-	-	-	-
10	Cmax/MIC	2	0.271	152	19.130	4.212 FIX	1.535 FIX	0.6671	successful	3	-	-	-	-
11	Cmax/MIC	3	0.294	151	7.706	3.171	1.535 FIX	1 FIX	successful	3	-	-	-	-
12	Cmax/MIC	4	0.313	152	5.898	2.669	1.535 FIX	2.705	successful	3	-	-	-	-
13	Cmax/MIC	5	0.297	152	5.335	3.606	2.041	1 FIX	successful	3	-	-	-	-
14	Cmax/MIC	6	0.314	153	6.538	2.488	1.372	3.301	successful	3	-	-	-	-
15	Cmax/MIC	7	0.296	151	4.049	4.212 FIX	2.606	1 FIX	successful	3	-	-	-	-
16	Cmax/MIC	8	0.294	153	4.322	4.212 FIX	2.51	0.8534	successful	3	-	-	-	-
17	T>MIC	1	0.644	129	16.390	4.212 FIX	1.535 FIX	1 FIX	successful	8	-	-	-	-
18	T>MIC	2	0.787	105	19.750	4.212 FIX	1.535 FIX	3.8	successful	8	-	-	-	-
19	T>MIC	3	0.726	114	77.750	10.74	1.535 FIX	1 FIX	successful	8	-	-	-	-
20	T>MIC	4	0.799	104	22.570	5.005	1.535 FIX	2.923	successful	8	-	-	-	-
21	T>MIC	5	0.729	115	58.470	10.1	1.926	1 FIX	successful	8	-	-	-	-
22	T>MIC	6	0.801	105	23.240	4.641	1.276	3.364	successful	8	-	-	-	-
23	T>MIC	7	0.673	131	20.540	4.212 FIX	1.266	1 FIX	successful	8	-	-	-	-
24	T>MIC	8	0.799	103	22.650	4.212 FIX	1.144	3.934	successful	8	T>MIC	18	23	30

Craig and Andes (2013) PMID:23274659 Drug:Ceftolozane

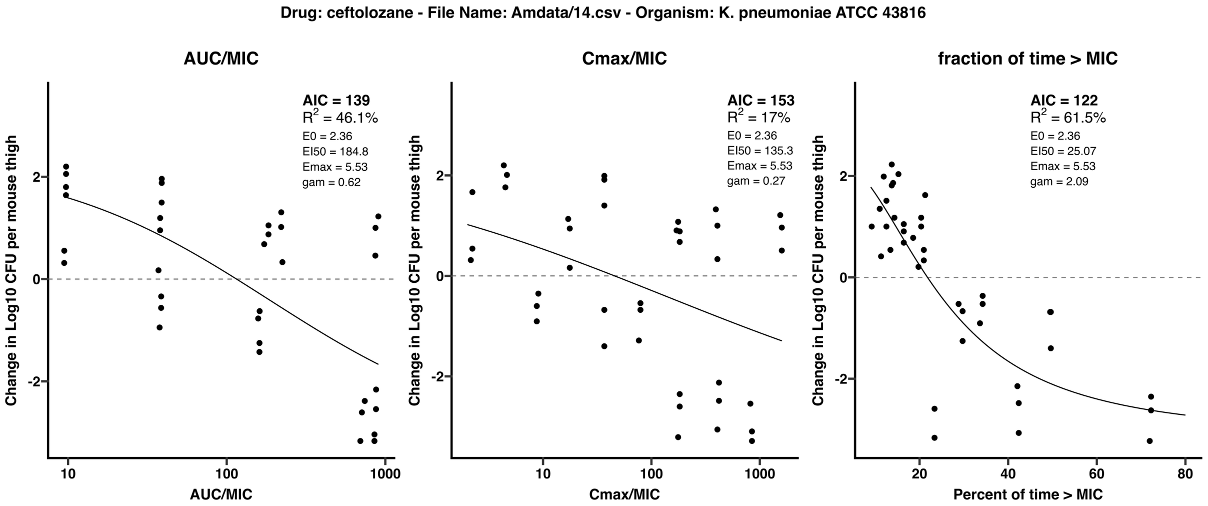

#	Model	Model Number	R²	AIC	EC50 hat	Emax hat	E0 hat	Gamma hat	Status	Best Fit Model	Optimal PKPD Index	Target for Stasis	Log1 Kill	Log2 Kill
1	AUC/MIC	1	0.466	141	203.800	5.525 FIX	2.36 FIX	1 FIX	successful	2	-	-	-	-
2	AUC/MIC	2	0.461	139	184.800	5.525 FIX	2.36 FIX	0.6226	successful	2	-	-	-	-
3	AUC/MIC	3	0.439	141	103.100	4.506	2.36 FIX	1 FIX	successful	2	-	-	-	-
4	AUC/MIC	4							unsuccessful	2	-	-	-	-
5	AUC/MIC	5	0.480	139	548.700	5.102	1.22	1 FIX	successful	2	-	-	-	-
6	AUC/MIC	6							unsuccessful	2	-	-	-	-
7	AUC/MIC	7	0.353	147	20.710	5.525 FIX	4.063	1 FIX	successful	2	-	-	-	-
8	AUC/MIC	8	0.481	139	619.800	5.525 FIX	1.291	0.9055	successful	2	-	-	-	-
9	Cmax/MIC	1	0.176	167	115.900	5.525 FIX	2.36 FIX	1 FIX	successful	2	-	-	-	-
10	Cmax/MIC	2	0.170	153	135.300	5.525 FIX	2.36 FIX	0.2706	successful	2	-	-	-	-
11	Cmax/MIC	3	0.175	153	7.791	3.166	2.36 FIX	1 FIX	successful	2	-	-	-	-
12	Cmax/MIC	4	0.186	154	9.945	3.536	2.36 FIX	0.5895	successful	2	-	-	-	-
13	Cmax/MIC	5	0.193	154	35.090	2.258	1.207	1 FIX	successful	2	-	-	-	-
14	Cmax/MIC	6							unsuccessful	2	-	-	-	-
15	Cmax/MIC	7	0.145	154	1.693	5.525 FIX	4.896	1 FIX	successful	2	-	-	-	-
16	Cmax/MIC	8							unsuccessful	2	-	-	-	-
17	T>MIC	1	0.606	132	27.570	5.525 FIX	2.36 FIX	1 FIX	successful	2	-	-	-	-
18	T>MIC	2	0.615	122	25.070	5.525 FIX	2.36 FIX	2.094	successful	2	T>MIC	22	31	22
19	T>MIC	3	0.581	126	94.860	12.19	2.36 FIX	1 FIX	successful	2	-	-	-	-
20	T>MIC	4	0.623	124	20.800	4.67	2.36 FIX	2.932	successful	2	-	-	-	-
21	T>MIC	5	0.608	125	18.390	10.1	5.42	1 FIX	successful	2	-	-	-	-
22	T>MIC	6							unsuccessful	2	-	-	-	-
23	T>MIC	7							unsuccessful	2	-	-	-	-
24	T>MIC	8	0.618	124	21.520	5.525 FIX	2.723	2.166	successful	2	-	-	-	-

Dudhani et al. (2010) PMID:20028824 Drug:Colistin

Drug: colistin - File Name: Amdata/15.csv - Organism: P. aeruginosa ATCC 27853,

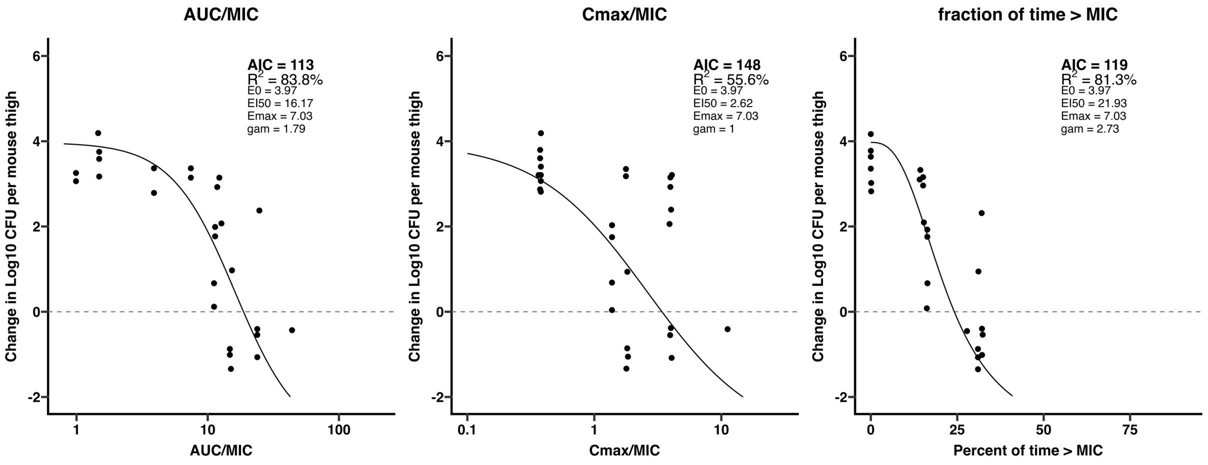

#	Model	Model Number	R²	AIC	EC50 hat	Emax hat	E0 hat	Gamma hat	Status	Best Fit Model	Optimal PKPD Index	Target for Stasis	Log1 Kill	Log2 Kill
1	AUC/MIC	1	0.812	122	15.390	7.027 FIX	3.972 FIX	1 FIX	successful	2	-	-	-	-
2	AUC/MIC	2	0.838	113	16.170	7.027 FIX	3.972 FIX	1.786	successful	2	AUC/MIC	18.7	26.5	42.7
3	AUC/MIC	3	0.820	117	25.560	8.852	3.972 FIX	1 FIX	successful	2	-	-	-	-
4	AUC/MIC	4	0.838	115	16.670	7.169	3.972 FIX	1.707	successful	2	-	-	-	-
5	AUC/MIC	5	0.820	119	22.530	8.988	4.275	1 FIX	successful	2	-	-	-	-
6	AUC/MIC	6	0.840	116	17.420	6.645	3.59	1.934	successful	2	-	-	-	-
7	AUC/MIC	7	0.818	123	18.320	7.027 FIX	3.683	1 FIX	successful	2	-	-	-	-
8	AUC/MIC	8	0.839	115	17.940	7.027 FIX	3.724	1.742	successful	2	-	-	-	-
9	Cmax/MIC	1	0.556	148	2.621	7.027 FIX	3.972 FIX	1 FIX	successful	1	-	-	-	-
10	Cmax/MIC	2	0.554	149	2.638	7.027 FIX	3.972 FIX	1.089	successful	1	-	-	-	-
11	Cmax/MIC	3	0.561	149	3.945	8.412	3.972 FIX	1 FIX	successful	1	-	-	-	-
12	Cmax/MIC	4	0.568	150	15.280	14.32	3.972 FIX	0.697	successful	1	-	-	-	-
13	Cmax/MIC	5	0.562	151	4.880	8.642	3.702	1 FIX	successful	1	-	-	-	-
14	Cmax/MIC	6							unsuccessful	1	-	-	-	-
15	Cmax/MIC	7	0.558	150	3.015	7.027 FIX	3.764	1 FIX	successful	1	-	-	-	-
16	Cmax/MIC	8							unsuccessful	1	-	-	-	-
17	T>MIC	1	0.692	136	17.740	7.027 FIX	3.972 FIX	1 FIX	successful	2	-	-	-	-
18	T>MIC	2	0.813	119	21.930	7.027 FIX	3.972 FIX	2.729	successful	2	-	-	-	-
19	T>MIC	3	0.760	128	49.700	11.61	3.972 FIX	1 FIX	successful	2	-	-	-	-
20	T>MIC	4	0.813	121	21.620	6.924	3.972 FIX	2.813	successful	2	-	-	-	-
21	T>MIC	5	0.760	130	55.490	11.67	3.708	1 FIX	successful	2	-	-	-	-
22	T>MIC	6	0.816	122	22.910	6.393	3.476	3.045	successful	2	-	-	-	-
23	T>MIC	7	0.730	136	25.880	7.027 FIX	3.281	1 FIX	successful	2	-	-	-	-
24	T>MIC	8	0.814	121	23.760	7.027 FIX	3.677	2.597	successful	2	-	-	-	-

Dudhani et al. (2010) PMID:20573659 Drug:Colistin

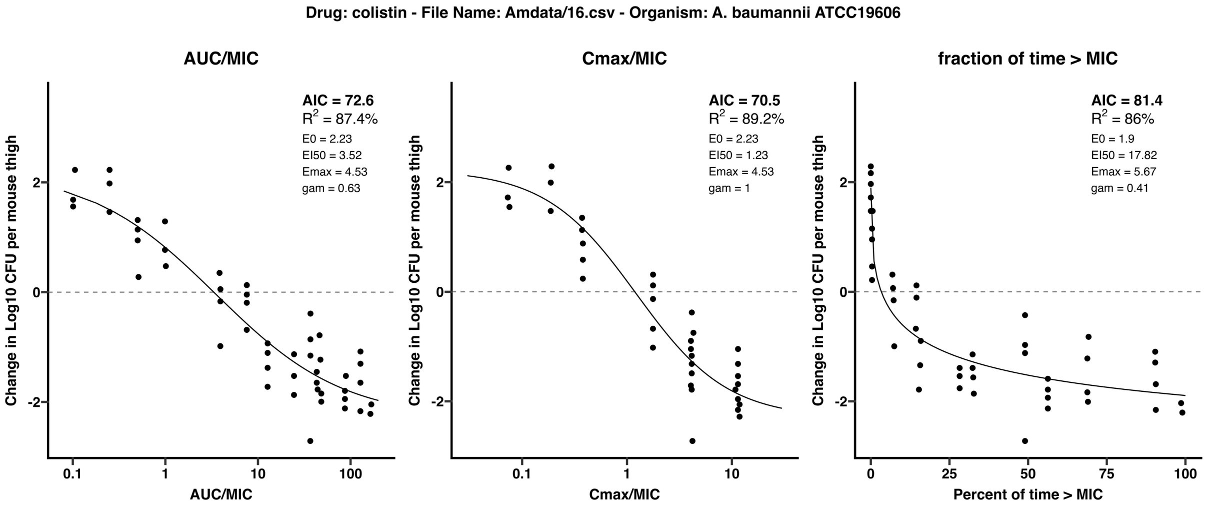

#	Model	Model Number	R²	AIC	EC50 hat	Emax hat	E0 hat	Gamma hat	Status	Best Fit Model	Optimal PKPD Index	Target for Stasis	Log1 Kill	Log2 Kill
1	AUC/MIC	1	0.868	92.5	4.402	4.531 FIX	2.227 FIX	1 FIX	successful	2	-	-	-	-
2	AUC/MIC	2	0.874	72.6	3.517	4.531 FIX	2.227 FIX	0.6288	successful	2	-	-	-	-
3	AUC/MIC	3	0.862	78.5	2.163	3.93	2.227 FIX	1 FIX	successful	2	-	-	-	-
4	AUC/MIC	4	0.874	74.2	2.935	4.34	2.227 FIX	0.6828	successful	2	-	-	-	-
5	AUC/MIC	5	0.869	76.2	3.606	3.582	1.759	1 FIX	successful	2	-	-	-	-
6	AUC/MIC	6	0.875	76.1	2.627	4.616	2.424	0.6249	successful	2	-	-	-	-
7	AUC/MIC	7	0.846	89.9	1.367	4.531 FIX	2.799	1 FIX	successful	2	-	-	-	-
8	AUC/MIC	8							unsuccessful	2	-	-	-	-
9	Cmax/MIC	1	0.892	70.5	1.227	4.531 FIX	2.227 FIX	1 FIX	successful	1	Cmax/MIC	1.19	3.04	17.06
10	Cmax/MIC	2	0.891	72.1	1.207	4.531 FIX	2.227 FIX	0.9465	successful	1	-	-	-	-
11	Cmax/MIC	3	0.892	72.0	1.113	4.403	2.227 FIX	1 FIX	successful	1	-	-	-	-
12	Cmax/MIC	4	0.892	74.0	1.135	4.436	2.227 FIX	0.981	successful	1	-	-	-	-
13	Cmax/MIC	5	0.892	73.9	1.174	4.369	2.167	1 FIX	successful	1	-	-	-	-
14	Cmax/MIC	6	0.892	75.9	1.186	4.269	2.112	1.042	successful	1	-	-	-	-
15	Cmax/MIC	7	0.892	72.5	1.153	4.531 FIX	2.273	1 FIX	successful	1	-	-	-	-
16	Cmax/MIC	8							unsuccessful	1	-	-	-	-
17	T>MIC	1	0.839	106.0	6.663	4.531 FIX	2.227 FIX	1 FIX	successful	6	-	-	-	-
18	T>MIC	2	0.850	82.2	2.730	4.531 FIX	2.227 FIX	0.516	successful	6	-	-	-	-
19	T>MIC	3	0.800	97.4	1.016	3.717	2.227 FIX	1 FIX	successful	6	-	-	-	-
20	T>MIC	4	0.858	81.4	26.620	6.771	2.227 FIX	0.3367	successful	6	-	-	-	-
21	T>MIC	5	0.839	86.4	7.220	3.42	1.414	1 FIX	successful	6	-	-	-	-
22	T>MIC	6	0.860	81.4	17.820	5.67	1.901	0.4082	successful	6	-	-	-	-
23	T>MIC	7	0.812	99.2	18.460	4.531 FIX	1.521	1 FIX	successful	6	-	-	-	-
24	T>MIC	8							unsuccessful	6	-	-	-	-

Zhao et al. (2017) PMID:28416552 Drug:Eravacycline

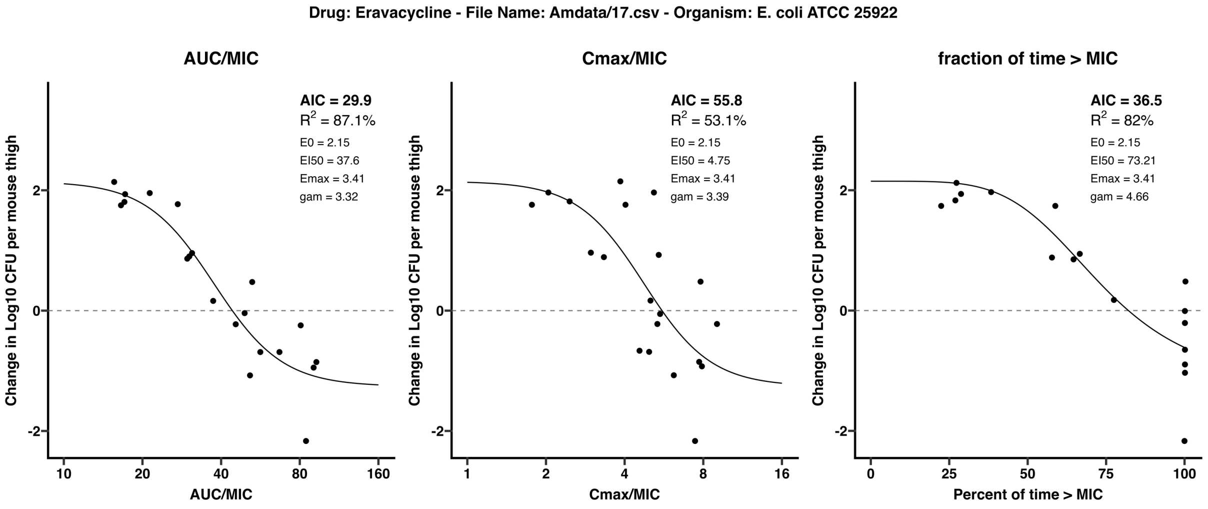

#	Model	Model Number	R²	AIC	EC50 hat	Emax hat	E0 hat	Gamma hat	Status	Best Fit Model	Optimal PKPD Index	Target for Stasis	Log1 Kill	Log2 Kill
1	AUC/MIC	1	0.857	51.9	36.420	3.411 FIX	2.15 FIX	1 FIX	successful	2	-	-	-	-
2	AUC/MIC	2	0.871	29.9	37.600	3.411 FIX	2.15 FIX	3.318	successful	2	AUC/MIC	44.2	79.7	160
3	AUC/MIC	3	0.800	40.2	709.600	30.85	2.15 FIX	1 FIX	successful	2	-	-	-	-
4	AUC/MIC	4	0.871	31.8	36.650	3.311	2.15 FIX	3.522	successful	2	-	-	-	-
5	AUC/MIC	5	0.857	33.8	32.830	8.126	4.767	1 FIX	successful	2	-	-	-	-
6	AUC/MIC	6							unsuccessful	2	-	-	-	-
7	AUC/MIC	7							unsuccessful	2	-	-	-	-
8	AUC/MIC	8	0.871	31.8	36.530	3.411 FIX	2.208	3.337	successful	2	-	-	-	-
9	Cmax/MIC	1	0.504	60.6	4.423	3.411 FIX	2.15 FIX	1 FIX	successful	2	-	-	-	-
10	Cmax/MIC	2	0.531	55.8	4.752	3.411 FIX	2.15 FIX	3.391	successful	2	-	-	-	-
11	Cmax/MIC	3							unsuccessful	2	-	-	-	-
12	Cmax/MIC	4	0.532	57.7	4.624	3.268	2.15 FIX	3.671	successful	2	-	-	-	-
13	Cmax/MIC	5	0.508	58.6	9.263	9.302	3.589	1 FIX	successful	2	-	-	-	-
14	Cmax/MIC	6	0.545	59.1	4.872	2.468	1.663	7.05	successful	2	-	-	-	-
15	Cmax/MIC	7							unsuccessful	2	-	-	-	-
16	Cmax/MIC	8	0.531	57.7	4.977	3.411 FIX	2.041	3.433	successful	2	-	-	-	-
17	T>MIC	1	0.741	55.2	61.680	3.411 FIX	2.15 FIX	1 FIX	successful	2	-	-	-	-
18	T>MIC	2	0.820	36.5	73.210	3.411 FIX	2.15 FIX	4.655	successful	2	-	-	-	-
19	T>MIC	3							unsuccessful	2	-	-	-	-
20	T>MIC	4	0.818	38.4	93.940	5.057	2.15 FIX	3.087	successful	2	-	-	-	-
21	T>MIC	5							unsuccessful	2	-	-	-	-
22	T>MIC	6	0.823	39.8	72.220	2.888	1.928	6.19	successful	2	-	-	-	-
23	T>MIC	7							unsuccessful	2	-	-	-	-
24	T>MIC	8	0.822	37.9	78.050	3.411 FIX	1.966	4.666	successful	2	-	-	-	-

Tashiro et al. (2021) PMID:33404990 Drug:Flomoxef

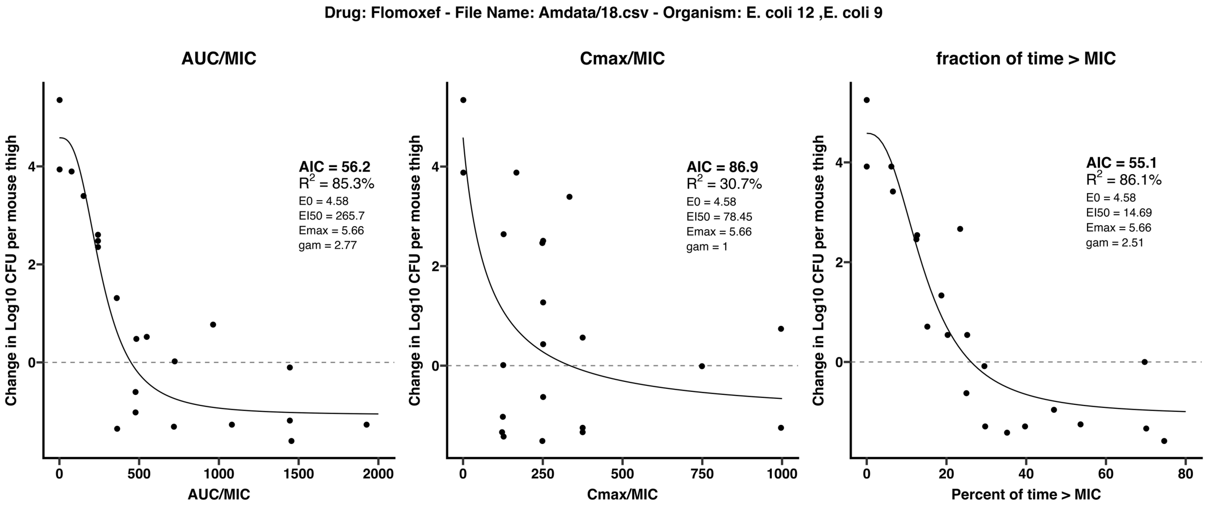

#	Model	Model Number	R²	AIC	EC50 hat	Emax hat	E0 hat	Gamma hat	Status	Best Fit Model	Optimal PKPD Index	Target for Stasis	Log1 Kill	Log2 Kill
1	AUC/MIC	1	0.785	65.0	185.2000	5.656 FIX	4.583 FIX	1 FIX	successful	2	-	-	-	-
2	AUC/MIC	2	0.853	56.2	265.7000	5.656 FIX	4.583 FIX	2.771	successful	2	-	-	-	-
3	AUC/MIC	3	0.803	62.4	351.6000	7.164	4.583 FIX	1 FIX	successful	2	-	-	-	-
4	AUC/MIC	4	0.856	57.6	257.9000	5.362	4.583 FIX	3.591	successful	2	-	-	-	-
5	AUC/MIC	5	0.804	63.9	303.4000	7.486	5.011	1 FIX	successful	2	-	-	-	-
6	AUC/MIC	6							unsuccessful	2	-	-	-	-
7	AUC/MIC	7	0.801	66.2	248.7000	5.656 FIX	4.193	1 FIX	successful	2	-	-	-	-
8	AUC/MIC	8							unsuccessful	2	-	-	-	-
9	Cmax/MIC	1	0.307	86.9	78.4500	5.656 FIX	4.583 FIX	1 FIX	successful	1	-	-	-	-
10	Cmax/MIC	2	0.334	87.8	40.9600	5.656 FIX	4.583 FIX	0.5568	successful	1	-	-	-	-
11	Cmax/MIC	3	0.349	87.3	-10.9800	3.946	4.583 FIX	1 FIX	successful	1	-	-	-	-
12	Cmax/MIC	4							unsuccessful	1	-	-	-	-
13	Cmax/MIC	5	0.350	89.2	-0.8892	0.3735	0.8025	1 FIX	successful	1	-	-	-	-
14	Cmax/MIC	6							unsuccessful	1	-	-	-	-
15	Cmax/MIC	7	0.350	87.2	2.4050	5.656 FIX	6.025	1 FIX	successful	1	-	-	-	-
16	Cmax/MIC	8							unsuccessful	1	-	-	-	-
17	T>MIC	1	0.780	66.6	9.8300	5.656 FIX	4.583 FIX	1 FIX	successful	2	-	-	-	-
18	T>MIC	2	0.861	55.1	14.6900	5.656 FIX	4.583 FIX	2.513	successful	2	T>MIC	26	80	80
19	T>MIC	3	0.830	59.4	26.0600	8.412	4.583 FIX	1 FIX	successful	2	-	-	-	-
20	T>MIC	4	0.865	56.3	15.8400	6.089	4.583 FIX	2.158	successful	2	-	-	-	-
21	T>MIC	5	0.831	60.9	22.6500	8.596	4.954	1 FIX	successful	2	-	-	-	-
22	T>MIC	6	0.865	58.2	16.0800	5.971	4.489	2.224	successful	2	-	-	-	-
23	T>MIC	7	0.817	67.1	14.6300	5.656 FIX	4.03	1 FIX	successful	2	-	-	-	-
24	T>MIC	8	0.864	56.4	16.2000	5.656 FIX	4.324	2.444	successful	2	-	-	-	-

Roelofsen et al. (2022) PMID:36009918 Drug:Flucloxacillin

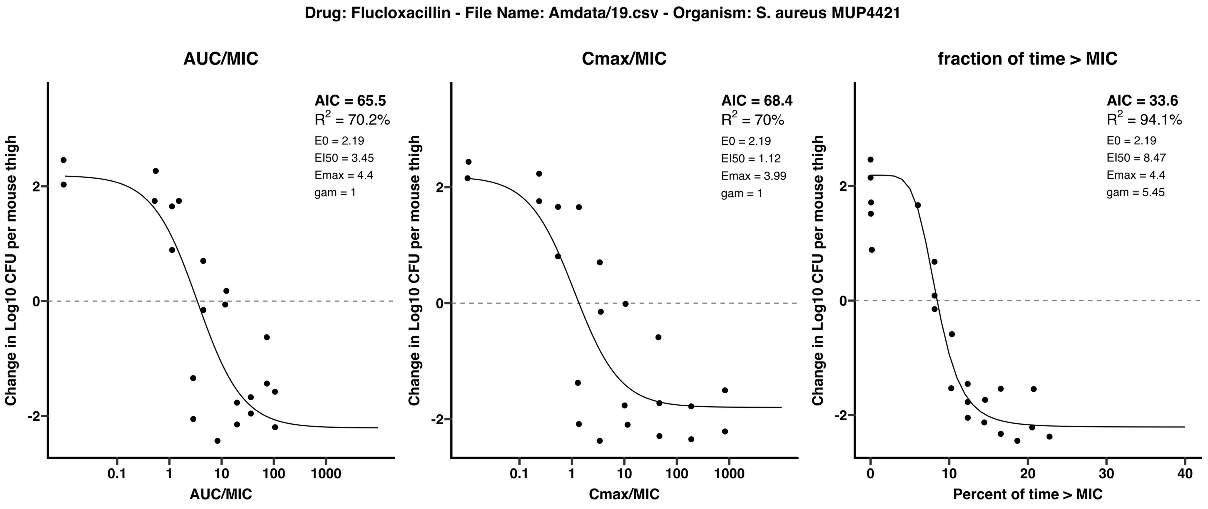

#	Model	Model Number	R²	AIC	EC50 hat	Emax hat	E0 hat	Gamma hat	Status	Best Fit Model	Optimal PKPD Index	Target for Stasis	Log1 Kill	Log2 Kill
1	AUC/MIC	1	0.702	65.5	3.4510	4.398 FIX	2.19 FIX	1 FIX	successful	1	-	-	-	-
2	AUC/MIC	2	0.700	67.5	3.4780	4.398 FIX	2.19 FIX	0.9597	successful	1	-	-	-	-
3	AUC/MIC	3	0.711	66.5	2.7020	4.024	2.19 FIX	1 FIX	successful	1	-	-	-	-
4	AUC/MIC	4							unsuccessful	1	-	-	-	-
5	AUC/MIC	5	0.714	67.9	2.1580	4.387	2.589	1 FIX	successful	1	-	-	-	-
6	AUC/MIC	6							unsuccessful	1	-	-	-	-
7	AUC/MIC	7	0.714	65.9	2.1530	4.398 FIX	2.596	1 FIX	successful	1	-	-	-	-
8	AUC/MIC	8	0.728	67.2	1.8730	4.398 FIX	2.746	1.417	successful	1	-	-	-	-
9	Cmax/MIC	1	0.690	68.1	1.5010	4.398 FIX	2.19 FIX	1 FIX	successful	3	-	-	-	-
10	Cmax/MIC	2	0.687	69.6	1.5650	4.398 FIX	2.19 FIX	0.7855	successful	3	-	-	-	-
11	Cmax/MIC	3	0.700	68.4	1.1180	3.987	2.19 FIX	1 FIX	successful	3	-	-	-	-
12	Cmax/MIC	4	0.701	70.2	1.0170	3.868	2.19 FIX	1.268	successful	3	-	-	-	-
13	Cmax/MIC	5	0.702	70.0	0.9058	4.327	2.554	1 FIX	successful	3	-	-	-	-
14	Cmax/MIC	6	0.703	72.0	0.8990	4.201	2.493	1.141	successful	3	-	-	-	-
15	Cmax/MIC	7	0.702	68.1	0.8865	4.398 FIX	2.613	1 FIX	successful	3	-	-	-	-
16	Cmax/MIC	8	0.703	70.0	0.8748	4.398 FIX	2.627	1.041	successful	3	-	-	-	-
17	T>MIC	1	0.703	65.8	4.5970	4.398 FIX	2.19 FIX	1 FIX	successful	2	-	-	-	-
18	T>MIC	2	0.941	33.6	8.4710	4.398 FIX	2.19 FIX	5.447	successful	2	T>MIC	8	10	15
19	T>MIC	3	0.835	53.9	37.3600	13.09	2.19 FIX	1 FIX	successful	2	-	-	-	-
20	T>MIC	4							unsuccessful	2	-	-	-	-
21	T>MIC	5	0.835	55.3	47.0800	14.51	1.96	1 FIX	successful	2	-	-	-	-
22	T>MIC	6	0.945	32.2	8.6970	3.875	1.791	6.703	successful	2	-	-	-	-
23	T>MIC	7	0.757	65.0	7.7290	4.398 FIX	1.627	1 FIX	successful	2	-	-	-	-
24	T>MIC	8	0.940	34.0	8.8160	4.398 FIX	2.02	5.002	successful	2	-	-	-	-

Roelofsen et al. (2022) PMID:36009918 Drug:Flucloxacillin

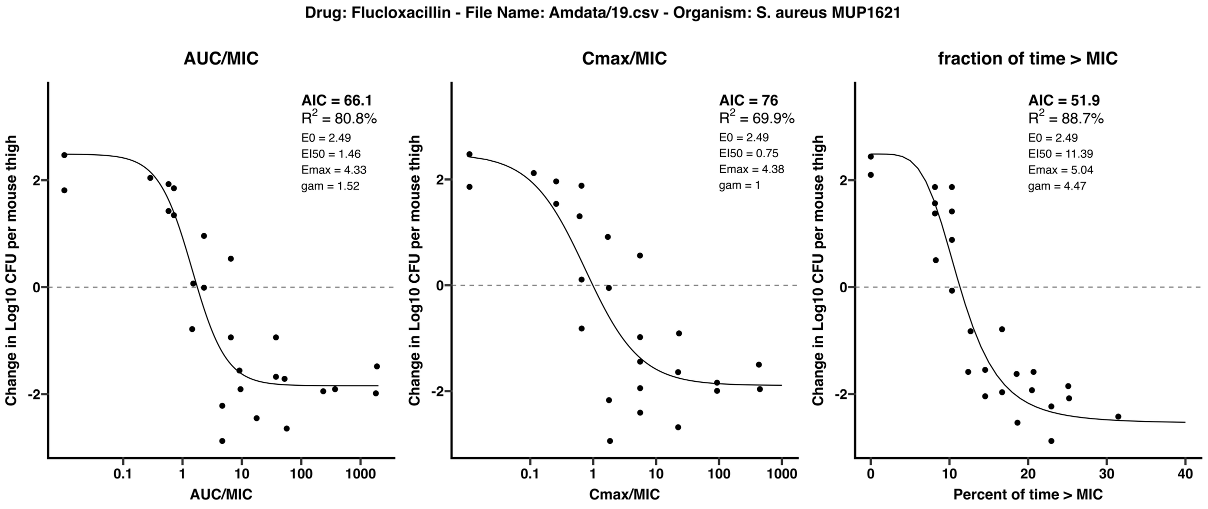

#	Model	Model Number	R²	AIC	EC50 hat	Emax hat	E0 hat	Gamma hat	Status	Best Fit Model	Optimal PKPD Index	Target for Stasis	Log1 Kill	Log2 Kill
1	AUC/MIC	1	0.783	67.7	2.0910	5.037 FIX	2.49 FIX	1 FIX	successful	4	-	-	-	-
2	AUC/MIC	2	0.781	69.7	2.0920	5.037 FIX	2.49 FIX	0.9836	successful	4	-	-	-	-
3	AUC/MIC	3	0.790	66.3	1.5750	4.542	2.49 FIX	1 FIX	successful	4	-	-	-	-
4	AUC/MIC	4	0.808	66.1	1.4580	4.333	2.49 FIX	1.523	successful	4	-	-	-	-
5	AUC/MIC	5	0.790	68.3	1.5210	4.61	2.562	1 FIX	successful	4	-	-	-	-
6	AUC/MIC	6	0.809	67.8	1.6090	4.053	2.227	1.671	successful	4	-	-	-	-
7	AUC/MIC	7	0.789	67.0	1.3760	5.037 FIX	2.904	1 FIX	successful	4	-	-	-	-
8	AUC/MIC	8							unsuccessful	4	-	-	-	-
9	Cmax/MIC	1	0.688	78.0	1.1380	5.037 FIX	2.49 FIX	1 FIX	successful	3	-	-	-	-
10	Cmax/MIC	2	0.671	79.1	1.1480	5.037 FIX	2.49 FIX	0.7375	successful	3	-	-	-	-
11	Cmax/MIC	3	0.699	76.0	0.7514	4.383	2.49 FIX	1 FIX	successful	3	-	-	-	-
12	Cmax/MIC	4	0.711	77.0	0.7093	4.175	2.49 FIX	1.554	successful	3	-	-	-	-
13	Cmax/MIC	5	0.699	78.0	0.7344	4.425	2.535	1 FIX	successful	3	-	-	-	-
14	Cmax/MIC	6	0.712	78.8	0.7791	3.857	2.207	1.805	successful	3	-	-	-	-
15	Cmax/MIC	7	0.697	76.9	0.6291	5.037 FIX	3.04	1 FIX	successful	3	-	-	-	-
16	Cmax/MIC	8	0.696	78.9	0.6308	5.037 FIX	3.028	0.9661	successful	3	-	-	-	-
17	T>MIC	1	0.631	83.6	8.0580	5.037 FIX	2.49 FIX	1 FIX	successful	2	-	-	-	-
18	T>MIC	2	0.887	51.9	11.3900	5.037 FIX	2.49 FIX	4.474	successful	2	T>MIC	11	14	18
19	T>MIC	3	0.767	70.3	61.9400	16.64	2.49 FIX	1 FIX	successful	2	-	-	-	-
20	T>MIC	4	0.890	52.9	11.0300	4.77	2.49 FIX	5.335	successful	2	-	-	-	-
21	T>MIC	5	0.769	71.9	45.6400	14.6	2.848	1 FIX	successful	2	-	-	-	-
22	T>MIC	6	0.896	53.2	11.4900	3.857	1.789	10.49	successful	2	-	-	-	-
23	T>MIC	7	0.693	84.3	12.0000	5.037 FIX	1.957	1 FIX	successful	2	-	-	-	-
24	T>MIC	8							unsuccessful	2	-	-	-	-

Chavan et al. (2023) PMID:36346323 Drug:Fosfomycin

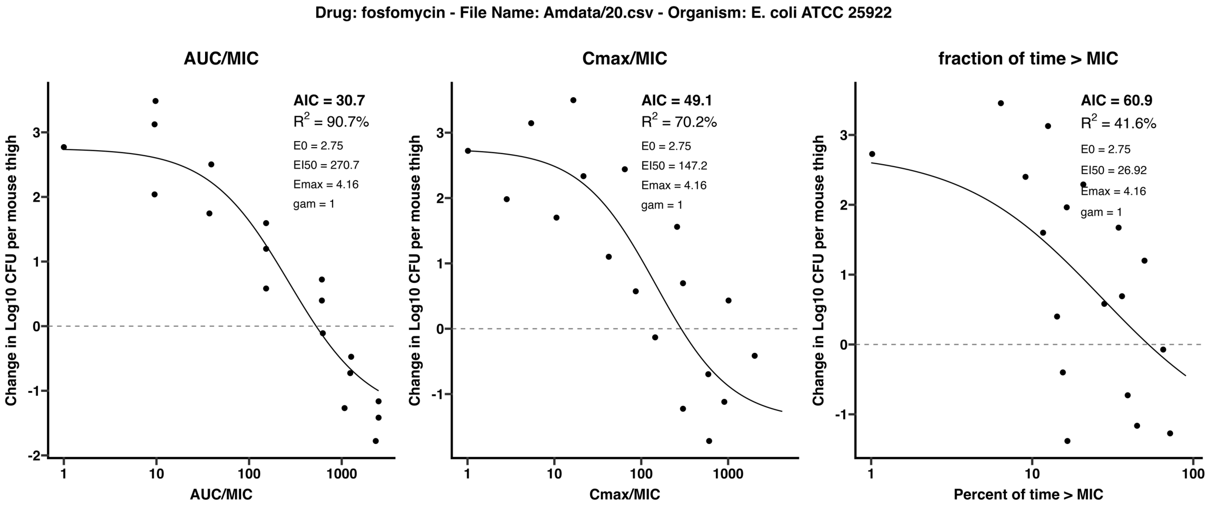

#	Model	Model Number	R²	AIC	EC50 hat	Emax hat	E0 hat	Gamma hat	Status	Best Fit Model	Optimal PKPD Index	Target for Stasis	Log1 Kill	Log2 Kill
1	AUC/MIC	1	0.907	30.7	270.70	4.156 FIX	2.75 FIX	1 FIX	successful	1	AUC/MIC	529.5	2500.3	2501
2	AUC/MIC	2	0.905	32.6	276.80	4.156 FIX	2.75 FIX	1.062	successful	1	-	-	-	-
3	AUC/MIC	3	0.911	31.0	406.40	4.726	2.75 FIX	1 FIX	successful	1	-	-	-	-
4	AUC/MIC	4	0.918	31.7	761.50	5.913	2.75 FIX	0.7501	successful	1	-	-	-	-
5	AUC/MIC	5	0.911	33.0	406.30	4.726	2.75	1 FIX	successful	1	-	-	-	-
6	AUC/MIC	6	0.922	32.6	1,475.00	8.235	3.22	0.5296	successful	1	-	-	-	-
7	AUC/MIC	7	0.908	32.6	293.10	4.156 FIX	2.691	1 FIX	successful	1	-	-	-	-
8	AUC/MIC	8	0.906	34.4	307.70	4.156 FIX	2.673	1.071	successful	1	-	-	-	-
9	Cmax/MIC	1	0.702	49.1	147.20	4.156 FIX	2.75 FIX	1 FIX	successful	1	-	-	-	-
10	Cmax/MIC	2	0.701	51.1	146.80	4.156 FIX	2.75 FIX	0.9466	successful	1	-	-	-	-
11	Cmax/MIC	3	0.706	50.7	112.80	3.792	2.75 FIX	1 FIX	successful	1	-	-	-	-
12	Cmax/MIC	4	0.713	52.3	92.46	3.466	2.75 FIX	1.374	successful	1	-	-	-	-
13	Cmax/MIC	5	0.706	52.7	107.50	3.829	2.804	1 FIX	successful	1	-	-	-	-
14	Cmax/MIC	6	0.714	54.2	97.67	3.3	2.622	1.497	successful	1	-	-	-	-
15	Cmax/MIC	7	0.706	51.0	118.70	4.156 FIX	2.908	1 FIX	successful	1	-	-	-	-
16	Cmax/MIC	8	0.704	52.9	117.80	4.156 FIX	2.908	0.9466	successful	1	-	-	-	-
17	T>MIC	1	0.416	60.9	26.92	4.156 FIX	2.75 FIX	1 FIX	successful	1	-	-	-	-
18	T>MIC	2	0.412	62.4	26.02	4.156 FIX	2.75 FIX	1.313	successful	1	-	-	-	-
19	T>MIC	3	0.409	62.6	45.85	5.533	2.75 FIX	1 FIX	successful	1	-	-	-	-
20	T>MIC	4							unsuccessful	1	-	-	-	-
21	T>MIC	5	0.416	64.3	23.73	5.209	3.391	1 FIX	successful	1	-	-	-	-
22	T>MIC	6							unsuccessful	1	-	-	-	-
23	T>MIC	7	0.415	62.7	19.00	4.156 FIX	3.095	1 FIX	successful	1	-	-	-	-
24	T>MIC	8	0.420	64.2	15.69	4.156 FIX	3.355	1.449	successful	1	-	-	-	-

Chavan et al. (2023) PMID:36346323 Drug:Fosfomycin

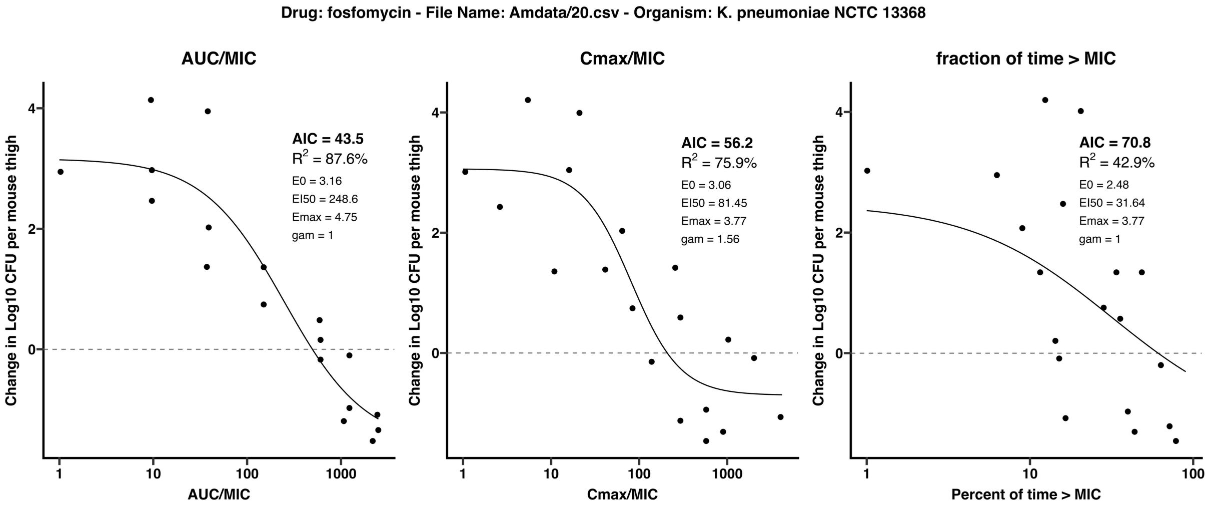

#	Model	Model Number	R²	AIC	EC50 hat	Emax hat	E0 hat	Gamma hat	Status	Best Fit Model	Optimal PKPD Index	Target for Stasis	Log1 Kill	Log2 Kill
1	AUC/MIC	1	0.875	45.8	301.80	3.765 FIX	2.48 FIX	1 FIX	successful	5	-	-	-	-
2	AUC/MIC	2	0.860	46.1	315.40	3.765 FIX	2.48 FIX	1.326	successful	5	-	-	-	-
3	AUC/MIC	3	0.863	46.0	523.00	4.611	2.48 FIX	1 FIX	successful	5	-	-	-	-
4	AUC/MIC	4	0.861	47.8	415.60	4.202	2.48 FIX	1.159	successful	5	-	-	-	-
5	AUC/MIC	5	0.876	43.5	248.60	4.749	3.163	1 FIX	successful	5	AUC/MIC	495.8	1766.1	2500
6	AUC/MIC	6	0.880	44.9	355.50	5.87	3.412	0.7159	successful	5	-	-	-	-
7	AUC/MIC	7	0.874	46.2	190.90	3.765 FIX	2.827	1 FIX	successful	5	-	-	-	-
8	AUC/MIC	8	0.864	46.2	194.40	3.765 FIX	2.85	1.364	successful	5	-	-	-	-
9	Cmax/MIC	1	0.732	56.1	164.60	3.765 FIX	2.48 FIX	1 FIX	successful	8	-	-	-	-
10	Cmax/MIC	2	0.733	57.4	159.60	3.765 FIX	2.48 FIX	1.318	successful	8	-	-	-	-
11	Cmax/MIC	3	0.735	58.1	155.30	3.679	2.48 FIX	1 FIX	successful	8	-	-	-	-
12	Cmax/MIC	4	0.751	58.2	103.00	3.138	2.48 FIX	1.933	successful	8	-	-	-	-
13	Cmax/MIC	5	0.751	56.8	86.21	4.352	3.307	1 FIX	successful	8	-	-	-	-
14	Cmax/MIC	6	0.759	58.2	81.59	3.786	3.072	1.546	successful	8	-	-	-	-
15	Cmax/MIC	7	0.751	55.8	85.05	3.765 FIX	2.997	1 FIX	successful	8	-	-	-	-
16	Cmax/MIC	8	0.759	56.2	81.45	3.765 FIX	3.062	1.564	successful	8	-	-	-	-
17	T>MIC	1	0.429	70.8	31.64	3.765 FIX	2.48 FIX	1 FIX	successful	1	-	-	-	-
18	T>MIC	2	0.419	71.1	30.03	3.765 FIX	2.48 FIX	1.877	successful	1	-	-	-	-
19	T>MIC	3	0.428	70.9	284.20	17.53	2.48 FIX	1 FIX	successful	1	-	-	-	-
20	T>MIC	4	0.428	72.8	77.66	7.351	2.48 FIX	1.251	successful	1	-	-	-	-
21	T>MIC	5	0.434	72.5	68.30	7.873	3.082	1 FIX	successful	1	-	-	-	-
22	T>MIC	6							unsuccessful	1	-	-	-	-
23	T>MIC	7	0.421	72.7	23.20	3.765 FIX	2.78	1 FIX	successful	1	-	-	-	-
24	T>MIC	8	0.420	73.1	27.91	3.765 FIX	2.577	1.835	successful	1	-	-	-	-

Andes and Craig (2003) PMID:14638504 Drug:Garenoxacin

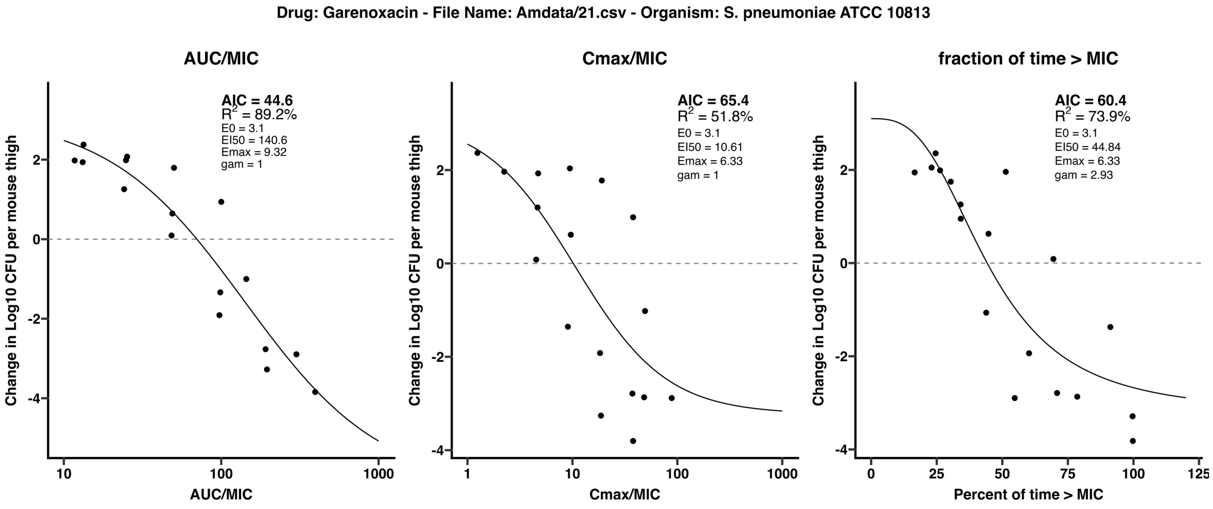

#	Model	Model Number	R²	AIC	EC50 hat	Emax hat	E0 hat	Gamma hat	Status	Best Fit Model	Optimal PKPD Index	Target for Stasis	Log1 Kill	Log2 Kill
1	AUC/MIC	1	0.864	52.4	61.20	6.327 FIX	3.1 FIX	1 FIX	successful	3	-	-	-	-
2	AUC/MIC	2	0.868	48.8	65.19	6.327 FIX	3.1 FIX	1.525	successful	3	-	-	-	-
3	AUC/MIC	3	0.892	44.6	140.60	9.325	3.1 FIX	1 FIX	successful	3	AUC/MIC	70	110.3	169.7
4	AUC/MIC	4	0.892	46.6	138.50	9.263	3.1 FIX	1.007	successful	3	-	-	-	-
5	AUC/MIC	5	0.892	46.5	155.10	9.435	2.959	1 FIX	successful	3	-	-	-	-
6	AUC/MIC	6	0.896	47.8	108.90	6.566	2.274	1.648	successful	3	-	-	-	-
7	AUC/MIC	7							unsuccessful	3	-	-	-	-
8	AUC/MIC	8	0.896	45.8	106.20	6.327 FIX	2.211	1.748	successful	3	-	-	-	-
9	Cmax/MIC	1	0.518	65.4	10.61	6.327 FIX	3.1 FIX	1 FIX	successful	1	-	-	-	-
10	Cmax/MIC	2	0.518	67.4	10.62	6.327 FIX	3.1 FIX	1.001	successful	1	-	-	-	-
11	Cmax/MIC	3	0.518	67.4	11.06	6.425	3.1 FIX	1 FIX	successful	1	-	-	-	-
12	Cmax/MIC	4	0.519	69.4	13.00	6.853	3.1 FIX	0.897	successful	1	-	-	-	-
13	Cmax/MIC	5	0.519	69.4	12.75	6.329	2.865	1 FIX	successful	1	-	-	-	-
14	Cmax/MIC	6	0.519	71.4	12.97	6.631	2.993	0.9371	successful	1	-	-	-	-
15	Cmax/MIC	7	0.519	67.4	12.75	6.327 FIX	2.864	1 FIX	successful	1	-	-	-	-
16	Cmax/MIC	8	0.519	69.4	12.75	6.327 FIX	2.864	0.9982	successful	1	-	-	-	-
17	T>MIC	1	0.725	70.1	41.17	6.327 FIX	3.1 FIX	1 FIX	successful	2	-	-	-	-
18	T>MIC	2	0.739	60.4	44.84	6.327 FIX	3.1 FIX	2.927	successful	2	-	-	-	-
19	T>MIC	3							unsuccessful	2	-	-	-	-
20	T>MIC	4	0.746	61.8	55.80	7.904	3.1 FIX	2.203	successful	2	-	-	-	-
21	T>MIC	5	0.740	62.2	145.50	19.52	4.689	1 FIX	successful	2	-	-	-	-
22	T>MIC	6	0.747	63.7	53.05	6.415	2.552	2.952	successful	2	-	-	-	-
23	T>MIC	7							unsuccessful	2	-	-	-	-
24	T>MIC	8	0.747	61.7	52.80	6.327 FIX	2.526	3.012	successful	2	-	-	-	-

Andes and Craig (2003) PMID:14638504 Drug:Garenoxacin

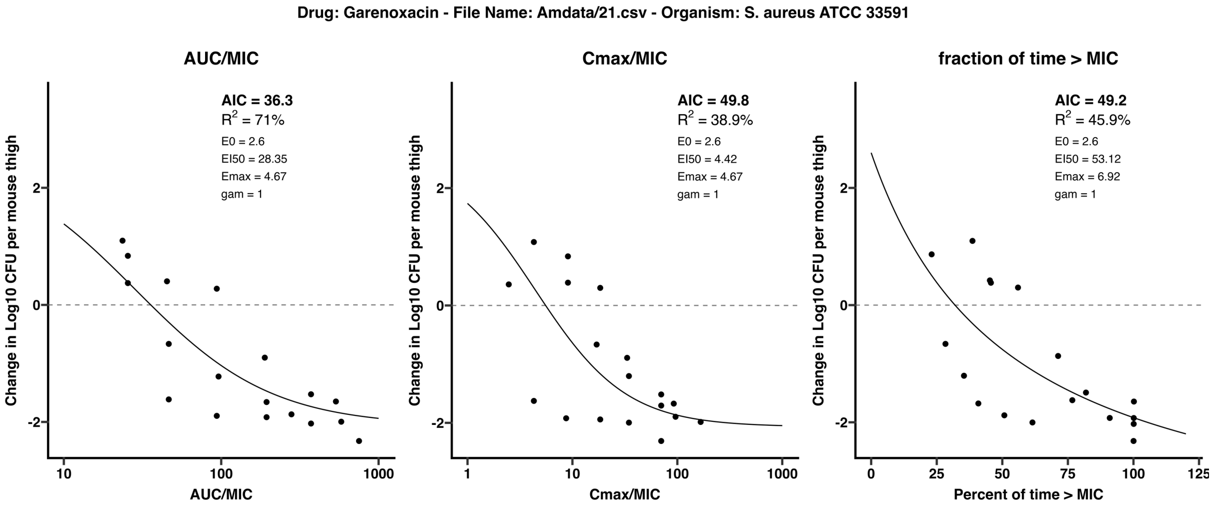

#	Model	Model Number	R²	AIC	EC50 hat	Emax hat	E0 hat	Gamma hat	Status	Best Fit Model	Optimal PKPD Index	Target for Stasis	Log1 Kill	Log2 Kill
1	AUC/MIC	1	0.710	36.3	28.350	4.667 FIX	2.6 FIX	1 FIX	successful	1	AUC/MIC	35.7	95.7	1000
2	AUC/MIC	2	0.711	37.6	31.100	4.667 FIX	2.6 FIX	1.213	successful	1	-	-	-	-
3	AUC/MIC	3	0.707	37.9	31.970	4.834	2.6 FIX	1 FIX	successful	1	-	-	-	-
4	AUC/MIC	4	0.712	39.6	30.260	4.569	2.6 FIX	1.326	successful	1	-	-	-	-
5	AUC/MIC	5	0.722	39.0	3.930	20.01	18.01	1 FIX	successful	1	-	-	-	-
6	AUC/MIC	6							unsuccessful	1	-	-	-	-
7	AUC/MIC	7	0.706	38.0	33.350	4.667 FIX	2.45	1 FIX	successful	1	-	-	-	-
8	AUC/MIC	8	0.712	39.6	28.990	4.667 FIX	2.718	1.338	successful	1	-	-	-	-
9	Cmax/MIC	1	0.389	49.8	4.415	4.667 FIX	2.6 FIX	1 FIX	successful	1	-	-	-	-
10	Cmax/MIC	2							unsuccessful	1	-	-	-	-
11	Cmax/MIC	3	0.380	51.5	3.513	4.431	2.6 FIX	1 FIX	successful	1	-	-	-	-
12	Cmax/MIC	4	0.428	51.9	23.060	7.229	2.6 FIX	0.3389	successful	1	-	-	-	-
13	Cmax/MIC	5	0.435	51.7	23.650	2.889	0.4257	1 FIX	successful	1	-	-	-	-
14	Cmax/MIC	6	0.439	53.6	22.920	1.995	0.006172	1.988	successful	1	-	-	-	-
15	Cmax/MIC	7	0.377	51.6	3.272	4.667 FIX	2.821	1 FIX	successful	1	-	-	-	-
16	Cmax/MIC	8	0.430	51.9	27.330	4.667 FIX	1.222	0.5517	successful	1	-	-	-	-
17	T>MIC	1	0.445	50.6	17.680	4.667 FIX	2.6 FIX	1 FIX	successful	3	-	-	-	-
18	T>MIC	2	0.426	50.3	27.600	4.667 FIX	2.6 FIX	1.822	successful	3	-	-	-	-
19	T>MIC	3	0.459	49.2	53.120	6.921	2.6 FIX	1 FIX	successful	3	-	-	-	-
20	T>MIC	4							unsuccessful	3	-	-	-	-
21	T>MIC	5	0.465	51.0	248.500	11.13	1.199	1 FIX	successful	3	-	-	-	-
22	T>MIC	6							unsuccessful	3	-	-	-	-
23	T>MIC	7							unsuccessful	3	-	-	-	-
24	T>MIC	8	0.464	51.0	86.820	4.667 FIX	0.6357	1.734	successful	3	-	-	-	-

Andes and Craig (2002) PMID:12019073 Drug:Gatifloxacin

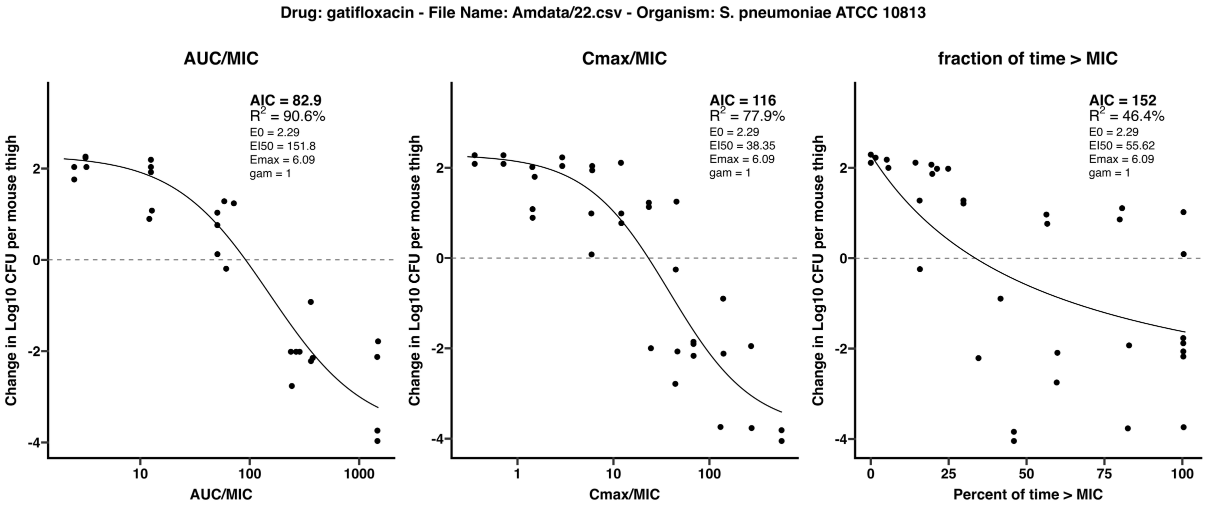

#	Model	Model Number	R²	AIC	EC50 hat	Emax hat	E0 hat	Gamma hat	Status	Best Fit Model	Optimal PKPD Index	Target for Stasis	Log1 Kill	Log2 Kill
1	AUC/MIC	1	0.906	82.9	151.80	6.089 FIX	2.292 FIX	1 FIX	successful	1	AUC/MIC	91.6	178.7	362.6
2	AUC/MIC	2	0.906	84.8	151.20	6.089 FIX	2.292 FIX	0.9624	successful	1	-	-	-	-
3	AUC/MIC	3	0.907	84.1	133.00	5.817	2.292 FIX	1 FIX	successful	1	-	-	-	-
4	AUC/MIC	4	0.909	85.7	121.40	5.586	2.292 FIX	1.134	successful	1	-	-	-	-
5	AUC/MIC	5	0.907	86.1	136.20	5.795	2.257	1 FIX	successful	1	-	-	-	-
6	AUC/MIC	6	0.910	86.8	126.30	5.199	2.07	1.328	successful	1	-	-	-	-
7	AUC/MIC	7	0.906	84.9	149.20	6.089 FIX	2.311	1 FIX	successful	1	-	-	-	-
8	AUC/MIC	8	0.906	86.8	146.70	6.089 FIX	2.324	0.959	successful	1	-	-	-	-
9	Cmax/MIC	1	0.779	116.0	38.35	6.089 FIX	2.292 FIX	1 FIX	successful	1	-	-	-	-
10	Cmax/MIC	2	0.779	118.0	38.39	6.089 FIX	2.292 FIX	0.9853	successful	1	-	-	-	-
11	Cmax/MIC	3	0.779	118.0	38.27	6.084	2.292 FIX	1 FIX	successful	1	-	-	-	-
12	Cmax/MIC	4	0.779	120.0	40.62	6.221	2.292 FIX	0.955	successful	1	-	-	-	-
13	Cmax/MIC	5	0.779	120.0	41.95	6.032	2.163	1 FIX	successful	1	-	-	-	-
14	Cmax/MIC	6	0.780	122.0	38.17	5.629	2.07	1.147	successful	1	-	-	-	-
15	Cmax/MIC	7	0.779	118.0	42.72	6.089 FIX	2.169	1 FIX	successful	1	-	-	-	-
16	Cmax/MIC	8							unsuccessful	1	-	-	-	-
17	T>MIC	1	0.464	152.0	55.62	6.089 FIX	2.292 FIX	1 FIX	successful	1	-	-	-	-
18	T>MIC	2	0.469	153.0	53.29	6.089 FIX	2.292 FIX	1.278	successful	1	-	-	-	-
19	T>MIC	3	0.462	154.0	68.98	6.819	2.292 FIX	1 FIX	successful	1	-	-	-	-
20	T>MIC	4							unsuccessful	1	-	-	-	-
21	T>MIC	5	0.464	156.0	53.00	6.76	2.672	1 FIX	successful	1	-	-	-	-
22	T>MIC	6							unsuccessful	1	-	-	-	-
23	T>MIC	7	0.463	154.0	43.90	6.089 FIX	2.63	1 FIX	successful	1	-	-	-	-
24	T>MIC	8	0.475	155.0	42.28	6.089 FIX	2.675	1.293	successful	1	-	-	-	-

Bulik et al. (2017) PMID:27872075 Drug:Gepotidacin

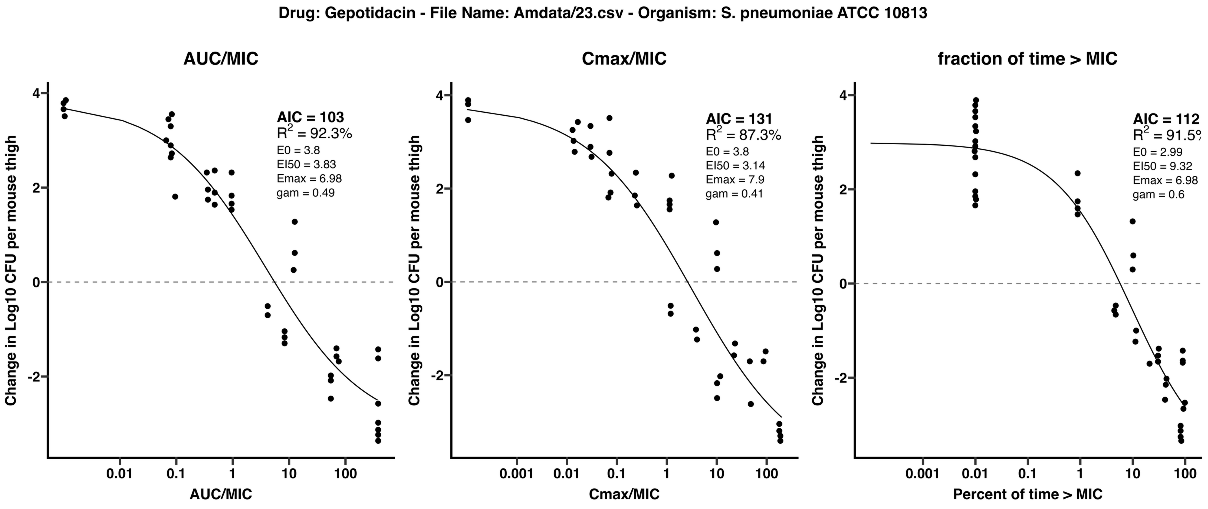

#	Model	Model Number	R²	AIC	EC50 hat	Emax hat	E0 hat	Gamma hat	Status	Best Fit Model	Optimal PKPD Index	Target for Stasis	Log1 Kill	Log2 Kill
1	AUC/MIC	1	0.899	149	3.450	6.976 FIX	3.8 FIX	1 FIX	successful	2	-	-	-	-
2	AUC/MIC	2	0.923	103	3.825	6.976 FIX	3.8 FIX	0.4878	successful	2	AUC/MIC	5.521	19.361	100.771
3	AUC/MIC	3	0.892	123	1.359	5.74	3.8 FIX	1 FIX	successful	2	-	-	-	-
4	AUC/MIC	4	0.923	105	3.293	6.796	3.8 FIX	0.5103	successful	2	-	-	-	-
5	AUC/MIC	5	0.901	117	2.605	5.248	3.132	1 FIX	successful	2	-	-	-	-
6	AUC/MIC	6	0.923	107	3.215	6.959	3.898	0.4914	successful	2	-	-	-	-
7	AUC/MIC	7	0.891	148	1.319	6.976 FIX	4.392	1 FIX	successful	2	-	-	-	-
8	AUC/MIC	8							unsuccessful	2	-	-	-	-
9	Cmax/MIC	1	0.841	160	1.736	6.976 FIX	3.8 FIX	1 FIX	successful	4	-	-	-	-
10	Cmax/MIC	2	0.870	131	1.550	6.976 FIX	3.8 FIX	0.4872	successful	4	-	-	-	-
11	Cmax/MIC	3	0.833	148	0.819	5.846	3.8 FIX	1 FIX	successful	4	-	-	-	-
12	Cmax/MIC	4	0.873	131	3.140	7.898	3.8 FIX	0.4147	successful	4	-	-	-	-
13	Cmax/MIC	5	0.842	142	1.446	5.283	3.05	1 FIX	successful	4	-	-	-	-
14	Cmax/MIC	6	0.873	133	3.361	8.355	3.979	0.3837	successful	4	-	-	-	-
15	Cmax/MIC	7	0.839	162	2.035	6.976 FIX	3.673	1 FIX	successful	4	-	-	-	-
16	Cmax/MIC	8	0.871	132	1.982	6.976 FIX	3.644	0.4903	successful	4	-	-	-	-
17	T>MIC	1	0.909	144	5.255	6.976 FIX	3.8 FIX	1 FIX	successful	8	-	-	-	-
18	T>MIC	2	0.891	124	2.100	6.976 FIX	3.8 FIX	0.4443	successful	8	-	-	-	-
19	T>MIC	3	0.900	141	2.945	6.229	3.8 FIX	1 FIX	successful	8	-	-	-	-
20	T>MIC	4	0.910	114	468.400	16.42	3.8 FIX	0.2688	successful	8	-	-	-	-
21	T>MIC	5	0.909	115	5.688	5.54	2.81	1 FIX	successful	8	-	-	-	-
22	T>MIC	6	0.915	114	8.498	6.763	2.966	0.623	successful	8	-	-	-	-
23	T>MIC	7	0.893	129	12.450	6.976 FIX	2.933	1 FIX	successful	8	-	-	-	-
24	T>MIC	8	0.915	112	9.321	6.976 FIX	2.988	0.5952	successful	8	-	-	-	-

Bulik et al. (2017) PMID:27872075 Drug:Gepotidacin

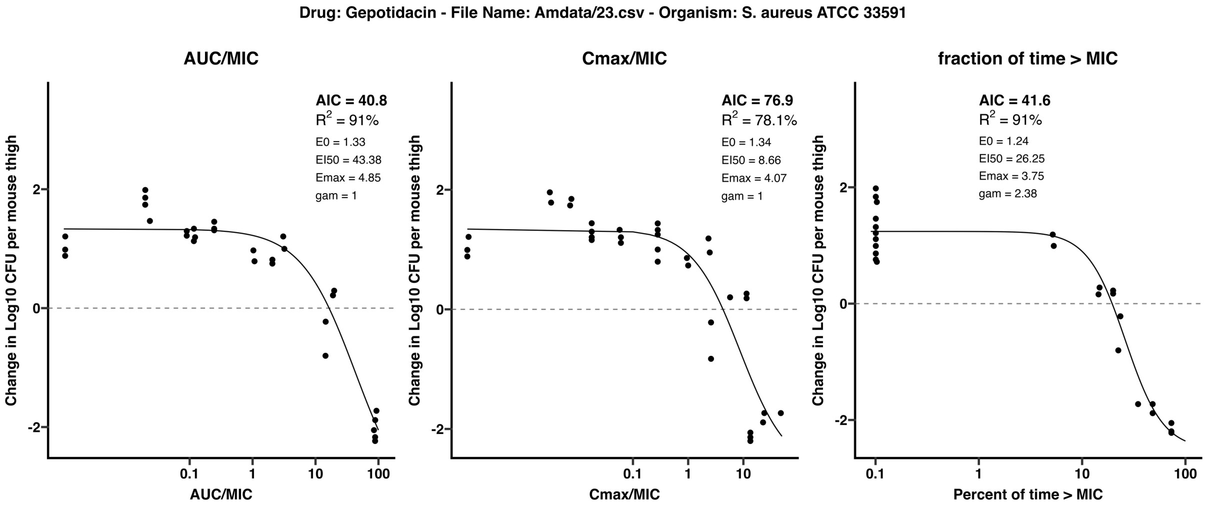

#	Model	Model Number	R²	AIC	EC50 hat	Emax hat	E0 hat	Gamma hat	Status	Best Fit Model	Optimal PKPD Index	Target for Stasis	Log1 Kill	Log2 Kill
1	AUC/MIC	1	0.862	82.8	11.860	4.065 FIX	1.98 FIX	1 FIX	successful	5	-	-	-	-
2	AUC/MIC	2	0.821	76.0	9.455	4.065 FIX	1.98 FIX	0.5929	successful	5	-	-	-	-
3	AUC/MIC	3	0.877	84.4	14.870	4.368	1.98 FIX	1 FIX	successful	5	-	-	-	-
4	AUC/MIC	4							unsuccessful	5	-	-	-	-
5	AUC/MIC	5	0.910	40.8	43.380	4.852	1.332	1 FIX	successful	5	AUC/MIC	16.401	40.101	95.101
6	AUC/MIC	6							unsuccessful	5	-	-	-	-
7	AUC/MIC	7	0.906	41.7	29.100	4.065 FIX	1.34	1 FIX	successful	5	-	-	-	-
8	AUC/MIC	8	0.906	43.1	29.830	4.065 FIX	1.319	1.105	successful	5	-	-	-	-
9	Cmax/MIC	1	0.752	97.0	3.587	4.065 FIX	1.98 FIX	1 FIX	successful	7	-	-	-	-
10	Cmax/MIC	2	0.713	93.7	3.237	4.065 FIX	1.98 FIX	0.6135	successful	7	-	-	-	-
11	Cmax/MIC	3	0.746	98.9	3.241	3.925	1.98 FIX	1 FIX	successful	7	-	-	-	-
12	Cmax/MIC	4							unsuccessful	7	-	-	-	-
13	Cmax/MIC	5	0.781	78.9	9.325	4.205	1.34	1 FIX	successful	7	-	-	-	-
14	Cmax/MIC	6	0.781	80.8	10.650	4.482	1.361	0.9175	successful	7	-	-	-	-
15	Cmax/MIC	7	0.781	76.9	8.658	4.065 FIX	1.342	1 FIX	successful	7	-	-	-	-
16	Cmax/MIC	8	0.781	78.9	8.672	4.065 FIX	1.339	1.009	successful	7	-	-	-	-
17	T>MIC	1	0.793	97.1	15.510	4.065 FIX	1.98 FIX	1 FIX	successful	6	-	-	-	-
18	T>MIC	2	0.867	95.6	18.590	4.065 FIX	1.98 FIX	1.866	successful	6	-	-	-	-
19	T>MIC	3	0.877	91.3	44.270	6.821	1.98 FIX	1 FIX	successful	6	-	-	-	-
20	T>MIC	4							unsuccessful	6	-	-	-	-
21	T>MIC	5	0.896	46.2	110.200	9.095	1.294	1 FIX	successful	6	-	-	-	-
22	T>MIC	6	0.910	41.6	26.250	3.752	1.241	2.378	successful	6	-	-	-	-
23	T>MIC	7	0.857	60.7	31.400	4.065 FIX	1.287	1 FIX	successful	6	-	-	-	-
24	T>MIC	8	0.910	40.0	28.810	4.065 FIX	1.253	2.041	successful	6	-	-	-	-

Ferrari et al. (2003) PMID:12543690 Drug:GV143253A

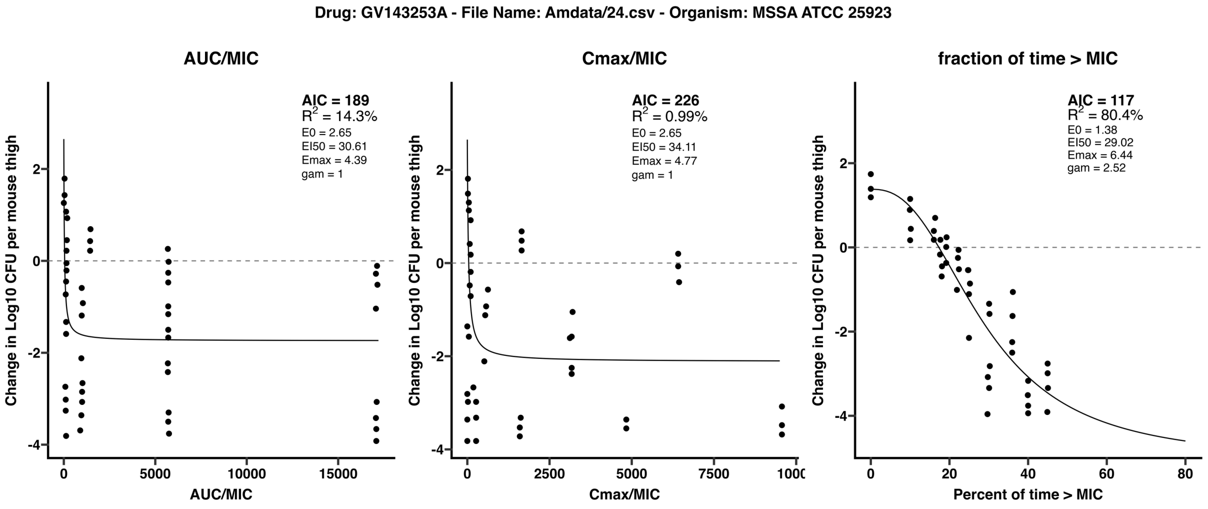

#	Model	Model Number	R²	AIC	EC50 hat	Emax hat	E0 hat	Gamma hat	Status	Best Fit Model	Optimal PKPD Index	Target for Stasis	Log1 Kill	Log2 Kill
1	AUC/MIC	1	0.12400	222	152.30	6.445 FIX	2.65 FIX	1 FIX	successful	3	-	-	-	-
2	AUC/MIC	2							unsuccessful	3	-	-	-	-
3	AUC/MIC	3	0.14300	189	30.61	4.391	2.65 FIX	1 FIX	successful	3	-	-	-	-
4	AUC/MIC	4							unsuccessful	3	-	-	-	-
5	AUC/MIC	5	0.14600	191	43.70	3.354	1.612	1 FIX	successful	3	-	-	-	-
6	AUC/MIC	6							unsuccessful	3	-	-	-	-
7	AUC/MIC	7	0.05830	194	77,790.00	6.445 FIX	-0.9988	1 FIX	successful	3	-	-	-	-
8	AUC/MIC	8							unsuccessful	3	-	-	-	-
9	Cmax/MIC	1	0.05380	235	117.10	6.445 FIX	2.65 FIX	1 FIX	successful	3	-	-	-	-
10	Cmax/MIC	2							unsuccessful	3	-	-	-	-
11	Cmax/MIC	3	0.00994	226	34.11	4.767	2.65 FIX	1 FIX	successful	3	-	-	-	-
12	Cmax/MIC	4							unsuccessful	3	-	-	-	-
13	Cmax/MIC	5	0.07400	197	487.20	1.291	-0.9387	1 FIX	successful	3	-	-	-	-
14	Cmax/MIC	6							unsuccessful	3	-	-	-	-
15	Cmax/MIC	7	0.06450	196	32,620.00	6.445 FIX	-1.248	1 FIX	successful	3	-	-	-	-
16	Cmax/MIC	8							unsuccessful	3	-	-	-	-
17	T>MIC	1	0.59400	151	15.46	6.445 FIX	2.65 FIX	1 FIX	successful	8	-	-	-	-
18	T>MIC	2	0.75700	130	19.62	6.445 FIX	2.65 FIX	2.293	successful	8	-	-	-	-
19	T>MIC	3	0.77200	125	144.90	26.26	2.65 FIX	1 FIX	successful	8	-	-	-	-
20	T>MIC	4							unsuccessful	8	-	-	-	-
21	T>MIC	5							unsuccessful	8	-	-	-	-
22	T>MIC	6	0.80600	119	25.44	5.074	1.164	3.472	successful	8	-	-	-	-
23	T>MIC	7	0.67800	148	27.14	6.445 FIX	1.717	1 FIX	successful	8	-	-	-	-
24	T>MIC	8	0.80400	117	29.02	6.445 FIX	1.38	2.516	successful	8	T>MIC	17	23	30

Wicha et al. (2019) PMID:30949706 Drug:Lefamulin

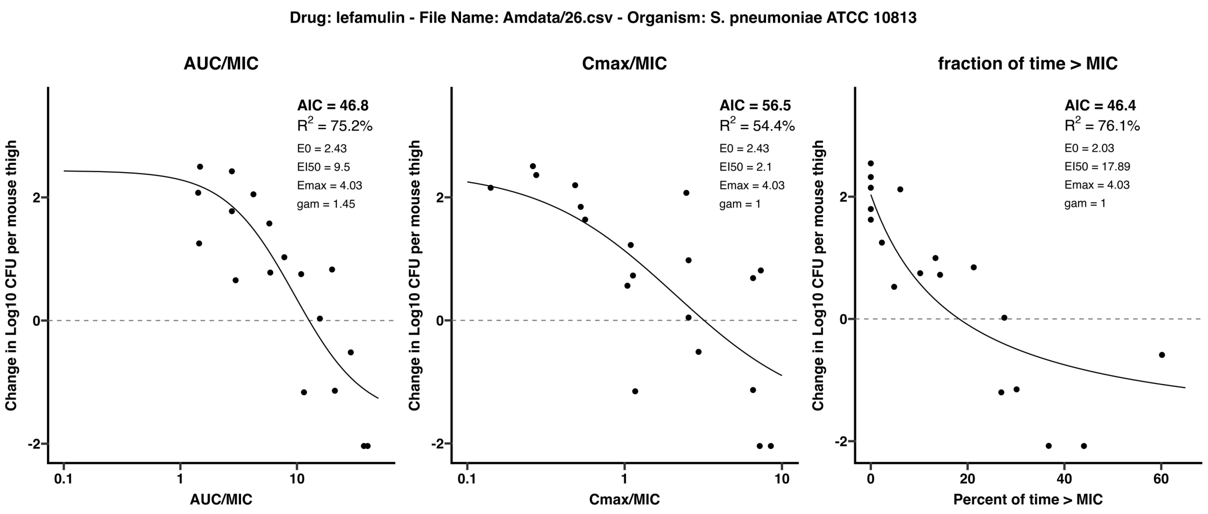

#	Model	Model Number	R²	AIC	EC50 hat	Emax hat	E0 hat	Gamma hat	Status	Best Fit Model	Optimal PKPD Index	Target for Stasis	Log1 Kill	Log2 Kill
1	AUC/MIC	1	0.750	47.3	9.503	4.029 FIX	2.434 FIX	1 FIX	successful	2	-	-	-	-
2	AUC/MIC	2	0.752	46.8	9.503	4.029 FIX	2.434 FIX	1.452	successful	2	-	-	-	-
3	AUC/MIC	3	0.781	44.3	27.400	7.02	2.434 FIX	1 FIX	successful	2	-	-	-	-
4	AUC/MIC	4	0.785	45.8	389.100	26.11	2.434 FIX	0.7102	successful	2	-	-	-	-
5	AUC/MIC	5	0.783	46.0	40.150	8.125	2.239	1 FIX	successful	2	-	-	-	-
6	AUC/MIC	6							unsuccessful	2	-	-	-	-
7	AUC/MIC	7	0.756	49.2	10.850	4.029 FIX	2.311	1 FIX	successful	2	-	-	-	-
8	AUC/MIC	8	0.770	47.3	14.160	4.029 FIX	1.981	1.676	successful	2	-	-	-	-
9	Cmax/MIC	1	0.544	56.5	2.095	4.029 FIX	2.434 FIX	1 FIX	successful	1	-	-	-	-
10	Cmax/MIC	2	0.544	58.5	2.096	4.029 FIX	2.434 FIX	0.9987	successful	1	-	-	-	-
11	Cmax/MIC	3	0.544	58.5	2.164	4.083	2.434 FIX	1 FIX	successful	1	-	-	-	-
12	Cmax/MIC	4	0.545	60.5	3.233	4.771	2.434 FIX	0.8603	successful	1	-	-	-	-
13	Cmax/MIC	5	0.547	60.3	1.330	4.151	2.859	1 FIX	successful	1	-	-	-	-
14	Cmax/MIC	6							unsuccessful	1	-	-	-	-
15	Cmax/MIC	7	0.547	58.3	1.348	4.029 FIX	2.784	1 FIX	successful	1	-	-	-	-
16	Cmax/MIC	8	0.547	60.3	1.348	4.029 FIX	2.784	0.9997	successful	1	-	-	-	-
17	T>MIC	1	0.740	46.4	11.610	4.029 FIX	2.434 FIX	1 FIX	successful	7	-	-	-	-
18	T>MIC	2	0.764	47.5	13.340	4.029 FIX	2.434 FIX	1.406	successful	7	-	-	-	-
19	T>MIC	3	0.765	47.0	20.730	5.105	2.434 FIX	1 FIX	successful	7	-	-	-	-
20	T>MIC	4	0.766	49.0	19.890	5.007	2.434 FIX	1.03	successful	7	-	-	-	-
21	T>MIC	5	0.768	47.5	28.470	5.111	2.075	1 FIX	successful	7	-	-	-	-
22	T>MIC	6	0.789	47.7	18.770	3.163	1.765	3.151	successful	7	-	-	-	-
23	T>MIC	7	0.761	46.4	17.890	4.029 FIX	2.034	1 FIX	successful	7	T>MIC	18	55	65
24	T>MIC	8	0.779	46.6	20.230	4.029 FIX	1.971	1.625	successful	7	-	-	-	-

Wicha et al. (2019) PMID:30949706 Drug:Lefamulin

Drug: lefamulin - File Name: Amdata/26.csv - Organism: S. aureus ATCC 25923

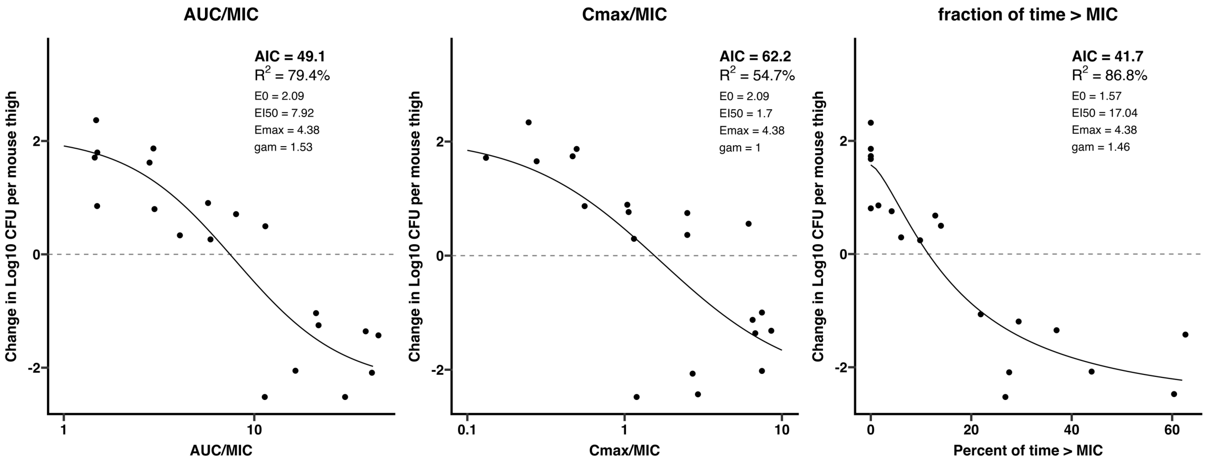

#	Model	Model Number	R²	AIC	EC50 hat	Emax hat	E0 hat	Gamma hat	Status	Best Fit Model	Optimal PKPD Index	Target for Stasis	Log1 Kill	Log2 Kill
1	AUC/MIC	1	0.784	50.9	8.0180	4.384 FIX	2.09 FIX	1 FIX	successful	2	-	-	-	-
2	AUC/MIC	2	0.794	49.1	7.9240	4.384 FIX	2.09 FIX	1.529	successful	2	-	-	-	-
3	AUC/MIC	3	0.776	51.0	12.2300	5.326	2.09 FIX	1 FIX	successful	2	-	-	-	-
4	AUC/MIC	4	0.795	50.9	7.2090	4.152	2.09 FIX	1.694	successful	2	-	-	-	-
5	AUC/MIC	5	0.784	51.9	7.4600	5.535	2.734	1 FIX	successful	2	-	-	-	-
6	AUC/MIC	6	0.799	52.5	7.9380	3.564	1.689	2.383	successful	2	-	-	-	-
7	AUC/MIC	7	0.784	52.9	7.5530	4.384 FIX	2.15	1 FIX	successful	2	-	-	-	-
8	AUC/MIC	8	0.794	51.1	7.6910	4.384 FIX	2.125	1.527	successful	2	-	-	-	-
9	Cmax/MIC	1	0.547	62.2	1.6990	4.384 FIX	2.09 FIX	1 FIX	successful	1	-	-	-	-
10	Cmax/MIC	2	0.547	64.2	1.7000	4.384 FIX	2.09 FIX	0.9911	successful	1	-	-	-	-
11	Cmax/MIC	3	0.558	63.9	1.3270	3.98	2.09 FIX	1 FIX	successful	1	-	-	-	-
12	Cmax/MIC	4	0.601	63.6	0.8533	3.151	2.09 FIX	2.902	successful	1	-	-	-	-
13	Cmax/MIC	5	0.572	64.9	0.6874	4.794	3.138	1 FIX	successful	1	-	-	-	-
14	Cmax/MIC	6	0.602	65.5	0.9102	2.93	1.896	3.654	successful	1	-	-	-	-
15	Cmax/MIC	7	0.572	63.1	0.7992	4.384 FIX	2.758	1 FIX	successful	1	-	-	-	-
16	Cmax/MIC	8							unsuccessful	1	-	-	-	-
17	T>MIC	1	0.834	44.2	9.2220	4.384 FIX	2.09 FIX	1 FIX	successful	8	-	-	-	-
18	T>MIC	2	0.852	45.3	10.7200	4.384 FIX	2.09 FIX	1.324	successful	8	-	-	-	-
19	T>MIC	3	0.861	44.0	16.6100	5.45	2.09 FIX	1 FIX	successful	8	-	-	-	-
20	T>MIC	4	0.858	45.8	25.1600	6.46	2.09 FIX	0.7981	successful	8	-	-	-	-
21	T>MIC	5	0.865	42.2	23.4800	5.4	1.653	1 FIX	successful	8	-	-	-	-
22	T>MIC	6							unsuccessful	8	-	-	-	-
23	T>MIC	7	0.858	41.8	15.1500	4.384 FIX	1.611	1 FIX	successful	8	-	-	-	-
24	T>MIC	8	0.868	41.7	17.0400	4.384 FIX	1.575	1.457	successful	8	T>MIC	11	22	47

Growcott et al. (2019) PMID:30325447 Drug:LYS228

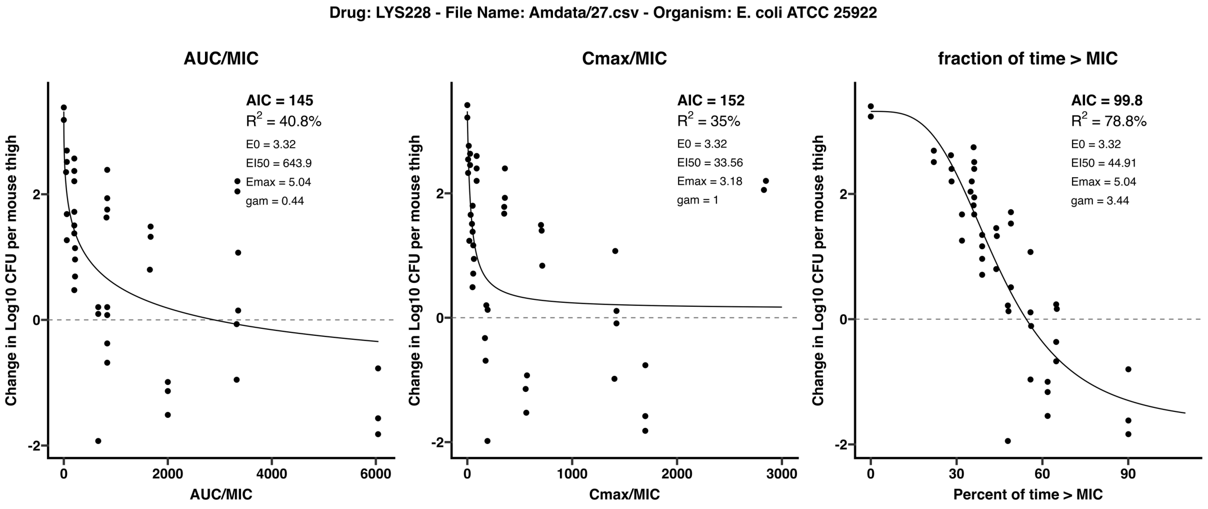

#	Model	Model Number	R²	AIC	EC50 hat	Emax hat	E0 hat	Gamma hat	Status	Best Fit Model	Optimal PKPD Index	Target for Stasis	Log1 Kill	Log2 Kill
1	AUC/MIC	1	0.372	158.0	713.30	5.038 FIX	3.318 FIX	1 FIX	successful	2	-	-	-	-
2	AUC/MIC	2	0.408	145.0	643.90	5.038 FIX	3.318 FIX	0.4374	successful	2	-	-	-	-
3	AUC/MIC	3	0.387	147.0	149.30	3.406	3.318 FIX	1 FIX	successful	2	-	-	-	-
4	AUC/MIC	4							unsuccessful	2	-	-	-	-
5	AUC/MIC	5	0.389	149.0	221.70	3.078	2.9	1 FIX	successful	2	-	-	-	-
6	AUC/MIC	6							unsuccessful	2	-	-	-	-
7	AUC/MIC	7	0.360	153.0	66.87	5.038 FIX	4.92	1 FIX	successful	2	-	-	-	-
8	AUC/MIC	8							unsuccessful	2	-	-	-	-
9	Cmax/MIC	1	0.290	172.0	204.10	5.038 FIX	3.318 FIX	1 FIX	successful	3	-	-	-	-
10	Cmax/MIC	2	0.315	155.0	243.10	5.038 FIX	3.318 FIX	0.33	successful	3	-	-	-	-
11	Cmax/MIC	3	0.350	152.0	33.56	3.182	3.318 FIX	1 FIX	successful	3	-	-	-	-
12	Cmax/MIC	4	0.350	154.0	33.59	3.194	3.318 FIX	0.9739	successful	3	-	-	-	-
13	Cmax/MIC	5	0.350	154.0	36.31	3.083	3.21	1 FIX	successful	3	-	-	-	-
14	Cmax/MIC	6	0.350	156.0	36.77	3.052	3.19	1.028	successful	3	-	-	-	-
15	Cmax/MIC	7	0.329	158.0	15.47	5.038 FIX	5.082	1 FIX	successful	3	-	-	-	-
16	Cmax/MIC	8							unsuccessful	3	-	-	-	-
17	T>MIC	1	0.619	136.0	44.59	5.038 FIX	3.318 FIX	1 FIX	successful	2	-	-	-	-
18	T>MIC	2	0.788	99.8	44.91	5.038 FIX	3.318 FIX	3.442	successful	2	T>MIC	54	76	110
19	T>MIC	3							unsuccessful	2	-	-	-	-
20	T>MIC	4							unsuccessful	2	-	-	-	-
21	T>MIC	5	0.750	110.0	1,011.00	70.21	3.92	1 FIX	successful	2	-	-	-	-
22	T>MIC	6							unsuccessful	2	-	-	-	-
23	T>MIC	7	0.626	138.0	46.54	5.038 FIX	3.26	1 FIX	successful	2	-	-	-	-
24	T>MIC	8	0.790	101.0	47.42	5.038 FIX	3.123	3.459	successful	2	-	-	-	-

Kristoffersson et al. (2016) PMID:26786016 Drug:Meropenem

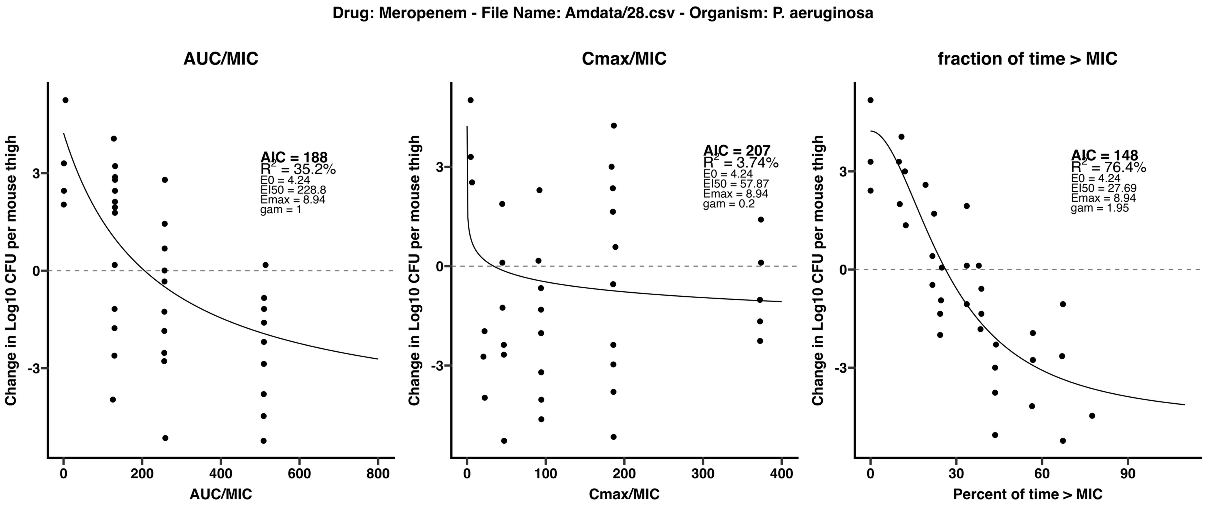

#	Model	Model Number	R²	AIC	EC50 hat	Emax hat	E0 hat	Gamma hat	Status	Best Fit Model	Optimal PKPD Index	Target for Stasis	Log1 Kill	Log2 Kill
1	AUC/MIC	1	0.3520	188	228.800	8.94 FIX	4.235 FIX	1 FIX	successful	1	-	-	-	-
2	AUC/MIC	2	0.3580	190	230.300	8.94 FIX	4.235 FIX	1.128	successful	1	-	-	-	-
3	AUC/MIC	3	0.3650	190	343.000	10.94	4.235 FIX	1 FIX	successful	1	-	-	-	-
4	AUC/MIC	4							unsuccessful	1	-	-	-	-
5	AUC/MIC	5	0.3730	190	921.100	15.28	3.034	1 FIX	successful	1	-	-	-	-
6	AUC/MIC	6							unsuccessful	1	-	-	-	-
7	AUC/MIC	7	0.3660	189	361.900	8.94 FIX	3.266	1 FIX	successful	1	-	-	-	-
8	AUC/MIC	8							unsuccessful	1	-	-	-	-
9	Cmax/MIC	1	0.0142	220	82.600	8.94 FIX	4.235 FIX	1 FIX	successful	2	-	-	-	-
10	Cmax/MIC	2	0.0374	207	57.870	8.94 FIX	4.235 FIX	0.196	successful	2	-	-	-	-
11	Cmax/MIC	3							unsuccessful	2	-	-	-	-
12	Cmax/MIC	4							unsuccessful	2	-	-	-	-
13	Cmax/MIC	5							unsuccessful	2	-	-	-	-
14	Cmax/MIC	6							unsuccessful	2	-	-	-	-
15	Cmax/MIC	7	0.1320	203	2.816	8.94 FIX	8.001	1 FIX	successful	2	-	-	-	-
16	Cmax/MIC	8							unsuccessful	2	-	-	-	-
17	T>MIC	1	0.6760	161	26.850	8.94 FIX	4.235 FIX	1 FIX	successful	2	-	-	-	-
18	T>MIC	2	0.7640	148	27.690	8.94 FIX	4.235 FIX	1.945	successful	2	T>MIC	26	33	43
19	T>MIC	3	0.7430	151	103.600	20.09	4.235 FIX	1 FIX	successful	2	-	-	-	-
20	T>MIC	4	0.7640	150	28.920	9.252	4.235 FIX	1.858	successful	2	-	-	-	-
21	T>MIC	5	0.7440	153	123.600	21.81	4.004	1 FIX	successful	2	-	-	-	-
22	T>MIC	6							unsuccessful	2	-	-	-	-
23	T>MIC	7	0.6970	163	32.910	8.94 FIX	3.766	1 FIX	successful	2	-	-	-	-
24	T>MIC	8	0.7690	149	33.200	8.94 FIX	3.588	1.986	successful	2	-	-	-	-

Fratoni et al. (2022) PMID:35134195 Drug:Minocycline

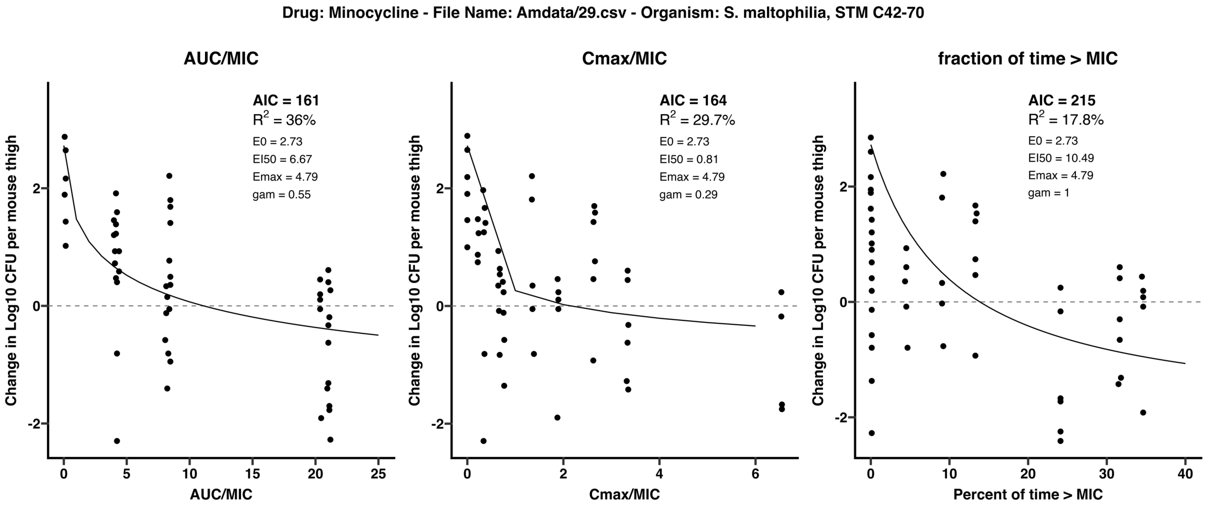

#	Model	Model Number	R²	AIC	EC50 hat	Emax hat	E0 hat	Gamma hat	Status	Best Fit Model	Optimal PKPD Index	Target for Stasis	Log1 Kill	Log2 Kill
1	AUC/MIC	1	0.363	164	7.49100	4.789 FIX	2.726 FIX	1 FIX	successful	2	-	-	-	-
2	AUC/MIC	2	0.360	161	6.67200	4.789 FIX	2.726 FIX	0.5472	successful	2	AUC/MIC	11	25	25
3	AUC/MIC	3	0.352	163	3.81400	3.765	2.726 FIX	1 FIX	successful	2	-	-	-	-
4	AUC/MIC	4							unsuccessful	2	-	-	-	-
5	AUC/MIC	5	0.363	162	8.14300	3.466	1.967	1 FIX	successful	2	-	-	-	-
6	AUC/MIC	6							unsuccessful	2	-	-	-	-
7	AUC/MIC	7	0.353	161	20.86000	4.789 FIX	1.708	1 FIX	successful	2	-	-	-	-
8	AUC/MIC	8	0.369	162	15.48000	4.789 FIX	2.127	0.6852	successful	2	-	-	-	-
9	Cmax/MIC	1	0.252	182	1.03200	4.789 FIX	2.726 FIX	1 FIX	successful	2	-	-	-	-
10	Cmax/MIC	2	0.297	164	0.81440	4.789 FIX	2.726 FIX	0.2886	successful	2	-	-	-	-
11	Cmax/MIC	3	0.289	165	0.13260	2.921	2.726 FIX	1 FIX	successful	2	-	-	-	-
12	Cmax/MIC	4							unsuccessful	2	-	-	-	-
13	Cmax/MIC	5	0.294	164	0.21190	2.246	2.017	1 FIX	successful	2	-	-	-	-
14	Cmax/MIC	6							unsuccessful	2	-	-	-	-
15	Cmax/MIC	7	0.203	168	9.21000	4.789 FIX	1.042	1 FIX	successful	2	-	-	-	-
16	Cmax/MIC	8							unsuccessful	2	-	-	-	-
17	T>MIC	1	0.178	215	10.49000	4.789 FIX	2.726 FIX	1 FIX	successful	1	-	-	-	-
18	T>MIC	2							unsuccessful	1	-	-	-	-
19	T>MIC	3	0.289	171	0.01656	2.797	2.726 FIX	1 FIX	successful	1	-	-	-	-
20	T>MIC	4							unsuccessful	1	-	-	-	-
21	T>MIC	5	0.198	178	91.49000	5.436	0.8369	1 FIX	successful	1	-	-	-	-
22	T>MIC	6							unsuccessful	1	-	-	-	-
23	T>MIC	7	0.198	176	77.11000	4.789 FIX	0.8405	1 FIX	successful	1	-	-	-	-
24	T>MIC	8	0.199	178	68.15000	4.789 FIX	0.8174	1.151	successful	1	-	-	-	-

Melchers et al. (2019) PMID:30642931 Drug:Murepavadin

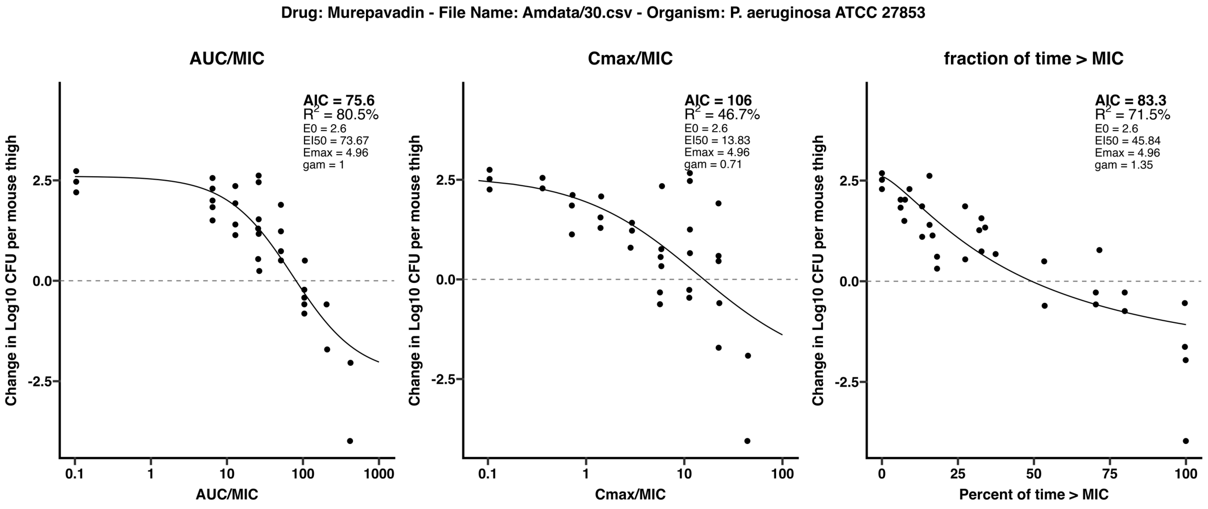

#	Model	Model Number	R²	AIC	EC50 hat	Emax hat	E0 hat	Gamma hat	Status	Best Fit Model	Optimal PKPD Index	Target for Stasis	Log1 Kill	Log2 Kill
1	AUC/MIC	1	0.805	75.6	73.67	4.964 FIX	2.601 FIX	1 FIX	successful	1	AUC/MIC	81.1	194.6	933.8
2	AUC/MIC	2	0.811	76.0	72.65	4.964 FIX	2.601 FIX	1.217	successful	1	-	-	-	-
3	AUC/MIC	3	0.849	69.0	162.30	7.442	2.601 FIX	1 FIX	successful	1	-	-	-	-
4	AUC/MIC	4	0.855	68.2	1,877.00	20.52	2.601 FIX	0.6481	successful	1	-	-	-	-
5	AUC/MIC	5	0.854	68.3	252.10	8.326	2.263	1 FIX	successful	1	-	-	-	-
6	AUC/MIC	6							unsuccessful	1	-	-	-	-
7	AUC/MIC	7	0.826	76.1	97.08	4.964 FIX	2.31	1 FIX	successful	1	-	-	-	-
8	AUC/MIC	8	0.840	71.7	114.50	4.964 FIX	2.008	1.591	successful	1	-	-	-	-
9	Cmax/MIC	1	0.463	106.0	13.32	4.964 FIX	2.601 FIX	1 FIX	successful	2	-	-	-	-
10	Cmax/MIC	2	0.467	106.0	13.83	4.964 FIX	2.601 FIX	0.7149	successful	2	-	-	-	-
11	Cmax/MIC	3	0.498	107.0	29.85	7.522	2.601 FIX	1 FIX	successful	2	-	-	-	-
12	Cmax/MIC	4							unsuccessful	2	-	-	-	-
13	Cmax/MIC	5							unsuccessful	2	-	-	-	-
14	Cmax/MIC	6							unsuccessful	2	-	-	-	-
15	Cmax/MIC	7	0.489	105.0	24.33	4.964 FIX	2.011	1 FIX	successful	2	-	-	-	-
16	Cmax/MIC	8	0.515	105.0	28.66	4.964 FIX	1.386	4.058	successful	2	-	-	-	-
17	T>MIC	1	0.699	83.7	46.86	4.964 FIX	2.601 FIX	1 FIX	successful	2	-	-	-	-
18	T>MIC	2	0.715	83.3	45.84	4.964 FIX	2.601 FIX	1.347	successful	2	-	-	-	-
19	T>MIC	3	0.764	78.0	310.50	17.37	2.601 FIX	1 FIX	successful	2	-	-	-	-
20	T>MIC	4							unsuccessful	2	-	-	-	-
21	T>MIC	5							unsuccessful	2	-	-	-	-
22	T>MIC	6							unsuccessful	2	-	-	-	-
23	T>MIC	7	0.707	85.5	52.44	4.964 FIX	2.472	1 FIX	successful	2	-	-	-	-
24	T>MIC	8	0.744	81.4	70.31	4.964 FIX	1.89	2.075	successful	2	-	-	-	-

Zhao et al. (2018) PMID:29987156 Drug:NOSO-502 (Odilorhabdin)

Drug: NOSO-502 - File Name: Amdata/32.csv - Organism: E. coli ATCC 25922

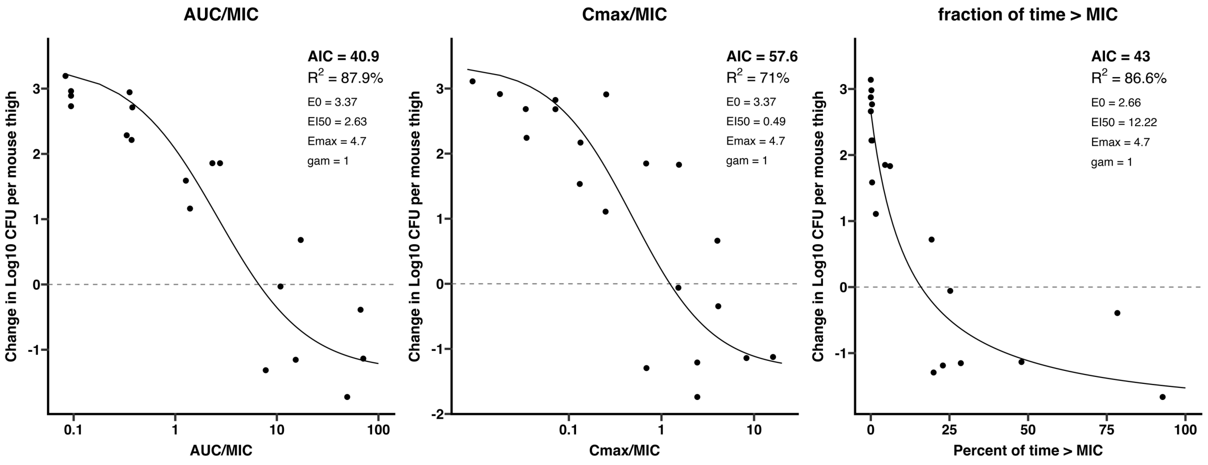

#	Model	Model Number	R²	AIC	EC50 hat	Emax hat	E0 hat	Gamma hat	Status	Best Fit Model	Optimal PKPD Index	Target for Stasis	Log1 Kill	Log2 Kill
1	AUC/MIC	1	0.879	40.9	2.6280	4.702 FIX	3.37 FIX	1 FIX	successful	1	AUC/MIC	6.68	34.58	99.98
2	AUC/MIC	2	0.878	41.5	2.6070	4.702 FIX	3.37 FIX	0.811	successful	1	-	-	-	-
3	AUC/MIC	3	0.877	42.5	2.3060	4.512	3.37 FIX	1 FIX	successful	1	-	-	-	-
4	AUC/MIC	4	0.879	43.3	3.0140	4.898	3.37 FIX	0.7576	successful	1	-	-	-	-
5	AUC/MIC	5	0.881	42.8	3.2720	4.27	3.009	1 FIX	successful	1	-	-	-	-
6	AUC/MIC	6	0.881	44.8	3.2640	4.206	2.98	1.04	successful	1	-	-	-	-
7	AUC/MIC	7	0.881	42.3	3.5600	4.702 FIX	3.157	1 FIX	successful	1	-	-	-	-
8	AUC/MIC	8							unsuccessful	1	-	-	-	-
9	Cmax/MIC	1	0.710	57.6	0.4883	4.702 FIX	3.37 FIX	1 FIX	successful	1	-	-	-	-
10	Cmax/MIC	2	0.710	58.3	0.4785	4.702 FIX	3.37 FIX	0.756	successful	1	-	-	-	-
11	Cmax/MIC	3	0.705	58.9	0.3554	4.277	3.37 FIX	1 FIX	successful	1	-	-	-	-
12	Cmax/MIC	4	0.710	60.2	0.5514	4.892	3.37 FIX	0.714	successful	1	-	-	-	-
13	Cmax/MIC	5	0.711	60.2	0.5471	4.048	2.963	1 FIX	successful	1	-	-	-	-
14	Cmax/MIC	6	0.711	62.2	0.5666	4.328	3.076	0.8752	successful	1	-	-	-	-
15	Cmax/MIC	7	0.708	59.3	0.7335	4.702 FIX	3.089	1 FIX	successful	1	-	-	-	-
16	Cmax/MIC	8	0.711	60.2	0.5999	4.702 FIX	3.221	0.7647	successful	1	-	-	-	-
17	T>MIC	1	0.850	49.4	4.7030	4.702 FIX	3.37 FIX	1 FIX	successful	7	-	-	-	-
18	T>MIC	2	0.839	48.1	3.3430	4.702 FIX	3.37 FIX	0.6741	successful	7	-	-	-	-
19	T>MIC	3	0.847	51.4	4.4010	4.637	3.37 FIX	1 FIX	successful	7	-	-	-	-
20	T>MIC	4	0.857	47.7	29.6500	7.753	3.37 FIX	0.4393	successful	7	-	-	-	-
21	T>MIC	5	0.867	44.9	10.6000	4.462	2.651	1 FIX	successful	7	-	-	-	-
22	T>MIC	6	0.868	46.7	12.8500	5.165	2.783	0.7383	successful	7	-	-	-	-
23	T>MIC	7	0.866	43.0	12.2200	4.702 FIX	2.663	1 FIX	successful	7	-	-	-	-
24	T>MIC	8							unsuccessful	7	-	-	-	-

Andes et al. (2009) PMID:19414576 Drug:NZ2114 (Plectasin)

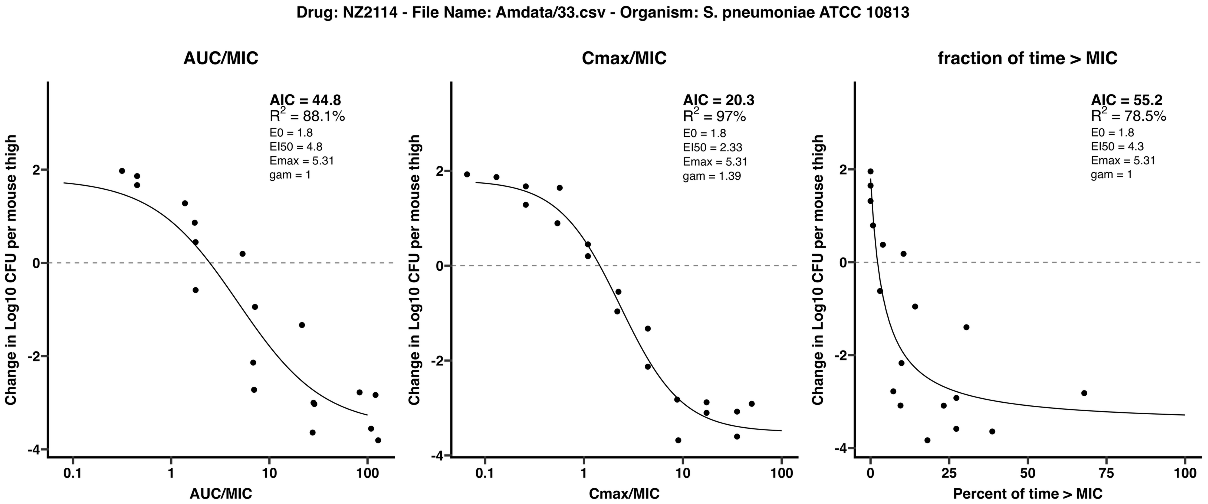

#	Model	Model Number	R²	AIC	EC50 hat	Emax hat	E0 hat	Gamma hat	Status	Best Fit Model	Optimal PKPD Index	Target for Stasis	Log1 Kill	Log2 Kill
1	AUC/MIC	1	0.881	44.8	4.800	5.31 FIX	1.803 FIX	1 FIX	successful	1	-	-	-	-
2	AUC/MIC	2	0.881	46.1	4.769	5.31 FIX	1.803 FIX	1.165	successful	1	-	-	-	-
3	AUC/MIC	3	0.880	46.7	5.044	5.407	1.803 FIX	1 FIX	successful	1	-	-	-	-
4	AUC/MIC	4	0.882	47.9	4.371	5.13	1.803 FIX	1.256	successful	1	-	-	-	-
5	AUC/MIC	5	0.885	47.2	3.530	5.92	2.445	1 FIX	successful	1	-	-	-	-
6	AUC/MIC	6	0.885	49.2	3.499	5.976	2.483	0.982	successful	1	-	-	-	-
7	AUC/MIC	7	0.884	46.5	4.104	5.31 FIX	1.964	1 FIX	successful	1	-	-	-	-
8	AUC/MIC	8							unsuccessful	1	-	-	-	-
9	Cmax/MIC	1	0.963	27.6	2.323	5.31 FIX	1.803 FIX	1 FIX	successful	2	-	-	-	-
10	Cmax/MIC	2	0.970	20.3	2.330	5.31 FIX	1.803 FIX	1.393	successful	2	Cmax/MIC	1.45	2.52	4.53
11	Cmax/MIC	3	0.960	27.7	2.701	5.634	1.803 FIX	1 FIX	successful	2	-	-	-	-
12	Cmax/MIC	4	0.971	21.6	2.185	5.144	1.803 FIX	1.489	successful	2	-	-	-	-
13	Cmax/MIC	5	0.963	25.8	2.085	5.989	2.27	1 FIX	successful	2	-	-	-	-
14	Cmax/MIC	6	0.971	23.6	2.172	5.17	1.824	1.474	successful	2	-	-	-	-
15	Cmax/MIC	7	0.963	29.3	2.145	5.31 FIX	1.89	1 FIX	successful	2	-	-	-	-
16	Cmax/MIC	8	0.971	21.7	2.155	5.31 FIX	1.902	1.395	successful	2	-	-	-	-
17	T>MIC	1	0.785	55.2	4.301	5.31 FIX	1.803 FIX	1 FIX	successful	1	-	-	-	-
18	T>MIC	2	0.785	57.2	4.406	5.31 FIX	1.803 FIX	1.038	successful	1	-	-	-	-
19	T>MIC	3	0.785	57.2	4.453	5.362	1.803 FIX	1 FIX	successful	1	-	-	-	-
20	T>MIC	4	0.785	59.2	4.355	5.279	1.803 FIX	1.053	successful	1	-	-	-	-
21	T>MIC	5	0.785	59.0	4.811	5.237	1.643	1 FIX	successful	1	-	-	-	-
22	T>MIC	6	0.786	61.0	4.609	5.008	1.613	1.142	successful	1	-	-	-	-
23	T>MIC	7	0.785	57.0	4.943	5.31 FIX	1.664	1 FIX	successful	1	-	-	-	-
24	T>MIC	8							unsuccessful	1	-	-	-	-

Andes et al. (2009) PMID:19414576 Drug:NZ2114 (Plectasin)

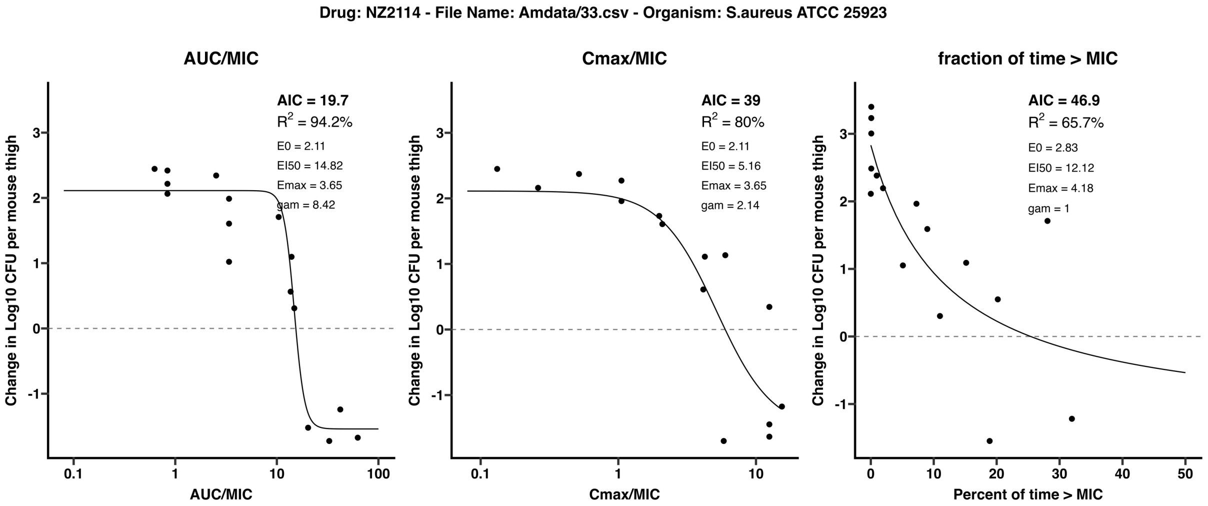

#	Model	Model Number	R²	AIC	EC50 hat	Emax hat	E0 hat	Gamma hat	Status	Best Fit Model	Optimal PKPD Index	Target for Stasis	Log1 Kill	Log2 Kill
1	AUC/MIC	1	0.847	39.6	13.710	3.652 FIX	2.112 FIX	1 FIX	successful	2	-	-	-	-
2	AUC/MIC	2	0.942	19.7	14.820	3.652 FIX	2.112 FIX	8.418	successful	2	AUC/MIC	15.39	18.25	100
3	AUC/MIC	3	0.864	34.2	45.410	7.302	2.112 FIX	1 FIX	successful	2	-	-	-	-
4	AUC/MIC	4	0.943	21.7	14.890	3.709	2.112 FIX	8.171	successful	2	-	-	-	-
5	AUC/MIC	5	0.870	34.0	29.460	6.687	2.494	1 FIX	successful	2	-	-	-	-
6	AUC/MIC	6							unsuccessful	2	-	-	-	-
7	AUC/MIC	7	0.833	41.2	11.180	3.652 FIX	2.296	1 FIX	successful	2	-	-	-	-
8	AUC/MIC	8	0.943	20.9	15.040	3.652 FIX	2.015	8.511	successful	2	-	-	-	-
9	Cmax/MIC	1	0.788	42.9	5.429	3.652 FIX	2.112 FIX	1 FIX	successful	2	-	-	-	-
10	Cmax/MIC	2	0.800	39.0	5.156	3.652 FIX	2.112 FIX	2.138	successful	2	-	-	-	-
11	Cmax/MIC	3	0.781	41.4	20.520	8.054	2.112 FIX	1 FIX	successful	2	-	-	-	-
12	Cmax/MIC	4	0.801	40.7	4.519	3.25	2.112 FIX	2.884	successful	2	-	-	-	-
13	Cmax/MIC	5	0.793	41.1	8.535	6.09	2.662	1 FIX	successful	2	-	-	-	-
14	Cmax/MIC	6	0.804	42.3	4.421	3.64	2.323	2.162	successful	2	-	-	-	-
15	Cmax/MIC	7	0.772	43.9	3.660	3.652 FIX	2.47	1 FIX	successful	2	-	-	-	-
16	Cmax/MIC	8	0.804	40.3	4.429	3.652 FIX	2.326	2.146	successful	2	-	-	-	-
17	T>MIC	1	0.652	47.0	20.940	3.652 FIX	2.112 FIX	1 FIX	successful	5	-	-	-	-
18	T>MIC	2	0.631	48.7	19.040	3.652 FIX	2.112 FIX	1.364	successful	5	-	-	-	-
19	T>MIC	3	0.637	48.9	38.880	5.348	2.112 FIX	1 FIX	successful	5	-	-	-	-
20	T>MIC	4							unsuccessful	5	-	-	-	-
21	T>MIC	5	0.657	46.9	12.120	4.182	2.831	1 FIX	successful	5	-	-	-	-
22	T>MIC	6	0.657	48.9	12.030	4.167	2.83	1.004	successful	5	-	-	-	-
23	T>MIC	7	0.655	45.0	8.840	3.652 FIX	2.828	1 FIX	successful	5	-	-	-	-
24	T>MIC	8	0.657	46.9	9.409	3.652 FIX	2.802	1.157	successful	5	-	-	-	-

Umezaki et al. (2022) PMID:35884236 Drug:Pazufloxacin

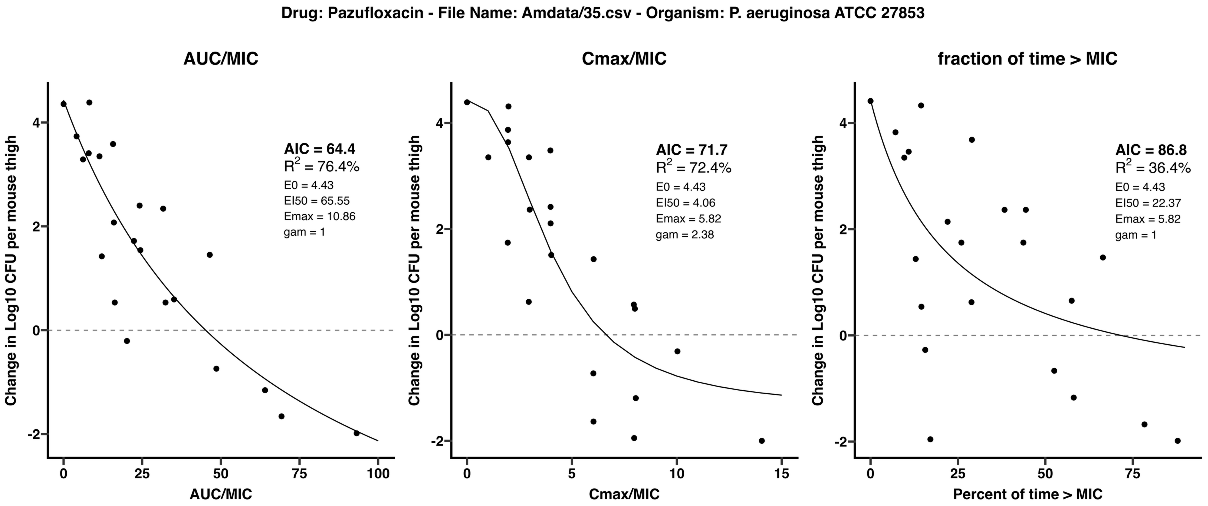

#	Model	Model Number	R²	AIC	EC50 hat	Emax hat	E0 hat	Gamma hat	Status	Best Fit Model	Optimal PKPD Index	Target for Stasis	Log1 Kill	Log2 Kill
1	AUC/MIC	1	0.722	69.3	21.020	5.815 FIX	4.43 FIX	1 FIX	successful	3	-	-	-	-
2	AUC/MIC	2	0.731	67.5	21.570	5.815 FIX	4.43 FIX	1.555	successful	3	-	-	-	-
3	AUC/MIC	3	0.764	64.4	65.550	10.86	4.43 FIX	1 FIX	successful	3	AUC/MIC	45	66	95
4	AUC/MIC	4	0.765	66.3	177.100	17.66	4.43 FIX	0.8195	successful	3	-	-	-	-
5	AUC/MIC	5	0.764	66.4	73.970	11.31	4.31	1 FIX	successful	3	-	-	-	-
6	AUC/MIC	6	0.766	68.3	206.000	19.05	4.51	0.788	successful	3	-	-	-	-
7	AUC/MIC	7	0.735	71.1	25.010	5.815 FIX	4.181	1 FIX	successful	3	-	-	-	-
8	AUC/MIC	8	0.743	68.6	29.480	5.815 FIX	3.843	1.655	successful	3	-	-	-	-
9	Cmax/MIC	1	0.661	78.7	3.710	5.815 FIX	4.43 FIX	1 FIX	successful	2	-	-	-	-
10	Cmax/MIC	2	0.724	71.7	4.055	5.815 FIX	4.43 FIX	2.381	successful	2	-	-	-	-
11	Cmax/MIC	3	0.719	71.9	20.450	16.74	4.43 FIX	1 FIX	successful	2	-	-	-	-
12	Cmax/MIC	4	0.733	72.7	5.524	7.54	4.43 FIX	1.727	successful	2	-	-	-	-
13	Cmax/MIC	5	0.721	73.6	14.570	14.45	4.794	1 FIX	successful	2	-	-	-	-
14	Cmax/MIC	6	0.736	74.4	5.155	6.059	3.897	2.386	successful	2	-	-	-	-
15	Cmax/MIC	7							unsuccessful	2	-	-	-	-
16	Cmax/MIC	8							unsuccessful	2	-	-	-	-
17	T>MIC	1	0.364	86.8	22.370	5.815 FIX	4.43 FIX	1 FIX	successful	1	-	-	-	-
18	T>MIC	2	0.364	88.8	22.330	5.815 FIX	4.43 FIX	0.9884	successful	1	-	-	-	-
19	T>MIC	3	0.367	88.7	29.180	6.513	4.43 FIX	1 FIX	successful	1	-	-	-	-
20	T>MIC	4							unsuccessful	1	-	-	-	-
21	T>MIC	5							unsuccessful	1	-	-	-	-
22	T>MIC	6							unsuccessful	1	-	-	-	-
23	T>MIC	7	0.367	88.7	30.650	5.815 FIX	4.024	1 FIX	successful	1	-	-	-	-
24	T>MIC	8	0.367	90.7	30.790	5.815 FIX	4.018	1.004	successful	1	-	-	-	-

Lepak et al. (2020) PMID:32868332 Drug:Polymyxin

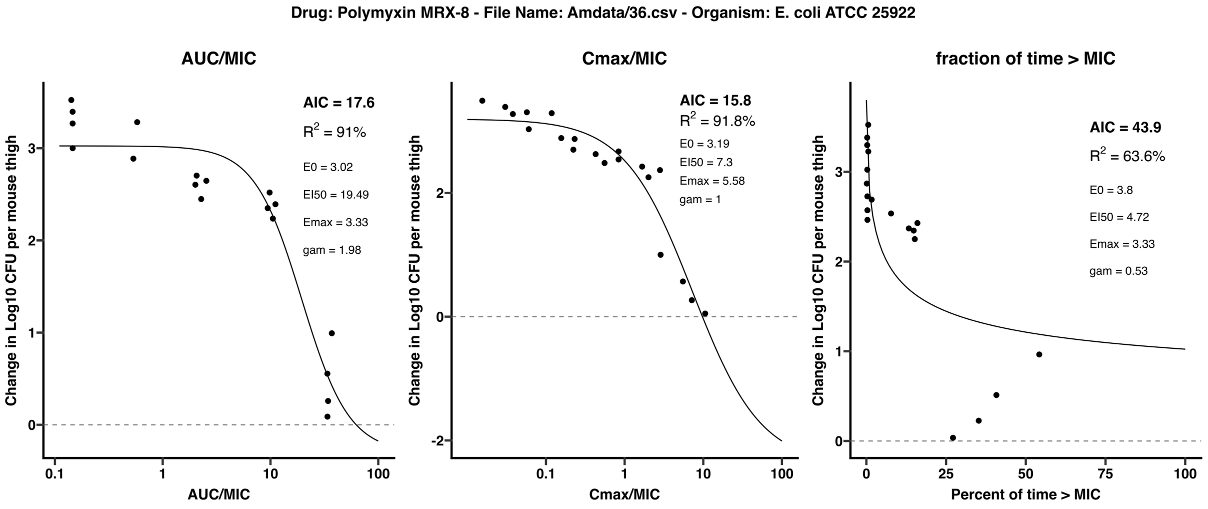

#	Model	Model Number	R²	AIC	EC50 hat	Emax hat	E0 hat	Gamma hat	Status	Best Fit Model	Optimal PKPD Index	Target for Stasis	Log1 Kill	Log2 Kill
1	AUC/MIC	1	0.793	34.9	5.8350	3.327 FIX	3.8 FIX	1 FIX	successful	8	-	-	-	-
2	AUC/MIC	2	0.781	34.0	5.0710	3.327 FIX	3.8 FIX	0.705	successful	8	-	-	-	-
3	AUC/MIC	3	0.849	36.0	11.0400	4.119	3.8 FIX	1 FIX	successful	8	-	-	-	-
4	AUC/MIC	4							unsuccessful	8	-	-	-	-
5	AUC/MIC	5	0.918	15.6	138.3000	13	3.121	1 FIX	successful	8	-	-	-	-
6	AUC/MIC	6							unsuccessful	8	-	-	-	-
7	AUC/MIC	7	0.875	23.7	16.0100	3.327 FIX	3.189	1 FIX	successful	8	-	-	-	-
8	AUC/MIC	8	0.910	17.6	19.4900	3.327 FIX	3.025	1.978	successful	8	-	-	-	-
9	Cmax/MIC	1	0.811	32.0	1.0700	3.327 FIX	3.8 FIX	1 FIX	successful	5	-	-	-	-
10	Cmax/MIC	2	0.805	31.9	0.9846	3.327 FIX	3.8 FIX	0.758	successful	5	-	-	-	-
11	Cmax/MIC	3	0.866	32.3	1.9450	4.137	3.8 FIX	1 FIX	successful	5	-	-	-	-
12	Cmax/MIC	4							unsuccessful	5	-	-	-	-
13	Cmax/MIC	5	0.918	15.8	7.2960	5.581	3.193	1 FIX	successful	5	Cmax/MIC	9.76	22.04	97.65
14	Cmax/MIC	6							unsuccessful	5	-	-	-	-
15	Cmax/MIC	7	0.890	21.7	2.7300	3.327 FIX	3.223	1 FIX	successful	5	-	-	-	-
16	Cmax/MIC	8	0.906	18.7	3.2810	3.327 FIX	3.053	1.701	successful	5	-	-	-	-
17	T>MIC	1	0.682	47.3	8.2930	3.327 FIX	3.8 FIX	1 FIX	successful	2	-	-	-	-
18	T>MIC	2	0.636	43.9	4.7220	3.327 FIX	3.8 FIX	0.53	successful	2	-	-	-	-
19	T>MIC	3	0.534	49.6	1.1720	2.47	3.8 FIX	1 FIX	successful	2	-	-	-	-
20	T>MIC	4							unsuccessful	2	-	-	-	-
21	T>MIC	5	0.789	34.9	65.7800	6.322	3.093	1 FIX	successful	2	-	-	-	-
22	T>MIC	6							unsuccessful	2	-	-	-	-
23	T>MIC	7	0.762	35.8	23.3300	3.327 FIX	3.083	1 FIX	successful	2	-	-	-	-
24	T>MIC	8	0.847	29.6	21.5500	3.327 FIX	3.059	2.756	successful	2	-	-	-	-

Andes and Craig (2006) PMID:16569855 Drug:PPI-0903 (TAK-599)

Drug: PPI-0903 (TAK-599) - File Name: Amdata/37.csv - Organism: S. pneumoniae ATCC 10813

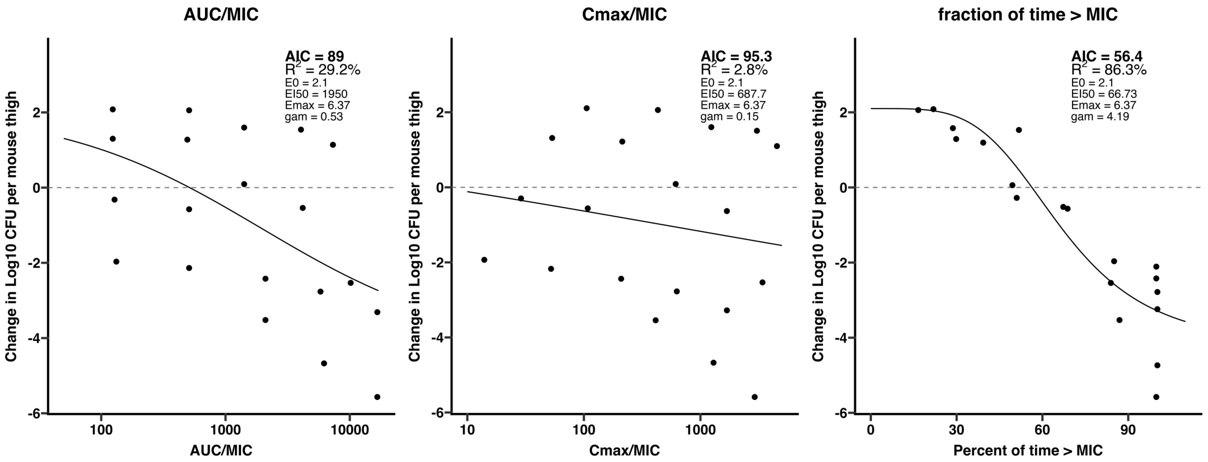

#	Model	Model Number	R²	AIC	EC50 hat	Emax hat	E0 hat	Gamma hat	Status	Best Fit Model	Optimal PKPD Index	Target for Stasis	Log1 Kill	Log2 Kill
1	AUC/MIC	1	0.299000	89.3	2,308.0000	6.368 FIX	2.1 FIX	1 FIX	successful	2	-	-	-	-
2	AUC/MIC	2	0.292000	89.0	1,950.0000	6.368 FIX	2.1 FIX	0.5322	successful	2	-	-	-	-
3	AUC/MIC	3	0.258000	90.7	947.2000	5.008	2.1 FIX	1 FIX	successful	2	-	-	-	-
4	AUC/MIC	4							unsuccessful	2	-	-	-	-
5	AUC/MIC	5	0.361000	88.9	104,900.0000	32.91	0.1914	1 FIX	successful	2	-	-	-	-
6	AUC/MIC	6							unsuccessful	2	-	-	-	-
7	AUC/MIC	7	0.164000	92.4	107.7000	6.368 FIX	4.481	1 FIX	successful	2	-	-	-	-
8	AUC/MIC	8							unsuccessful	2	-	-	-	-
9	Cmax/MIC	1	0.043300	99.9	675.5000	6.368 FIX	2.1 FIX	1 FIX	successful	2	-	-	-	-
10	Cmax/MIC	2	0.028000	95.3	687.7000	6.368 FIX	2.1 FIX	0.1485	successful	2	-	-	-	-
11	Cmax/MIC	3	0.000203	95.9	0.8770	3.095	2.1 FIX	1 FIX	successful	2	-	-	-	-
12	Cmax/MIC	4							unsuccessful	2	-	-	-	-
13	Cmax/MIC	5	0.017800	97.5	-951.3000	0.1885	-0.8587	1 FIX	successful	2	-	-	-	-
14	Cmax/MIC	6	0.058300	98.7	498.7000	1.243	-0.3446	3.715	successful	2	-	-	-	-
15	Cmax/MIC	7	0.000128	95.9	0.2789	6.368 FIX	5.381	1 FIX	successful	2	-	-	-	-
16	Cmax/MIC	8							unsuccessful	2	-	-	-	-
17	T>MIC	1	0.800000	79.4	61.8800	6.368 FIX	2.1 FIX	1 FIX	successful	2	-	-	-	-
18	T>MIC	2	0.863000	56.4	66.7300	6.368 FIX	2.1 FIX	4.188	successful	2	T>MIC	56	66	77
19	T>MIC	3							unsuccessful	2	-	-	-	-
20	T>MIC	4	0.873000	56.6	104.3000	11.93	2.1 FIX	2.561	successful	2	-	-	-	-
21	T>MIC	5							unsuccessful	2	-	-	-	-
22	T>MIC	6	0.873000	58.6	96.1400	10.52	2.003	2.797	successful	2	-	-	-	-
23	T>MIC	7							unsuccessful	2	-	-	-	-
24	T>MIC	8	0.870000	57.1	73.8500	6.368 FIX	1.644	4.561	successful	2	-	-	-	-

Andes and Craig (2006) PMID:16569855 Drug:PPI-0903 (TAK-599)

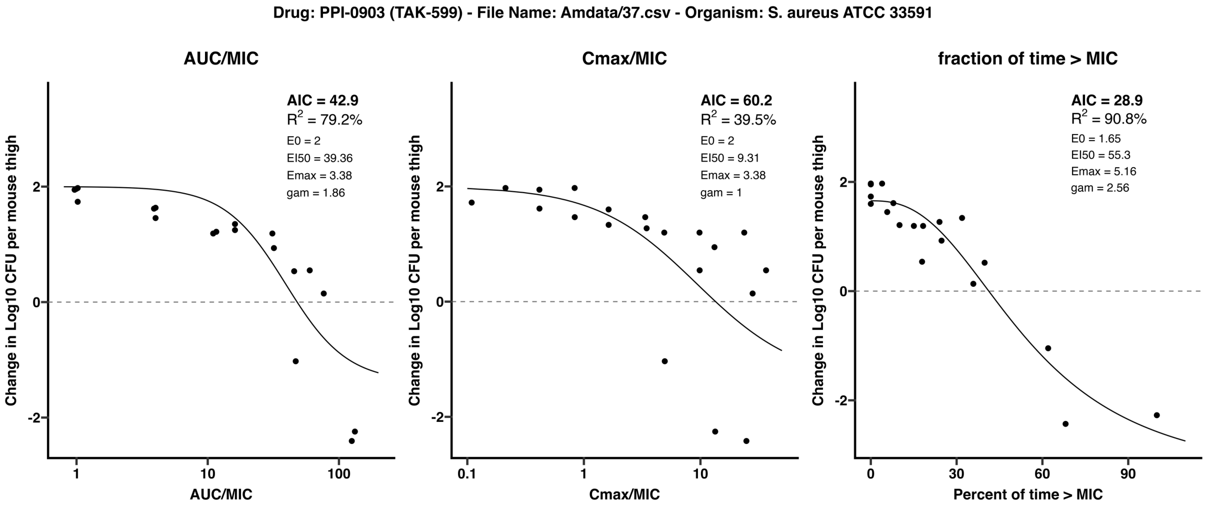

#	Model	Model Number	R²	AIC	EC50 hat	Emax hat	E0 hat	Gamma hat	Status	Best Fit Model	Optimal PKPD Index	Target for Stasis	Log1 Kill	Log2 Kill
1	AUC/MIC	1	0.753	44.6	35.310	3.381 FIX	2 FIX	1 FIX	successful	2	-	-	-	-
2	AUC/MIC	2	0.792	42.9	39.360	3.381 FIX	2 FIX	1.86	successful	2	-	-	-	-
3	AUC/MIC	3	0.893	29.2	788.800	29.69	2 FIX	1 FIX	successful	2	-	-	-	-
4	AUC/MIC	4							unsuccessful	2	-	-	-	-
5	AUC/MIC	5							unsuccessful	2	-	-	-	-
6	AUC/MIC	6							unsuccessful	2	-	-	-	-
7	AUC/MIC	7	0.771	46.3	41.790	3.381 FIX	1.866	1 FIX	successful	2	-	-	-	-
8	AUC/MIC	8	0.837	39.6	55.940	3.381 FIX	1.569	2.625	successful	2	-	-	-	-
9	Cmax/MIC	1	0.395	60.2	9.314	3.381 FIX	2 FIX	1 FIX	successful	1	-	-	-	-
10	Cmax/MIC	2	0.396	61.9	9.747	3.381 FIX	2 FIX	0.797	successful	1	-	-	-	-
11	Cmax/MIC	3	0.406	61.6	4.982	2.646	2 FIX	1 FIX	successful	1	-	-	-	-
12	Cmax/MIC	4	0.414	63.4	3.762	2.291	2 FIX	1.552	successful	1	-	-	-	-
13	Cmax/MIC	5	0.406	63.6	4.688	2.67	2.044	1 FIX	successful	1	-	-	-	-
14	Cmax/MIC	6	0.417	65.2	4.083	1.978	1.797	2.245	successful	1	-	-	-	-
15	Cmax/MIC	7	0.397	62.2	8.782	3.381 FIX	2.036	1 FIX	successful	1	-	-	-	-
16	Cmax/MIC	8	0.399	63.8	6.660	3.381 FIX	2.21	0.7528	successful	1	-	-	-	-
17	T>MIC	1	0.708	48.3	30.040	3.381 FIX	2 FIX	1 FIX	successful	6	-	-	-	-
18	T>MIC	2	0.828	41.2	32.790	3.381 FIX	2 FIX	2.721	successful	6	-	-	-	-
19	T>MIC	3	0.893	28.4	1,766.000	87.64	2 FIX	1 FIX	successful	6	-	-	-	-
20	T>MIC	4							unsuccessful	6	-	-	-	-
21	T>MIC	5							unsuccessful	6	-	-	-	-
22	T>MIC	6	0.908	28.9	55.300	5.157	1.654	2.558	successful	6	T>MIC	41	57	78
23	T>MIC	7							unsuccessful	6	-	-	-	-
24	T>MIC	8	0.894	31.1	43.650	3.381 FIX	1.46	5.397	successful	6	-	-	-	-

Andes and Craig (2006) PMID:16569855 Drug:PPI-0903 (TAK-599)

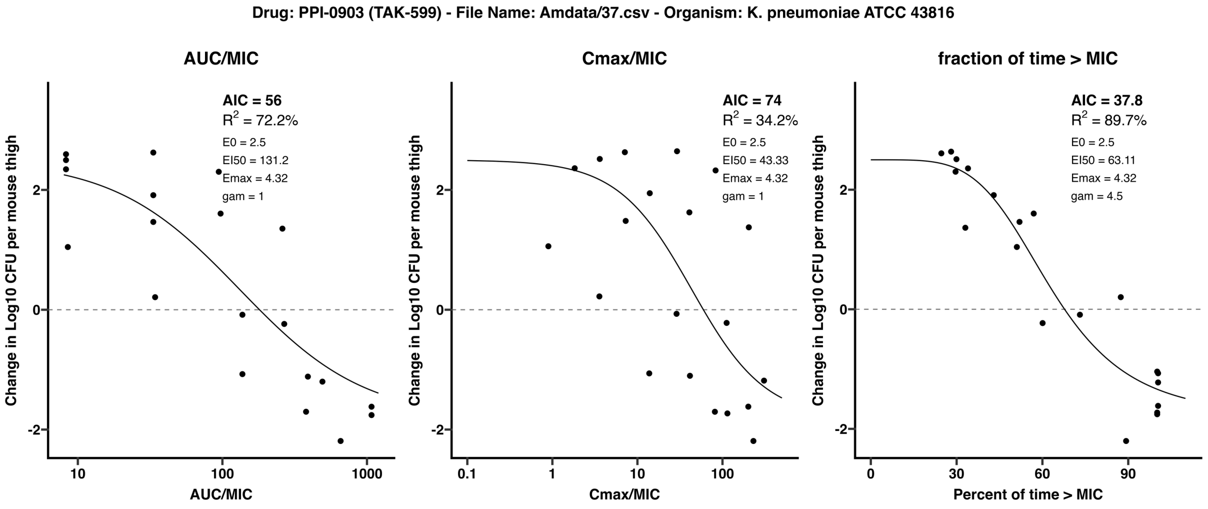

#	Model	Model Number	R²	AIC	EC50 hat	Emax hat	E0 hat	Gamma hat	Status	Best Fit Model	Optimal PKPD Index	Target for Stasis	Log1 Kill	Log2 Kill
1	AUC/MIC	1	0.722	56.0	131.20	4.317 FIX	2.5 FIX	1 FIX	successful	1	-	-	-	-
2	AUC/MIC	2	0.728	57.4	138.60	4.317 FIX	2.5 FIX	1.26	successful	1	-	-	-	-
3	AUC/MIC	3	0.740	56.3	224.30	5.378	2.5 FIX	1 FIX	successful	1	-	-	-	-
4	AUC/MIC	4	0.739	58.2	277.60	5.854	2.5 FIX	0.8868	successful	1	-	-	-	-
5	AUC/MIC	5	0.742	58.0	279.10	5.398	2.277	1 FIX	successful	1	-	-	-	-
6	AUC/MIC	6	0.744	59.8	225.00	4.452	2.067	1.384	successful	1	-	-	-	-
7	AUC/MIC	7	0.736	57.4	184.90	4.317 FIX	2.189	1 FIX	successful	1	-	-	-	-
8	AUC/MIC	8	0.744	57.8	218.40	4.317 FIX	2.038	1.454	successful	1	-	-	-	-
9	Cmax/MIC	1	0.342	74.0	43.33	4.317 FIX	2.5 FIX	1 FIX	successful	1	-	-	-	-
10	Cmax/MIC	2	0.333	75.2	40.04	4.317 FIX	2.5 FIX	0.6662	successful	1	-	-	-	-
11	Cmax/MIC	3	0.329	75.7	26.98	3.716	2.5 FIX	1 FIX	successful	1	-	-	-	-
12	Cmax/MIC	4							unsuccessful	1	-	-	-	-
13	Cmax/MIC	5	0.349	76.6	89.61	3.792	1.716	1 FIX	successful	1	-	-	-	-
14	Cmax/MIC	6	0.349	78.6	87.96	3.75	1.71	1.014	successful	1	-	-	-	-
15	Cmax/MIC	7	0.348	74.7	125.80	4.317 FIX	1.67	1 FIX	successful	1	-	-	-	-
16	Cmax/MIC	8							unsuccessful	1	-	-	-	-
17	T>MIC	1	0.880	67.6	62.60	4.317 FIX	2.5 FIX	1 FIX	successful	2	-	-	-	-
18	T>MIC	2	0.897	37.8	63.11	4.317 FIX	2.5 FIX	4.505	successful	2	T>MIC	68	87	110
19	T>MIC	3							unsuccessful	2	-	-	-	-
20	T>MIC	4	0.898	39.5	68.39	4.841	2.5 FIX	3.759	successful	2	-	-	-	-
21	T>MIC	5	0.898	39.6	1,181.00	71.06	4.107	1 FIX	successful	2	-	-	-	-
22	T>MIC	6	0.899	41.3	74.42	5.988	2.762	2.841	successful	2	-	-	-	-
23	T>MIC	7							unsuccessful	2	-	-	-	-
24	T>MIC	8	0.897	39.7	64.46	4.317 FIX	2.434	4.509	successful	2	-	-	-	-

Hirai et al. (2016) PMID:27029221 Drug:Rifampicin

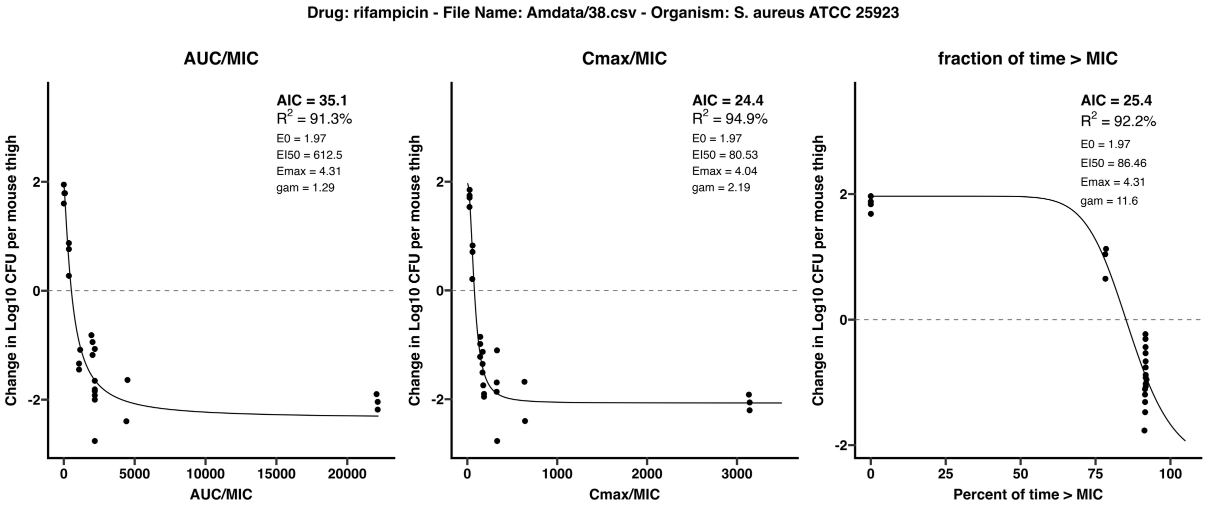

#	Model	Model Number	R²	AIC	EC50 hat	Emax hat	E0 hat	Gamma hat	Status	Best Fit Model	Optimal PKPD Index	Target for Stasis	Log1 Kill	Log2 Kill
1	AUC/MIC	1	0.900	36.6	526.50	4.312 FIX	1.968 FIX	1 FIX	successful	2	-	-	-	-
2	AUC/MIC	2	0.913	35.1	612.50	4.312 FIX	1.968 FIX	1.288	successful	2	-	-	-	-
3	AUC/MIC	3	0.900	38.4	564.50	4.399	1.968 FIX	1 FIX	successful	2	-	-	-	-
4	AUC/MIC	4	0.917	35.3	531.60	3.994	1.968 FIX	1.588	successful	2	-	-	-	-
5	AUC/MIC	5	0.900	40.3	543.40	4.469	2.047	1 FIX	successful	2	-	-	-	-
6	AUC/MIC	6	0.918	36.9	556.60	3.844	1.836	1.664	successful	2	-	-	-	-
7	AUC/MIC	7	0.900	38.6	536.30	4.312 FIX	1.952	1 FIX	successful	2	-	-	-	-
8	AUC/MIC	8							unsuccessful	2	-	-	-	-
9	Cmax/MIC	1	0.896	51.7	77.48	4.312 FIX	1.968 FIX	1 FIX	successful	4	-	-	-	-
10	Cmax/MIC	2	0.944	26.1	88.92	4.312 FIX	1.968 FIX	1.93	successful	4	-	-	-	-
11	Cmax/MIC	3	0.875	51.4	95.19	4.698	1.968 FIX	1 FIX	successful	4	-	-	-	-
12	Cmax/MIC	4	0.949	24.4	80.53	4.038	1.968 FIX	2.194	successful	4	Cmax/MIC	79	128	507
13	Cmax/MIC	5	0.933	31.9	27.72	8.031	5.63	1 FIX	successful	4	-	-	-	-
14	Cmax/MIC	6	0.949	26.3	76.74	4.195	2.108	2.062	successful	4	-	-	-	-
15	Cmax/MIC	7	0.899	53.7	74.96	4.312 FIX	2.002	1 FIX	successful	4	-	-	-	-
16	Cmax/MIC	8							unsuccessful	4	-	-	-	-
17	T>MIC	1	0.749	54.6	59.33	4.312 FIX	1.968 FIX	1 FIX	successful	2	-	-	-	-
18	T>MIC	2	0.922	25.4	86.46	4.312 FIX	1.968 FIX	11.6	successful	2	-	-	-	-
19	T>MIC	3							unsuccessful	2	-	-	-	-
20	T>MIC	4							unsuccessful	2	-	-	-	-
21	T>MIC	5							unsuccessful	2	-	-	-	-
22	T>MIC	6							unsuccessful	2	-	-	-	-
23	T>MIC	7	0.751	56.4	65.89	4.312 FIX	1.857	1 FIX	successful	2	-	-	-	-
24	T>MIC	8							unsuccessful	2	-	-	-	-

Griffith et al. (2008) PMID:17954697 Drug:RWJ-54428

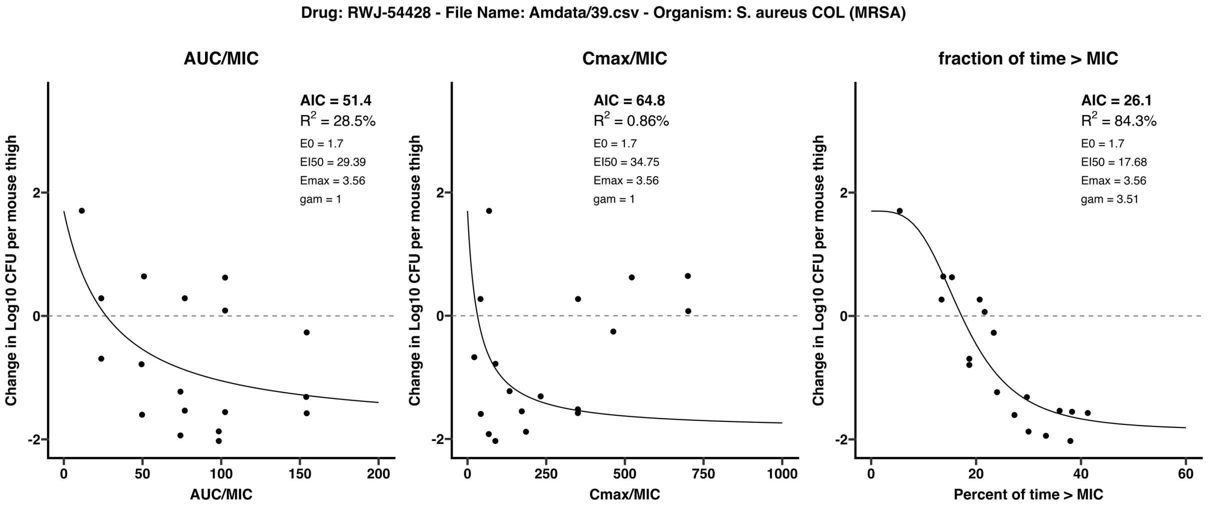

#	Model	Model Number	R²	AIC	EC50 hat	Emax hat	E0 hat	Gamma hat	Status	Best Fit Model	Optimal PKPD Index	Target for Stasis	Log1 Kill	Log2 Kill
1	AUC/MIC	1	0.28500	51.4	29.3900	3.559 FIX	1.7 FIX	1 FIX	successful	1	-	-	-	-
2	AUC/MIC	2	0.28500	53.3	30.3000	3.559 FIX	1.7 FIX	1.057	successful	1	-	-	-	-
3	AUC/MIC	3	0.29300	53.3	24.8900	3.387	1.7 FIX	1 FIX	successful	1	-	-	-	-
4	AUC/MIC	4	0.35000	53.6	20.1000	2.645	1.7 FIX	5.297	successful	1	-	-	-	-
5	AUC/MIC	5							unsuccessful	1	-	-	-	-
6	AUC/MIC	6							unsuccessful	1	-	-	-	-
7	AUC/MIC	7							unsuccessful	1	-	-	-	-
8	AUC/MIC	8	0.35500	53.5	16.1600	3.559 FIX	2.574	2.944	successful	1	-	-	-	-
9	Cmax/MIC	1	0.00855	64.8	34.7500	3.559 FIX	1.7 FIX	1 FIX	successful	1	-	-	-	-
10	Cmax/MIC	2							unsuccessful	1	-	-	-	-
11	Cmax/MIC	3	0.00104	59.2	-1.1350	2.379	1.7 FIX	1 FIX	successful	1	-	-	-	-
12	Cmax/MIC	4							unsuccessful	1	-	-	-	-
13	Cmax/MIC	5							unsuccessful	1	-	-	-	-
14	Cmax/MIC	6							unsuccessful	1	-	-	-	-
15	Cmax/MIC	7	0.00108	59.2	-0.8037	3.559 FIX	2.881	1 FIX	successful	1	-	-	-	-
16	Cmax/MIC	8							unsuccessful	1	-	-	-	-
17	T>MIC	1	0.81500	43.8	11.2900	3.559 FIX	1.7 FIX	1 FIX	successful	2	-	-	-	-
18	T>MIC	2	0.84300	26.1	17.6800	3.559 FIX	1.7 FIX	3.512	successful	2	T>MIC	17	25	60
19	T>MIC	3	0.81200	30.1	187.9000	21.22	1.7 FIX	1 FIX	successful	2	-	-	-	-
20	T>MIC	4	0.85100	27.0	19.4400	4.035	1.7 FIX	2.772	successful	2	-	-	-	-
21	T>MIC	5	0.83800	28.4	30.8400	9.124	3.2	1 FIX	successful	2	-	-	-	-
22	T>MIC	6	0.85100	29.0	19.3100	4.152	1.774	2.665	successful	2	-	-	-	-
23	T>MIC	7							unsuccessful	2	-	-	-	-
24	T>MIC	8	0.84800	27.4	19.3200	3.559 FIX	1.48	3.346	successful	2	-	-	-	-

Griffith et al. (2008) PMID:17954697 Drug:RWJ-54428

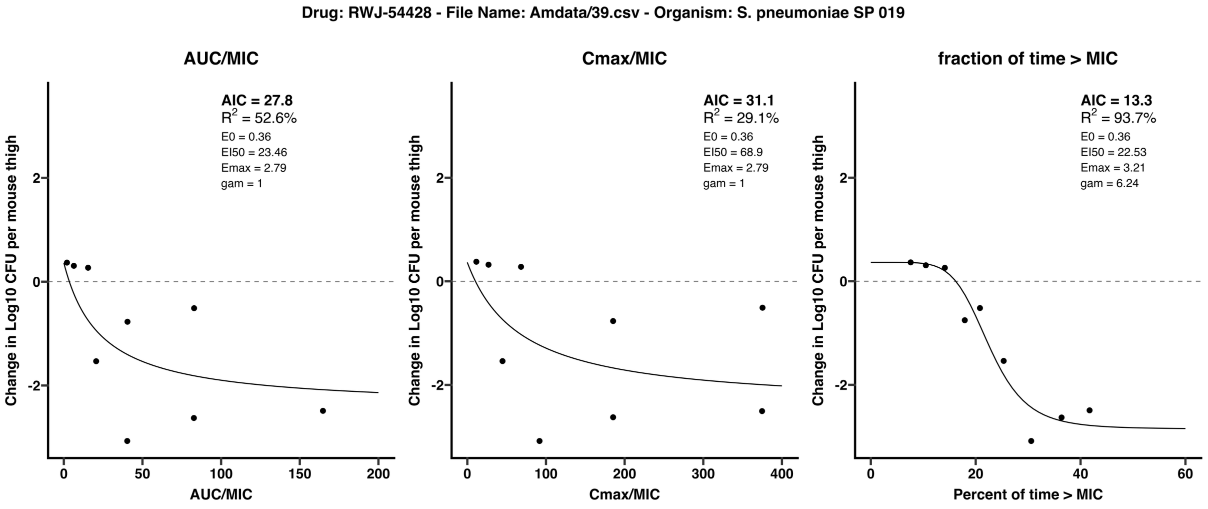

#	Model	Model Number	R²	AIC	EC50 hat	Emax hat	E0 hat	Gamma hat	Status	Best Fit Model	Optimal PKPD Index	Target for Stasis	Log1 Kill	Log2 Kill
1	AUC/MIC	1	0.526	27.8	23.46	2.794 FIX	0.3646 FIX	1 FIX	successful	1	-	-	-	-
2	AUC/MIC	2	0.541	29.1	23.87	2.794 FIX	0.3646 FIX	1.838	successful	1	-	-	-	-
3	AUC/MIC	3	0.518	29.6	33.98	3.271	0.3646 FIX	1 FIX	successful	1	-	-	-	-
4	AUC/MIC	4	0.632	29.0	18.61	2.258	0.3646 FIX	16.62	successful	1	-	-	-	-
5	AUC/MIC	5	0.527	31.3	20.04	3.625	0.9418	1 FIX	successful	1	-	-	-	-
6	AUC/MIC	6	0.632	31.0	18.75	2.23	0.3369	17.76	successful	1	-	-	-	-
7	AUC/MIC	7	0.527	29.8	19.85	2.794 FIX	0.4743	1 FIX	successful	1	-	-	-	-
8	AUC/MIC	8	0.626	29.7	17.92	2.794 FIX	0.7393	10.66	successful	1	-	-	-	-
9	Cmax/MIC	1	0.291	31.1	68.90	2.794 FIX	0.3646 FIX	1 FIX	successful	1	-	-	-	-
10	Cmax/MIC	2	0.295	33.1	68.22	2.794 FIX	0.3646 FIX	1.13	successful	1	-	-	-	-
11	Cmax/MIC	3	0.306	33.0	51.99	2.503	0.3646 FIX	1 FIX	successful	1	-	-	-	-
12	Cmax/MIC	4	0.367	34.0	41.52	2.066	0.3646 FIX	3.374	successful	1	-	-	-	-
13	Cmax/MIC	5	0.333	34.5	15.54	4.413	2.432	1 FIX	successful	1	-	-	-	-
14	Cmax/MIC	6	0.368	36.0	39.15	2.222	0.523	3.247	successful	1	-	-	-	-
15	Cmax/MIC	7	0.323	32.8	32.47	2.794 FIX	0.8244	1 FIX	successful	1	-	-	-	-
16	Cmax/MIC	8							unsuccessful	1	-	-	-	-
17	T>MIC	1	0.842	28.9	17.99	2.794 FIX	0.3646 FIX	1 FIX	successful	4	-	-	-	-
18	T>MIC	2							unsuccessful	4	-	-	-	-
19	T>MIC	3							unsuccessful	4	-	-	-	-
20	T>MIC	4	0.937	13.3	22.53	3.215	0.3646 FIX	6.242	successful	4	T>MIC	16	21	27
21	T>MIC	5	0.869	19.8	75.66	14	1.985	1 FIX	successful	4	-	-	-	-
22	T>MIC	6	0.937	15.2	22.79	3.111	0.2808	6.75	successful	4	-	-	-	-
23	T>MIC	7							unsuccessful	4	-	-	-	-
24	T>MIC	8	0.935	13.9	22.98	2.794 FIX	0.126	8.395	successful	4	-	-	-	-

Griffith et al. (2008) PMID:17954697 Drug:RWJ-54428

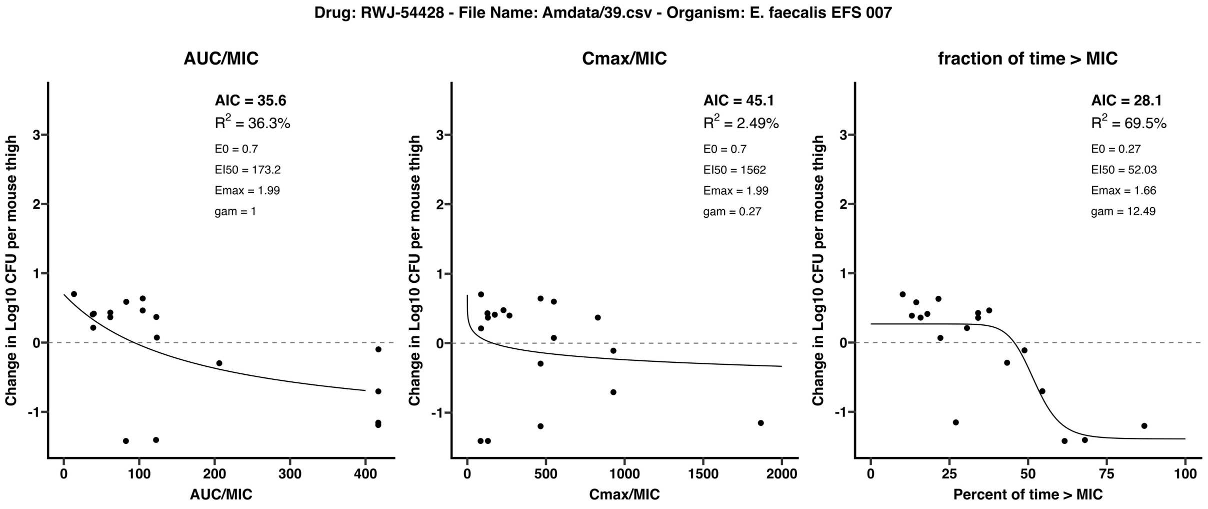

#	Model	Model Number	R²	AIC	EC50 hat	Emax hat	E0 hat	Gamma hat	Status	Best Fit Model	Optimal PKPD Index	Target for Stasis	Log1 Kill	Log2 Kill
1	AUC/MIC	1	0.363000	35.6	173.200	1.988 FIX	0.6953 FIX	1 FIX	successful	1	-	-	-	-
2	AUC/MIC	2	0.362000	37.5	167.400	1.988 FIX	0.6953 FIX	1.14	successful	1	-	-	-	-
3	AUC/MIC	3	0.362000	37.5	223.100	2.269	0.6953 FIX	1 FIX	successful	1	-	-	-	-
4	AUC/MIC	4	0.362000	39.5	179.800	2.057	0.6953 FIX	1.101	successful	1	-	-	-	-
5	AUC/MIC	5	0.363000	39.5	169.500	2.22	0.8036	1 FIX	successful	1	-	-	-	-
6	AUC/MIC	6							unsuccessful	1	-	-	-	-
7	AUC/MIC	7	0.362000	37.6	141.900	1.988 FIX	0.7824	1 FIX	successful	1	-	-	-	-
8	AUC/MIC	8							unsuccessful	1	-	-	-	-
9	Cmax/MIC	1	0.033300	45.2	668.300	1.988 FIX	0.6953 FIX	1 FIX	successful	2	-	-	-	-
10	Cmax/MIC	2	0.024900	45.1	1,562.000	1.988 FIX	0.6953 FIX	0.2745	successful	2	-	-	-	-
11	Cmax/MIC	3	0.000733	45.5	8.759	0.8152	0.6953 FIX	1 FIX	successful	2	-	-	-	-
12	Cmax/MIC	4							unsuccessful	2	-	-	-	-
13	Cmax/MIC	5							unsuccessful	2	-	-	-	-
14	Cmax/MIC	6							unsuccessful	2	-	-	-	-
15	Cmax/MIC	7	0.000494	45.5	2.372	1.988 FIX	1.878	1 FIX	successful	2	-	-	-	-
16	Cmax/MIC	8							unsuccessful	2	-	-	-	-
17	T>MIC	1	0.586000	33.3	45.460	1.988 FIX	0.6953 FIX	1 FIX	successful	6	-	-	-	-
18	T>MIC	2	0.605000	29.1	41.070	1.988 FIX	0.6953 FIX	2.759	successful	6	-	-	-	-
19	T>MIC	3							unsuccessful	6	-	-	-	-
20	T>MIC	4	0.641000	29.1	91.420	4.619	0.6953 FIX	1.72	successful	6	-	-	-	-
21	T>MIC	5							unsuccessful	6	-	-	-	-
22	T>MIC	6	0.695000	28.1	52.030	1.659	0.268	12.49	successful	6	T>MIC	46	57	100
23	T>MIC	7							unsuccessful	6	-	-	-	-
24	T>MIC	8	0.687000	26.9	53.440	1.988 FIX	0.3207	8.159	successful	6	-	-	-	-

Yokoyama et al. (2014) PMID:24796218 Drug:Sulbactam

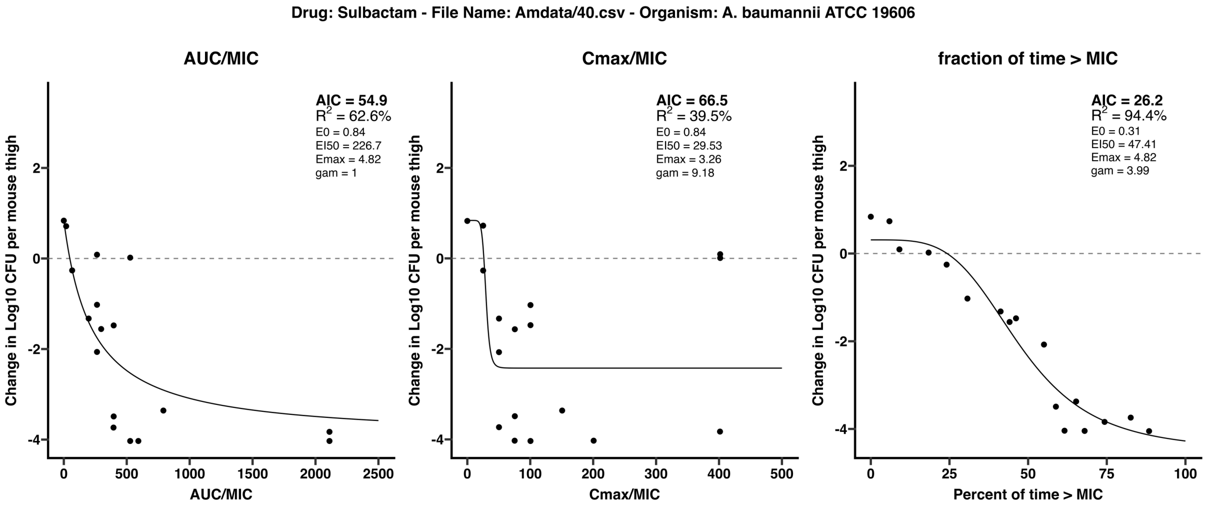

#	Model	Model Number	R²	AIC	EC50 hat	Emax hat	E0 hat	Gamma hat	Status	Best Fit Model	Optimal PKPD Index	Target for Stasis	Log1 Kill	Log2 Kill
1	AUC/MIC	1	0.626	54.9	226.70	4.819 FIX	0.839 FIX	1 FIX	successful	1	-	-	-	-
2	AUC/MIC	2	0.641	55.7	272.10	4.819 FIX	0.839 FIX	1.723	successful	1	-	-	-	-
3	AUC/MIC	3	0.635	55.9	357.70	5.826	0.839 FIX	1 FIX	successful	1	-	-	-	-
4	AUC/MIC	4	0.642	57.6	291.30	5.112	0.839 FIX	1.497	successful	1	-	-	-	-
5	AUC/MIC	5	0.635	57.9	339.30	5.899	0.9482	1 FIX	successful	1	-	-	-	-
6	AUC/MIC	6	0.643	59.5	303.90	4.699	0.5853	1.8	successful	1	-	-	-	-
7	AUC/MIC	7	0.633	56.7	277.70	4.819 FIX	0.6106	1 FIX	successful	1	-	-	-	-
8	AUC/MIC	8							unsuccessful	1	-	-	-	-
9	Cmax/MIC	1	0.183	68.7	44.98	4.819 FIX	0.839 FIX	1 FIX	successful	4	-	-	-	-
10	Cmax/MIC	2	0.179	69.7	23.00	4.819 FIX	0.839 FIX	0.321	successful	4	-	-	-	-
11	Cmax/MIC	3	0.236	68.5	16.43	3.565	0.839 FIX	1 FIX	successful	4	-	-	-	-
12	Cmax/MIC	4	0.395	66.5	29.53	3.264	0.839 FIX	9.18	successful	4	-	-	-	-
13	Cmax/MIC	5							unsuccessful	4	-	-	-	-
14	Cmax/MIC	6	0.395	68.5	29.59	3.252	0.8267	9.194	successful	4	-	-	-	-
15	Cmax/MIC	7	0.236	68.8	15.37	4.819 FIX	1.87	1 FIX	successful	4	-	-	-	-
16	Cmax/MIC	8							unsuccessful	4	-	-	-	-
17	T>MIC	1	0.792	50.4	26.26	4.819 FIX	0.839 FIX	1 FIX	successful	8	-	-	-	-
18	T>MIC	2	0.925	31.2	39.72	4.819 FIX	0.839 FIX	3.584	successful	8	-	-	-	-
19	T>MIC	3	0.927	29.2	11,440.00	706	0.839 FIX	1 FIX	successful	8	-	-	-	-
20	T>MIC	4	0.942	27.3	55.48	7.144	0.839 FIX	2.093	successful	8	-	-	-	-
21	T>MIC	5	0.928	30.5	727.30	51.84	1.078	1 FIX	successful	8	-	-	-	-
22	T>MIC	6	0.946	27.7	49.40	5.481	0.4502	3.127	successful	8	-	-	-	-
23	T>MIC	7	0.817	52.1	32.12	4.819 FIX	0.5838	1 FIX	successful	8	-	-	-	-
24	T>MIC	8	0.944	26.2	47.41	4.819 FIX	0.311	3.989	successful	8	T>MIC	24	37	46

Hegde et al. (2012) PMID:22155835 Drug:TD-1792

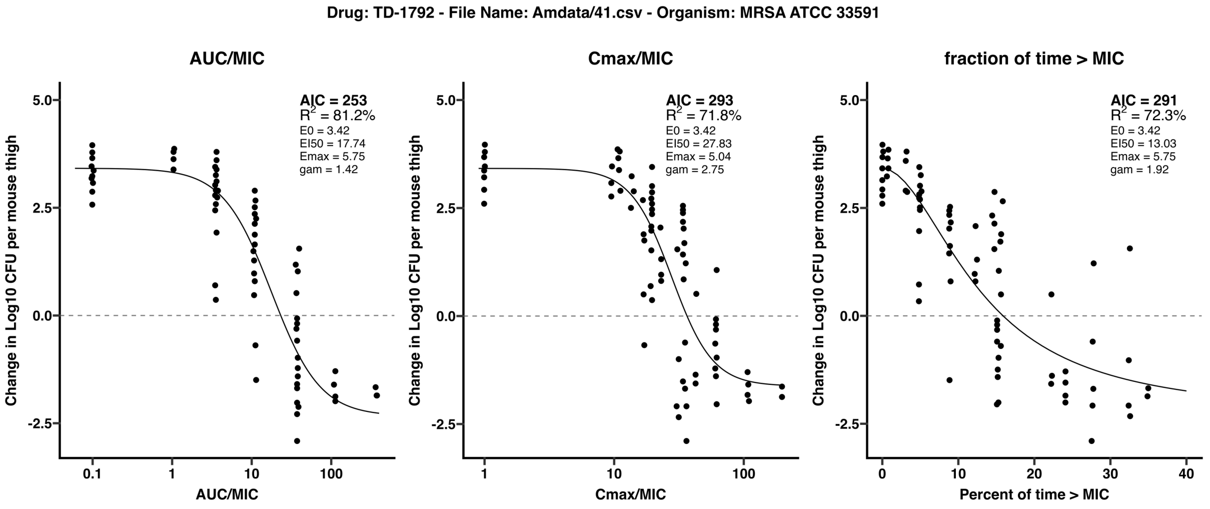

#	Model	Model Number	R²	AIC	EC50 hat	Emax hat	E0 hat	Gamma hat	Status	Best Fit Model	Optimal PKPD Index	Target for Stasis	Log1 Kill	Log2 Kill
1	AUC/MIC	1	0.796	261	18.67	5.753 FIX	3.417 FIX	1 FIX	successful	2	-	-	-	-
2	AUC/MIC	2	0.812	253	17.74	5.753 FIX	3.417 FIX	1.421	successful	2	AUC/MIC	23.16	41.16	125.46
3	AUC/MIC	3	0.796	261	22.82	6.356	3.417 FIX	1 FIX	successful	2	-	-	-	-
4	AUC/MIC	4	0.813	254	15.70	5.38	3.417 FIX	1.57	successful	2	-	-	-	-
5	AUC/MIC	5	0.797	262	20.18	6.473	3.631	1 FIX	successful	2	-	-	-	-
6	AUC/MIC	6	0.814	255	16.00	5.205	3.302	1.675	successful	2	-	-	-	-
7	AUC/MIC	7	0.795	263	17.14	5.753 FIX	3.525	1 FIX	successful	2	-	-	-	-
8	AUC/MIC	8	0.812	255	17.60	5.753 FIX	3.429	1.418	successful	2	-	-	-	-
9	Cmax/MIC	1	0.661	320	38.62	5.753 FIX	3.417 FIX	1 FIX	successful	4	-	-	-	-
10	Cmax/MIC	2	0.712	293	32.37	5.753 FIX	3.417 FIX	2.205	successful	4	-	-	-	-
11	Cmax/MIC	3	0.657	313	74.71	8.597	3.417 FIX	1 FIX	successful	4	-	-	-	-
12	Cmax/MIC	4	0.718	293	27.83	5.039	3.417 FIX	2.747	successful	4	-	-	-	-
13	Cmax/MIC	5	0.665	309	50.58	8.598	4.117	1 FIX	successful	4	-	-	-	-
14	Cmax/MIC	6	0.718	295	27.56	5.135	3.492	2.665	successful	4	-	-	-	-
15	Cmax/MIC	7	0.652	321	31.94	5.753 FIX	3.694	1 FIX	successful	4	-	-	-	-
16	Cmax/MIC	8							unsuccessful	4	-	-	-	-
17	T>MIC	1	0.681	308	12.81	5.753 FIX	3.417 FIX	1 FIX	successful	2	-	-	-	-
18	T>MIC	2	0.723	291	13.03	5.753 FIX	3.417 FIX	1.923	successful	2	-	-	-	-
19	T>MIC	3	0.713	295	49.15	13.02	3.417 FIX	1 FIX	successful	2	-	-	-	-
20	T>MIC	4	0.723	293	13.95	6.072	3.417 FIX	1.8	successful	2	-	-	-	-
21	T>MIC	5	0.713	297	41.54	12.12	3.567	1 FIX	successful	2	-	-	-	-
22	T>MIC	6	0.724	295	13.90	5.95	3.368	1.856	successful	2	-	-	-	-
23	T>MIC	7	0.678	310	12.30	5.753 FIX	3.477	1 FIX	successful	2	-	-	-	-
24	T>MIC	8	0.723	293	13.45	5.753 FIX	3.348	1.94	successful	2	-	-	-	-

Liu et al. (2023) PMID:36329373 Drug:Tedizolid

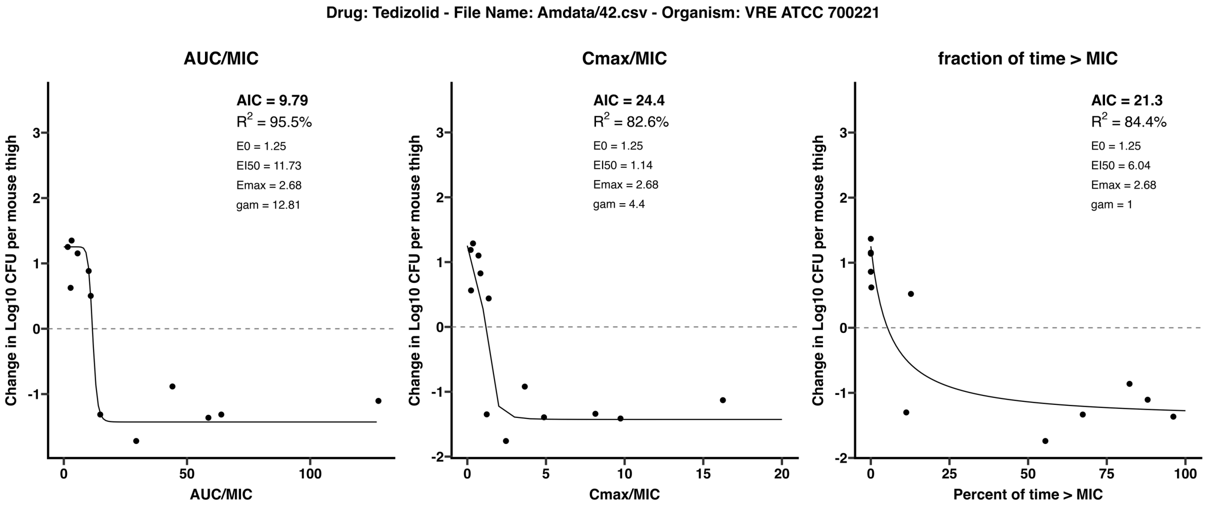

#	Model	Model Number	R²	AIC	EC50 hat	Emax hat	E0 hat	Gamma hat	Status	Best Fit Model	Optimal PKPD Index	Target for Stasis	Log1 Kill	Log2 Kill
1	AUC/MIC	1	0.771	27.70	12.4400	2.68 FIX	1.253 FIX	1 FIX	successful	2	-	-	-	-
2	AUC/MIC	2	0.955	9.79	11.7300	2.68 FIX	1.253 FIX	12.81	successful	2	AUC/MIC	12	13	127
3	AUC/MIC	3	0.757	28.70	17.2600	3.101	1.253 FIX	1 FIX	successful	2	-	-	-	-
4	AUC/MIC	4	0.955	9.34	11.4900	2.516	1.253 FIX	15.45	successful	2	-	-	-	-
5	AUC/MIC	5	0.774	29.00	9.5860	3.671	1.969	1 FIX	successful	2	-	-	-	-
6	AUC/MIC	6	0.957	9.52	11.5600	2.348	1.089	19.01	successful	2	-	-	-	-
7	AUC/MIC	7							unsuccessful	2	-	-	-	-
8	AUC/MIC	8	0.954	11.60	11.6300	2.68 FIX	1.292	13.1	successful	2	-	-	-	-
9	Cmax/MIC	1	0.724	29.90	1.1250	2.68 FIX	1.253 FIX	1 FIX	successful	2	-	-	-	-
10	Cmax/MIC	2	0.826	24.40	1.1350	2.68 FIX	1.253 FIX	4.405	successful	2	-	-	-	-
11	Cmax/MIC	3	0.712	30.70	1.6250	3.166	1.253 FIX	1 FIX	successful	2	-	-	-	-
12	Cmax/MIC	4	0.826	26.00	1.1020	2.557	1.253 FIX	4.763	successful	2	-	-	-	-
13	Cmax/MIC	5	0.726	31.60	0.9557	3.709	1.924	1 FIX	successful	2	-	-	-	-
14	Cmax/MIC	6	0.828	27.60	1.1440	2.381	1.079	5.22	successful	2	-	-	-	-
15	Cmax/MIC	7							unsuccessful	2	-	-	-	-
16	Cmax/MIC	8	0.826	26.40	1.1300	2.68 FIX	1.263	4.409	successful	2	-	-	-	-
17	T>MIC	1	0.844	21.30	6.0390	2.68 FIX	1.253 FIX	1 FIX	successful	1	-	-	-	-
18	T>MIC	2	0.843	22.90	2.4430	2.68 FIX	1.253 FIX	0.6262	successful	1	-	-	-	-
19	T>MIC	3	0.845	23.20	6.7440	2.736	1.253 FIX	1 FIX	successful	1	-	-	-	-
20	T>MIC	4							unsuccessful	1	-	-	-	-
21	T>MIC	5	0.846	24.20	8.0560	2.548	1.042	1 FIX	successful	1	-	-	-	-
22	T>MIC	6							unsuccessful	1	-	-	-	-
23	T>MIC	7	0.845	22.30	9.3740	2.68 FIX	1.077	1 FIX	successful	1	-	-	-	-
24	T>MIC	8	0.849	24.00	6.1500	2.68 FIX	1.059	0.684	successful	1	-	-	-	-

Liu et al. (2023) PMID:36329373 Drug:Tedizolid

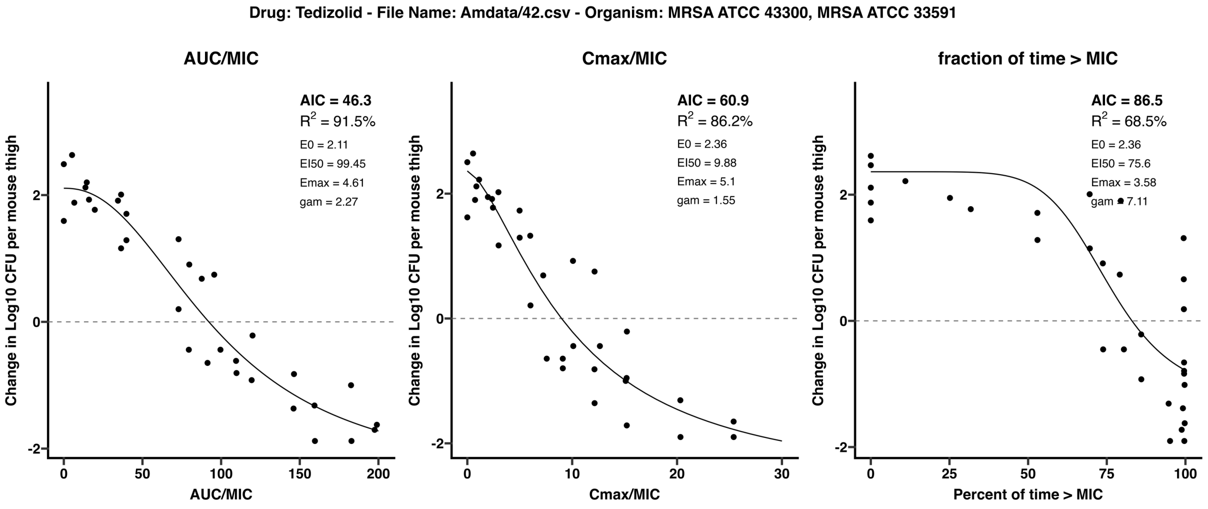

#	Model	Model Number	R²	AIC	EC50 hat	Emax hat	E0 hat	Gamma hat	Status	Best Fit Model	Optimal PKPD Index	Target for Stasis	Log1 Kill	Log2 Kill
1	AUC/MIC	1	0.779	81.6	48.330	3.579 FIX	2.363 FIX	1 FIX	successful	6	-	-	-	-
2	AUC/MIC	2	0.885	55.9	68.870	3.579 FIX	2.363 FIX	3.034	successful	6	-	-	-	-
3	AUC/MIC	3	0.901	47.3	633.700	17.95	2.363 FIX	1 FIX	successful	6	-	-	-	-
4	AUC/MIC	4	0.912	46.0	114.800	5.779	2.363 FIX	1.673	successful	6	-	-	-	-
5	AUC/MIC	5	0.901	49.2	531.500	15.96	2.432	1 FIX	successful	6	-	-	-	-
6	AUC/MIC	6	0.915	46.3	99.450	4.613	2.108	2.272	successful	6	AUC/MIC	92	137	200
7	AUC/MIC	7	0.812	82.5	63.460	3.579 FIX	2.101	1 FIX	successful	6	-	-	-	-
8	AUC/MIC	8							unsuccessful	6	-	-	-	-
9	Cmax/MIC	1	0.775	81.4	4.806	3.579 FIX	2.363 FIX	1 FIX	successful	4	-	-	-	-
10	Cmax/MIC	2	0.844	64.0	6.349	3.579 FIX	2.363 FIX	2.581	successful	4	-	-	-	-
11	Cmax/MIC	3	0.853	61.0	25.520	8.754	2.363 FIX	1 FIX	successful	4	-	-	-	-
12	Cmax/MIC	4	0.862	60.9	9.880	5.099	2.363 FIX	1.55	successful	4	-	-	-	-
13	Cmax/MIC	5	0.853	62.7	22.260	8.348	2.47	1 FIX	successful	4	-	-	-	-
14	Cmax/MIC	6	0.863	62.5	9.483	4.657	2.218	1.762	successful	4	-	-	-	-
15	Cmax/MIC	7	0.801	82.4	6.249	3.579 FIX	2.117	1 FIX	successful	4	-	-	-	-
16	Cmax/MIC	8	0.857	62.3	7.732	3.579 FIX	2.04	2.587	successful	4	-	-	-	-
17	T>MIC	1	0.490	99.6	44.080	3.579 FIX	2.363 FIX	1 FIX	successful	2	-	-	-	-
18	T>MIC	2	0.685	86.5	75.600	3.579 FIX	2.363 FIX	7.106	successful	2	-	-	-	-
19	T>MIC	3							unsuccessful	2	-	-	-	-
20	T>MIC	4	0.682	88.4	79.950	4.198	2.363 FIX	5.028	successful	2	-	-	-	-
21	T>MIC	5							unsuccessful	2	-	-	-	-
22	T>MIC	6							unsuccessful	2	-	-	-	-
23	T>MIC	7	0.509	101.0	57.180	3.579 FIX	2.123	1 FIX	successful	2	-	-	-	-
24	T>MIC	8							unsuccessful	2	-	-	-	-

Watanabe et al. (2021) PMID:33290889 Drug:Teicoplanin

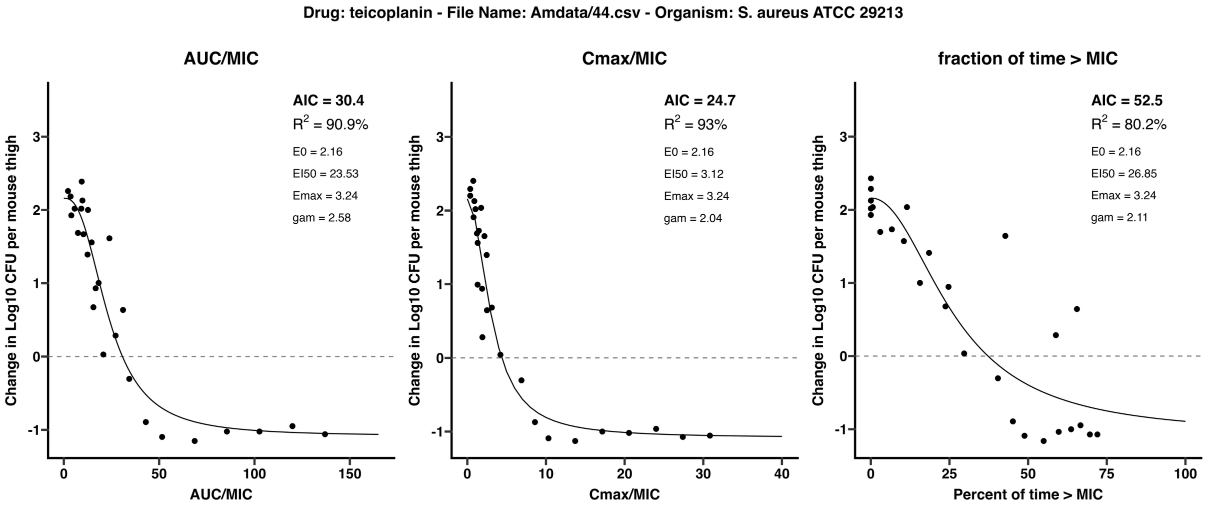

#	Model	Model Number	R²	AIC	EC50 hat	Emax hat	E0 hat	Gamma hat	Status	Best Fit Model	Optimal PKPD Index	Target for Stasis	Log1 Kill	Log2 Kill
1	AUC/MIC	1	0.875	56.3	26.490	3.243 FIX	2.16 FIX	1 FIX	successful	2	-	-	-	-
2	AUC/MIC	2	0.909	30.4	23.530	3.243 FIX	2.16 FIX	2.58	successful	2	-	-	-	-
3	AUC/MIC	3	0.852	46.2	64.130	5.421	2.16 FIX	1 FIX	successful	2	-	-	-	-
4	AUC/MIC	4	0.910	32.1	24.510	3.366	2.16 FIX	2.451	successful	2	-	-	-	-
5	AUC/MIC	5	0.876	40.7	30.820	5.355	2.935	1 FIX	successful	2	-	-	-	-
6	AUC/MIC	6	0.910	34.1	24.470	3.374	2.167	2.439	successful	2	-	-	-	-
7	AUC/MIC	7	0.873	58.2	24.420	3.243 FIX	2.23	1 FIX	successful	2	-	-	-	-
8	AUC/MIC	8	0.909	32.3	24.390	3.243 FIX	2.107	2.607	successful	2	-	-	-	-
9	Cmax/MIC	1	0.919	45.7	3.641	3.243 FIX	2.16 FIX	1 FIX	successful	2	-	-	-	-
10	Cmax/MIC	2	0.930	24.7	3.120	3.243 FIX	2.16 FIX	2.043	successful	2	Cmax/MIC	4	19	40
11	Cmax/MIC	3	0.898	37.1	6.310	4.294	2.16 FIX	1 FIX	successful	2	-	-	-	-
12	Cmax/MIC	4	0.930	26.5	3.221	3.308	2.16 FIX	1.97	successful	2	-	-	-	-
13	Cmax/MIC	5	0.921	29.6	2.995	4.72	3.008	1 FIX	successful	2	-	-	-	-
14	Cmax/MIC	6	0.931	27.9	3.046	3.554	2.354	1.725	successful	2	-	-	-	-
15	Cmax/MIC	7	0.919	47.7	3.605	3.243 FIX	2.168	1 FIX	successful	2	-	-	-	-
16	Cmax/MIC	8	0.930	26.7	3.106	3.243 FIX	2.165	2.043	successful	2	-	-	-	-
17	T>MIC	1	0.750	58.4	21.640	3.243 FIX	2.16 FIX	1 FIX	successful	2	-	-	-	-
18	T>MIC	2	0.802	52.5	26.850	3.243 FIX	2.16 FIX	2.108	successful	2	-	-	-	-
19	T>MIC	3	0.800	52.6	118.300	8.199	2.16 FIX	1 FIX	successful	2	-	-	-	-
20	T>MIC	4	0.803	54.2	35.840	4.005	2.16 FIX	1.577	successful	2	-	-	-	-
21	T>MIC	5	0.800	54.6	116.100	8.119	2.168	1 FIX	successful	2	-	-	-	-
22	T>MIC	6	0.804	56.1	33.730	3.685	2.086	1.758	successful	2	-	-	-	-
23	T>MIC	7	0.757	60.3	23.860	3.243 FIX	2.081	1 FIX	successful	2	-	-	-	-
24	T>MIC	8	0.803	54.2	29.300	3.243 FIX	2.053	2.104	successful	2	-	-	-	-

Watanabe et al. (2021) PMID:33290889 Drug:Teicoplanin

Drug: teicoplanin - File Name: Amdata/44.csv - Organism: S. aureus ATCC 43300

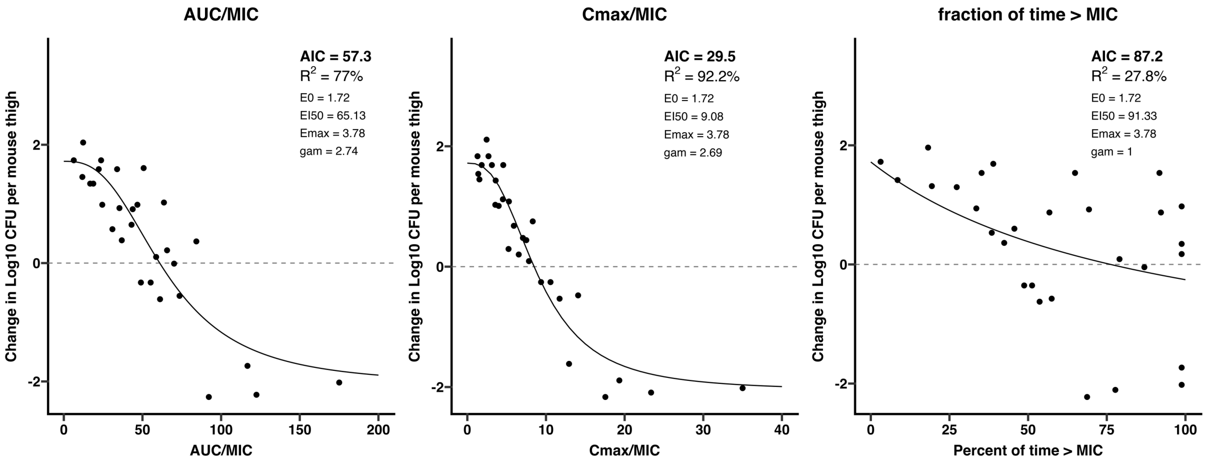

#	Model	Model Number	R²	AIC	EC50 hat	Emax hat	E0 hat	Gamma hat	Status	Best Fit Model	Optimal PKPD Index	Target for Stasis	Log1 Kill	Log2 Kill
1	AUC/MIC	1	0.729	72.6	78.480	3.781 FIX	1.72 FIX	1 FIX	successful	2	-	-	-	-
2	AUC/MIC	2	0.770	57.3	65.130	3.781 FIX	1.72 FIX	2.737	successful	2	-	-	-	-
3	AUC/MIC	3	0.756	60.0	3,361.000	90.2	1.72 FIX	1 FIX	successful	2	-	-	-	-
4	AUC/MIC	4	0.784	57.2	85.780	5.074	1.72 FIX	2.112	successful	2	-	-	-	-
5	AUC/MIC	5	0.769	59.1	327.100	13.99	2.253	1 FIX	successful	2	-	-	-	-
6	AUC/MIC	6	0.785	59.1	82.220	4.634	1.592	2.423	successful	2	-	-	-	-
7	AUC/MIC	7	0.704	73.8	56.140	3.781 FIX	2.047	1 FIX	successful	2	-	-	-	-
8	AUC/MIC	8	0.781	57.7	73.480	3.781 FIX	1.44	3.163	successful	2	-	-	-	-
9	Cmax/MIC	1	0.890	65.5	10.900	3.781 FIX	1.72 FIX	1 FIX	successful	2	-	-	-	-
10	Cmax/MIC	2	0.922	29.5	9.076	3.781 FIX	1.72 FIX	2.689	successful	2	Cmax/MIC	8	13	40
11	Cmax/MIC	3	0.874	48.0	53.250	11.63	1.72 FIX	1 FIX	successful	2	-	-	-	-
12	Cmax/MIC	4	0.926	29.5	10.170	4.239	1.72 FIX	2.37	successful	2	-	-	-	-
13	Cmax/MIC	5	0.901	37.8	18.570	8.041	2.555	1 FIX	successful	2	-	-	-	-
14	Cmax/MIC	6	0.926	31.5	10.150	4.32	1.764	2.29	successful	2	-	-	-	-
15	Cmax/MIC	7	0.872	66.3	7.997	3.781 FIX	2.029	1 FIX	successful	2	-	-	-	-
16	Cmax/MIC	8	0.923	31.0	9.514	3.781 FIX	1.629	2.766	successful	2	-	-	-	-
17	T>MIC	1	0.278	87.2	91.330	3.781 FIX	1.72 FIX	1 FIX	successful	1	-	-	-	-
18	T>MIC	2	0.278	88.8	82.860	3.781 FIX	1.72 FIX	1.326	successful	1	-	-	-	-
19	T>MIC	3	0.274	89.1	145.200	5.064	1.72 FIX	1 FIX	successful	1	-	-	-	-
20	T>MIC	4							unsuccessful	1	-	-	-	-
21	T>MIC	5	0.279	90.7	69.410	4.305	2.203	1 FIX	successful	1	-	-	-	-
22	T>MIC	6	0.341	90.1	41.600	1.631	1.483	15.05	successful	1	-	-	-	-
23	T>MIC	7	0.278	88.8	53.890	3.781 FIX	2.195	1 FIX	successful	1	-	-	-	-
24	T>MIC	8	0.283	90.6	59.910	3.781 FIX	2.089	1.238	successful	1	-	-	-	-

Hegde et al. (2004) PMID:15273119 Drug:Telavancin (TD-6424)

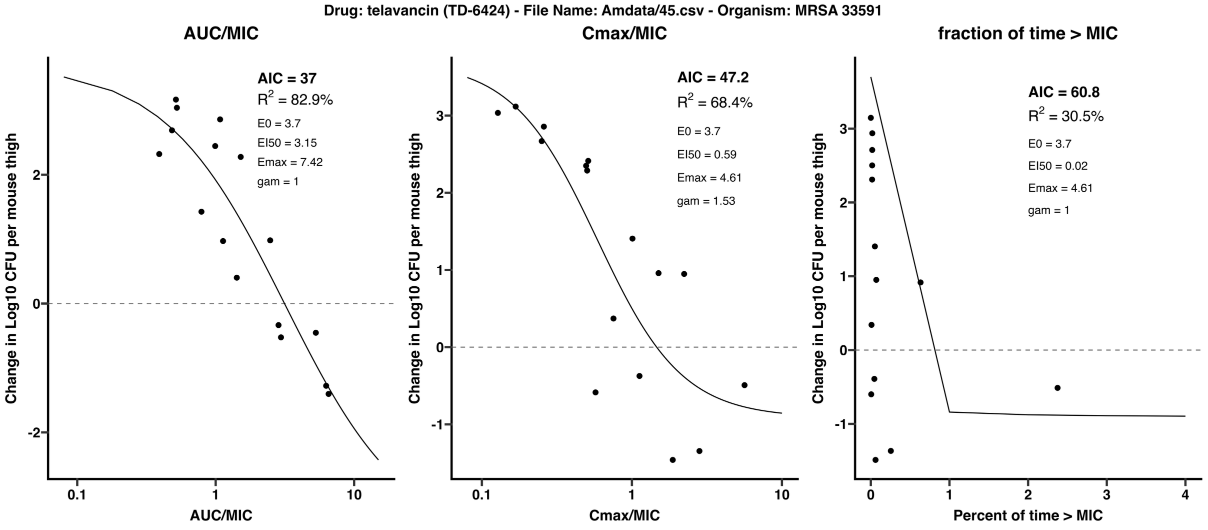

#	Model	Model Number	R²	AIC	EC50 hat	Emax hat	E0 hat	Gamma hat	Status	Best Fit Model	Optimal PKPD Index	Target for Stasis	Log1 Kill	Log2 Kill
1	AUC/MIC	1	0.809	43.1	1.236000	4.614 FIX	3.7 FIX	1 FIX	successful	3	-	-	-	-
2	AUC/MIC	2	0.802	39.7	1.291000	4.614 FIX	3.7 FIX	1.707	successful	3	-	-	-	-
3	AUC/MIC	3	0.829	37.0	3.155000	7.417	3.7 FIX	1 FIX	successful	3	AUC/MIC	3.18	5.48	10.48
4	AUC/MIC	4	0.829	38.9	2.573000	6.759	3.7 FIX	1.094	successful	3	-	-	-	-
5	AUC/MIC	5	0.829	39.0	3.015000	7.373	3.752	1 FIX	successful	3	-	-	-	-
6	AUC/MIC	6	0.832	40.7	2.166000	4.85	3.023	1.732	successful	3	-	-	-	-
7	AUC/MIC	7							unsuccessful	3	-	-	-	-
8	AUC/MIC	8							unsuccessful	3	-	-	-	-
9	Cmax/MIC	1	0.675	47.2	0.569400	4.614 FIX	3.7 FIX	1 FIX	successful	2	-	-	-	-
10	Cmax/MIC	2	0.684	47.2	0.587900	4.614 FIX	3.7 FIX	1.534	successful	2	-	-	-	-
11	Cmax/MIC	3	0.667	48.1	0.825900	5.452	3.7 FIX	1 FIX	successful	2	-	-	-	-
12	Cmax/MIC	4	0.684	49.1	0.554700	4.451	3.7 FIX	1.652	successful	2	-	-	-	-
13	Cmax/MIC	5	0.676	49.6	0.498600	6.015	4.618	1 FIX	successful	2	-	-	-	-
14	Cmax/MIC	6	0.686	51.0	0.588300	3.903	3.334	2.101	successful	2	-	-	-	-
15	Cmax/MIC	7	0.674	49.1	0.628900	4.614 FIX	3.592	1 FIX	successful	2	-	-	-	-
16	Cmax/MIC	8							unsuccessful	2	-	-	-	-
17	T>MIC	1	0.305	60.8	0.016270	4.614 FIX	3.7 FIX	1 FIX	successful	1	-	-	-	-
18	T>MIC	2	0.302	61.8	0.009873	4.614 FIX	3.7 FIX	0.5571	successful	1	-	-	-	-
19	T>MIC	3	0.293	62.7	0.013280	4.358	3.7 FIX	1 FIX	successful	1	-	-	-	-
20	T>MIC	4	0.303	63.8	0.011810	4.832	3.7 FIX	0.5161	successful	1	-	-	-	-
21	T>MIC	5	0.326	63.3	0.034380	3.25	2.354	1 FIX	successful	1	-	-	-	-
22	T>MIC	6							unsuccessful	1	-	-	-	-
23	T>MIC	7							unsuccessful	1	-	-	-	-
24	T>MIC	8							unsuccessful	1	-	-	-	-

Sugihara et al. (2010) PMID:20921311 Drug:Tomopenem (+cilastatin)

Drug: Tomopenem (+cilastatin) - File Name: Amdata/46.csv - Organism: MRSA 12372

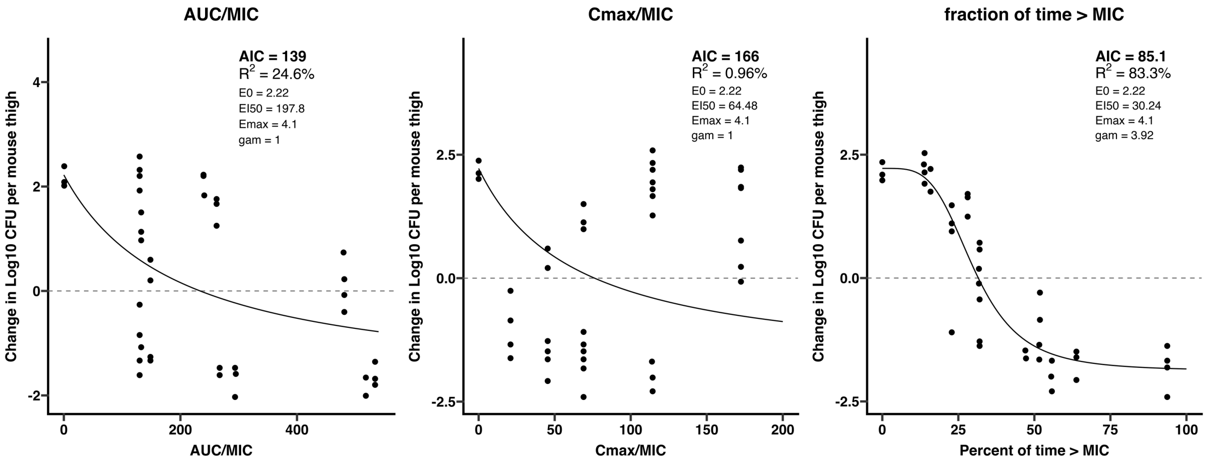

#	Model	Model Number	R²	AIC	EC50 hat	Emax hat	E0 hat	Gamma hat	Status	Best Fit Model	Optimal PKPD Index	Target for Stasis	Log1 Kill	Log2 Kill
1	AUC/MIC	1	0.24600	139.0	197.800	4.101 FIX	2.222 FIX	1 FIX	successful	1	-	-	-	-
2	AUC/MIC	2	0.24600	141.0	198.600	4.101 FIX	2.222 FIX	1.038	successful	1	-	-	-	-
3	AUC/MIC	3	0.24900	141.0	267.000	4.72	2.222 FIX	1 FIX	successful	1	-	-	-	-
4	AUC/MIC	4							unsuccessful	1	-	-	-	-
5	AUC/MIC	5	0.25000	143.0	372.600	4.97	1.965	1 FIX	successful	1	-	-	-	-
6	AUC/MIC	6							unsuccessful	1	-	-	-	-
7	AUC/MIC	7	0.24800	141.0	245.600	4.101 FIX	2.014	1 FIX	successful	1	-	-	-	-
8	AUC/MIC	8							unsuccessful	1	-	-	-	-
9	Cmax/MIC	1	0.00958	166.0	64.480	4.101 FIX	2.222 FIX	1 FIX	successful	1	-	-	-	-
10	Cmax/MIC	2							unsuccessful	1	-	-	-	-
11	Cmax/MIC	3	0.22500	147.0	-10.490	1.776	2.222 FIX	1 FIX	successful	1	-	-	-	-
12	Cmax/MIC	4							unsuccessful	1	-	-	-	-
13	Cmax/MIC	5	0.22500	149.0	-10.180	1.911	2.371	1 FIX	successful	1	-	-	-	-
14	Cmax/MIC	6							unsuccessful	1	-	-	-	-
15	Cmax/MIC	7	0.19300	153.0	-5.762	4.101 FIX	4.394	1 FIX	successful	1	-	-	-	-
16	Cmax/MIC	8							unsuccessful	1	-	-	-	-
17	T>MIC	1	0.66900	120.0	25.500	4.101 FIX	2.222 FIX	1 FIX	successful	2	-	-	-	-
18	T>MIC	2	0.83300	85.1	30.240	4.101 FIX	2.222 FIX	3.924	successful	2	T>MIC	32	42	100
19	T>MIC	3	0.74200	104.0	152.400	12.26	2.222 FIX	1 FIX	successful	2	-	-	-	-
20	T>MIC	4	0.83300	87.0	30.590	4.17	2.222 FIX	3.777	successful	2	-	-	-	-
21	T>MIC	5	0.75000	103.0	86.560	10.22	2.867	1 FIX	successful	2	-	-	-	-
22	T>MIC	6	0.83300	89.0	30.540	4.188	2.236	3.748	successful	2	-	-	-	-
23	T>MIC	7							unsuccessful	2	-	-	-	-
24	T>MIC	8	0.83300	87.1	30.520	4.101 FIX	2.193	3.907	successful	2	-	-	-	-

Sugihara et al. (2010) PMID:20921311 Drug:Tomopenem (+cilastatin)

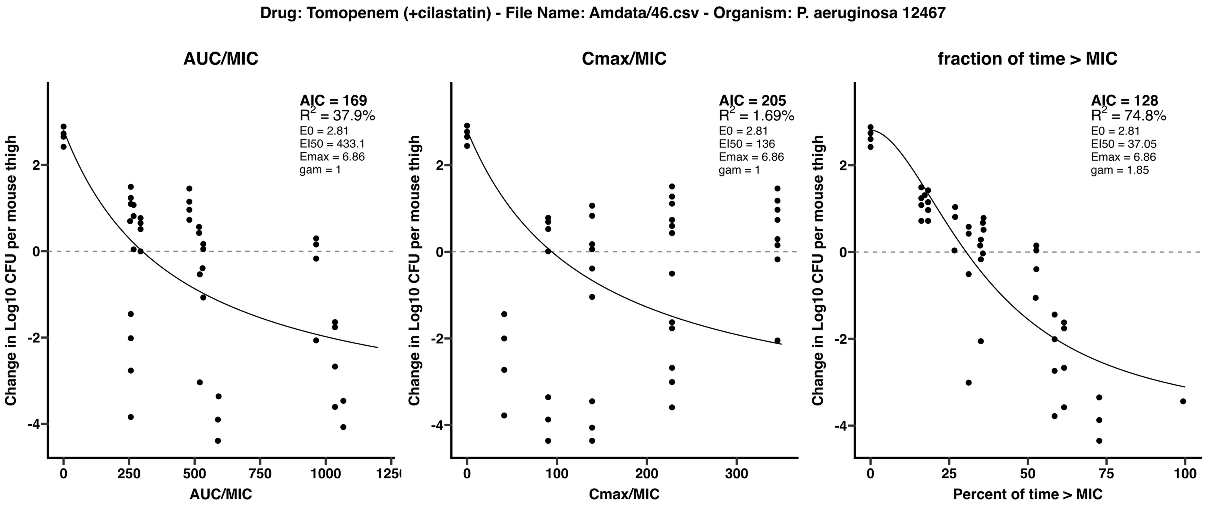

#	Model	Model Number	R²	AIC	EC50 hat	Emax hat	E0 hat	Gamma hat	Status	Best Fit Model	Optimal PKPD Index	Target for Stasis	Log1 Kill	Log2 Kill
1	AUC/MIC	1	0.3790	169	433.100	6.862 FIX	2.809 FIX	1 FIX	successful	1	-	-	-	-
2	AUC/MIC	2	0.3800	171	427.800	6.862 FIX	2.809 FIX	0.8768	successful	1	-	-	-	-
3	AUC/MIC	3	0.3790	171	393.700	6.581	2.809 FIX	1 FIX	successful	1	-	-	-	-
4	AUC/MIC	4							unsuccessful	1	-	-	-	-
5	AUC/MIC	5	0.3790	173	471.500	6.558	2.522	1 FIX	successful	1	-	-	-	-
6	AUC/MIC	6							unsuccessful	1	-	-	-	-
7	AUC/MIC	7	0.3790	171	523.000	6.862 FIX	2.51	1 FIX	successful	1	-	-	-	-
8	AUC/MIC	8							unsuccessful	1	-	-	-	-
9	Cmax/MIC	1	0.0169	205	136.000	6.862 FIX	2.809 FIX	1 FIX	successful	1	-	-	-	-
10	Cmax/MIC	2							unsuccessful	1	-	-	-	-
11	Cmax/MIC	3	0.3370	177	-17.850	3.118	2.809 FIX	1 FIX	successful	1	-	-	-	-
12	Cmax/MIC	4							unsuccessful	1	-	-	-	-
13	Cmax/MIC	5	0.3370	179	-17.990	3.07	2.758	1 FIX	successful	1	-	-	-	-
14	Cmax/MIC	6							unsuccessful	1	-	-	-	-
15	Cmax/MIC	7	0.2880	193	-6.491	6.862 FIX	6.021	1 FIX	successful	1	-	-	-	-
16	Cmax/MIC	8							unsuccessful	1	-	-	-	-
17	T>MIC	1	0.6900	139	36.100	6.862 FIX	2.809 FIX	1 FIX	successful	2	-	-	-	-
18	T>MIC	2	0.7480	128	37.050	6.862 FIX	2.809 FIX	1.85	successful	2	T>MIC	30	42	59
19	T>MIC	3	0.7770	123	324.200	31.35	2.809 FIX	1 FIX	successful	2	-	-	-	-
20	T>MIC	4							unsuccessful	2	-	-	-	-
21	T>MIC	5	0.7770	125	457.900	40.8	2.658	1 FIX	successful	2	-	-	-	-
22	T>MIC	6							unsuccessful	2	-	-	-	-
23	T>MIC	7							unsuccessful	2	-	-	-	-
24	T>MIC	8	0.7610	128	47.250	6.862 FIX	2.117	2.085	successful	2	-	-	-	-

Sugihara et al. (2010) PMID:20921311 Drug:Meropenem

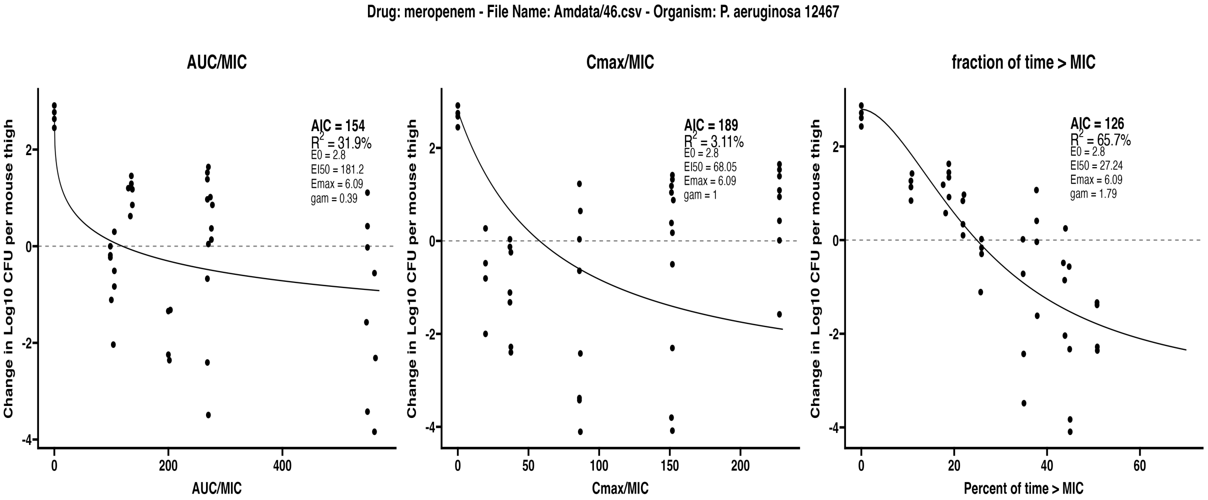

#	Model	Model Number	R²	AIC	EC50 hat	Emax hat	E0 hat	Gamma hat	Status	Best Fit Model	Optimal PKPD Index	Target for Stasis	Log1 Kill	Log2 Kill
1	AUC/MIC	1	0.2770	156	203.70000	6.086 FIX	2.795 FIX	1 FIX	successful	2	-	-	-	-
2	AUC/MIC	2	0.3190	154	181.20000	6.086 FIX	2.795 FIX	0.3914	successful	2	-	-	-	-
3	AUC/MIC	3	0.3080	154	46.96000	3.907	2.795 FIX	1 FIX	successful	2	-	-	-	-
4	AUC/MIC	4							unsuccessful	2	-	-	-	-
5	AUC/MIC	5	0.3080	156	50.52000	3.785	2.655	1 FIX	successful	2	-	-	-	-
6	AUC/MIC	6							unsuccessful	2	-	-	-	-
7	AUC/MIC	7	0.2510	158	409.70000	6.086 FIX	1.915	1 FIX	successful	2	-	-	-	-
8	AUC/MIC	8							unsuccessful	2	-	-	-	-
9	Cmax/MIC	1	0.0311	189	68.05000	6.086 FIX	2.795 FIX	1 FIX	successful	1	-	-	-	-
10	Cmax/MIC	2							unsuccessful	1	-	-	-	-
11	Cmax/MIC	3	0.2760	165	-4.21800	3.124	2.795 FIX	1 FIX	successful	1	-	-	-	-
12	Cmax/MIC	4							unsuccessful	1	-	-	-	-
13	Cmax/MIC	5	0.2760	167	-4.29000	3.045	2.714	1 FIX	successful	1	-	-	-	-
14	Cmax/MIC	6							unsuccessful	1	-	-	-	-
15	Cmax/MIC	7	0.2580	176	-0.09023	6.086 FIX	5.262	1 FIX	successful	1	-	-	-	-
16	Cmax/MIC	8							unsuccessful	1	-	-	-	-
17	T>MIC	1	0.6090	131	25.65000	6.086 FIX	2.795 FIX	1 FIX	successful	2	-	-	-	-
18	T>MIC	2	0.6570	126	27.24000	6.086 FIX	2.795 FIX	1.795	successful	2	T>MIC	25	36	57
19	T>MIC	3	0.6640	125	181.40000	22.15	2.795 FIX	1 FIX	successful	2	-	-	-	-
20	T>MIC	4							unsuccessful	2	-	-	-	-
21	T>MIC	5	0.6640	127	286.90000	31.28	2.632	1 FIX	successful	2	-	-	-	-
22	T>MIC	6							unsuccessful	2	-	-	-	-
23	T>MIC	7	0.6160	133	28.19000	6.086 FIX	2.65	1 FIX	successful	2	-	-	-	-
24	T>MIC	8	0.6600	128	31.68000	6.086 FIX	2.43	1.874	successful	2	-	-	-	-

Louie al. (2011) PMID:21502615 Drug:Torezolid Phosphate (TR-701)

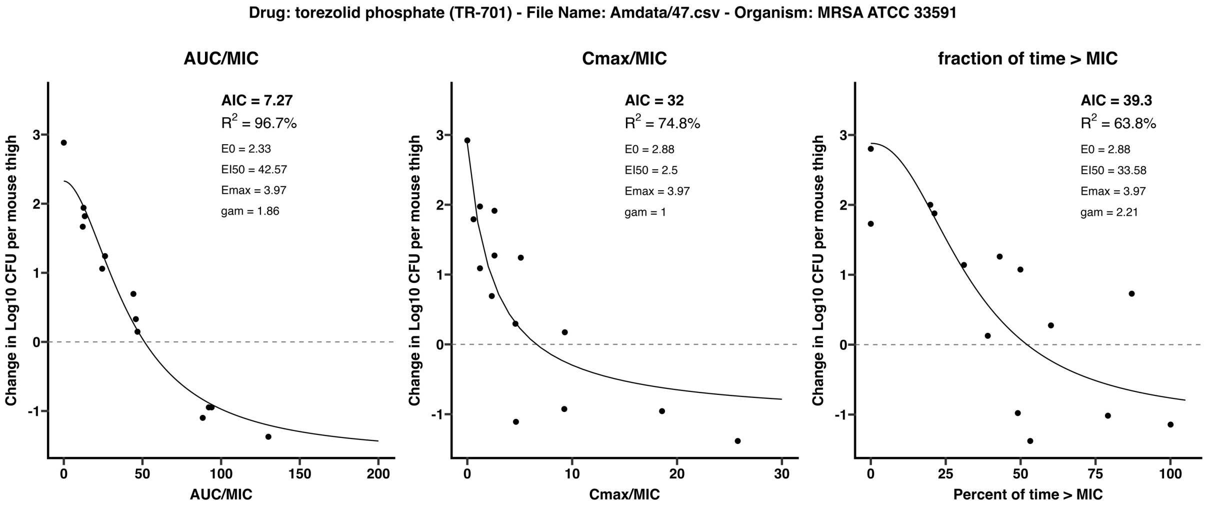

#	Model	Model Number	R²	AIC	EC50 hat	Emax hat	E0 hat	Gamma hat	Status	Best Fit Model	Optimal PKPD Index	Target for Stasis	Log1 Kill	Log2 Kill
1	AUC/MIC	1	0.915	23.60	24.560	3.971 FIX	2.88 FIX	1 FIX	successful	8	-	-	-	-
2	AUC/MIC	2	0.941	14.10	28.090	3.971 FIX	2.88 FIX	1.726	successful	8	-	-	-	-
3	AUC/MIC	3	0.985	-5.86	78.470	7.019	2.88 FIX	1 FIX	successful	8	-	-	-	-
4	AUC/MIC	4	0.985	-4.29	109.900	8.215	2.88 FIX	0.9051	successful	8	-	-	-	-
5	AUC/MIC	5	0.985	-4.18	85.400	7.157	2.806	1 FIX	successful	8	-	-	-	-
6	AUC/MIC	6	0.985	-2.34	104.800	7.965	2.844	0.9282	successful	8	-	-	-	-
7	AUC/MIC	7	0.954	23.90	35.530	3.971 FIX	2.512	1 FIX	successful	8	-	-	-	-
8	AUC/MIC	8	0.967	7.27	42.570	3.971 FIX	2.327	1.864	successful	8	AUC/MIC	51	103	200
9	Cmax/MIC	1	0.748	32.00	2.498	3.971 FIX	2.88 FIX	1 FIX	successful	1	-	-	-	-
10	Cmax/MIC	2	0.747	33.80	2.590	3.971 FIX	2.88 FIX	1.141	successful	1	-	-	-	-
11	Cmax/MIC	3	0.764	32.80	3.746	4.671	2.88 FIX	1 FIX	successful	1	-	-	-	-
12	Cmax/MIC	4	0.770	34.30	8.478	6.239	2.88 FIX	0.6822	successful	1	-	-	-	-
13	Cmax/MIC	5	0.766	34.60	4.571	4.594	2.65	1 FIX	successful	1	-	-	-	-
14	Cmax/MIC	6	0.770	36.30	8.434	6.217	2.874	0.6848	successful	1	-	-	-	-
15	Cmax/MIC	7	0.764	33.30	3.821	3.971 FIX	2.51	1 FIX	successful	1	-	-	-	-
16	Cmax/MIC	8	0.760	35.00	3.971	3.971 FIX	2.48	1.168	successful	1	-	-	-	-
17	T>MIC	1	0.536	40.20	26.470	3.971 FIX	2.88 FIX	1 FIX	successful	2	-	-	-	-
18	T>MIC	2	0.638	39.30	33.580	3.971 FIX	2.88 FIX	2.206	successful	2	-	-	-	-
19	T>MIC	3	0.596	40.50	75.240	6.571	2.88 FIX	1 FIX	successful	2	-	-	-	-
20	T>MIC	4	0.643	41.10	30.360	3.569	2.88 FIX	2.809	successful	2	-	-	-	-
21	T>MIC	5	0.600	41.90	112.100	7.092	2.465	1 FIX	successful	2	-	-	-	-
22	T>MIC	6	0.656	41.80	34.800	2.819	2.257	3.987	successful	2	-	-	-	-
23	T>MIC	7	0.577	41.10	44.900	3.971 FIX	2.349	1 FIX	successful	2	-	-	-	-
24	T>MIC	8	0.635	40.70	41.460	3.971 FIX	2.499	1.989	successful	2	-	-	-	-

Hagihara et al. (2020) PMID:32434196 Drug:Trimethoprim/Sulfamethoxazole

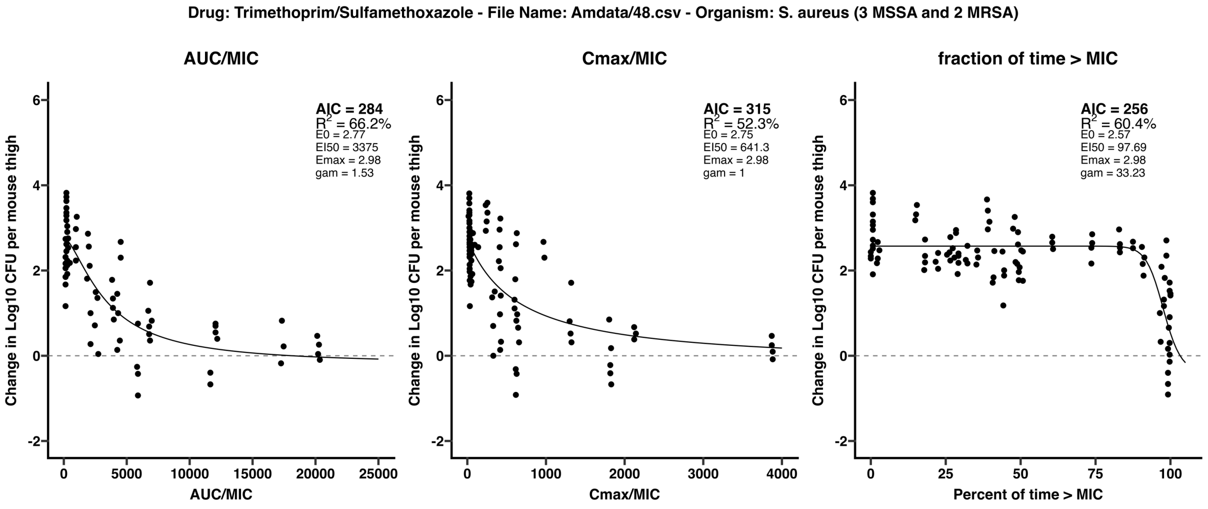

#	Model	Model Number	R²	AIC	EC50 hat	Emax hat	E0 hat	Gamma hat	Status	Best Fit Model	Optimal PKPD Index	Target for Stasis	Log1 Kill	Log2 Kill
1	AUC/MIC	1	0.644	318	6,963.00	2.984 FIX	2.309 FIX	1 FIX	successful	8	-	-	-	-
2	AUC/MIC	2	0.636	311	5,848.00	2.984 FIX	2.309 FIX	1.548	successful	8	-	-	-	-
3	AUC/MIC	3	0.632	319	10,010.00	3.656	2.309 FIX	1 FIX	successful	8	-	-	-	-
4	AUC/MIC	4	0.658	307	3,520.00	2.168	2.309 FIX	2.398	successful	8	-	-	-	-
5	AUC/MIC	5	0.652	288	4,083.00	3.51	2.882	1 FIX	successful	8	-	-	-	-
6	AUC/MIC	6	0.665	285	2,859.00	2.608	2.731	1.97	successful	8	-	-	-	-
7	AUC/MIC	7	0.648	289	2,855.00	2.984 FIX	2.87	1 FIX	successful	8	-	-	-	-
8	AUC/MIC	8	0.662	284	3,375.00	2.984 FIX	2.774	1.534	successful	8	-	-	-	-
9	Cmax/MIC	1	0.521	328	1,325.00	2.984 FIX	2.309 FIX	1 FIX	successful	7	-	-	-	-
10	Cmax/MIC	2	0.510	325	1,105.00	2.984 FIX	2.309 FIX	1.441	successful	7	-	-	-	-
11	Cmax/MIC	3	0.510	329	1,978.00	3.792	2.309 FIX	1 FIX	successful	7	-	-	-	-
12	Cmax/MIC	4							unsuccessful	7	-	-	-	-
13	Cmax/MIC	5	0.525	316	855.30	3.423	2.749	1 FIX	successful	7	-	-	-	-
14	Cmax/MIC	6	0.533	316	419.80	2.077	2.593	3.808	successful	7	-	-	-	-
15	Cmax/MIC	7	0.523	315	641.30	2.984 FIX	2.752	1 FIX	successful	7	-	-	-	-
16	Cmax/MIC	8	0.528	316	698.60	2.984 FIX	2.671	1.3	successful	7	-	-	-	-
17	T>MIC	1	0.413	340	307.50	2.984 FIX	2.309 FIX	1 FIX	successful	8	-	-	-	-
18	T>MIC	2	0.602	269	98.84	2.984 FIX	2.309 FIX	40.53	successful	8	-	-	-	-
19	T>MIC	3							unsuccessful	8	-	-	-	-
20	T>MIC	4							unsuccessful	8	-	-	-	-
21	T>MIC	5							unsuccessful	8	-	-	-	-
22	T>MIC	6							unsuccessful	8	-	-	-	-
23	T>MIC	7	0.372	315	94.77	2.984 FIX	3.044	1 FIX	successful	8	-	-	-	-
24	T>MIC	8	0.604	256	97.69	2.984 FIX	2.572	33.23	successful	8	T>MIC	18154	25000	25000

Lepak et al. (2015) PMID:26259789 Drug:TXA-709

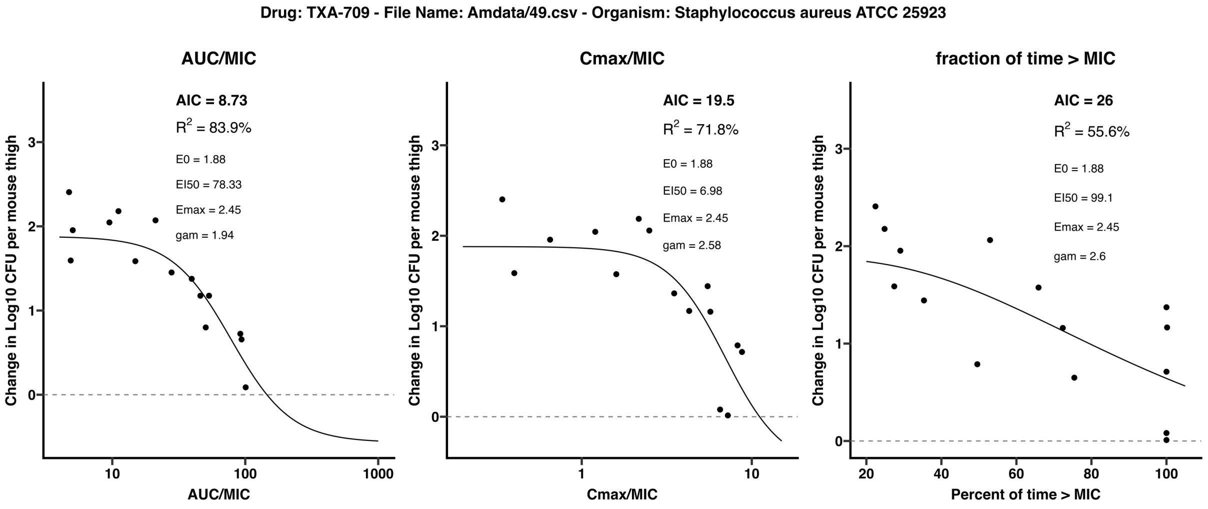

#	Model	Model Number	R²	AIC	EC50 hat	Emax hat	E0 hat	Gamma hat	Status	Best Fit Model	Optimal PKPD Index	Target for Stasis	Log1 Kill	Log2 Kill
1	AUC/MIC	1	0.840	14.20	108.200	2.447 FIX	1.88 FIX	1 FIX	successful	2	-	-	-	-
2	AUC/MIC	2	0.839	8.73	78.330	2.447 FIX	1.88 FIX	1.935	successful	2	AUC/MIC	145.5	1000	1000
3	AUC/MIC	3							unsuccessful	2	-	-	-	-
4	AUC/MIC	4	0.844	10.00	49.190	1.591	1.88 FIX	3.019	successful	2	-	-	-	-
5	AUC/MIC	5	0.846	8.86	265.100	6.55	2.183	1 FIX	successful	2	-	-	-	-
6	AUC/MIC	6	0.851	10.40	53.940	2.076	2.044	2.003	successful	2	-	-	-	-
7	AUC/MIC	7							unsuccessful	2	-	-	-	-
8	AUC/MIC	8							unsuccessful	2	-	-	-	-
9	Cmax/MIC	1	0.679	23.70	10.330	2.447 FIX	1.88 FIX	1 FIX	successful	2	-	-	-	-
10	Cmax/MIC	2	0.718	19.50	6.977	2.447 FIX	1.88 FIX	2.577	successful	2	-	-	-	-
11	Cmax/MIC	3							unsuccessful	2	-	-	-	-
12	Cmax/MIC	4							unsuccessful	2	-	-	-	-
13	Cmax/MIC	5	0.698	22.10	121.300	27.1	2.202	1 FIX	successful	2	-	-	-	-
14	Cmax/MIC	6							unsuccessful	2	-	-	-	-
15	Cmax/MIC	7							unsuccessful	2	-	-	-	-
16	Cmax/MIC	8							unsuccessful	2	-	-	-	-
17	T>MIC	1	0.581	28.30	155.900	2.447 FIX	1.88 FIX	1 FIX	successful	2	-	-	-	-
18	T>MIC	2	0.556	26.00	99.100	2.447 FIX	1.88 FIX	2.605	successful	2	-	-	-	-
19	T>MIC	3							unsuccessful	2	-	-	-	-
20	T>MIC	4							unsuccessful	2	-	-	-	-
21	T>MIC	5	0.582	26.90	74.460	4.182	3.089	1 FIX	successful	2	-	-	-	-
22	T>MIC	6							unsuccessful	2	-	-	-	-
23	T>MIC	7							unsuccessful	2	-	-	-	-
24	T>MIC	8	0.576	27.10	58.690	2.447 FIX	2.465	1.754	successful	2	-	-	-	-

Andes and Craig (2006) PMID:16377693 Drug:XRP 2868 (Streptograrnin)

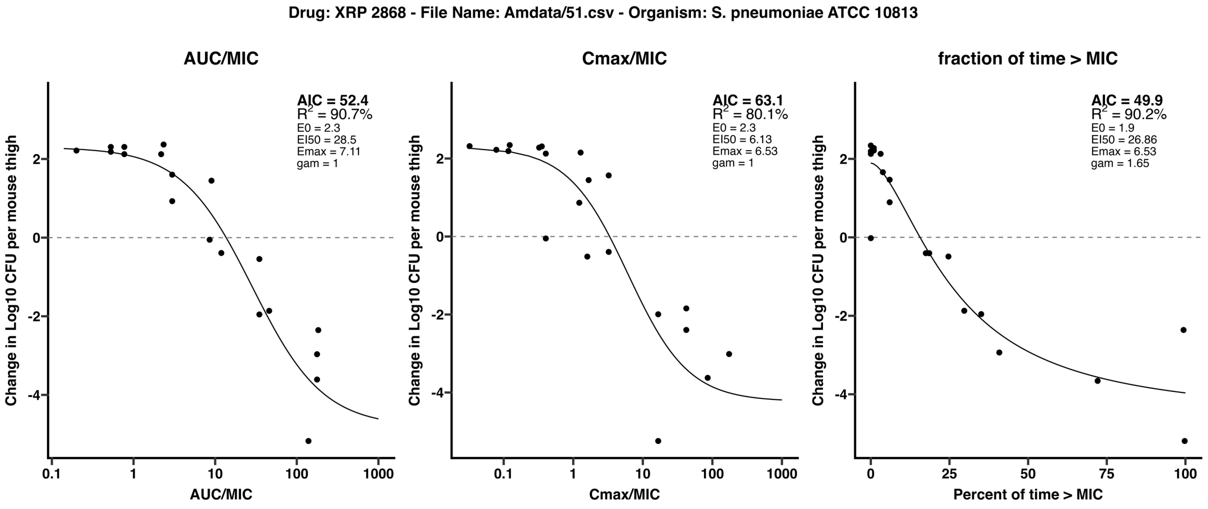

#	Model	Model Number	R²	AIC	EC50 hat	Emax hat	E0 hat	Gamma hat	Status	Best Fit Model	Optimal PKPD Index	Target for Stasis	Log1 Kill	Log2 Kill
1	AUC/MIC	1	0.906	51.4	22.520	6.53 FIX	2.3 FIX	1 FIX	successful	3	-	-	-	-
2	AUC/MIC	2	0.906	52.6	22.800	6.53 FIX	2.3 FIX	1.149	successful	3	-	-	-	-
3	AUC/MIC	3	0.907	52.4	28.500	7.106	2.3 FIX	1 FIX	successful	3	-	-	-	-
4	AUC/MIC	4	0.907	54.4	26.970	6.963	2.3 FIX	1.045	successful	3	-	-	-	-
5	AUC/MIC	5	0.907	54.3	27.140	7.127	2.369	1 FIX	successful	3	-	-	-	-
6	AUC/MIC	6	0.907	56.3	27.000	7.098	2.362	1.008	successful	3	-	-	-	-
7	AUC/MIC	7	0.906	53.4	22.810	6.53 FIX	2.285	1 FIX	successful	3	-	-	-	-
8	AUC/MIC	8	0.906	54.5	24.330	6.53 FIX	2.222	1.161	successful	3	-	-	-	-
9	Cmax/MIC	1	0.801	63.1	6.129	6.53 FIX	2.3 FIX	1 FIX	successful	1	-	-	-	-
10	Cmax/MIC	2	0.797	64.9	6.453	6.53 FIX	2.3 FIX	0.8871	successful	1	-	-	-	-
11	Cmax/MIC	3	0.805	63.9	4.624	5.849	2.3 FIX	1 FIX	successful	1	-	-	-	-
12	Cmax/MIC	4	0.812	65.4	3.864	5.539	2.3 FIX	1.337	successful	1	-	-	-	-
13	Cmax/MIC	5	0.805	65.9	4.518	5.874	2.334	1 FIX	successful	1	-	-	-	-
14	Cmax/MIC	6	0.815	66.8	4.219	5.049	1.934	1.838	successful	1	-	-	-	-
15	Cmax/MIC	7	0.805	64.8	4.913	6.53 FIX	2.521	1 FIX	successful	1	-	-	-	-
16	Cmax/MIC	8	0.800	66.6	4.973	6.53 FIX	2.556	0.8704	successful	1	-	-	-	-
17	T>MIC	1	0.861	53.8	20.990	6.53 FIX	2.3 FIX	1 FIX	successful	8	-	-	-	-
18	T>MIC	2	0.896	50.8	22.050	6.53 FIX	2.3 FIX	1.569	successful	8	-	-	-	-
19	T>MIC	3	0.888	51.1	39.830	8.874	2.3 FIX	1 FIX	successful	8	-	-	-	-
20	T>MIC	4	0.897	52.3	26.330	7.277	2.3 FIX	1.337	successful	8	-	-	-	-
21	T>MIC	5	0.889	52.4	46.130	8.976	2.069	1 FIX	successful	8	-	-	-	-
22	T>MIC	6	0.903	51.9	25.530	6.226	1.857	1.803	successful	8	-	-	-	-
23	T>MIC	7	0.874	54.7	25.680	6.53 FIX	1.995	1 FIX	successful	8	-	-	-	-
24	T>MIC	8	0.902	49.9	26.860	6.53 FIX	1.896	1.652	successful	8	T>MIC	13.64	24.74	43.64

Andes and Craig (2006) PMID:16377693 Drug:XRP 2868 (Streptograrnin)

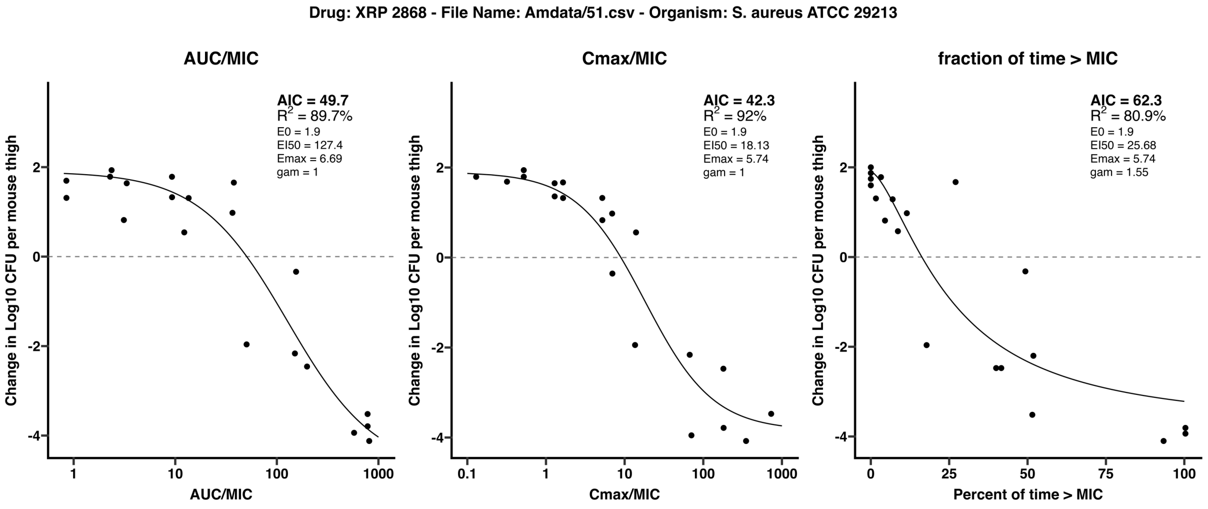

#	Model	Model Number	R²	AIC	EC50 hat	Emax hat	E0 hat	Gamma hat	Status	Best Fit Model	Optimal PKPD Index	Target for Stasis	Log1 Kill	Log2 Kill
1	AUC/MIC	1	0.885	50.2	84.79	5.743 FIX	1.9 FIX	1 FIX	successful	3	-	-	-	-
2	AUC/MIC	2	0.889	51.5	86.27	5.743 FIX	1.9 FIX	1.181	successful	3	-	-	-	-
3	AUC/MIC	3	0.897	49.7	127.40	6.69	1.9 FIX	1 FIX	successful	3	-	-	-	-
4	AUC/MIC	4	0.896	51.3	176.50	7.482	1.9 FIX	0.8354	successful	3	-	-	-	-
5	AUC/MIC	5	0.898	50.9	154.40	6.658	1.668	1 FIX	successful	3	-	-	-	-
6	AUC/MIC	6	0.898	52.9	145.70	6.449	1.635	1.058	successful	3	-	-	-	-
7	AUC/MIC	7	0.894	50.9	112.60	5.743 FIX	1.598	1 FIX	successful	3	-	-	-	-
8	AUC/MIC	8	0.897	51.2	121.20	5.743 FIX	1.52	1.275	successful	3	-	-	-	-
9	Cmax/MIC	1	0.920	42.3	18.13	5.743 FIX	1.9 FIX	1 FIX	successful	1	Cmax/MIC	9	18.5	38.4
10	Cmax/MIC	2	0.922	43.5	17.10	5.743 FIX	1.9 FIX	1.172	successful	1	-	-	-	-
11	Cmax/MIC	3	0.919	44.2	19.23	5.884	1.9 FIX	1 FIX	successful	1	-	-	-	-
12	Cmax/MIC	4	0.923	45.3	15.57	5.564	1.9 FIX	1.27	successful	1	-	-	-	-
13	Cmax/MIC	5	0.920	46.1	18.13	5.943	1.984	1 FIX	successful	1	-	-	-	-
14	Cmax/MIC	6	0.923	47.2	15.69	5.398	1.79	1.374	successful	1	-	-	-	-
15	Cmax/MIC	7	0.920	44.3	17.77	5.743 FIX	1.92	1 FIX	successful	1	-	-	-	-
16	Cmax/MIC	8	0.922	45.5	16.92	5.743 FIX	1.912	1.172	successful	1	-	-	-	-
17	T>MIC	1	0.789	63.2	22.78	5.743 FIX	1.9 FIX	1 FIX	successful	2	-	-	-	-
18	T>MIC	2	0.809	62.3	25.68	5.743 FIX	1.9 FIX	1.546	successful	2	-	-	-	-
19	T>MIC	3	0.832	59.5	76.93	10.4	1.9 FIX	1 FIX	successful	2	-	-	-	-
20	T>MIC	4	0.832	61.4	141.00	13.89	1.9 FIX	0.8613	successful	2	-	-	-	-
21	T>MIC	5	0.832	61.3	88.75	10.88	1.76	1 FIX	successful	2	-	-	-	-
22	T>MIC	6	0.832	63.3	105.20	11.89	1.782	0.9489	successful	2	-	-	-	-
23	T>MIC	7	0.799	64.9	26.33	5.743 FIX	1.707	1 FIX	successful	2	-	-	-	-
24	T>MIC	8	0.819	63.0	34.51	5.743 FIX	1.458	1.799	successful	2	-	-	-	-

Vogelman et al. (1988) PMID:3139779 Drug:Tobramycin

Drug: Tobramycin - File Name: Amdata/52.csv - Organism: P. aeruginosa ATCC 27853

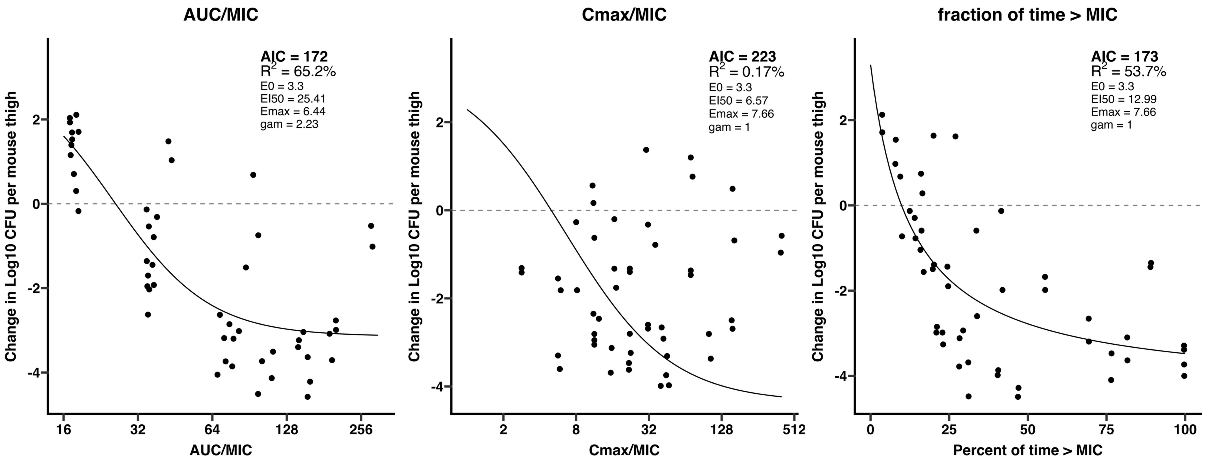

#	Model	Model Number	R²	AIC	EC50 hat	Emax hat	E0 hat	Gamma hat	Status	Best Fit Model	Optimal PKPD Index	Target for Stasis	Log1 Kill	Log2 Kill
1	AUC/MIC	1	0.592000000	178	30.650000	7.658 FIX	3.3 FIX	1 FIX	successful	4	-	-	-	-
2	AUC/MIC	2	0.612000000	177	32.610000	7.658 FIX	3.3 FIX	1.337	successful	4	-	-	-	-
3	AUC/MIC	3	0.589000000	180	32.250000	7.807	3.3 FIX	1 FIX	successful	4	-	-	-	-
4	AUC/MIC	4	0.652000000	172	25.410000	6.441	3.3 FIX	2.23	successful	4	AUC/MIC	26	34.7	50.6
5	AUC/MIC	5							unsuccessful	4	-	-	-	-
6	AUC/MIC	6	0.653000000	174	29.200000	5.567	2.47	2.599	successful	4	-	-	-	-
7	AUC/MIC	7	0.599000000	180	26.780000	7.658 FIX	3.533	1 FIX	successful	4	-	-	-	-
8	AUC/MIC	8	0.651000000	173	21.330000	7.658 FIX	4.467	1.944	successful	4	-	-	-	-
9	Cmax/MIC	1	0.001730000	223	6.570000	7.658 FIX	3.3 FIX	1 FIX	successful	1	-	-	-	-
10	Cmax/MIC	2							unsuccessful	1	-	-	-	-
11	Cmax/MIC	3							unsuccessful	1	-	-	-	-
12	Cmax/MIC	4							unsuccessful	1	-	-	-	-
13	Cmax/MIC	5							unsuccessful	1	-	-	-	-
14	Cmax/MIC	6							unsuccessful	1	-	-	-	-
15	Cmax/MIC	7	0.000000191	188	-0.001073	7.658 FIX	5.81	1 FIX	successful	1	-	-	-	-
16	Cmax/MIC	8							unsuccessful	1	-	-	-	-
17	T>MIC	1	0.537000000	173	12.990000	7.658 FIX	3.3 FIX	1 FIX	successful	1	-	-	-	-
18	T>MIC	2							unsuccessful	1	-	-	-	-
19	T>MIC	3	0.537000000	175	13.160000	7.691	3.3 FIX	1 FIX	successful	1	-	-	-	-
20	T>MIC	4							unsuccessful	1	-	-	-	-
21	T>MIC	5	0.545000000	176	8.001000	9.442	5.329	1 FIX	successful	1	-	-	-	-
22	T>MIC	6	0.566000000	176	15.450000	4.998	1.839	2.664	successful	1	-	-	-	-
23	T>MIC	7	0.540000000	175	11.720000	7.658 FIX	3.466	1 FIX	successful	1	-	-	-	-
24	T>MIC	8	0.553000000	175	10.360000	7.658 FIX	4.004	1.388	successful	1	-	-	-	-

Vogelman et al. (1988) PMID:3139779 Drug:Ticarcillin

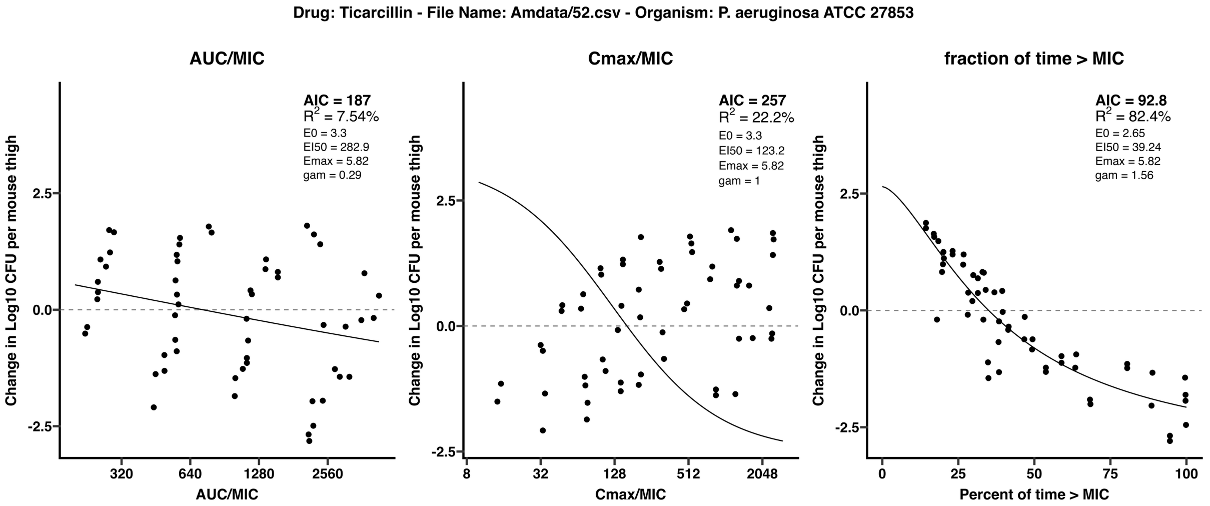

#	Model	Model Number	R²	AIC	EC50 hat	Emax hat	E0 hat	Gamma hat	Status	Best Fit Model	Optimal PKPD Index	Target for Stasis	Log1 Kill	Log2 Kill
1	AUC/MIC	1	0.0783	203.0	572.200	5.82 FIX	3.3 FIX	1 FIX	successful	2	-	-	-	-
2	AUC/MIC	2	0.0754	187.0	282.900	5.82 FIX	3.3 FIX	0.2863	successful	2	-	-	-	-
3	AUC/MIC	3	0.0758	187.0	107.400	3.903	3.3 FIX	1 FIX	successful	2	-	-	-	-
4	AUC/MIC	4	0.0775	189.0	87.350	4.211	3.3 FIX	0.6406	successful	2	-	-	-	-
5	AUC/MIC	5	0.0785	189.0	430.400	2	1.224	1 FIX	successful	2	-	-	-	-
6	AUC/MIC	6	0.0795	191.0	709.600	1.234	0.6205	1.792	successful	2	-	-	-	-
7	AUC/MIC	7	0.0744	187.0	61.220	5.82 FIX	5.251	1 FIX	successful	2	-	-	-	-
8	AUC/MIC	8							unsuccessful	2	-	-	-	-
9	Cmax/MIC	1	0.2220	257.0	123.200	5.82 FIX	3.3 FIX	1 FIX	successful	1	-	-	-	-
10	Cmax/MIC	2	0.2050	165.0	570.300	5.82 FIX	3.3 FIX	-0.2501	successful	1	-	-	-	-
11	Cmax/MIC	3	0.1580	169.0	-6.100	2.97	3.3 FIX	1 FIX	successful	1	-	-	-	-
12	Cmax/MIC	4							unsuccessful	1	-	-	-	-
13	Cmax/MIC	5	0.2330	165.0	46.990	-2.716	-1.993	1 FIX	successful	1	-	-	-	-
14	Cmax/MIC	6	0.2340	167.0	62.770	-2.29	-1.614	1.222	successful	1	-	-	-	-
15	Cmax/MIC	7	0.1710	168.0	-4.087	5.82 FIX	6.196	1 FIX	successful	1	-	-	-	-
16	Cmax/MIC	8							unsuccessful	1	-	-	-	-
17	T>MIC	1	0.8200	115.0	24.930	5.82 FIX	3.3 FIX	1 FIX	successful	8	-	-	-	-
18	T>MIC	2	0.8160	93.4	28.500	5.82 FIX	3.3 FIX	1.634	successful	8	-	-	-	-
19	T>MIC	3	0.8210	91.7	56.080	8.534	3.3 FIX	1 FIX	successful	8	-	-	-	-
20	T>MIC	4	0.8230	92.9	38.410	7.035	3.3 FIX	1.241	successful	8	-	-	-	-
21	T>MIC	5	0.8230	93.0	39.340	8.492	3.982	1 FIX	successful	8	-	-	-	-
22	T>MIC	6	0.8240	94.7	39.240	5.341	2.407	1.737	successful	8	-	-	-	-
23	T>MIC	7							unsuccessful	8	-	-	-	-
24	T>MIC	8	0.8240	92.8	39.240	5.82 FIX	2.648	1.557	successful	8	T>MIC	35	55	95

He et al. (2023) PMID:36845018 Drug:LYSC98

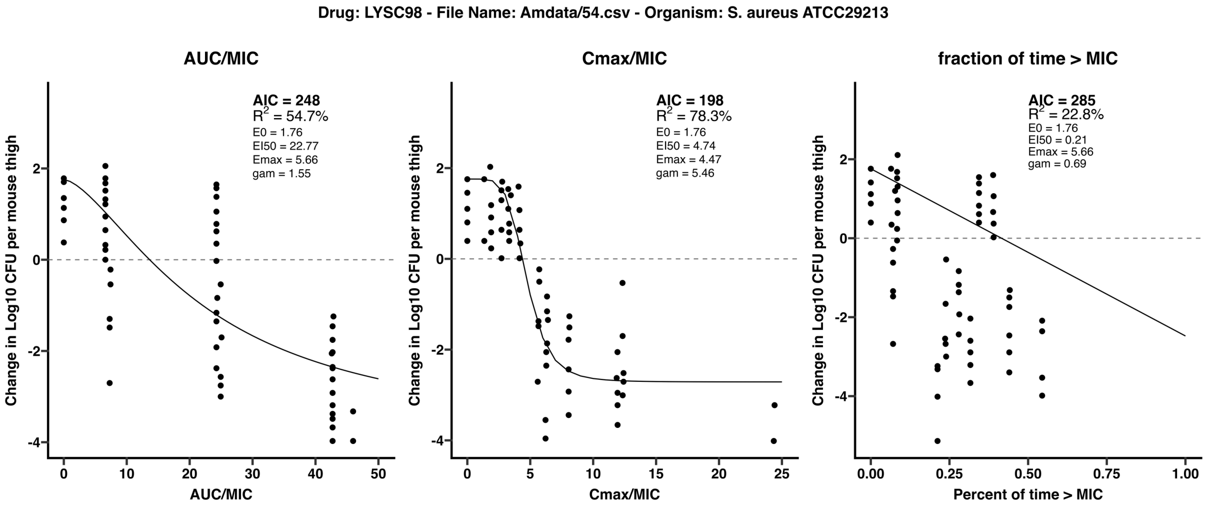

#	Model	Model Number	R²	AIC	EC50 hat	Emax hat	E0 hat	Gamma hat	Status	Best Fit Model	Optimal PKPD Index	Target for Stasis	Log1 Kill	Log2 Kill
1	AUC/MIC	1	0.513	250	20.38000	5.658 FIX	1.759 FIX	1 FIX	successful	2	-			
2	AUC/MIC	2	0.547	248	22.77000	5.658 FIX	1.759 FIX	1.552	successful	2	-			
3	AUC/MIC	3							unsuccessful	2	-			
4	AUC/MIC	4							unsuccessful	2	-			
5	AUC/MIC	5							unsuccessful	2	-			
6	AUC/MIC	6							unsuccessful	2	-			
7	AUC/MIC	7							unsuccessful	2	-			
8	AUC/MIC	8	0.646	230	37.42000	5.658 FIX	0.5873	3.567	successful	2	-			
9	Cmax/MIC	1	0.647	226	5.86000	5.658 FIX	1.759 FIX	1 FIX	successful	4	-			
10	Cmax/MIC	2	0.731	206	5.66400	5.658 FIX	1.759 FIX	2.159	successful	4	-			
11	Cmax/MIC	3	0.670	219	12.18000	8.476	1.759 FIX	1 FIX	successful	4	-			
12	Cmax/MIC	4	0.783	198	4.74300	4.471	1.759 FIX	5.458	successful	4	Cmax/MIC	4	5	7
13	Cmax/MIC	5	0.670	221	10.97000	8.433	1.934	1 FIX	successful	4	-			
14	Cmax/MIC	6	0.805	185	5.28000	3.579	1.018	12.25	successful	4	-			
15	Cmax/MIC	7	0.655	228	6.56000	5.658 FIX	1.596	1 FIX	successful	4	-			
16	Cmax/MIC	8							unsuccessful	4	-			
17	T>MIC	1	0.229	285	0.21260	5.658 FIX	1.759 FIX	1 FIX	successful	2	-			
18	T>MIC	2	0.228	285	0.20750	5.658 FIX	1.759 FIX	0.6935	successful	2	-			
19	T>MIC	3	0.233	284	0.09542	4.245	1.759 FIX	1 FIX	successful	2	-			
20	T>MIC	4							unsuccessful	2	-			
21	T>MIC	5	0.234	286	0.11890	3.95	1.381	1 FIX	successful	2	-			
22	T>MIC	6							unsuccessful	2	-			
23	T>MIC	7							unsuccessful	2	-			
24	T>MIC	8							unsuccessful	2	-			

van den Berg et al. (2025) PMID:39804217 Drug:NOSO-502

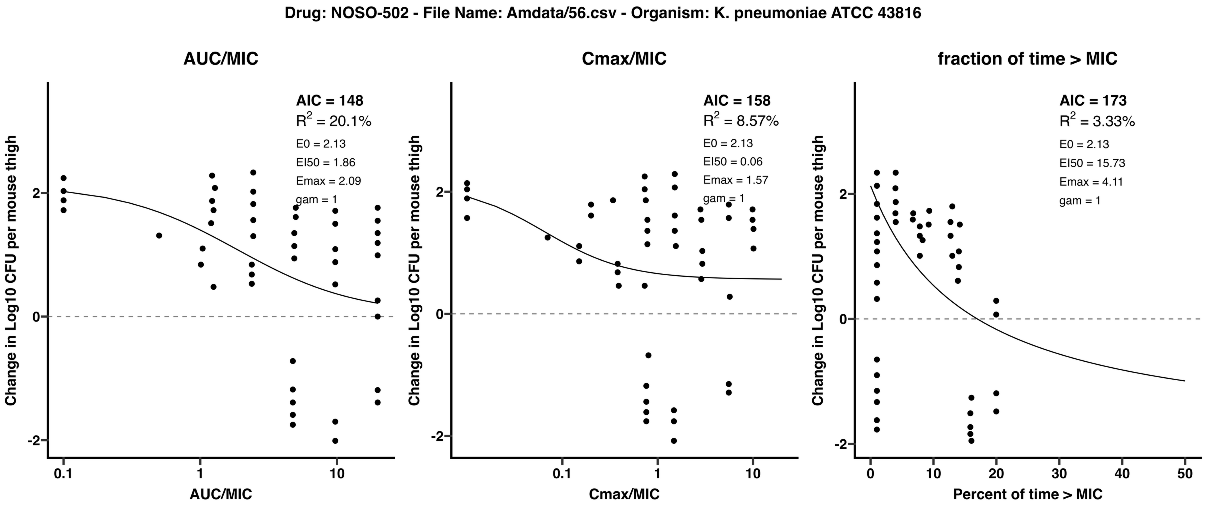

#	Model	Model Number	R²	AIC	EC50 hat	Emax hat	E0 hat	Gamma hat	Status	Best Fit Model	Optimal PKPD Index	Target for Stasis	Log1 Kill	Log2 Kill
1	AUC/MIC	1	0.14400	155	11.530000	4.105 FIX	2.13 FIX	1 FIX	successful	3	-			
2	AUC/MIC	2	0.17200	150	22.020000	4.105 FIX	2.13 FIX	0.4362	successful	3	-			
3	AUC/MIC	3	0.20100	148	1.864000	2.094	2.13 FIX	1 FIX	successful	3	AUC/MIC	20	20	20
4	AUC/MIC	4	0.22600	149	2.030000	1.883	2.13 FIX	2.444	successful	3	-			
5	AUC/MIC	5	0.20100	150	1.789000	2.13	2.171	1 FIX	successful	3	-			
6	AUC/MIC	6							unsuccessful	3	-			
7	AUC/MIC	7	0.11900	153	0.072660	4.105 FIX	4.691	1 FIX	successful	3	-			
8	AUC/MIC	8							unsuccessful	3	-			
9	Cmax/MIC	1	0.00357	177	4.159000	4.105 FIX	2.13 FIX	1 FIX	successful	3	-			
10	Cmax/MIC	2	0.03950	160	115.200000	4.105 FIX	2.13 FIX	0.15	successful	3	-			
11	Cmax/MIC	3	0.08570	158	0.064760	1.566	2.13 FIX	1 FIX	successful	3	-			
12	Cmax/MIC	4							unsuccessful	3	-			
13	Cmax/MIC	5	0.08570	160	0.063550	1.578	2.143	1 FIX	successful	3	-			
14	Cmax/MIC	6							unsuccessful	3	-			
15	Cmax/MIC	7	0.07770	158	0.004721	4.105 FIX	4.765	1 FIX	successful	3	-			
16	Cmax/MIC	8							unsuccessful	3	-			
17	T>MIC	1	0.03330	173	15.730000	4.105 FIX	2.13 FIX	1 FIX	successful	1	-			
18	T>MIC	2							unsuccessful	1	-			
19	T>MIC	3	0.00366	166	-0.126800	1.458	2.13 FIX	1 FIX	successful	1	-			
20	T>MIC	4							unsuccessful	1	-			
21	T>MIC	5							unsuccessful	1	-			
22	T>MIC	6							unsuccessful	1	-			
23	T>MIC	7	0.06520	164	72.500000	4.105 FIX	0.9734	1 FIX	successful	1	-			
24	T>MIC	8							unsuccessful	1	-			

van den Berg et al. (2025) PMID:39804217 Drug:NOSO-502

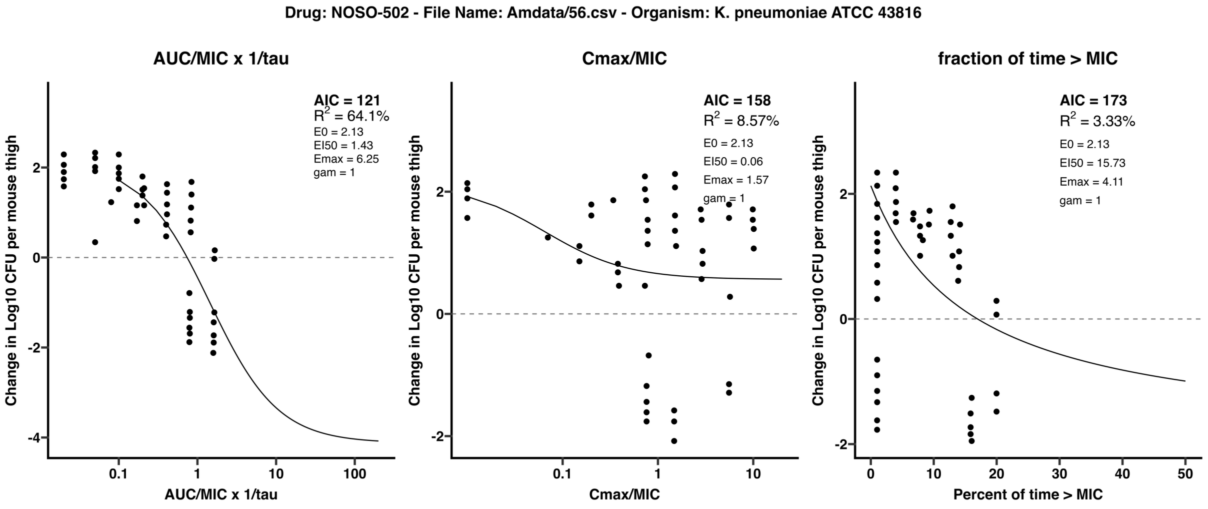

#	Model	Model Number	R²	AIC	EC50 hat	Emax hat	E0 hat	Gamma hat	Status	Best Fit Model	Optimal PKPD Index	Target for Stasis	Log1 Kill	Log2 Kill
1	AUC/MIC	1	0.62500	121	0.664500	4.105 FIX	2.13 FIX	1 FIX	successful	3	-			
2	AUC/MIC	2	0.63900	121	0.654600	4.105 FIX	2.13 FIX	1.363	successful	3	-			
3	AUC/MIC	3	0.64100	121	1.431000	6.252	2.13 FIX	1 FIX	successful	3	AUC/MIC	0.7	1.4	2.8
4	AUC/MIC	4							unsuccessful	3	-			
5	AUC/MIC	5	0.64100	123	1.691000	6.635	2.043	1 FIX	successful	3	-			
6	AUC/MIC	6							unsuccessful	3	-			
7	AUC/MIC	7	0.62600	123	0.681900	4.105 FIX	2.106	1 FIX	successful	3	-			
8	AUC/MIC	8	0.64500	122	0.812700	4.105 FIX	1.868	1.563	successful	3	-			
9	Cmax/MIC	1	0.00357	177	4.159000	4.105 FIX	2.13 FIX	1 FIX	successful	3	-			
10	Cmax/MIC	2	0.03950	160	115.200000	4.105 FIX	2.13 FIX	0.15	successful	3	-			
11	Cmax/MIC	3	0.08570	158	0.064760	1.566	2.13 FIX	1 FIX	successful	3	-			
12	Cmax/MIC	4							unsuccessful	3	-			
13	Cmax/MIC	5	0.08570	160	0.063550	1.578	2.143	1 FIX	successful	3	-			
14	Cmax/MIC	6							unsuccessful	3	-			
15	Cmax/MIC	7	0.07770	158	0.004721	4.105 FIX	4.765	1 FIX	successful	3	-			
16	Cmax/MIC	8							unsuccessful	3	-			
17	T>MIC	1	0.03330	173	15.730000	4.105 FIX	2.13 FIX	1 FIX	successful	1	-			
18	T>MIC	2							unsuccessful	1	-			
19	T>MIC	3	0.00366	166	-0.126800	1.458	2.13 FIX	1 FIX	successful	1	-			
20	T>MIC	4							unsuccessful	1	-			
21	T>MIC	5							unsuccessful	1	-			
22	T>MIC	6							unsuccessful	1	-			
23	T>MIC	7	0.06520	164	72.500000	4.105 FIX	0.9734	1 FIX	successful	1	-			
24	T>MIC	8							unsuccessful	1	-			

Eguchi et al. (2009) PMID:19487438 Drug:SMP-601 (PTZ601) Razupenem

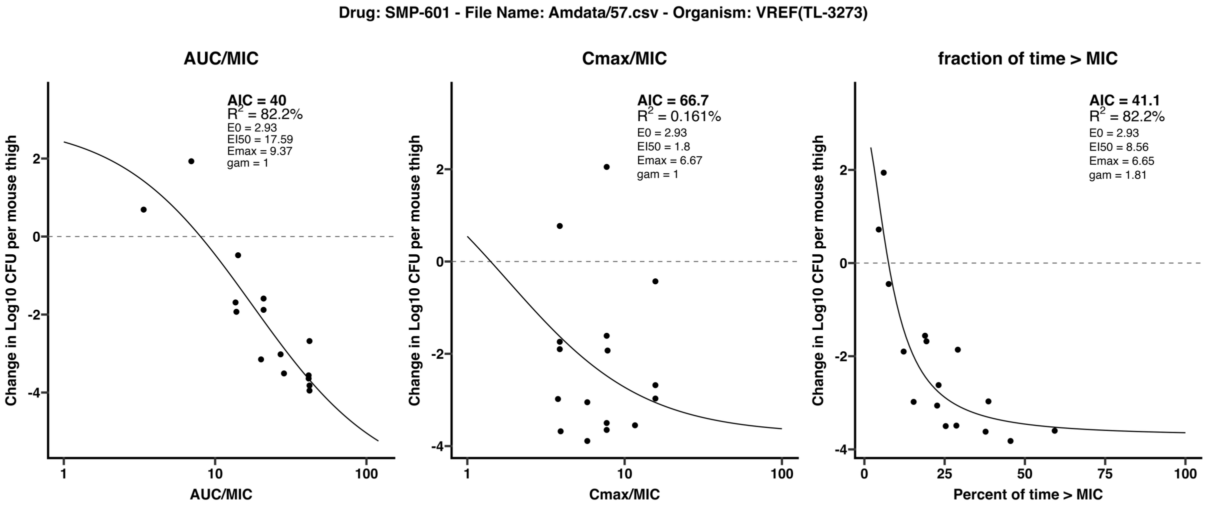

#	Model	Model Number	R²	AIC	EC50 hat	Emax hat	E0 hat	Gamma hat	Status	Best Fit Model	Optimal PKPD Index	Target for Stasis	Log1 Kill	Log2 Kill
1	AUC/MIC	1	0.79100	47.4	6.5300	6.672 FIX	2.93 FIX	1 FIX	successful	3	-			
2	AUC/MIC	2	0.82500	40.8	10.6600	6.672 FIX	2.93 FIX	2.101	successful	3	-			
3	AUC/MIC	3	0.82200	40.0	17.5900	9.37	2.93 FIX	1 FIX	successful	3	AUC/MIC	8	12.7	19.5
4	AUC/MIC	4	0.83000	41.5	12.5200	7.751	2.93 FIX	1.403	successful	3	-			
5	AUC/MIC	5	0.82200	42.0	16.6700	9.41	3.067	1 FIX	successful	3	-			
6	AUC/MIC	6							unsuccessful	3	-			
7	AUC/MIC	7	0.82200	45.3	15.6900	6.672 FIX	1.612	1 FIX	successful	3	-			
8	AUC/MIC	8	0.84300	40.2	14.1500	6.672 FIX	2.146	1.771	successful	3	-			
9	Cmax/MIC	1	0.00161	66.7	1.7980	6.672 FIX	2.93 FIX	1 FIX	successful	1	-			
10	Cmax/MIC	2							unsuccessful	1	-			
11	Cmax/MIC	3	0.00207	67.4	0.2022	5.266	2.93 FIX	1 FIX	successful	1	-			
12	Cmax/MIC	4							unsuccessful	1	-			
13	Cmax/MIC	5							unsuccessful	1	-			
14	Cmax/MIC	6							unsuccessful	1	-			
15	Cmax/MIC	7	0.00209	67.4	0.1585	6.672 FIX	4.337	1 FIX	successful	1	-			
16	Cmax/MIC	8							unsuccessful	1	-			
17	T>MIC	1	0.81400	45.5	6.2190	6.672 FIX	2.93 FIX	1 FIX	successful	4	-			
18	T>MIC	2							unsuccessful	4	-			
19	T>MIC	3	0.79500	41.6	12.4200	8.408	2.93 FIX	1 FIX	successful	4	-			
20	T>MIC	4	0.82200	41.1	8.5630	6.65	2.93 FIX	1.807	successful	4	-			
21	T>MIC	5	0.81700	41.5	4.0160	12.6	8.059	1 FIX	successful	4	-			
22	T>MIC	6							unsuccessful	4	-			
23	T>MIC	7	0.79700	45.0	12.0000	6.672 FIX	1.948	1 FIX	successful	4	-			
24	T>MIC	8							unsuccessful	4	-			

Eguchi et al. (2009) PMID:19487438 Drug:SMP-601 (PTZ601) Razupenem

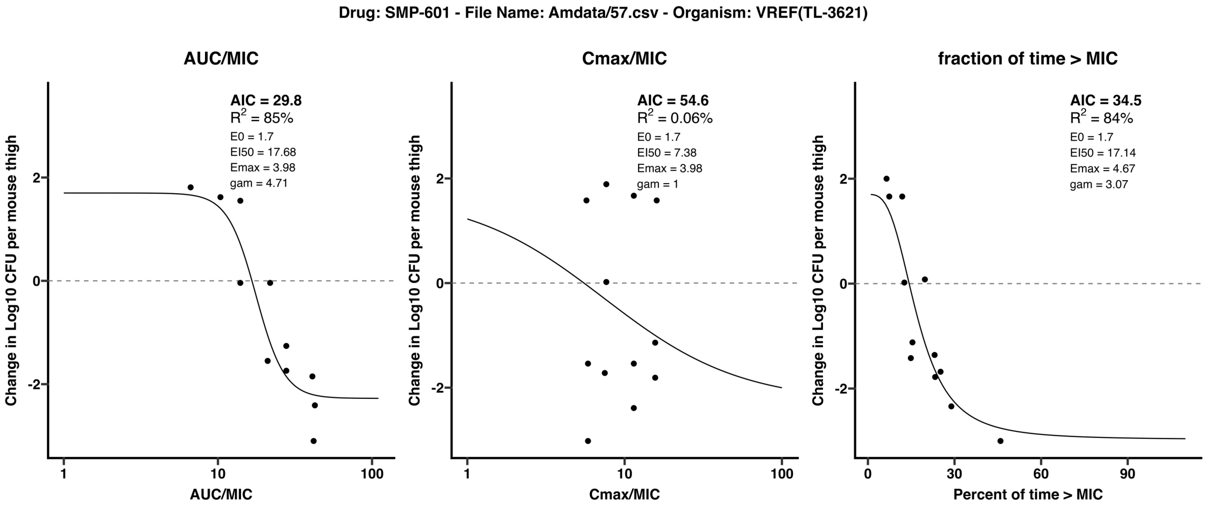

#	Model	Model Number	R²	AIC	EC50 hat	Emax hat	E0 hat	Gamma hat	Status	Best Fit Model	Optimal PKPD Index	Target for Stasis	Log1 Kill	Log2 Kill
1	AUC/MIC	1	0.827000	43.8	13.3600	3.975 FIX	1.7 FIX	1 FIX	successful	2	-			
2	AUC/MIC	2	0.850000	29.8	17.6800	3.975 FIX	1.7 FIX	4.706	successful	2	AUC/MIC	16.6	20.7	30.7
3	AUC/MIC	3	0.760000	36.7	340.6000	39.68	1.7 FIX	1 FIX	successful	2	-			
4	AUC/MIC	4	0.850000	31.7	18.0400	4.11	1.7 FIX	4.382	successful	2	-			
5	AUC/MIC	5	0.828000	33.3	16.4600	11.13	5.429	1 FIX	successful	2	-			
6	AUC/MIC	6	0.853000	33.3	17.3100	4.597	2.085	3.703	successful	2	-			
7	AUC/MIC	7							unsuccessful	2	-			
8	AUC/MIC	8	0.850000	31.8	17.5700	3.975 FIX	1.721	4.726	successful	2	-			
9	Cmax/MIC	1	0.000588	54.6	7.3790	3.975 FIX	1.7 FIX	1 FIX	successful	1	-			
10	Cmax/MIC	2							unsuccessful	1	-			
11	Cmax/MIC	3	0.001620	55.9	-0.7349	2.005	1.7 FIX	1 FIX	successful	1	-			
12	Cmax/MIC	4							unsuccessful	1	-			
13	Cmax/MIC	5							unsuccessful	1	-			
14	Cmax/MIC	6							unsuccessful	1	-			
15	Cmax/MIC	7	0.001500	55.9	-0.3762	3.975 FIX	3.664	1 FIX	successful	1	-			
16	Cmax/MIC	8							unsuccessful	1	-			
17	T>MIC	1	0.857000	45.1	13.9800	3.975 FIX	1.7 FIX	1 FIX	successful	4	-			
18	T>MIC	2							unsuccessful	4	-			
19	T>MIC	3	0.770000	38.5	275.5000	36.52	1.7 FIX	1 FIX	successful	4	-			
20	T>MIC	4	0.840000	34.5	17.1400	4.672	1.7 FIX	3.067	successful	4	-			
21	T>MIC	5	0.857000	32.6	12.0100	11.57	6.111	1 FIX	successful	4	-			
22	T>MIC	6							unsuccessful	4	-			
23	T>MIC	7							unsuccessful	4	-			
24	T>MIC	8	0.834000	34.9	14.2100	3.975 FIX	1.837	4.195	successful	4	-			

Eguchi et al. (2009) PMID:19487438 Drug:SMP-601 (PTZ601) Razupenem

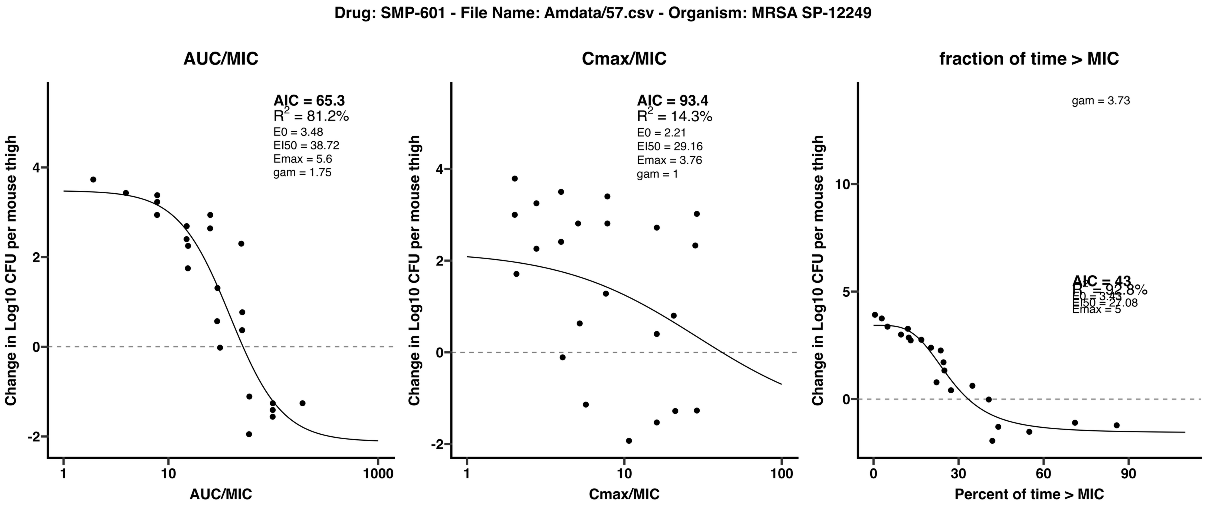

#	Model	Model Number	R²	AIC	EC50 hat	Emax hat	E0 hat	Gamma hat	Status	Best Fit Model	Optimal PKPD Index	Target for Stasis	Log1 Kill	Log2 Kill
1	AUC/MIC	1	0.800	80.2	61.0500	3.755 FIX	2.21 FIX	1 FIX	successful	6	-			
2	AUC/MIC	2	0.771	69.1	47.3200	3.755 FIX	2.21 FIX	3.703	successful	6	-			
3	AUC/MIC	3	0.720	77.6	268.1000	11.02	2.21 FIX	1 FIX	successful	6	-			
4	AUC/MIC	4	0.770	71.1	48.1100	3.847	2.21 FIX	3.62	successful	6	-			
5	AUC/MIC	5	0.801	64.5	49.6300	7.996	4.224	1 FIX	successful	6	-			
6	AUC/MIC	6	0.812	65.3	38.7200	5.598	3.482	1.753	successful	6	-			
7	AUC/MIC	7	0.788	78.7	28.6400	3.755 FIX	3.039	1 FIX	successful	6	-			
8	AUC/MIC	8	0.793	67.1	34.9500	3.755 FIX	2.82	3.162	successful	6	-			
9	Cmax/MIC	1	0.143	93.4	29.1600	3.755 FIX	2.21 FIX	1 FIX	successful	1	-			
10	Cmax/MIC	2	0.141	95.4	27.9000	3.755 FIX	2.21 FIX	1.053	successful	1	-			
11	Cmax/MIC	3	0.157	95.3	17.4300	2.76	2.21 FIX	1 FIX	successful	1	-			
12	Cmax/MIC	4							unsuccessful	1	-			
13	Cmax/MIC	5	0.207	95.4	0.5668	12.44	12.72	1 FIX	successful	1	-			
14	Cmax/MIC	6							unsuccessful	1	-			
15	Cmax/MIC	7	0.197	93.8	3.6440	3.755 FIX	3.858	1 FIX	successful	1	-			
16	Cmax/MIC	8	0.212	95.2	2.9410	3.755 FIX	4.264	1.848	successful	1	-			
17	T>MIC	1	0.865	83.2	53.0700	3.755 FIX	2.21 FIX	1 FIX	successful	6	-			
18	T>MIC	2	0.872	62.7	32.1600	3.755 FIX	2.21 FIX	5.966	successful	6	-			
19	T>MIC	3							unsuccessful	6	-			
20	T>MIC	4							unsuccessful	6	-			
21	T>MIC	5	0.867	55.0	65.9700	11.72	4.467	1 FIX	successful	6	-			
22	T>MIC	6	0.928	43.0	27.0800	4.996	3.43	3.733	successful	6	T>MIC	33	47	110
23	T>MIC	7	0.802	79.8	20.5700	3.755 FIX	3.284	1 FIX	successful	6	-			
24	T>MIC	8	0.915	49.5	25.7400	3.755 FIX	2.996	6.158	successful	6	-			

Eguchi et al. (2009) PMID:19487438 Drug:SMP-601 (PTZ601) Razupenem

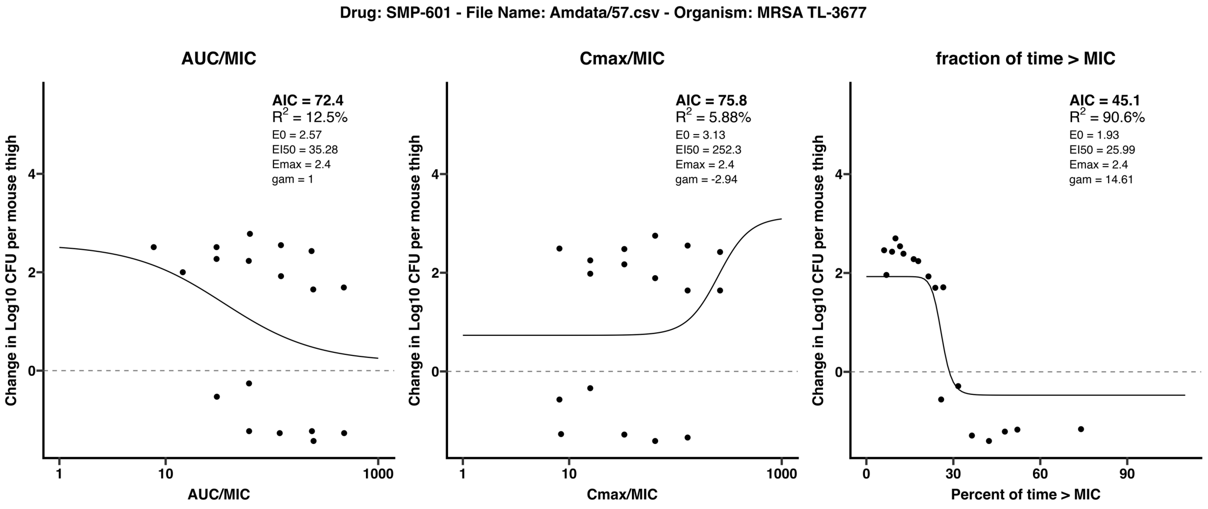

#	Model	Model Number	R²	AIC	EC50 hat	Emax hat	E0 hat	Gamma hat	Status	Best Fit Model	Optimal PKPD Index	Target for Stasis	Log1 Kill	Log2 Kill
1	AUC/MIC	1	0.0964	71.6	827.000	2.4 FIX	1.1 FIX	1 FIX	successful	7	-			
2	AUC/MIC	2	0.0830	73.5	546.100	2.4 FIX	1.1 FIX	1.508	successful	7	-			
3	AUC/MIC	3	0.0865	73.6	18,140.000	39.25	1.1 FIX	1 FIX	successful	7	-			
4	AUC/MIC	4							unsuccessful	7	-			
5	AUC/MIC	5	0.1270	74.3	20.510	3.067	3.286	1 FIX	successful	7	-			
6	AUC/MIC	6							unsuccessful	7	-			
7	AUC/MIC	7	0.1250	72.4	35.280	2.4 FIX	2.57	1 FIX	successful	7	-			
8	AUC/MIC	8	0.1250	74.4	30.300	2.4 FIX	2.674	1.17	successful	7	-			
9	Cmax/MIC	1							unsuccessful	8	-			
10	Cmax/MIC	2							unsuccessful	8	-			
11	Cmax/MIC	3	0.0267	74.4	-6.906	0.1061	1.1 FIX	1 FIX	successful	8	-			
12	Cmax/MIC	4							unsuccessful	8	-			
13	Cmax/MIC	5							unsuccessful	8	-			
14	Cmax/MIC	6							unsuccessful	8	-			
15	Cmax/MIC	7	0.0333	74.3	-2.203	2.4 FIX	3.605	1 FIX	successful	8	-			
16	Cmax/MIC	8	0.0588	75.8	252.300	2.4 FIX	3.132	-2.939	successful	8	-			
17	T>MIC	1	0.7920	67.9	80.180	2.4 FIX	1.1 FIX	1 FIX	successful	8	-			
18	T>MIC	2	0.8420	56.6	30.840	2.4 FIX	1.1 FIX	15.97	successful	8	-			
19	T>MIC	3							unsuccessful	8	-			
20	T>MIC	4							unsuccessful	8	-			
21	T>MIC	5	0.7930	47.9	64.910	11.32	3.943	1 FIX	successful	8	-			
22	T>MIC	6							unsuccessful	8	-			
23	T>MIC	7							unsuccessful	8	-			
24	T>MIC	8	0.9060	45.1	25.990	2.4 FIX	1.927	14.61	successful	8	T>MIC	29	110	110

Andes et al. (2002) PMID:12384354 Drug:Linezolid

Drug: Linezolid - File Name: Amdata/58.csv - Organism: S. pneumoniae ATCC 10813

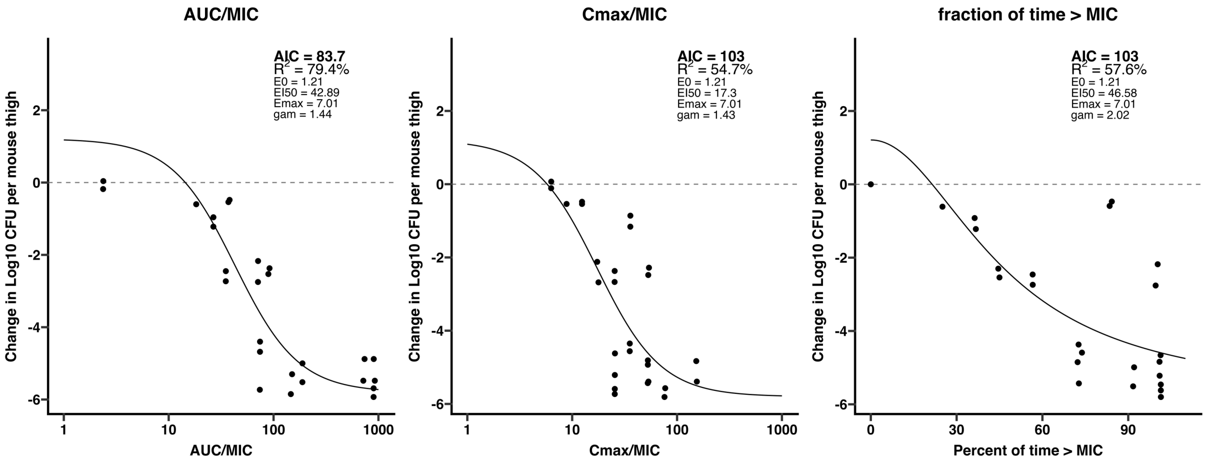

#	Model	Model Number	R²	AIC	EC50 hat	Emax hat	E0 hat	Gamma hat	Status	Best Fit Model	Optimal PKPD Index	Target for Stasis	Log1 Kill	Log2 Kill
1	AUC/MIC	1	0.764	84.2	38.860	7.01 FIX	1.21 FIX	1 FIX	successful	2	-			
2	AUC/MIC	2	0.794	83.7	42.890	7.01 FIX	1.21 FIX	1.436	successful	2	AUC/MIC	14.4	25	38.1
3	AUC/MIC	3	0.768	85.8	43.250	7.301	1.21 FIX	1 FIX	successful	2	-			
4	AUC/MIC	4	0.794	85.5	41.270	6.826	1.21 FIX	1.529	successful	2	-			
5	AUC/MIC	5	0.769	87.5	48.000	6.998	0.8734	1 FIX	successful	2	-			
6	AUC/MIC	6	0.814	83.4	55.250	5.339	-0.1411	2.228	successful	2	-			
7	AUC/MIC	7	0.769	85.5	47.930	7.01 FIX	0.8826	1 FIX	successful	2	-			
8	AUC/MIC	8	0.796	85.5	46.460	7.01 FIX	1.04	1.405	successful	2	-			
9	Cmax/MIC	1	0.541	103.0	15.180	7.01 FIX	1.21 FIX	1 FIX	successful	2	-			
10	Cmax/MIC	2	0.547	103.0	17.300	7.01 FIX	1.21 FIX	1.426	successful	2	-			
11	Cmax/MIC	3	0.531	104.0	20.200	7.791	1.21 FIX	1 FIX	successful	2	-			
12	Cmax/MIC	4	0.556	104.0	14.560	6.171	1.21 FIX	2.025	successful	2	-			
13	Cmax/MIC	5	0.552	105.0	6.988	11.69	5.843	1 FIX	successful	2	-			
14	Cmax/MIC	6	0.565	106.0	17.460	3.943	-0.3176	19.82	successful	2	-			
15	Cmax/MIC	7	0.537	105.0	17.170	7.01 FIX	1.015	1 FIX	successful	2	-			
16	Cmax/MIC	8	0.556	104.0	13.280	7.01 FIX	1.845	1.676	successful	2	-			
17	T>MIC	1	0.480	106.0	33.790	7.01 FIX	1.21 FIX	1 FIX	successful	2	-			
18	T>MIC	2	0.576	103.0	46.580	7.01 FIX	1.21 FIX	2.021	successful	2	-			
19	T>MIC	3	0.576	103.0	199.900	17.75	1.21 FIX	1 FIX	successful	2	-			
20	T>MIC	4	0.578	105.0	76.280	9.968	1.21 FIX	1.349	successful	2	-			
21	T>MIC	5	0.584	104.0	2,975.000	152.7	0.2304	1 FIX	successful	2	-			
22	T>MIC	6	0.589	105.0	64.150	6.621	0.09335	2.102	successful	2	-			
23	T>MIC	7							unsuccessful	2	-			
24	T>MIC	8							unsuccessful	2	-			

Andes et al. (2002) PMID:12384354 Drug:Linezolid

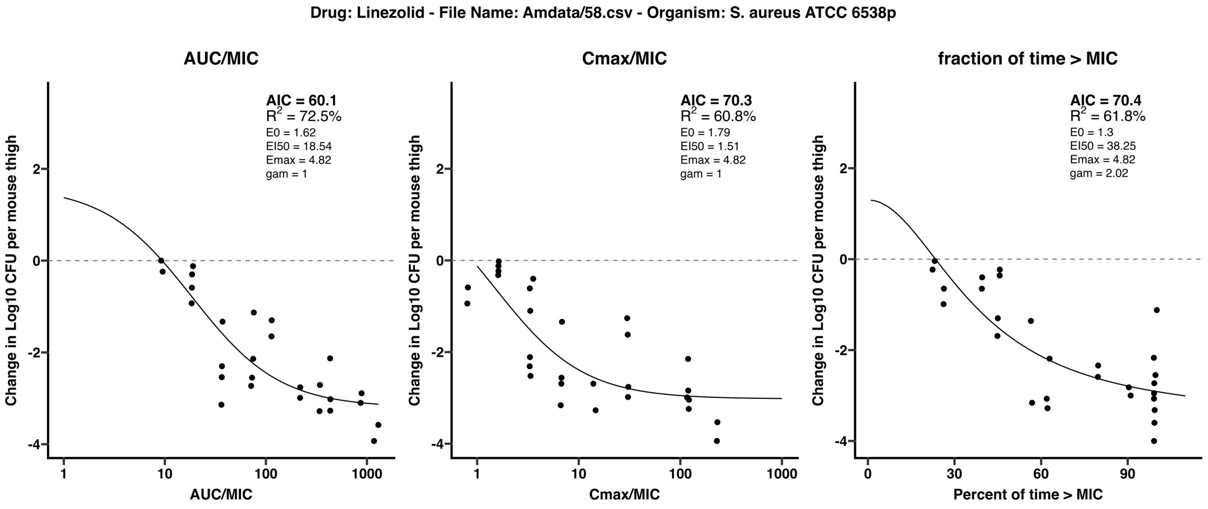

#	Model	Model Number	R²	AIC	EC50 hat	Emax hat	E0 hat	Gamma hat	Status	Best Fit Model	Optimal PKPD Index	Target for Stasis	Log1 Kill	Log2 Kill
1	AUC/MIC	1	0.722	60.2	26.230	4.815 FIX	1.3 FIX	1 FIX	successful	7	-			
2	AUC/MIC	2	0.723	60.4	24.710	4.815 FIX	1.3 FIX	0.7923	successful	7	-			
3	AUC/MIC	3	0.724	60.2	21.490	4.524	1.3 FIX	1 FIX	successful	7	-			
4	AUC/MIC	4	0.725	62.2	22.170	4.605	1.3 FIX	0.9192	successful	7	-			
5	AUC/MIC	5	0.725	62.1	17.140	4.996	1.81	1 FIX	successful	7	-			
6	AUC/MIC	6							unsuccessful	7	-			
7	AUC/MIC	7	0.725	60.1	18.540	4.815 FIX	1.619	1 FIX	successful	7	AUC/MIC	9.4	22.1	56.1
8	AUC/MIC	8							unsuccessful	7	-			
9	Cmax/MIC	1	0.615	72.0	2.568	4.815 FIX	1.3 FIX	1 FIX	successful	7	-			
10	Cmax/MIC	2	0.603	70.5	2.265	4.815 FIX	1.3 FIX	0.6273	successful	7	-			
11	Cmax/MIC	3	0.613	69.8	1.847	4.323	1.3 FIX	1 FIX	successful	7	-			
12	Cmax/MIC	4	0.613	71.8	1.849	4.371	1.3 FIX	0.9234	successful	7	-			
13	Cmax/MIC	5	0.615	71.6	2.454	3.812	0.7533	1 FIX	successful	7	-			
14	Cmax/MIC	6	0.648	71.0	3.510	2.447	-0.3198	3.558	successful	7	-			
15	Cmax/MIC	7	0.608	70.3	1.509	4.815 FIX	1.795	1 FIX	successful	7	-			
16	Cmax/MIC	8							unsuccessful	7	-			
17	T>MIC	1	0.610	77.1	27.500	4.815 FIX	1.3 FIX	1 FIX	successful	2	-			
18	T>MIC	2	0.618	70.4	38.250	4.815 FIX	1.3 FIX	2.023	successful	2	-			
19	T>MIC	3	0.605	71.4	90.640	8.206	1.3 FIX	1 FIX	successful	2	-			
20	T>MIC	4	0.619	72.3	40.570	5.069	1.3 FIX	1.828	successful	2	-			
21	T>MIC	5	0.612	72.8	39.640	8.147	2.86	1 FIX	successful	2	-			
22	T>MIC	6							unsuccessful	2	-			
23	T>MIC	7							unsuccessful	2	-			
24	T>MIC	8	0.622	72.1	45.220	4.815 FIX	0.9599	1.908	successful	2	-			

Van Wart et al. (2009) PMID:19249182 Drug:Doripenem

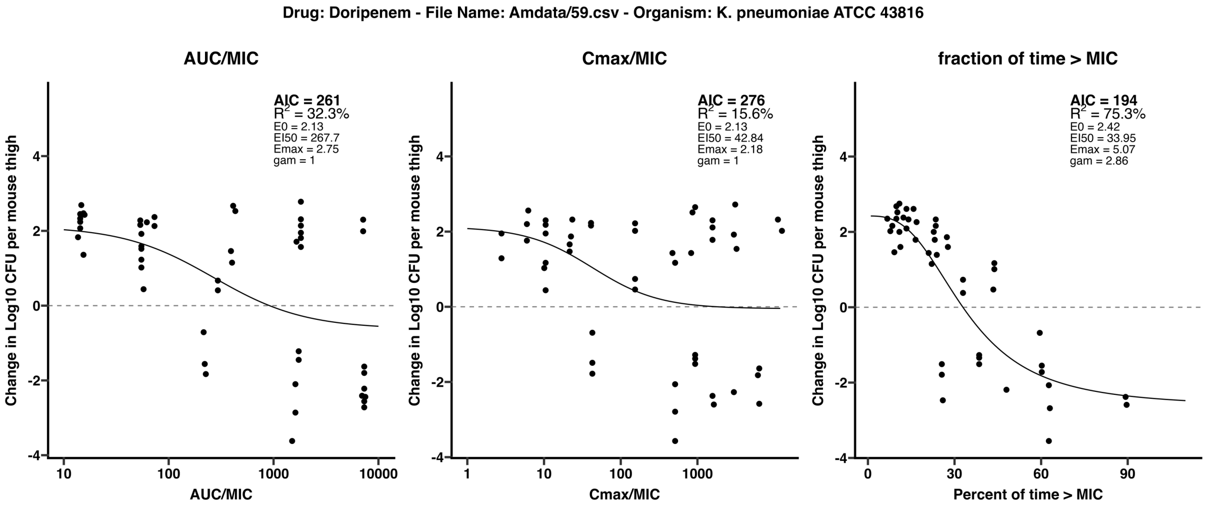

#	Model	Model Number	R²	AIC	EC50 hat	Emax hat	E0 hat	Gamma hat	Status	Best Fit Model	Optimal PKPD Index	Target for Stasis	Log1 Kill	Log2 Kill
1	AUC/MIC	1	0.2900	266	3,107.000	5.07 FIX	2.13 FIX	1 FIX	successful	3	-			
2	AUC/MIC	2	0.3240	261	3,460.000	5.07 FIX	2.13 FIX	0.5058	successful	3	-			
3	AUC/MIC	3	0.3230	261	267.700	2.753	2.13 FIX	1 FIX	successful	3	-			
4	AUC/MIC	4	0.3270	263	620.500	3.352	2.13 FIX	0.6984	successful	3	-			
5	AUC/MIC	5	0.3250	263	164.000	2.939	2.419	1 FIX	successful	3	-			
6	AUC/MIC	6							unsuccessful	3	-			
7	AUC/MIC	7	0.2780	266	5,707.000	5.07 FIX	1.646	1 FIX	successful	3	-			
8	AUC/MIC	8	0.3350	262	246.400	5.07 FIX	3.378	0.4116	successful	3	-			
9	Cmax/MIC	1	0.0692	297	2,317.000	5.07 FIX	2.13 FIX	1 FIX	successful	3	-			
10	Cmax/MIC	2	0.1150	279	10,200.000	5.07 FIX	2.13 FIX	0.2415	successful	3	-			
11	Cmax/MIC	3	0.1560	276	42.840	2.18	2.13 FIX	1 FIX	successful	3	-			
12	Cmax/MIC	4							unsuccessful	3	-			
13	Cmax/MIC	5	0.1560	278	47.620	2.116	2.059	1 FIX	successful	3	-			
14	Cmax/MIC	6	0.1560	280	48.250	1.995	1.976	1.185	successful	3	-			
15	Cmax/MIC	7	0.1140	280	3.648	5.07 FIX	5.234	1 FIX	successful	3	-			
16	Cmax/MIC	8							unsuccessful	3	-			
17	T>MIC	1	0.7350	236	54.850	5.07 FIX	2.13 FIX	1 FIX	successful	8	-			
18	T>MIC	2	0.7490	194	37.970	5.07 FIX	2.13 FIX	2.983	successful	8	-			
19	T>MIC	3							unsuccessful	8	-			
20	T>MIC	4							unsuccessful	8	-			
21	T>MIC	5	0.7410	197	109.300	14.51	3.503	1 FIX	successful	8	-			
22	T>MIC	6	0.7540	196	36.530	5.639	2.512	2.47	successful	8	-			
23	T>MIC	7							unsuccessful	8	-			
24	T>MIC	8	0.7530	194	33.950	5.07 FIX	2.422	2.862	successful	8	T>MIC	33	44	66

Basarab et al. (2015) PMID:26168713 Drug:ETX0914

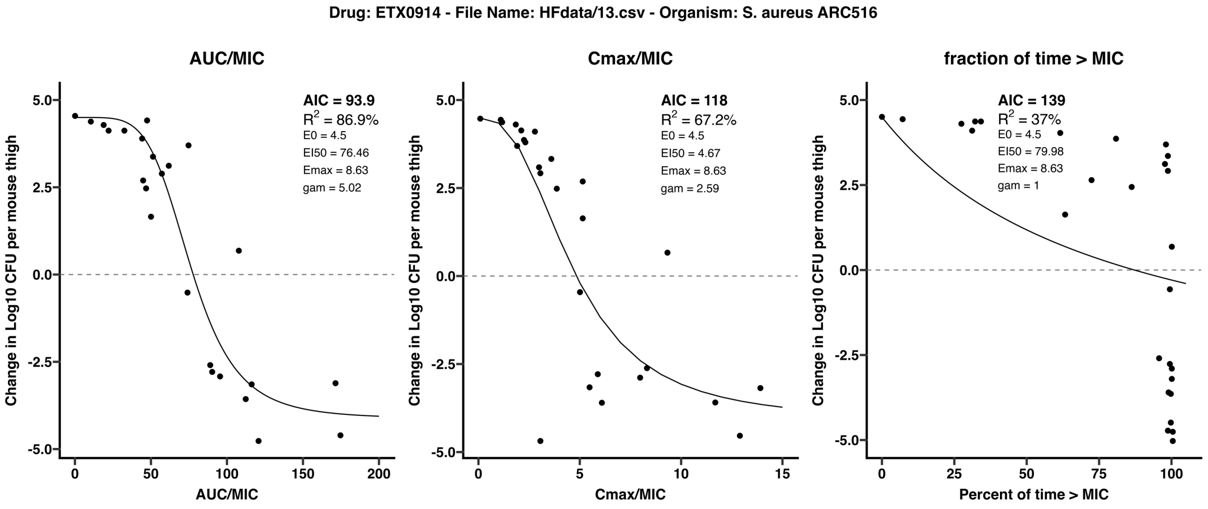

#	Model	Model Number	R²	AIC	EC50 hat	Emax hat	E0 hat	Gamma hat	Status	Best Fit Model	Optimal PKPD Index	Target for Stasis	Log1 Kill	Log2 Kill
1	AUC/MIC	1	0.684	127.0	84.730	8.628 FIX	4.5 FIX	1 FIX	successful	2	-			
2	AUC/MIC	2	0.869	93.9	76.460	8.628 FIX	4.5 FIX	5.017	successful	2	AUC/MIC	78	86	96
3	AUC/MIC	3							unsuccessful	2	-			
4	AUC/MIC	4	0.869	95.8	77.300	8.798	4.5 FIX	4.854	successful	2	-			
5	AUC/MIC	5	0.771	111.0	691.200	58.89	6.35	1 FIX	successful	2	-			
6	AUC/MIC	6	0.872	97.0	78.720	7.804	3.952	6.821	successful	2	-			
7	AUC/MIC	7	0.663	129.0	72.070	8.628 FIX	4.897	1 FIX	successful	2	-			
8	AUC/MIC	8	0.870	95.4	79.540	8.628 FIX	4.227	5.217	successful	2	-			
9	Cmax/MIC	1	0.635	126.0	5.257	8.628 FIX	4.5 FIX	1 FIX	successful	2	-			
10	Cmax/MIC	2	0.672	118.0	4.673	8.628 FIX	4.5 FIX	2.59	successful	2	-			
11	Cmax/MIC	3	0.631	122.0	29.130	27.82	4.5 FIX	1 FIX	successful	2	-			
12	Cmax/MIC	4	0.673	120.0	4.429	8.179	4.5 FIX	2.82	successful	2	-			
13	Cmax/MIC	5	0.647	122.0	9.845	17.93	6.237	1 FIX	successful	2	-			
14	Cmax/MIC	6	0.673	122.0	4.388	8.422	4.659	2.677	successful	2	-			
15	Cmax/MIC	7	0.624	128.0	4.273	8.628 FIX	4.98	1 FIX	successful	2	-			
16	Cmax/MIC	8	0.673	120.0	4.418	8.628 FIX	4.733	2.57	successful	2	-			
17	T>MIC	1	0.370	139.0	79.980	8.628 FIX	4.5 FIX	1 FIX	successful	1	-			
18	T>MIC	2							unsuccessful	1	-			
19	T>MIC	3							unsuccessful	1	-			
20	T>MIC	4							unsuccessful	1	-			
21	T>MIC	5							unsuccessful	1	-			
22	T>MIC	6							unsuccessful	1	-			
23	T>MIC	7	0.357	141.0	65.560	8.628 FIX	4.958	1 FIX	successful	1	-			
24	T>MIC	8	0.632	126.0	99.140	8.628 FIX	3.346	140.8	successful	1	-			

O'Donnell et al. (2024) PMID:38092676 Drug:Durlobactam

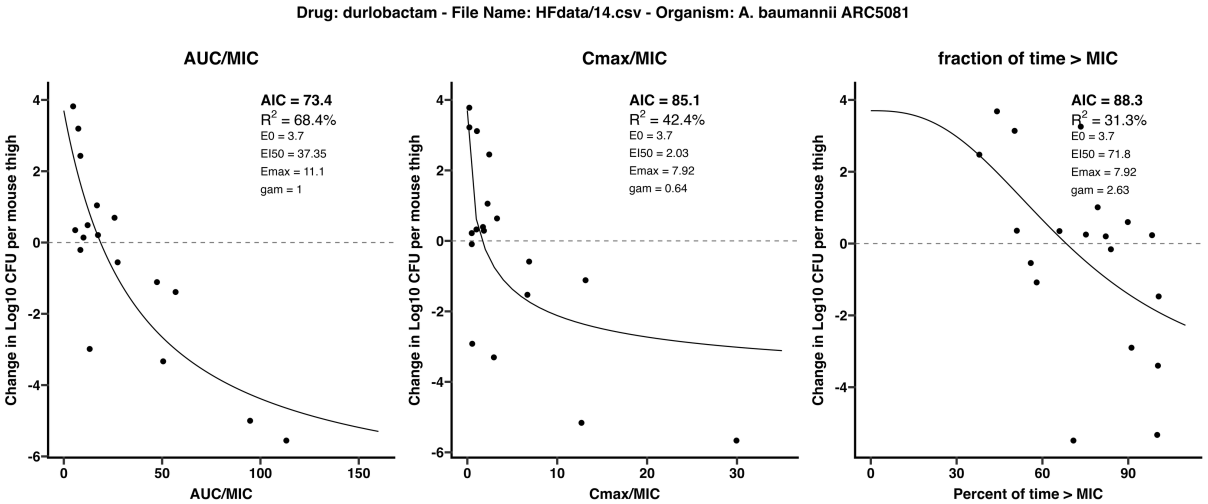

#	Model	Model Number	R²	AIC	EC50 hat	Emax hat	E0 hat	Gamma hat	Status	Best Fit Model	Optimal PKPD Index	Target for Stasis	Log1 Kill	Log2 Kill
1	AUC/MIC	1	0.665	74.1	18.840	7.919 FIX	3.7 FIX	1 FIX	successful	3	-			
2	AUC/MIC	2							unsuccessful	3	-			
3	AUC/MIC	3	0.684	73.4	37.350	11.1	3.7 FIX	1 FIX	successful	3	AUC/MIC	19	27	39
4	AUC/MIC	4							unsuccessful	3	-			
5	AUC/MIC	5	0.697	74.5	130.800	16.74	2.387	1 FIX	successful	3	-			
6	AUC/MIC	6							unsuccessful	3	-			
7	AUC/MIC	7	0.678	75.6	29.310	7.919 FIX	2.911	1 FIX	successful	3	-			
8	AUC/MIC	8	0.675	75.9	44.550	7.919 FIX	1.892	1.541	successful	3	-			
9	Cmax/MIC	1	0.400	85.2	2.121	7.919 FIX	3.7 FIX	1 FIX	successful	2	-			
10	Cmax/MIC	2	0.424	85.1	2.028	7.919 FIX	3.7 FIX	0.6387	successful	2	-			
11	Cmax/MIC	3	0.380	87.1	1.603	7.231	3.7 FIX	1 FIX	successful	2	-			
12	Cmax/MIC	4							unsuccessful	2	-			
13	Cmax/MIC	5	0.502	84.2	26.920	13.26	1.315	1 FIX	successful	2	-			
14	Cmax/MIC	6							unsuccessful	2	-			
15	Cmax/MIC	7	0.489	82.8	10.630	7.919 FIX	1.472	1 FIX	successful	2	-			
16	Cmax/MIC	8	0.498	84.4	11.790	7.919 FIX	1.057	1.462	successful	2	-			
17	T>MIC	1	0.320	89.1	71.200	7.919 FIX	3.7 FIX	1 FIX	successful	2	-			
18	T>MIC	2	0.313	88.3	71.800	7.919 FIX	3.7 FIX	2.633	successful	2	-			
19	T>MIC	3							unsuccessful	2	-			
20	T>MIC	4							unsuccessful	2	-			
21	T>MIC	5	0.321	90.0	596.500	54.71	5.751	1 FIX	successful	2	-			
22	T>MIC	6							unsuccessful	2	-			
23	T>MIC	7							unsuccessful	2	-			
24	T>MIC	8	0.313	90.3	65.910	7.919 FIX	4.093	2.616	successful	2	-			

O'Donnell et al. (2024) PMID:38092676 Drug:Sulbactam

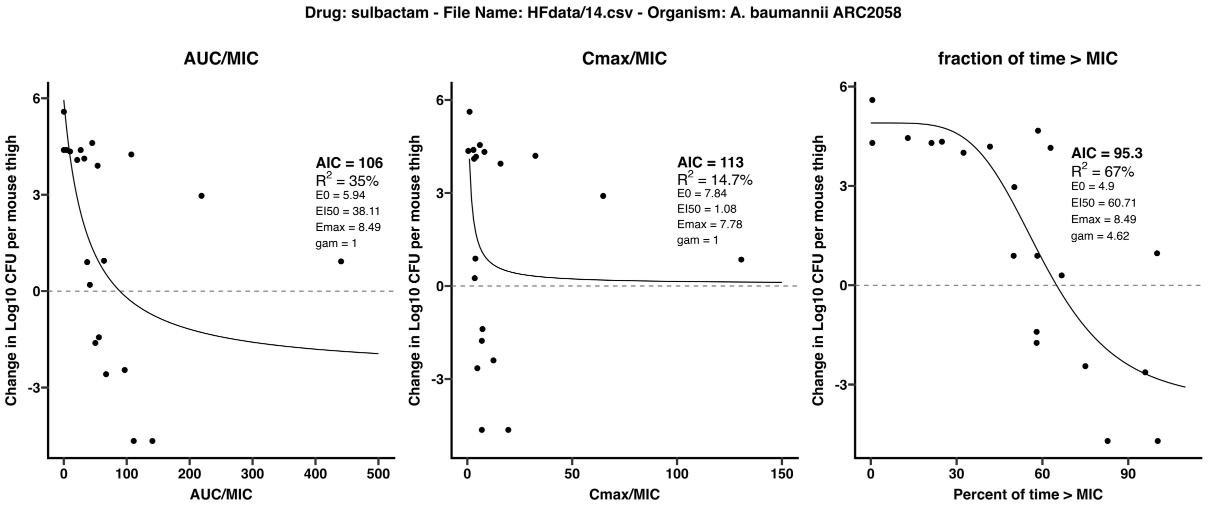

#	Model	Model Number	R²	AIC	EC50 hat	Emax hat	E0 hat	Gamma hat	Status	Best Fit Model	Optimal PKPD Index	Target for Stasis	Log1 Kill	Log2 Kill
1	AUC/MIC	1	0.32900	104.0	65.210	8.49 FIX	4.9 FIX	1 FIX	successful	7	-			
2	AUC/MIC	2	0.33100	106.0	64.540	8.49 FIX	4.9 FIX	1.042	successful	7	-			
3	AUC/MIC	3	0.35000	106.0	39.460	6.738	4.9 FIX	1 FIX	successful	7	-			
4	AUC/MIC	4							unsuccessful	7	-			
5	AUC/MIC	5	0.35100	108.0	33.550	7.353	5.528	1 FIX	successful	7	-			
6	AUC/MIC	6	0.44500	106.0	40.240	5.356	4.656	4.874	successful	7	-			
7	AUC/MIC	7	0.35000	106.0	38.110	8.49 FIX	5.945	1 FIX	successful	7	-			
8	AUC/MIC	8	0.39700	107.0	38.160	8.49 FIX	6.216	1.775	successful	7	-			
9	Cmax/MIC	1	0.06770	114.0	8.420	8.49 FIX	4.9 FIX	1 FIX	successful	5	-			
10	Cmax/MIC	2	0.06040	113.0	25.960	8.49 FIX	4.9 FIX	0.3025	successful	5	-			
11	Cmax/MIC	3	0.13800	112.0	2.109	4.827	4.9 FIX	1 FIX	successful	5	-			
12	Cmax/MIC	4							unsuccessful	5	-			
13	Cmax/MIC	5	0.14700	113.0	1.084	7.775	7.839	1 FIX	successful	5	-			
14	Cmax/MIC	6							unsuccessful	5	-			
15	Cmax/MIC	7	0.00178	115.0	1,643.000	8.49 FIX	1.621	1 FIX	successful	5	-			
16	Cmax/MIC	8							unsuccessful	5	-			
17	T>MIC	1	0.50700	105.0	67.780	8.49 FIX	4.9 FIX	1 FIX	successful	2	-			
18	T>MIC	2	0.67000	95.3	60.710	8.49 FIX	4.9 FIX	4.618	successful	2	T>MIC	65	73	83
19	T>MIC	3							unsuccessful	2	-			
20	T>MIC	4							unsuccessful	2	-			
21	T>MIC	5							unsuccessful	2	-			
22	T>MIC	6							unsuccessful	2	-			
23	T>MIC	7	0.48900	107.0	56.930	8.49 FIX	5.301	1 FIX	successful	2	-			
24	T>MIC	8	0.67100	97.2	62.380	8.49 FIX	4.686	4.672	successful	2	-			

Singh et al. (2015) PMID:26024868 Drug:Aztreonam/Avibactam

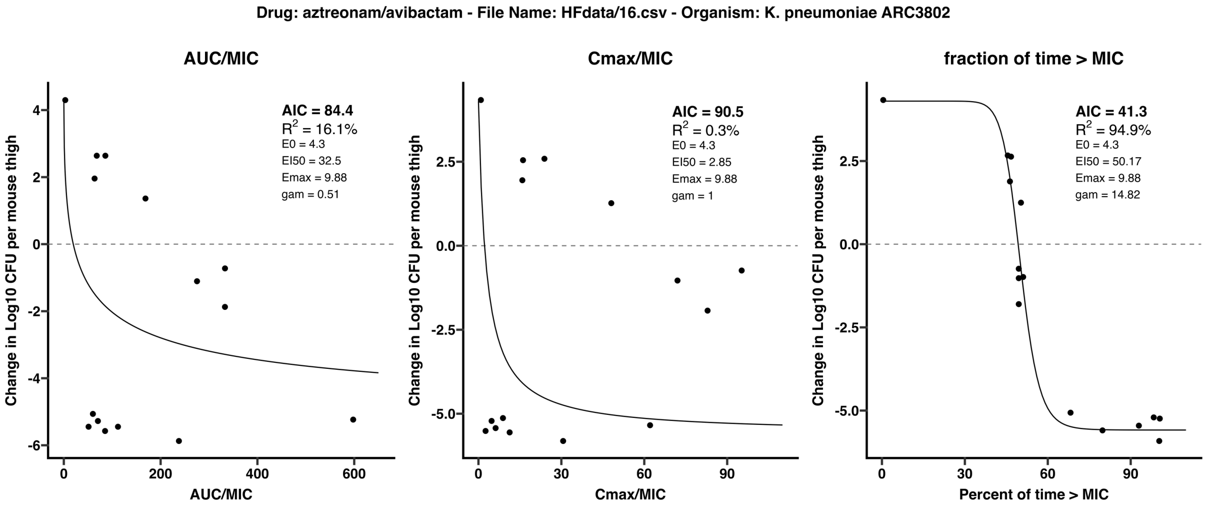

#	Model	Model Number	R²	AIC	EC50 hat	Emax hat	E0 hat	Gamma hat	Status	Best Fit Model	Optimal PKPD Index	Target for Stasis	Log1 Kill	Log2 Kill
1	AUC/MIC	1	0.14300	83.5	48.7200	9.885 FIX	4.3 FIX	1 FIX	successful	2	-			
2	AUC/MIC	2	0.16100	84.4	32.5000	9.885 FIX	4.3 FIX	0.5135	successful	2	-			
3	AUC/MIC	3	0.19900	83.8	11.5600	7.322	4.3 FIX	1 FIX	successful	2	-			
4	AUC/MIC	4							unsuccessful	2	-			
5	AUC/MIC	5							unsuccessful	2	-			
6	AUC/MIC	6							unsuccessful	2	-			
7	AUC/MIC	7	0.20700	83.5	5.9400	9.885 FIX	7.042	1 FIX	successful	2	-			
8	AUC/MIC	8							unsuccessful	2	-			
9	Cmax/MIC	1	0.00286	90.5	2.8460	9.885 FIX	4.3 FIX	1 FIX	successful	1	-			
10	Cmax/MIC	2							unsuccessful	1	-			
11	Cmax/MIC	3	0.33600	85.5	-1.6590	4.781	4.3 FIX	1 FIX	successful	1	-			
12	Cmax/MIC	4							unsuccessful	1	-			
13	Cmax/MIC	5							unsuccessful	1	-			
14	Cmax/MIC	6							unsuccessful	1	-			
15	Cmax/MIC	7	0.06010	86.0	0.3312	9.885 FIX	7.519	1 FIX	successful	1	-			
16	Cmax/MIC	8							unsuccessful	1	-			
17	T>MIC	1	0.56800	73.8	37.5800	9.885 FIX	4.3 FIX	1 FIX	successful	2	-			
18	T>MIC	2	0.94900	41.3	50.1700	9.885 FIX	4.3 FIX	14.82	successful	2	T>MIC	49	51	52
19	T>MIC	3							unsuccessful	2	-			
20	T>MIC	4							unsuccessful	2	-			
21	T>MIC	5							unsuccessful	2	-			
22	T>MIC	6							unsuccessful	2	-			
23	T>MIC	7	0.63100	75.5	52.8300	9.885 FIX	3.393	1 FIX	successful	2	-			
24	T>MIC	8	0.95000	43.0	49.9200	9.885 FIX	4.468	14.76	successful	2	-			

Reference:

1. Lepak AJ, Zhao M, VanScoy B et al. In vivo pharmacokinetics and pharmacodynamics of ZTI-01 (fosfomycin for injection) in the neutropenic murine thigh infection model against Escherichia coli, Klebsiella pneumoniae, and Pseudomonas aeruginosa. *Antimicrob Agents Chemother* 2017; **61**: 10.1128/aac. 00476-17.
2. XIAO Xm, XIAO Yh. Pharmacokinetics/pharmacodynamics of antofloxacin hydrochloride in a neutropenic murine thigh model of Staphylococcus aureus infection. *Acta Pharmacol Sin* 2008; **29**: 1253-60.
3. Ji X-w, Xue F, Kang Z-s et al. Model-informed drug development, pharmacokinetic/pharmacodynamic cutoff value determination, and antibacterial efficacy of benapenem against Enterobacteriaceae. *Antimicrob Agents Chemother* 2020; **64**: 10.1128/aac. 01751-19.
4. Takata T, Shimizu A, Sakakibara S et al. Optimization of dose and dose regimen of biapenem based on pharmacokinetic and pharmacodynamic analysis. *J Infect Chemother* 2004; **10**: 76-85.
5. Nakamura R, Ito-Horiyama T, Takemura M et al. In vivo pharmacodynamic study of cefiderocol, a novel parenteral siderophore cephalosporin, in murine thigh and lung infection models. *Antimicrob Agents Chemother* 2019; **63**: 10.1128/aac. 02031-18.
6. Takemura W, Tashiro S, Hayashi M et al. Cefmetazole as an alternative to carbapenems against extended-spectrum beta-lactamase-producing Escherichia coli infections based on in vitro and in vivo pharmacokinetics/pharmacodynamics experiments. *Pharm Res* 2021; **38**: 1839-46.
7. Guo C, Liao X, Wang M et al. In vivo pharmacodynamics of cefquinome in a neutropenic mouse thigh model of Streptococcus suis serotype 2 at varied initial inoculum sizes. *Antimicrob Agents Chemother* 2016; **60**: 1114-20.
8. Shan Q, Liang C, Wang J et al. In vivo activity of cefquinome against Escherichia coli in the thighs of neutropenic mice. *Antimicrob Agents Chemother* 2014; **58**: 5943-6.
9. Shan Q, Wang J. Activity of cefquinome against extended-spectrum β-lactamase-producing Klebsiella pneumoniae in neutropenic mouse thigh model. *J Vet Pharmacol Ther* 2017; **40**: 392-7.
10. Wang J, Shan Q, Ding H et al. Pharmacodynamics of cefquinome in a neutropenic mouse thigh model of Staphylococcus aureus infection. *Antimicrob Agents Chemother* 2014; **58**: 3008-12.
11. Craig W, Andes D. In vivo pharmacodynamics of ceftobiprole against multiple bacterial pathogens in murine thigh and lung infection models. *Antimicrob Agents Chemother* 2008; **52**: 3492-6.
12. Craig W, Andes D. In vivo activities of ceftolozane, a new cephalosporin, with and without tazobactam against Pseudomonas aeruginosa and Enterobacteriaceae, including strains with extended-spectrum β-lactamases, in the thighs of neutropenic mice. *Antimicrob Agents Chemother* 2013; **57**: 1577-82.
13. Dudhani RV, Turnidge JD, Coulthard K et al. Elucidation of the pharmacokinetic/pharmacodynamic determinant of colistin activity against Pseudomonas aeruginosa in murine thigh and lung infection models. *Antimicrob Agents Chemother* 2010; **54**: 1117-24.
14. Dudhani RV, Turnidge JD, Nation RL et al. f AUC/MIC is the most predictive pharmacokinetic/pharmacodynamic index of colistin against Acinetobacter baumannii in murine thigh and lung infection models. *J Antimicrob Chemother* 2010; **65**: 1984-90.
15. Zhao M, Lepak AJ, Marchillo K et al. In vivo pharmacodynamic target assessment of eravacycline against Escherichia coli in a murine thigh infection model. *Antimicrob Agents Chemother* 2017; **61**: 10.1128/aac. 00250-17.

16. Tashiro S, Hayashi M, Takemura W et al. Pharmacokinetics/pharmacodynamics evaluation of flomoxef against extended-spectrum beta-lactamase-producing Escherichia coli in vitro and in vivo in a murine thigh infection model. *Pharm Res* 2021; **38**: 27-35.
17. Roelofsen EE, de Winter B, van der Spek H et al. Pharmacodynamics of Flucloxacillin in a Neutropenic Murine Thigh Infection Model: A Piece of the Puzzle towards Evidence-Based Dosing. *Antibiotics* 2022; **11**: 1049.
18. Chavan R, Naphade B, Waykar B et al. Investigations on In Vivo Pharmacokinetic/Pharmacodynamic Determinants of Fosfomycin in Murine Thigh and Kidney Infection Models. *Microb Drug Resist* 2023; **29**: 18-27.
19. Andes D, Craig W. Pharmacodynamics of the new des-f (6)-quinolone garenoxacin in a murine thigh infection model. *Antimicrob Agents Chemother* 2003; **47**: 3935-41.
20. Andes D, Craig W. Pharmacodynamics of the new fluoroquinolone gatifloxacin in murine thigh and lung infection models. *Antimicrob Agents Chemother* 2002; **46**: 1665-70.
21. Bulik CC, Okusanya ÓO, Lakota EA et al. Pharmacokinetic-pharmacodynamic evaluation of gepotidacin against Gram-positive organisms using data from murine infection models. *Antimicrob Agents Chemother* 2017; **61**: 10.1128/aac. 00115-16.
22. Ferrari L, Iavarone L, Braggio S et al. In vitro and in vivo pharmacokinetics-pharmacodynamics of GV143253A, a novel trinem. *Antimicrob Agents Chemother* 2003; **47**: 770-6.
23. Wicha WW, Craig WA, Andes D. In vivo pharmacodynamics of lefamulin, the first systemic pleuromutilin for human use, in a neutropenic murine thigh infection model. *J Antimicrob Chemother* 2019; **74**: iii5-iii10.
24. Growcott E, Cariaga T, Morris L et al. Pharmacokinetics and pharmacodynamics of the novel monobactam LYS228 in a neutropenic murine thigh model of infection. *J Antimicrob Chemother* 2019; **74**: 108-16.
25. Kristoffersson AN, David-Pierson P, Parrott NJ et al. Simulation-based evaluation of PK/PD indices for meropenem across patient groups and experimental designs. *Pharm Res* 2016; **33**: 1115-25.
26. Fratoni AJ, Nicolau DP, Kuti JL. Minocycline pharmacodynamics against Stenotrophomonas maltophilia in the neutropenic murine infection model: implications for susceptibility breakpoints. *J Antimicrob Chemother* 2022; **77**: 1052-60.
27. Melchers M, Teague J, Warn P et al. Pharmacokinetics and pharmacodynamics of murepavadin in neutropenic mouse models. *Antimicrob Agents Chemother* 2019; **63**: 10.1128/aac. 01699-18.
28. Zhao M, Lepak AJ, Marchillo K et al. In vivo pharmacodynamic characterization of a novel odilorhabdin antibiotic, NOSO-502, against Escherichia coli and Klebsiella pneumoniae in a murine thigh infection model. *Antimicrob Agents Chemother* 2018; **62**: 10.1128/aac. 01067-18.
29. Andes D, Craig W, Nielsen L et al. In vivo pharmacodynamic characterization of a novel plectasin antibiotic, NZ2114, in a murine infection model. *Antimicrob Agents Chemother* 2009; **53**: 3003-9.
30. Umezaki Y, Matsumoto K, Ikawa K et al. Concentration-Dependent Activity of Pazufloxacin against Pseudomonas aeruginosa: An In Vivo Pharmacokinetic/Pharmacodynamic Study. *Antibiotics* 2022; **11**: 982.
31. Lepak AJ, Wang W, Andes DR. Pharmacodynamic evaluation of MRX-8, a novel polymyxin, in the neutropenic mouse thigh and lung infection models against Gram-negative pathogens. *Antimicrob Agents Chemother* 2020; **64**: 10.1128/aac. 01517-20.
32. Andes D, Craig W. Pharmacodynamics of a new cephalosporin, PPI-0903 (TAK-599), active against methicillin-resistant Staphylococcus aureus in murine thigh and lung infection

models: identification of an in vivo pharmacokinetic-pharmacodynamic target. *Antimicrob Agents Chemother* 2006; **50**: 1376-83.
33. Hirai J, Hagihara M, Kato H et al. Investigation on rifampicin administration from the standpoint of pharmacokinetics/pharmacodynamics in a neutropenic murine thigh infection model. *J Infect Chemother* 2016; **22**: 387-94.
34. Griffith DC, Rodriguez D, Corcoran E et al. Pharmacodynamics of RWJ-54428 against Staphylococcus aureus, Streptococcus pneumoniae, and Enterococcus faecalis in a neutropenic mouse thigh infection model. *Antimicrob Agents Chemother* 2008; **52**: 244-7.
35. Yokoyama Y, Matsumoto K, Ikawa K et al. Pharmacokinetic/pharmacodynamic evaluation of sulbactam against Acinetobacter baumannii in in vitro and murine thigh and lung infection models. *Int J Antimicrob Agents* 2014; **43**: 547-52.
36. Hegde SS, Okusanya OO, Skinner R et al. Pharmacodynamics of TD-1792, a novel glycopeptide-cephalosporin heterodimer antibiotic used against Gram-positive bacteria, in a neutropenic murine thigh model. *Antimicrob Agents Chemother* 2012; **56**: 1578-83.
37. Liu X, Tashiro S, Igarashi Y et al. Differences in pharmacokinetic/pharmacodynamic parameters of tedizolid against VRE and MRSA. *Pharm Res* 2023; **40**: 187-96.
38. Watanabe E, Matsumoto K, Ikawa K et al. Pharmacokinetic/pharmacodynamic evaluation of teicoplanin against Staphylococcus aureus in a murine thigh infection model. *J Glob Antimicrob Resist* 2021; **24**: 83-7.
39. Hegde SS, Reyes N, Wiens T et al. Pharmacodynamics of telavancin (TD-6424), a novel bactericidal agent, against gram-positive bacteria. *Antimicrob Agents Chemother* 2004; **48**: 3043-50.
40. Sugihara K, Sugihara C, Matsushita Y et al. In vivo pharmacodynamic activity of tomopenem (formerly CS-023) against Pseudomonas aeruginosa and methicillin-resistant Staphylococcus aureus in a murine thigh infection model. *Antimicrob Agents Chemother* 2010; **54**: 5298-302.
41. Louie A, Liu W, Kulawy R et al. In vivo pharmacodynamics of torezolid phosphate (TR-701), a new oxazolidinone antibiotic, against methicillin-susceptible and methicillin-resistant Staphylococcus aureus strains in a mouse thigh infection model. *Antimicrob Agents Chemother* 2011; **55**: 3453-60.
42. Hagihara M, Kato H, Uchida S et al. The first report on pharmacokinetic/pharmacodynamic study of trimethoprim/sulfamethoxazole against staphylococcus aureus with a neutropenic murine thigh infection model. *Chemotherapy* 2020; **64**: 224-32.
43. Lepak AJ, Parhi A, Madison M et al. In vivo pharmacodynamic evaluation of an FtsZ inhibitor, TXA-709, and its active metabolite, TXA-707, in a murine neutropenic thigh infection model. *Antimicrob Agents Chemother* 2015; **59**: 6568-74.
44. Andes D, Craig W. Pharmacodynamics of a new streptogramin, XRP 2868, in murine thigh and lung infection models. *Antimicrob Agents Chemother* 2006; **50**: 243-9.
45. Vogelman B, Gudmundsson S, Leggett J et al. Correlation of antimicrobial pharmacokinetic parameters with therapeutic efficacy in an animal model. *J Infect Dis* 1988; **158**: 831-47.
46. He P, Li X, Guo X et al. Pharmacokinetics and pharmacodynamics of a novel vancomycin derivative LYSC98 in a murine thigh infection model against Staphylococcus aureus. *Infect Drug Resist* 2023: 1019-28.
47. van den Berg S, Attwood MG, Griffin P et al. Pharmacodynamics of NOSO-502 studied in vitro and in vivo: determination of the dominant pharmacodynamic index driver. *J Antimicrob Chemother* 2025: dkae469.
48. Eguchi K, Kanazawa K, Eriguchi Y et al. Pharmacodynamics of SMP-601 (PTZ601) against vancomycin-resistant Enterococcus faecium and methicillin-resistant Staphylococcus

aureus in neutropenic murine thigh infection models. *Antimicrob Agents Chemother* 2009; **53**: 3391-8.
49. Andes D, Van Ogtrop M, Peng J et al. In vivo pharmacodynamics of a new oxazolidinone (linezolid). *Antimicrob Agents Chemother* 2002; **46**: 3484-9.
50. Van Wart SA, Andes DR, Ambrose PG et al. Pharmacokinetic–pharmacodynamic modeling to support doripenem dose regimen optimization for critically ill patients. *Diagn Microbiol Infect Dis* 2009; **63**: 409-14.
